# Ionic gel-mediated synthesis of nanostructured chiral 2D halide perovskites with amplified chiroptical response

**DOI:** 10.1038/s41467-026-77274-w

**Published:** 2026-08-29

**Authors:** Do-Kyoung Lee, Raphael F. Moral, Finn Babbe, Tim Kodalle, Fabrice Roncoroni, Harishankar Jayakumar, Kun Zhou, Yuanyuan Zhou, Yen Jea Lee, Brooks A. Abel, Craig P. Schwartz, Carolin M. Sutter-Fella

**Affiliations:** 1 Department of Physics & Astronomy, University of Nevada, Las Vegas, Las Vegas, NV USA; 2 Molecular Foundry Division, Lawrence Berkeley National Laboratory, Berkeley, CA USA; 3 Brazilian Synchrotron Light Laboratory (LNLS), Brazilian Center for Research in Energy and Materials (CNPEM), Campinas, SP Brazil; 4 Chemical Sciences Division, Lawrence Berkeley National Laboratory, Berkeley, CA USA; 5 Advanced Light Source, Lawrence Berkeley National Laboratory, Berkeley, CA USA; 6 Department of Chemical and Biological Engineering, The Hong Kong University of Science and Technology, Kowloon, Hong Kong SAR China; 7 Energy Institute, The Hong Kong University of Science and Technology, Hong Kong, China; 8 Otto Poon Centre for Climate Resilience and Sustainability, The Hong Kong University of Science and Technology, Hong Kong, China; 9 Materials Sciences Division, Lawrence Berkeley National Laboratory, Berkeley, CA USA; 10 Department of Chemistry, University of California, Berkeley, Berkeley, CA USA; 11 Advanced Photon Source, Argonne National Laboratory, Lemont, IL USA

**Keywords:** Materials science, Materials chemistry, Optical materials and structures

## Abstract

Chiroptical properties in metal halide perovskites typically arise from asymmetric crystal structures induced by chiral organic molecules during synthesis or chiral additives. However, the synthesis of two-dimensional chiral halide perovskites with sufficiently high dissymmetry factors for practical applications remains challenging. Herein, we present a two-step ionic gel-mediated nucleation and recrystallization reaction, enabling the synthesis of nanostructured chiral halide perovskite films exhibiting large intrinsic circular dichroism dissymmetry factors (*g*_CD_) up to 10^−2^. The ionic gel mediates the reaction between lead(II) iodide (PbI_2_) and *R*/*S*-methylbenzylammonium iodide (*R*/*S*-MBAI), to facilitate the formation of higher-order iodoplumbate PbI_3_^-^ and oligomeric Pb-I-MBA species, which recrystallize during heat treatment as anisotropic nanostructures without preferential orientation. Ionic gel-mediated films exhibit a very pronounced peak splitting originating from a well-defined exciton fine structure and narrow photoluminescence linewidth. This is indicative of the presence of electron-hole exchange interactions and minimized structural disorder in the material leading to significantly amplified chiroptical response. This work demonstrates a synthesis route for nanostructured chiral MBA_2_PbI_4_ films, whose unique structural feature with reduced crystalline imperfection enables amplified circular dichroism and *g*_CD_ response.

## Abstract (web summary)

An ionic gel-mediated method to synthesize nanostructured 2D chiral halide perovskite films was developed. By minimizing crystal defects, this breakthrough dramatically amplifies chiroptical properties for next-generation technologies.

## Introduction

Low-dimensional chiral halide perovskites have garnered substantial attention as promising materials for a wide range of advanced applications, such as spintronics, quantum computing, and chiroptical electronic devices^1–4^, thanks to their superior properties such as strong spin-orbit coupling, chiral-induced spin selectivity, and chirality-dependent spin polarization^2,5–7^. These materials are derived from the well-known three-dimensional (3D) perovskite structure with chemical formula ABX_3_, where A is an organic or inorganic monovalent cation (e.g., methylammonium, formamidinium, or cesium), B a divalent metal cation (e.g., Pb^2+^ or Sn^2+^), and X a halide anion (e.g., Cl^−^, Br^−^, or I^−^) but are engineered to have reduced dimensionality with chemical formula (A’)_2_(A)_n−1_B_n_X_3n+1_ (where A’ is a bulky and/or long-chain organic cation)^8^. Low-dimensional chiral halide perovskites consist of bulky chiral organic cations and an inorganic lead halide framework^2,9^. Chirality in these materials is transferred from the chiral organic cation to the inorganic perovskite framework, leading to symmetry breaking of the inorganic octahedral structure as well as splitting of the electronic bands^10–12^. The first reported chiral 2D halide perovskite thin film material is *R*-/*S*-methylbenzylammonium-based lead iodide, where *R*-/*S*-methylbenzylammonium (*R*-/*S*-MBA^+^) is used as a chiral organic component. Due to its large ionic radii, MBA cations interact with the [PbX_6_]^4−^ inorganic framework, adapting one-dimensional (1D, MBAPbX_3_) and two-dimensional (2D, MBA_2_PbX_4_) phases depending on the stoichiometry of the MBA cations.

Although compositionally indistinguishable, chiral enantiomers give rise to differential absorption of right- and left-handed circularly polarized light (CPL). This property is related to electronic and magnetic transition dipole moments and is referred to as circular dichroism (CD)^13–15^. The reported dissymmetry factors extracted from CD measurements (*g*_CD_) for chiral 2D halide perovskites tend to be low compared to chiral organic molecules or nanocrystals, often on the order of 10^−3^ or lower^16–19^. For practical applications, there is a need to achieve higher dissymmetry factors, aiming to approach total CPL selectivity (i.e., |*g*_CD_| = 2)^20^.

Little work has been done to understand the relationship between the crystallization process of chiral 2D halide perovskites and their chiroptical properties. The effect of morphology on chiroptical properties have been studied^19,21,22^, but it is still unclear. Ahn et al. found concentration-dependent changes of morphology, crystal orientation, and CD using a one-step spin coating process^9^. In the conventional one-step method, chiral 2D halide perovskite films are formed through dissolving pre-synthesized single crystals of the target material and subsequent spin-coating, followed by annealing. This method has the drawback of requiring the preparation of high-quality single crystals from strong hydrohalic acids, and the precursor is limited to the exact single-crystal stoichiometry. It has been widely used in the field of 3D metal halide perovskites, however, utilizing successful strategies such as non-stoichiometric and compositional precursor engineering approaches remains limited in low dimensional chiral halide perovskites to improve film quality and control crystallographic orientation^23–25^.

Here, we report an order of magnitude higher *g*_CD_ for 2D *R*/*S*-MBA_2_PbI_4_ thin films than previously reported for the pristine materials, achieved through nanostructured thin film growth via a two-step sequential deposition method. Conversion of inorganic PbI_2_ thin films was controlled through an ionic gel-mediated (IGM) recrystallization process, where *R*-/*S*-/racemic-MBAI organic salts in isopropyl alcohol (IPA) form a gel-like intermediate and PbI_3_^−^ iodoplumbate species. This is followed by templating the gel initiated from 1D oligomeric Pb-I-MBA species and preferred anisotropic 1D growth starting from the solid/gel interface. This work provides mechanistic insights into a synthetic approach for nanostructured chiral 2D halide perovskite films, leading to a significant boost in a CD dissymmetry factor due to stronger electron-hole exchange interactions and minimized structural disorder.

## Results and discussion

### Ionic gel-mediated synthesis of chiral 2D halide perovskite films

**Fig. 1:**
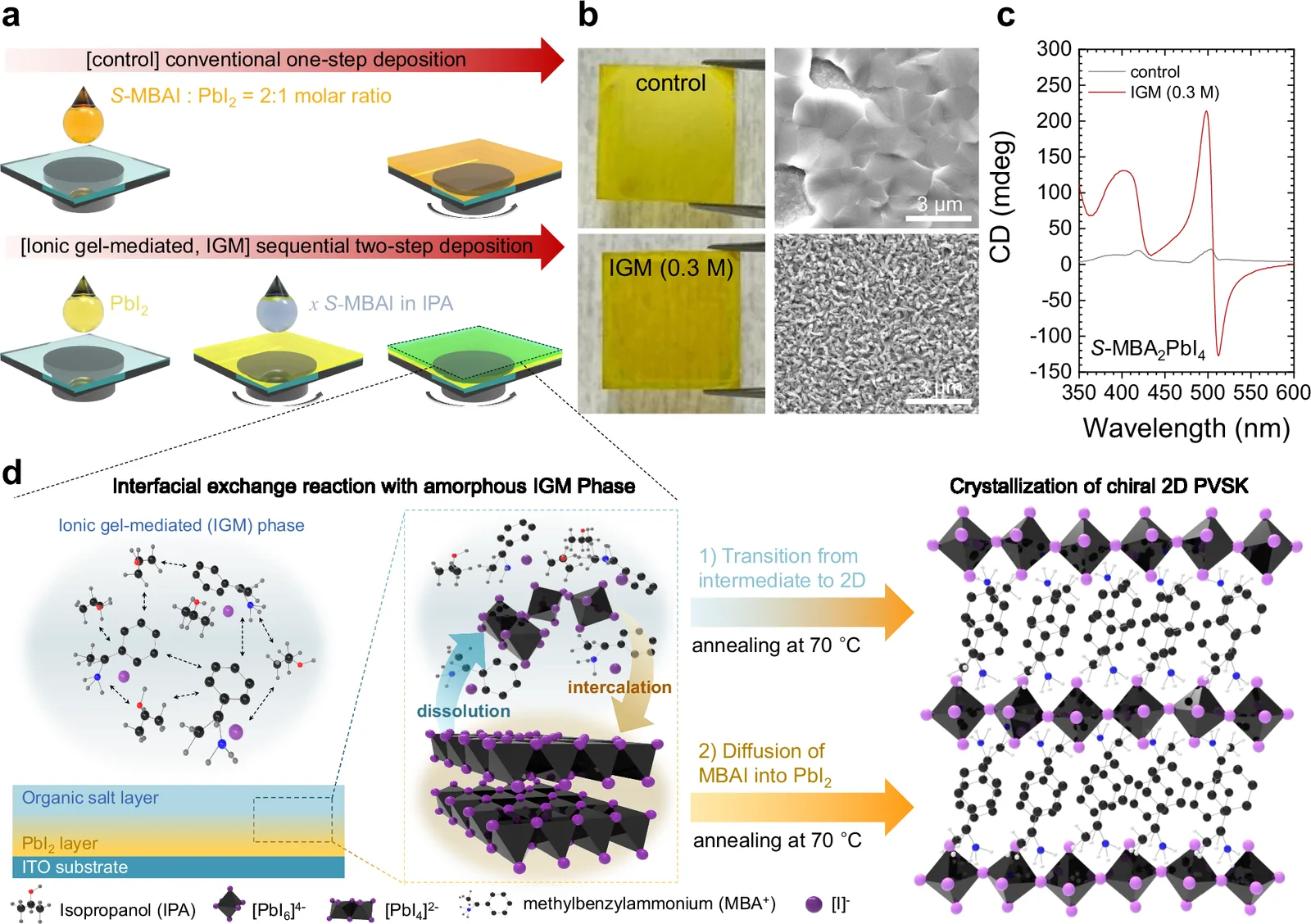
Deposition methods for chiral 2D MBA2PbI4 perovskite films. **a** Schematic illustration of the film deposition procedures for conventional one-step (control) and sequential two-step (IGM, ionic gel-mediated) methods. *S*-MBAI is (*S*)-(−)-α-methylbenzylammonium iodide. **b** Photos and scanning electron microscopy (SEM) images. **c** Circular dichroism (CD) spectra for control and IGM (0.3 M) *S*-MBA_2_PbI_4_ films. **d** Illustration depicting the IGM crystallization through interfacial exchange reaction occurring between the PbI_2_ film and the MBAI gel.

To synthesize the chiral 2D MBA_2_PbI_4_ thin films, we utilized one-step and two-step deposition techniques, schematically illustrated in Fig. 1a. For the one-step method, the coating solution containing both chiral *S*-methylbenzylammonium iodide (*S*-MBAI) and PbI_2_ precursors, with a 2:1 molar ratio dissolved in a mixture of dimethylformamide (DMF) and dimethyl sulfoxide (DMSO) was spin-coated onto an indium-doped tin oxide (ITO) substrate, and then annealed at 70 °C for 10 min. For the two-step method, a 0.5 M PbI_2_ solution was first spin-coated onto the bare ITO substrate, followed by thermal annealing at 70 °C for 1 min, forming an approximately 90 nm thick PbI_2_ film (Supplementary Fig. 1). Second, an *S*-MBAI solution (*S*-MBAI salts dissolved in IPA) was dropped onto the spinning PbI_2_ substrate and subsequently annealed at 70 °C for 10 min. The films will be referred to as “control” and “IGM” for those using the one-step and two-step deposition methods, respectively. The control shows an opaque appearance with inhomogeneous, large macroscopic crystals, indicating poor coverage and rough film morphology (Fig. 1b). In sharp contrast, the IGM film exhibits a semitransparent and reflective film appearance, indicating a homogeneous and smooth film surface, with uniform rod-like nanoscale crystallites. The average thickness is 276.87 nm for control and 239.39 nm for 0.3 M IGM, which is not a significant difference (Supplementary Fig. 2). It indicates that the optical transparency of the films are related to their coverage. It is in good agreement with the enhanced optical scattering absorption above 550 nm in absorbance spectra (Supplementary Fig. 3a). Phase purity was confirmed for both control and IGM samples by X-ray diffraction (XRD) measurements (Supplementary Fig. 3b). Impressively, the CD signal (Fig. 1c) is enhanced by one order of magnitude at the excitonic peak (~500 nm) in the case of 0.3 M IGM thin film (Supplementary Fig. 3a).

We hypothesize that the IGM crystallization plays a key role in the enhancement of the CD response. In Fig. 1d, we propose a crystallization pathway of 2D chiral halide perovskite films via IGM synthesis. It was found that an amorphous ionic gel (IG) phase is formed upon evaporation of the IPA solvent during spin coating and subsequent annealing (Supplementary Fig. 4). According to this basis of our finding, the crystallization mechanism via IGM methods can be expected as following several steps during film formation; (i) When treating *S*-MBAI solution onto the PbI_2_ layer, the treated *S*-MBAI solution partially dissolves the underlying PbI_2_ layer, starting from its surface. The extent of this solubility depends on the concentration of *S*-MBAI solution (Supplementary Fig. 5). For example, in the case of a low *S*-MBAI solution concentration, IG phase consisting of unreacted *S*-MBAI and residual IPA predominantly remains on the as-coated film surface. On the other hand, for 1.0 M IGM condition, the treated *S*-MBAI solution can mostly dissolve the underlying PbI_2_ layer, resulting in predominantly forming the intermediate phase. (ii) During the spin-coating process, the IPA solvent is partially evaporated, promoting the in-situ formation of both gel-like amorphous intermediate (consisting of a complex of PbI_2_, *S*-MBAI, and residual IPA) and IG (consisting of a complex of *S*-MBAI and residual IPA) phases on the surface. (iii) During the annealing process, there are two crystallization processes. The first is that the intermediate phase is directly converted to 2D phase. The second is that *S*-MBAI ion species in IG phase gradually diffuse into the underlying PbI_2_ layer, where the elevated temperature triggers a phase transformation into the 1D (if *S*-MBAI species is insufficient) and/or 2D (if *S*-MBAI species is sufficient) phase based on a diffusion-driven phase conversion mechanism.

**Fig. 2:**
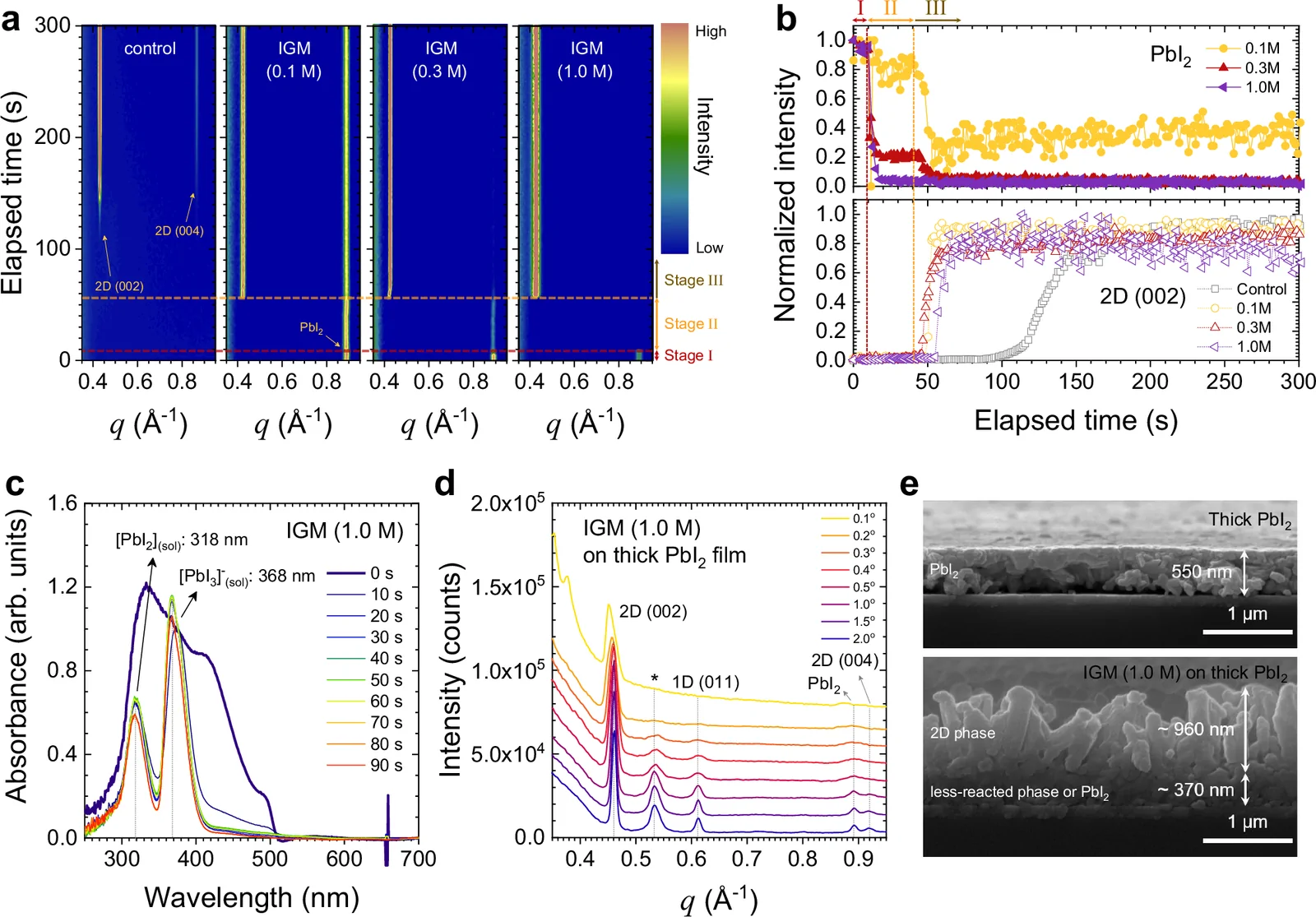
Crystallization pathway of IGM film formation. **a** Contour plots of in-situ GIWAXS during the film formation, taken at an angle of incidence of 2.0°. **b** Time evolution of normalized diffraction peak area corresponding to PbI_2_ (top) and 2D (002) (bottom) phase fractions. **c** Individual in-situ absorbance spectra taken during the spin coating step of an IGM (1.0 M) sample. The 1.0 M *S*-MBAI solution was dynamically dropped on the PbI_2_ film at 5 s after the beginning of the spinning process. **d** depth-dependent GIWAXS diffraction patterns collected for the IGM (1.0 M) sample. **e** Cross-sectional SEM images for thick PbI_2_ (top) and IGM (1.0 M) on thick PbI_2_ (bottom) films.

To unveil the IGM crystallization pathway, we employed synchrotron-based in-situ grazing incidence wide-angle X-ray scattering (GIWAXS) and in-situ ultraviolet–visible (UV–Vis) transmission measurements. Figure 2a, b show the time evolution of the GIWAXS patterns and the diffraction peak intensities for the control and IGM films with varying concentrations of *S*-MBAI (technical details in Supplementary Figs. 4–6). The phase conversion processes were divided into three stages: (I) PbI_2_ film spinning; (II) *S*-MBAI solution dropping; and (III) annealing at 70 °C. The diffraction signals at *q* = 0.46, 0.89, and 0.92 Å^−1^ are corresponding to chiral 2D (002), PbI_2_, and chiral 2D (004) phases, respectively, in agreement with literature reports^9^ (Supplementary Fig. 7). For the control sample, the (002) peak of the chiral 2D phase appeared after approximately 100 s and, slightly later, the corresponding (004) peak. No impurity or unreacted phases such as 1D *S*-MBAPbI_3_ or PbI_2_ were observed. In the IGM cases, a single peak at *q* = 0.89 Å^−1^ confirms the presence of phase-pure PbI_2_ at the beginning of the processes (stage I). From stage I to II, one can observe a rapid drop in the PbI_2_ diffraction signal, followed by a plateau in stage II. This indicates that the IG phase immediately reacted with the underlying PbI_2_ layer but requires thermal activation to completely react. It is noted, for 1.0 M IGM case, that there are no diffraction peaks for any crystalline phases in stage II, indicating that the PbI_2_-containing IGM intermediate phase is amorphous. The total drop from stage I to II scales with the *S*-MBAI concentration, and the highest concentration of 1.0 M shows the steepest PbI_2_ signal decrease. Upon thermal annealing (stage III), the PbI_2_ signal drops to 0—but only if there is sufficient *S*-MBAI present for the complete reaction with PbI_2_. At *S*-MBAI concentrations of 0.3 M or higher, PbI_2_ is fully converted to chiral 2D perovskite phases during annealing. There is unreacted PbI_2_ left for the 0.1 M *S*-MBAI condition because of the *S*-MBAI depletion.

In-situ UV–Vis transmission measurements during spin coating (Fig. 2c and Supplementary Fig. 8) show that the original PbI_2_-related absorption features diminish and a new absorption peak around 368 nm appears a few seconds after the MBAI is deposited. It is well-known that PbI_2_ undergoes complexation in the presence of excess iodide ions to form higher-coordinated plumbate species^26^. Consequently, during IGM crystallization, PbI_2_ undergoes complexation to form soluble PbI_3_^−^ with an absorption feature at 368 nm^27^. The PbI_3_^−^ likely forms a gel intermediate of oligomeric [PbI_3_^−^-MBA^+^] species through coulombic and hydrogen bonding interactions: [PbI_3_]_*n*_^−^_(sol)_ + *n* MBA^+^_(sol)_. During the subsequent spin coating process, IPA solvent evaporates. We hypothesize that templating of the chiral 2D material is initiated from a 1D gel framework of [PbI_3_^−^-MBA^+^]_*n*_ species, promoting anisotropic rod-like growth. The crystal growth is governed by the geometry of colloidal species and crystal structures^23,28^. The [PbI_3_]^−^ species have an one-dimensional polymeric face-sharing octahedral formed by interacting organic cations with their (001) edge^23,29,30^. It is inferred that these structures of oligomeric [PbI_3_^−^-MBA^+^] species can trigger 1D-structured crystal growth. Furthermore, a strong intermolecular interaction of organic components can modulate the directional crystal growth kinetics differently along longer and shorter axes, promoting needle-like shape formation^28^. Turning to the experimental results, at low *S*-MBAI concentrations, the conversion of PbI_2_ is limited to the surface and proceeds via the gel intermediate and MBAI intercalation. In contrast, at higher *S*-MBAI concentrations (e.g., 1.0 M), the IG phase can fully dissolve the underlying PbI_2_. This is followed by the fast growth of randomly oriented rods, similar to single crystal growth in solutions. By controlling the *S*-MBAI concentration, one can modulate the IGM growth and sample morphology. The formation kinetics are significantly slower and more gradual in the control compared to the IGM samples, as extracted from the slopes of the 2D peak intensities in the bottom panel of Fig. 2b. The rapid crystallization in the IGM process is indicative of a fast nucleation process with many nucleation sites, while in the control sample longer growth time likely leads to Ostwald ripening and consequently to the large-grained films discussed in Fig. 1b.

To confirm if PbI_2_ conversion starts from the IG/PbI_2_ interface, we performed GIWAXS measurements as a function of X-ray incidence angle from 0.1 to 2.0°, where shallow angles are surface sensitive while higher angles are more bulk sensitive (Fig. 2d and Supplementary Fig. 9)^31^. At low incidence angles, the PbI_2_ peak is significantly lower in intensity compared to higher angles of incidence, indicating that conversion starts from the surface and proceeds into the bulk upon diffusion during annealing. We were unable to identify the diffraction peak at 0.53 Å^−1^ (denoted with *), but speculate that it is related to an MBA-intercalated phase induced by the MBAI deficiency. Figure 2e illustrates the cross-sectional SEM view of the original PbI_2_ (top) and IGM film (bottom). The bottom cross section clearly shows nanostructures at the upper half and unreacted phases (PbI_2_ and 1D *S*-MBAPbI_3_) at the bottom of the film.

### Anisotropic growth of chiral 2D perovskite nanorods and structural insights

**Fig. 3:**
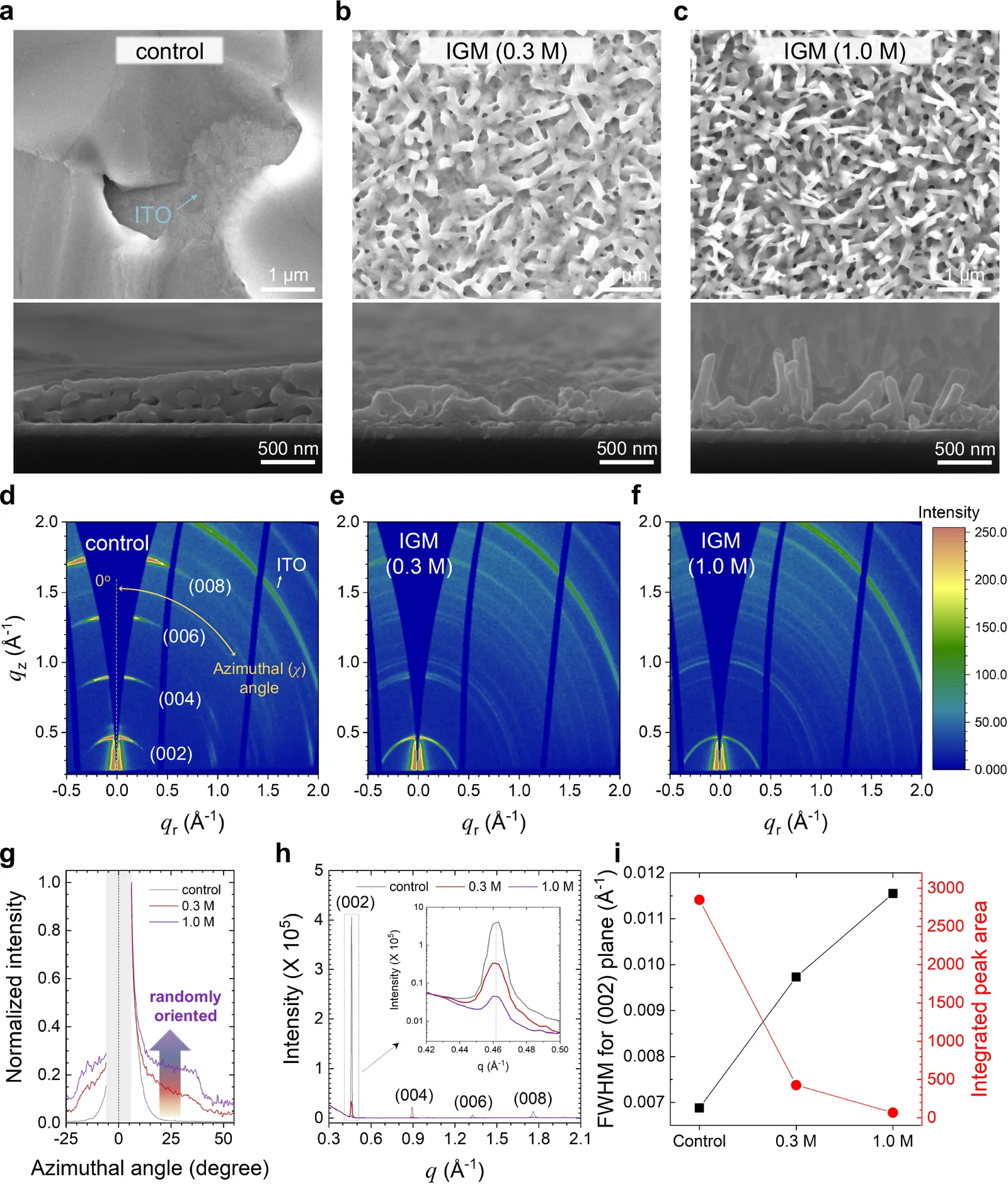
Morphological and structural characterizations of chiral 2D MBA2PbI4 perovskite films. **a–c** Top-view (top) and cross-sectional (bottom) SEM images of the control (**a**) and IGM (**b** and **c**) conditions. **d–f** Two-dimensional GIWAXS patterns in reciprocal space with respect to *q*_r_ and *q*_z_ axes for control (**d**) and IGM (**e** and **f**) films. GIWAXS frames were measured at an X-ray incidence angle of 2.0°. **g** Azimuthal intensity distribution of the (002) peak for 2D chiral halide perovskites shown in this figure (**d**–**f**). The grey area and black dash line are indicative of the missing wedge and out-of-plane position, respectively. **h** Diffraction patterns extracted from GIWAXS. The inset zooms in on the (002) peak region around 0.46 Å^−1^ for control and IGM films. **i** Full width at half maximum (FWHM) and integrated peak area corresponding to the (002) plane shown in this figure (**h**).

Next, we take a closer look at the structural features of the chiral 2D perovskite films for both control and IGM conditions. At an *S*-MBAI concentration between 0.5 and 1.0 M, anisotropic growth was found, resulting in rod-like nanostructures with a width of tens of nm to 100 nm (Fig. 3a–c and Supplementary Figs. 10 and 11). If the concentration is too low, a typical polycrystalline structure is observed with submicrometer grain size; if the concentration is too high (exceeding 1.5 M), the rod-like nanostructures continue growing laterally into micrometer-sized platelets with incomplete substrate coverage. The nanorod morphology, which exhibits similar shape as its single crystals^32–34^, suggests a higher structural order and better crystallinity. In contrast, the control film exhibits crystals with rounded edges and agglomerated particles, as seen in the cross-sectional SEM image (Fig. 3a), indicating there might be substantial crystallographic defects and inhomogeneity. To further evaluate the surface topography of control and IGM (0.3 and 1.0 M) films, we performed atomic force microscopy (AFM) measurements (see Supplementary Figs. 12 and 13, and Supplementary Note 1). These AFM results indicate poor coverage of control film, which is in line with the morphological results (Fig. 1b and Supplementary Fig. 3a).

The 2D GIWAXS patterns of the control and IGM samples reveal distinct diffraction intensity distributions over the azimuthal angle (*χ*) of the chiral 2D perovskite phase (Fig. 3d–f). The control films revealed intense and well-defined scattering patterns with preferred orientation along out-of-plane direction, indicative of the preferred orientation of the inorganic slabs parallel to the substrate and small mosaicity. In contrast, IGM films exhibit scattering patterns along arc segments, indicating more random crystal domain orientation on the film. Minor scattering peaks for 0.3 and 1.0 M conditions match well with the calculated diffraction pattern for *S*-MBA_2_PbI_4_ (Supplementary Fig. 14), in agreement with previous reports^9^. Integration of the (002) lattice plane along the azimuthal angle quantifies the above description, revealing narrower intensity distribution in the control films compared to a wider intensity distribution in IGM films (Fig. 3g). The azimuthal intensity distribution in the latter depends on the *S*-MBAI concentration and increases with increasing concentration. The randomly oriented rod-like nanostructures lead to homogeneous scattering along the azimuthal direction, similar to the diffraction of randomly oriented single-crystal powders (Supplementary Fig. 15). The (002) GIWAXS peak intensity is orders of magnitude higher for the control and can be explained by the macrosized crystal features seen in the SEM images in Fig. 1c. In sharp contrast, as the *S*-MBAI concentration in IPA increases for IGM films, the intensity of the (002) peak is gradually decreasing as a result of the nanoscale crystals and more randomly distributed crystal orientation. This intensity drop is accompanied by an increase in full width at half maximum (FWHM) of the (002) peak (Fig. 3i). Taking together, these results provide evidence that an increase in *S*-MBAI concentration leads to anisotropic growth of nanostructured crystals.

### How anisotropic nanostructured films lead to amplified chiroptical properties

**Fig. 4:**
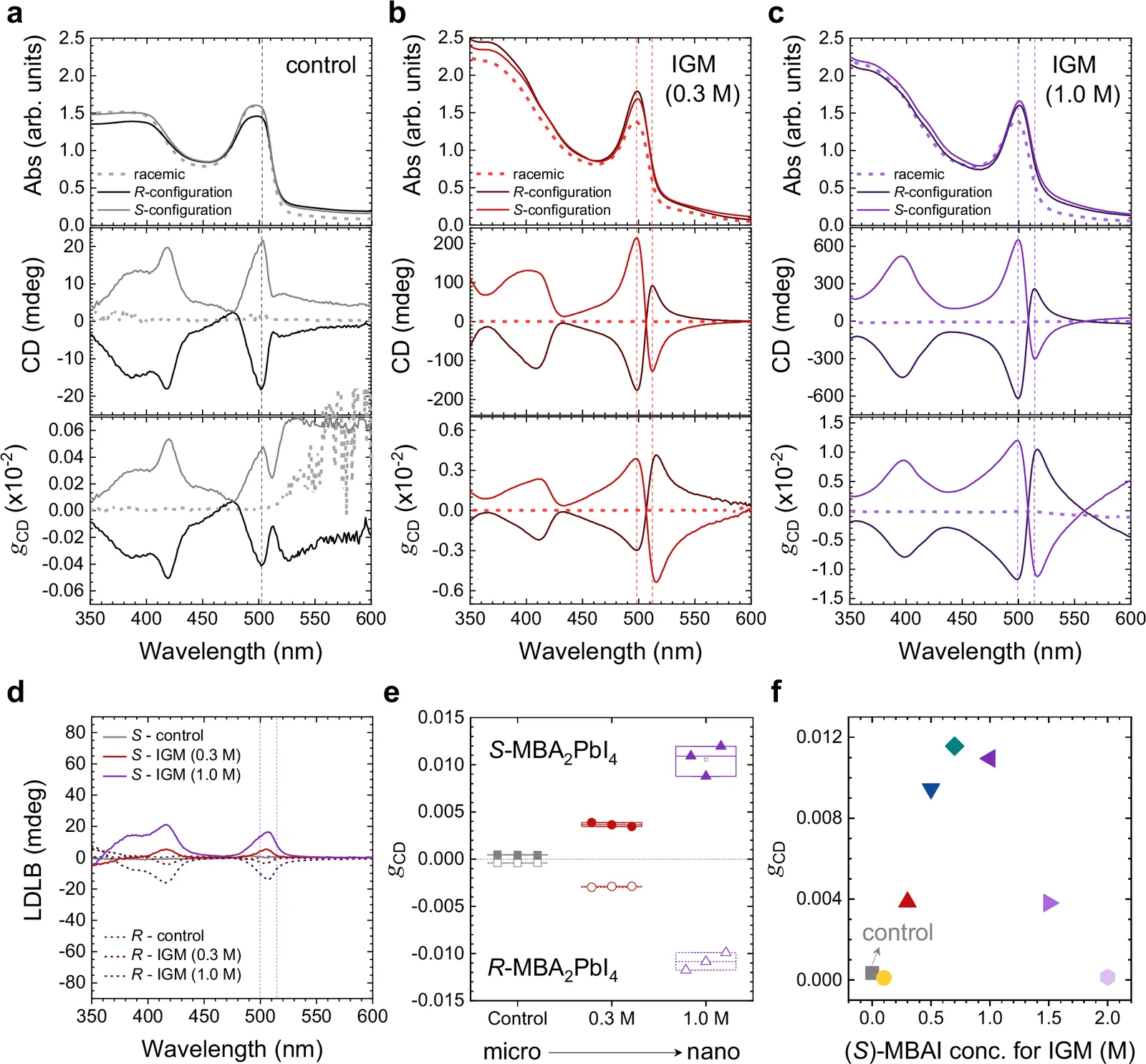
Effect of IGM growth on circular dichroism. **a–c** Absorption spectra (Abs), CD, and *g*_CD_ for control and IGM samples. Chiroptical activity can be tuned by IGM concentration. Vertical lines are a guide for the eye to indicate the CD maxima/minima around the exciton absorption peak. **d** Linear dichroism and linear birefringence (LDLB) contribution to the samples described in (**a–c**). **e** Statistics of the maximum *g*_CD_ at around 500 nm for control and IGM samples. 3 individual samples for each condition were measured. Opened and filled symbols are for *R*- and *S*-MBA_2_PbI_4_, respectively. Box-plot elements are defined as follow: the center line is the median, box limits are the 25th and 75th percentiles, and whiskers define a 1.5 interquartile range. **f** Overview of *g*_CD_ as a function of the concentration of *S*-MBAI solution.

Figure 4a–c depicts absorbance, CD, and *g*_CD_ data. The absorbance spectra (top part) show two distinct features: the excitonic absorption (~ 500 nm) and the continuum (<500 nm). The excitonic absorption region exhibits sharper peaks for the IGM films grown at 0.3 to 1.0 M *S*-MBAI concentrations, pointing to a stronger oscillator strength of the excitonic transitions. The CD minima and maxima amplitudes are centered around the excitonic absorption peak *ca*. 500 nm (Fig. 4a–c). In addition, IGM samples show bisignate CD with the zero-crossing point *ca*. 510 nm, slightly away from the excitonic peak maximum (Fig. 4b, c). The emergence of the bisignate curves is followed by an increase in the amplitude of both negative and positive bands. This is often an indication of exciton coupling, which occurs when two electric transition dipole moments are spatially close enough to couple, resulting in excited state splitting into two nondegenerate levels and bisignate CD response^35,36^. In such cases, the sum of the integrated intensity of each couplet should be zero. However, in this work, the CD spectra for IGM films (Fig. 4b, c) exhibit excitonic peak positions aligned with the excitonic peak in the absorption spectrum (positive couplet for *S*-MBA_2_PbI_4_ or negative couplet for *R*-MBA_2_PbI_4_ in the bisignate CD spectrum). In addition, given that the observed nanorod-shaped crystals in the IGM film are orders of magnitude larger than the typical Bohr radius, exciton coupling is excluded. For these reasons (further details are provided in Supplementary Note 2), we rule out the hypothesis of exciton coupling and speculate that the bisignate CD response might be more attributed to Rashba-type spin splitting of electronic transition states due to spin-orbit coupling (SOC)^37,38^.

To quantitatively assess the influence of crystallization on chiroptical activity, we calculate the dissymmetry factor from CD spectra using *g*_CD_ = CD/(32980 × *A*) equation, where *A* is the absorbance corresponding to CD spectra. CD spectra were measured from both the front and back sides to account for interference of macroscopic anisotropies; i.e., linear dichroism and linear birefringence (LDLB) effects^39–41^. The LDLB contributions are negligible (Fig. 4d) and *g*_CD_ is corrected for any contributions. Both control and IGM syntheses are very reproducible, as illustrated by the *g*_CD_ distribution shown in Fig. 4e and Supplementary Figs. 16 and 17 (Supplementary Fig. 18 for independent batches). We found excellent chiroptical properties and significantly amplified intrinsic *g*_CD_ for all IGM films with MBAI in IPA concentrations between 0.3 and 1.0 M. Noteworthy, IGM samples show almost two orders of magnitude enhancement in *g*_CD_ compared to the control (3.5 × 10^−4^ to 1.2 × 10^−2^, Fig. 4f and Supplementary Figs. 19 and 20). This gain in *g*_CD_ is independent of the *R*- and *S*-configuration of the chiral 2D perovskite.

We hypothesize that there are two major factors contributing to the amplified CD response in IGM films: the enhanced light-matter interaction via textured morphology with randomly oriented crystals as well as enhanced electrodynamic coupling of transition dipoles. First, these isotropic distributions of crystalline domains can substantially contribute to the enhanced CD response. When CPL passes the textured or patterned film, the incident light can be refracted depending on the crystal’s geometry^22^. When the CPL passes through randomly oriented crystals, strong and highly efficient light-matter interactions occur by coupling the electric field vector of the light with a more diverse population of crystalline domains, considering all possible interaction geometries^22,42^. Therefore, isotropic orientation of nanorods leads to more intense CD response. To further investigate the influence of the structural features of nanostructured films on CD response, we prepared the films via one-step deposition method using acetonitrile (ACN)- and *N*,*N*-dimethylformamide (DMF)-based *S*-MBA_2_PbI_4_ precursor solutions. While poor coverage and rough surface are observed in the DMF film, the ACN film shows highly semi-transparent, uniform, and fully-covered morphology (Supplementary Fig. 21). The corresponding CD response is shown in Supplementary Fig. 22. Although the CD response intensity and *g*_CD_ for ACN films are enhanced owing to better coverage and surface morphology, those values are still low (CD < 100 mdeg, *g*_CD_ < 10^−3^) compared to IGM films (CD > 600 mdeg, *g*_CD_ > 10^−2^). This indicates that reducing film roughness and improving coverage are not sufficient to significantly improve the chiroptical response. Second, the interaction strength of electrodynamic coupling of transition dipoles (excitons) can be enhanced by higher structural order and homogeneity of crystallite domains, as observed in the IGM films. Lower defects and homogeneous crystallite domains with smaller crystal sizes in IGM films can induce the enhancement of the rotational strength, resulting in an improved dissymmetry factor *g*_CD_ (Supplementary Note 3). To support this correlation, density functional theory (DFT) calculations were carried out to quantify octahedral distortion, where the angular difference of Pb-I-Pb bonding between an inequivalent pair of neighboring octahedra was denoted as ***Δβ*** (see Supplementary Fig. 23). The calculated ***Δβ*** values for various defect types are summarized in Supplementary Table 1. Given the decreased ***Δβ*** values for defect-induced conditions (6.01° for V_I_, 4.73° for V_Pb_, 5.11° for V_MBA_, 4.84° for I_i_, and 4.38° for Pb_i_), the presence of defects can attenuate the local geometric asymmetry, leading to loss of its chirality. The density of states calculation results with varying defect conditions are shown in Supplementary Fig. 23e–h. Since Rashba spin splitting relies on inversion symmetry breaking of crystal structures, defect-induced structural asymmetry relaxation corresponds directly to a reduction in energy level splitting, as observed in Supplementary Fig. 23e–h. In addition, these findings in the calculated density of states provide an explanation for a strong bisignate CD appearance in IGM films, indicating that the CD features result from less defects. Furthermore, CD spectra simulations (Supplementary Fig. 23i–l) corroborate that the chiroptical response is attenuated in the presence of defects due to the reduced structural asymmetry, which is good agreement with the calculated ***Δβ*** results. The correlation between the crystallite homogeneity and the amplified CD response is further discussed in the next section based on temperature-dependent PL linewidth analysis.

**Fig. 5:**
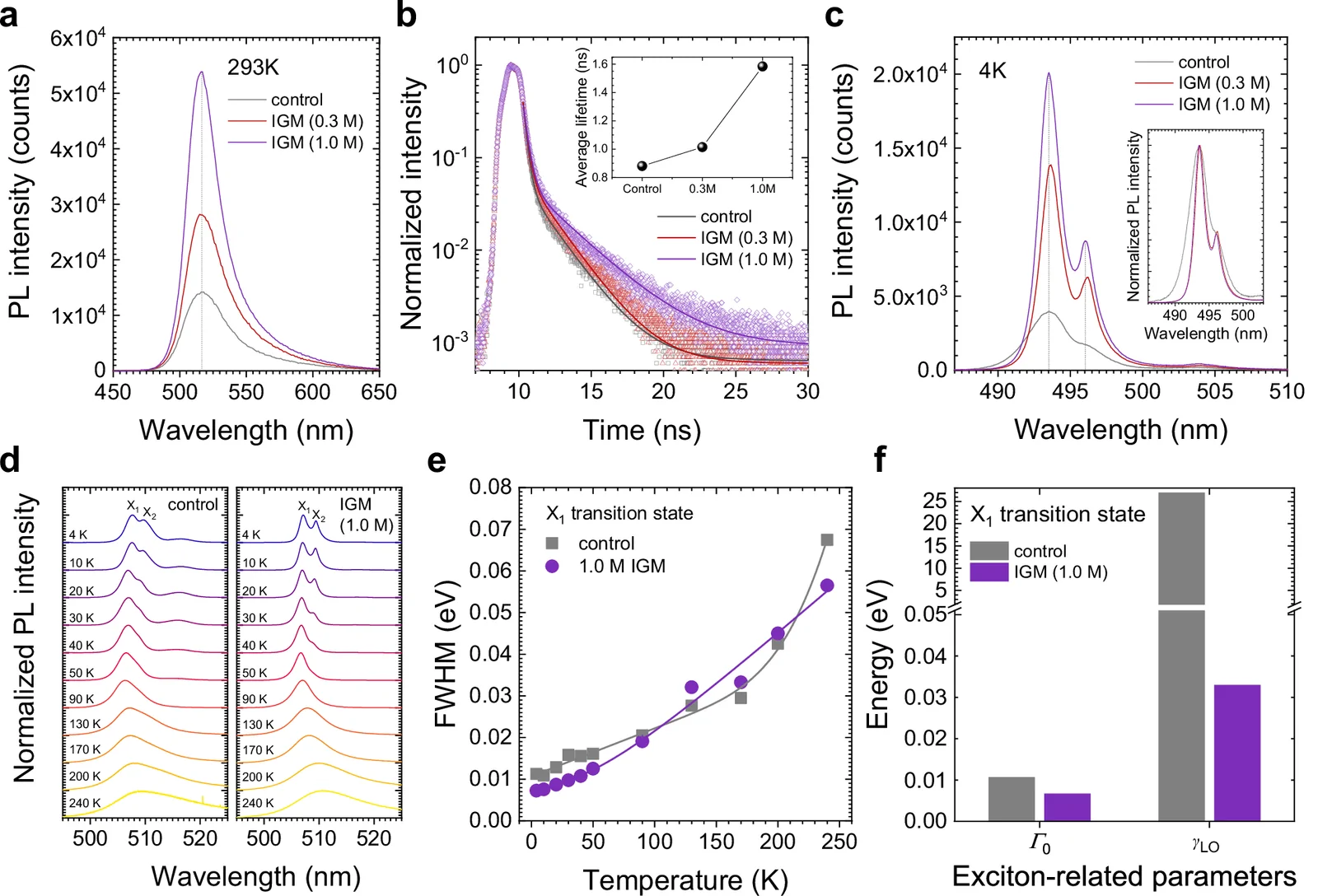
Effect of IGM growth on photon emission properties. **a** Steady-state PL spectra and **b** time-resolved PL decay with average carrier lifetime for control and IGM samples. Both measurements were performed at room temperature. **c–f** Low-temperature PL data and analyses. **c** PL spectra taken at 4 K. **d** Temperature-dependent PL spectra taken from 240 to 4 K. X_1_ and X_2_ refer to individual excitonic emissions discernible at low temperatures. **e** Analysis of temperature-dependent PL linewidth broadening (FWHM) associated with the X_1_ transition state. The solid lines indicate the fitting results applied to the experimental data using the linewidth broadening equation *Γ*(*T*) = *Γ*_0_ + γ_ac_*T* + γ_LO_(exp(*E*_LO_/*k*_*B*_*T*)−1)^−1^ as denoted (Eq. (1)). **f** Corresponding exciton-related *Γ*_0_ (inhomogeneity-related) and *γ*_LO_ (LO phonons-related) parameters contributing to the PL peak broadening.

To further evaluate the beneficial effect of the IGM nanocrystalline structure on the excitonic transitions and oscillator strength, we performed temperature-dependent and time-resolved PL (TRPL) measurements. Room temperature PL measurements display monotonously increasing PL intensity from control to 0.3 and 1.0 M IGM samples (Fig. 5a). The higher PL intensity indicates increased contribution of radiative recombination, typical of less defective sites. TRPL confirms an increase in charge carrier lifetime by 80%, from 0.88 ns for the control to 1.58 ns for the 1.0 M IGM film (Fig. 5b). The parameters obtained from fitted TRPL spectra are summarized in Supplementary Table 2, indicating that the number of defects is reduced in nanostructured IGM films. We also tested environmental stability as shown in Supplementary Fig. 24 where the chiral perovskite films were exposed to the ambient air with ~ 70% relative humidity for up to 192 h. It suggests the enhanced moisture stability of the IGM films can be attributed to less defects and improved crystal quality. Next, low-temperature PL was measured at 4 K (Fig. 5c). These measurements reveal a weak PL splitting of the excitonic emissions for the control, but a clear splitting for the IGM films, which originates from a more well-defined exciton fine structure, potentially stemming from crystal domain homogeneity and minimized structural disorder in the material^43^.

The consistent PL peak splitting energy for both control and IGM films (12.8 meV) suggests that the spin-split states are preserved with identical electron-hole exchange interaction strength. This indicates that the fundamental mechanism responsible for this spin splitting induced by SOC is not significantly altered by the size and shape perturbations of chiral halide perovskite crystals. Even so, the imperfection induced by defects and lattice disorder can influence the differential absorption of CPL and exciton formation associated with different spin states, ultimately impacting the rotational strength of CD transitions.

Lastly, to get a deeper understanding of exciton-phonon interactions, we analyze the different contributions to linewidth broadening *Γ*(*T*) by extracting the temperature-dependent FWHM of the PL spectra for the control and 1.0 M IGM samples (Fig. 5e). We apply a modified PL linewidth model to the data to differentiate scattering mechanisms caused by optical phonons and crystal imperfections (Eq. (1)),

1 $$\varGamma (T)={\varGamma }_{0}+{\varGamma }_{{ac}}+{\varGamma }_{{{{\rm{L}}}}O}={\varGamma }_{0}+{\gamma }_{{{{\rm{a}}}}c}T+{\gamma }_{{{{\rm{LO}}}}}{({e}^{{E}_{{{{\rm{LO}}}}}/{k}_{B}T}-1)}^{-1}$$

where *Γ*(*T*) is the linewidth at temperature *T*, *Γ*_0_ is the temperature-independent linewidth due to inhomogeneities such as disorder and crystalline imperfections, *γ*_ac_ and *γ*_LO_ are the analog broadening term for acoustic and longitudinal optical (LO) phonon, respectively, *E*_LO_ is the energy of the LO phonon, and *k*_*B*_ is the Boltzmann constant. Given that the contributions of acoustic phonons and ionized impurities can be negligible based on the characteristics of the temperature-dependent FWHM plot^44^, the related terms in the PL linewidth equation were omitted. Fitted parameters of the temperature-dependent PL linewidth analysis are demonstrated in Supplementary Table 3. *Γ*_0_ (X_1_) is 10.71 and 6.70 meV for the control and 1.0 M IGM, respectively, confirming higher inhomogeneity in the control sample (Fig. 5e, f). The same is observed in the X_2_ excitonic transition, with *Γ*_0_ of 14.34 and 7.22 meV for control and 1.0 M IGM samples, respectively (Supplementary Fig. 25). The narrower linewidth and higher intensity of the PL signals at 4 ~ 40 K for IGM films indicate less structural disorder and imperfections^44,45^. Additionally, higher crystalline imperfections and lattice distortions result in increased scattering by optical phonons, contributing to the PL linewidth^46^. This is reflected by the significantly larger *γ*_LO_ terms associated with X_1_ transition state, with values of 26.998 meV for control; in contrast, the 1.0 M IGM sample showed considerably lower values of 0.033 meV for X_1_ transition state. *γ*_*ac*_ values for both control (1.150 × 10^−4^ eV K^−1^) and 1.0 M IGM (1.011 × 10^−4^ eV K^−1^) are similar, and much lower than *γ*_*LO*_ values. This means that the contribution of the acoustic phonon to the PL linewidth broadening induced by exciton scattering is negligible, and the LO phonon in the control film is more dominantly influenced by exciton scattering than in IGM films.

This study presents an approach to amplify chiroptical response in chiral 2D MBA_2_PbI_4_ via IGM crystallization. Specifically, a dissymmetry factor *g*_CD_ of 1.2 × 10^−2^ with negligible LDLB contribution was measured for the best IGM sample. This is among the highest intrinsic *g*_CD_ reported, i.e., without any additional chiral additives, which is comparable to their single crystal counterparts as well as inorganic semiconductor clusters (Supplementary Table 4). This study leverages in-situ GIWAXS and UV–Vis transmission to investigate the IGM crystallization, leading to anisotropic crystal orientation. By mediating the reaction between the PbI_2_ and MBAI salt, nanostructured chiral 2D MBA_2_PbI_4_ with enhanced CD response was observed. CD analysis together with PL at 4 K revealed the origin of amplified CD response and spectral bisignate appearance. A more pronounced exciton fine structure feature for IGM samples is a direct consequence of superior crystallinity and crystallite domain homogeneity. This leads to enhanced rotational transitions owing to strong electrodynamic coupling of transition dipoles. In conclusion, we introduce a strategy to amplify chiroptical properties of chiral 2D MBA_2_PbI_4_ halide perovskites by tailoring a synthesis route towards nanostructured crystals. Although the IGM synthesis route might not be applicable to many organic spacers due to solubility constraints, the approach of the nanostructured film formation and manipulation of morphological features is transferable across other chiral 2D materials to boost chiroptical properties. Therefore, we believe that our study demonstrates versatility and applicability in its proof-of-concept, establishing a pathway to amplify chiroptical properties through controlled nanostructure formation.

## Methods

### Materials

*α*-Methylbenzylamine (99%), *R*-(+)-*α*-methylbenzylamine (98%), *S*-(−)-*α*-methylbenzylamine (98%), hydroiodic acid (HI, 57 wt. % in H_2_O, distilled, stabilized, 99.95%), N,N-dimethylformamide (anhydrous, 99.8%), DMSO (anhydrous, ≥ 99.9%), 2-propanol (99.5%, anhydrous), hexane (≥ 99%(GC), ACS reagent, EMSURE®), diethyl ether (≥ 99.0%, anhydrous, ACS reagent, contains BHT as inhibitor), and chlorobenzene (anhydrous, 99.8%) chemicals were purchased from Sigma-Aldrich. Lead(II) iodide (99.99%, trace metals basis)[for Perovskite precursor] chemical was purchased from TCI. All chemicals were used without any further purification and filtration.

### Synthesis of racemic-, *S*-, and *R*-methylbenzylammonium iodide (MBAI) salts

5.0 mL of racemic-, *S*-, or *R*-methylbenzylamine (MBA) solution was diluted with 20 mL of IPA in a round-bottom flask. After cooling down the solution in an ice bath, 4.9 mL of HI was added dropwise to the MBA solution under vigorous stirring in an ice bath. The reaction mixture was stirred for 3 h, after which the solvent was removed by rotary evaporation at 70 °C, yielding a reddish viscous liquid. This resulting viscous liquid was washed 3 ~ 4 times with hexane and diethyl ether under sonication to remove further residual solvents and unreacted HI, obtaining a white precipitate. The resulting white precipitate was filtered and dried under a vacuum oven at 70 °C.

### Preparation of precursor solutions

For the one-step deposition method, the precursor solution was prepared by dissolving S-MBAI (0.8 mmol, 2 eq) and PbI_2_ (0.4 mmol, 1 eq) in 1 mL mixed DMF/DMSO solvent (971 µL DMF and 29 µL DMSO). The fully-dissolved one-step coating solution was filtered through PTFE filters with 0.45 µm pore size before use. For a two-step sequential deposition method, the secondary solution of MBAI (racemic or homochiral organic salt) and PbI_2_ solution were prepared separately. PbI_2_ solution was prepared by dissolving 0.5 mmol PbI_2_ in 1 mL mixed DMF/DMSO solvent (964 µL DMF and 36 µL DMSO). MBAI powder was dissolved in IPA to prepare solutions with various concentrations ranging from 0.1 to 2.0 M. The PbI_2_ and MBAI solutions were filtered using PTFE filters with 0.45 µm pore size before use.

### Fabrication of chiral 2D halide perovskite films

The ITO substrates were subsequently sonicated with detergent solution, acetone, deionized water, and isopropanol for 20 min for cleaning. Before use, the bare cleaned ITO substrates were treated by UV/Ozone for 30 min. Only in case of the sample preparation for PL measurements, we deposited chiral halide perovskite films on glass substrates instead. All of the films were prepared in the N_2_-filled glovebox. For the one-step, 50 µL of 2D MBA_2_PbI_4_ chiral perovskite solution was loaded on the ITO substrate (static coating), and then spin-coated at 2000 rpm for 30 s with 1000 rpm/s acceleration. The as-coated film was transferred immediately onto the hot plate and annealed at 70 °C for 10 min. For the two-step to fabricate chiral halide perovskite films (denoted as IGM films), first the PbI_2_ layer was statically deposited on the ITO substrate by spin-coating at 2000 rpm for 30 s with 1000 rpm/s and then heated shortly at 70 °C for 1 min. Before treating the MBAI solution, the PbI_2_ films were cooled down to room temperature. The 50 µL MBAI solution was dynamically dropped onto the PbI_2_ film while spinning at 2000 rpm. This dropping process began once the spinning speed reached 2000 rpm and lasted for the remaining time (total spinning time is 30 s). The annealing condition is the same as the one-step condition (70 °C for 10 min). To ensure the removal of residual gel phase or MBAI on the resulting IGM films, the annealed films on the hot plate were immediately transferred to the spin coater and subsequently washed with 200 µL of chlorobenzene by slowly dispensing it on the spinning substrates (see Supplementary Note 4 for more details regarding the influence of CB rinsing step on IGM films).

### Characterization

CD and absorbance spectra were measured using a J-815 Jasco spectropolarimeter. The incident beam spot size on the film samples for CD measurements is around 0.6 × 0.7 cm^2^. In this work, all CD, absorbance, and *g*_CD_ spectra are demonstrated with genuine values. To evaluate the genuine *g*_CD_ values, both sides of the film were measured (CD_front_ for the front side and CD_back_ for the back side). The absorbance and CD values were calculated using Abs = (Abs_front_ + Abs_back_)/2 and CD = (CD_front_ + CD_back_)/2 formula, respectively. The LDLB values were calculated using LDLB = (CD_front_−CD_back_)/2. Absorption spectra were performed by the Cary 5000 UV–Vis-NIR spectrophotometer (Agilent Technologies). Scanning electron microscope (SEM) measurements were carried out by Quanta FEG 250 FEI with 10 kV accelerating voltage. For cross-sectional SEM images, 5 nm thickness of platinum was deposited onto the samples using a Pt sputter coater (Quorum, Q150RS Plus) with 20 mA sputter current under rough vacuum of 5 × 10^−2^ mbar. XRD measurements were performed by using Bruker D8-Discover with Co-Kɑ X-ray radiation operating at 35 kV and 40 mA. 2θ values in XRD patterns for Co-Kɑ (*λ* = 0.17890 nm) were converted to Cu–Kɑ (*λ* = 0.17890 nm). Room temperature photoluminescence (PL) measurements were conducted using FLS 980 Edinburgh Instruments with a 375 nm external picosecond pulsed diode laser (EPL–375) as an excitation light source, where the repetition rates are 20 MHz for steady-state PL and 50 kHz for TRPL. The TRPL data were fitted with a bi-exponential equation (*y* = *y*_0_ + *A*_1_exp(−(*x*−*x*_0_)/*t*_1_) + *A*_2_exp(−(*x*−*x*_0_)/*t*_2_)). Through the resulting parameters such as amplitude (*A*_1_ and *A*_2_) and time constant (*t*_1_ and *t*_2_) obtained from the TRPL fitting, average carrier lifetime was calculated from *τ*_average_ = (*A*_1_*t*_1_^2^ + *A*_2_*t*_2_^2^)/(*A*_1_*t*_1_ + *A*_2_*t*_2_). To acquire the in-situ UV–Vis spectra, the time-resolved transmissions were acquired first with deuterium & tungsten-halogen lamps (DH-2000, Ocean Insight) integrated in the system as an excitation white light source, then absorbances were calculated using equation *A* = −log_10_(*T*), where *A* is the calculated absorbance and *T* is the measured transmission. The optical response for in-situ UV–Vis measurements was collected using an optical fiber-coupled Ocean Insight spectrometer (QE-pro) with 100 ms/frame (10 ms of integration time with binning 10 frames). Grazing-incidence wide-angle X-ray scattering (GIWAXS) measurements were carried out at beamline 12.3.2 of the Advanced Light Source (ALS) synchrotron, with a home-built spin-coater housing chamber, enabling the collection of both in-situ and ex-situ data in an N_2_-filled environment. For both in-situ and ex-situ GIWAXS measurements, the X-ray incidence angle was set to 2.0° with 10 keV of beam energy. The sample detector distance was set to 350 mm, and a Pilatus 1 M 2D detector (Dectris Ltd.) detector was positioned at the angle of 35.0° for in-situ GIWAXS and 33.0° for ex-situ GIWAXS from the sample plane. The GIWAXS frames were acquired at a rate of 1 s/frame for in-situ and 30 s/frame for ex-situ GIWAXS data collection. For depth-dependent GIWAXS measurements, the X-ray incidence angles were controlled from 0.1° to 2.0°. Temperature-dependent PL measurements were carried out by using Cryostation S200 (closed-cycle cryostat, Montana Instruments) with a 405 nm external laser diode (Thorlabs LP405-SF30, CW laser). AFM measurements were carried out using an Asylum Jupiter XR atomic force microscope (Oxford Instruments) with tapping mode under ambient conditions, where TAP 300 Al-G probe with a resonant frequency of 300 kHz and a force constant 40 N/m was used.

### In-situ GIWAXS data analysis

The analysis of the synchrotron in-situ GIWAXS data was performed using the in-house developed MultiModalAnalysis (mmanalysis) package, an extension of the pyFAI module^47^ for analyzing X-ray scattering data collected at beamline 12.3.2 of the ALS. The package is implemented in Python and leverages available open-source data science modules: NumPy, SciPy, Pandas, lmfit, and h5py^48–52^. The plots are generated with the help of Matplotlib and Bokeh^53,54^.

The mmaanalysis package is an interactive tool for calibrating, integrating, visualizing, and fitting batches of time-resolved synchrotron GIWAXS data, as well as combining the results with additional optoelectronic in-situ data (e.g., photoluminescence spectra (PL)) and logged synthesis process parameters (e.g., spin-coating or annealing parameters). The calibration of the GIWAXS data is performed in two steps. First, the diffraction geometry at the beamline is calibrated using a two-dimensional GIWAXS image of an Al_2_O_3_ thin film, taken with an area detector as described above. Secondly, the position of every individual sample is recalibrated using the diffraction peaks of the ITO substrate. This calibration is then applied to every image of an in-situ experiment, and the calibrated 2D images are integrated to 1D diffraction patterns. Further, mmanalysis enables the user to correlate the evolution of these 1D diffraction patterns with PL spectra recorded simultaneously. Lastly, mmanalysis allows for peak fitting of both GIWAXS patterns and PL spectra using Pseudovoigt profiles and offers options for combined and interactive visualization of the results. Supplementary Fig. 26 in the Supplementary Information demonstrates the mmanalysis workflow.

### DFT calculation

First-principles calculations were performed within the framework of DFT using the plane-wave pseudopotential method as implemented in the Vienna Ab initio Simulation Package^55^. The generalized gradient approximation (GGA) formulated by Perdew, Burke, and Ernzerhof (PBE) was employed as the exchange–correlation functional^56^. Electron–ion interactions were described by the projector augmented wave (PAW) method^57^. A plane-wave cutoff energy of 520 eV was used to expand the Kohn–Sham orbitals. A Gamma-centered k-point mesh of 2 × 2 × 1 was adopted for electronic Brillouin-zone integration. Equilibrium structural parameters (including lattice parameters and internal coordinates) for each involved bulk material were obtained by total energy minimization using the conjugate-gradient algorithm, with force and energy convergence thresholds of 0.05 eV Å^−1^ and 1 × 10^−5^ eV, respectively. To properly account for the non-negligible long-range van der Waals interactions in hybrid perovskites containing organic molecules, the DFT-D3 van der Waals correction was included. Band structure calculations incorporating spin-orbit coupling were performed to evaluate the Rashba splitting strength. The electronic circular dichroism calculations were performed using the GPAW package with the LCAO mode^58,59^. The PBE functional within the GGA framework was used for the exchange-correlation functional, and the PAW method was used for core-valence interactions. A real-space grid spacing of 0.2 Å was employed. DZP basis sets were used for all atoms (C, H, N, Pb, I)^60^. The SCF density convergence criterion was set to eV. The number of empty bands was automatically determined based on the number of occupied orbitals, with an additional 12 bands to ensure convergence. The LCAO-based RT-TDDFT implementation in GPAW was used for the time propagation^61^. Electron dynamics were initiated by an electric kick perturbation of a.u. under the velocity gauge, using the adiabatic PBE kernel, a time step of 10 as, and a total propagation time of 20 fs.

LLM ChatGPT has been used as an assistant during manuscript writing to improve grammar, suggest alternative phrasings, and to improve the flow of the text. However, the text is the product of the author’s own work and mind.

## Supplementary information

Supplementary information.pdf

Transparent Peer Review file

## Source data

Source Data files

Source Data files (excel files)

## Data Availability

The data (dataset and image files) generated in this study have been deposited in the Zenodo repository and are publicly available in this link, https://doi.org/10.5281/zenodo.21365239 Source data are provided with this paper.

## Code availability

The code used for the analysis of GIWAXS data is available on GitHub https://github.com/sutterfellalab/MultiModalAnalysis and Zenodo^62^.
