# Supplementary material for: Ionic gel-mediated synthesis of nanostructured chiral 2D halide perovskites with amplified chiroptical response
Source: Nat Commun. 2026 Aug 29;17:10316. doi: 10.1038/s41467-026-77274-w (PMC13620143; doi:10.1038/s41467-026-77274-w)
Supplement: Supplementary file 1 [file 41467_2026_77274_MOESM1_ESM.pdf]

Supplementary Information

Ionic gel-mediated synthesis of nanostructured chiral 2D halide perovskites with amplified chiroptical response

Do-Kyoung Lee[1,2], Raphael F. Moral[1,2,3], Finn Babbe[4], Tim Kodalle[2,5], Fabrice Roncoroni[2], Harishankar Jayakumar[2], Kun Zhou[6], Yuanyuan Zhou[6,7,8], Yen Jea Lee[9], Brooks A. Abel[9,10], Craig P. Schwartz[1,11], Carolin M. Sutter-Fella[2]*

[1]Department of Physics & Astronomy, University of Nevada, Las Vegas, Las Vegas, Nevada 89154, USA

[2]Molecular Foundry Division, Lawrence Berkeley National Laboratory, 1 Cyclotron Road, Berkeley, California 94720, USA

[3]Brazilian Synchrotron Light Laboratory (LNLS), Brazilian Center for Research in Energy and Materials (CNPEM), Campinas, Sao Paulo 13083-970, Brazil.

[4]Chemical Sciences Division, Lawrence Berkeley National Laboratory, 1 Cyclotron Road, Berkeley, California 94720, USA

[5]Advanced Light Source, Lawrence Berkeley National Laboratory, 1 Cyclotron Road, Berkeley, California 94720, USA

[6]Department of Chemical and Biological Engineering, The Hong Kong University of Science and Technology, Kowloon, Hong Kong SAR, China

[7]Energy Institute, The Hong Kong University of Science and Technology, Hong Kong, China

[8]Otto Poon Centre for Climate Resilience and Sustainability, The Hong Kong University of Science and Technology, Hong Kong, China

[9]Materials Sciences Division, Lawrence Berkeley National Laboratory, 1 Cyclotron Road, Berkeley, California 94720, USA

[10]Department of Chemistry, University of California, Berkeley, Berkeley, California 94720, USA

[11]Advanced Photon Source, Argonne National Laboratory, Lemont, Illinois 60439, USA.

*Corresponding author email: csutterfella@lbl.gov

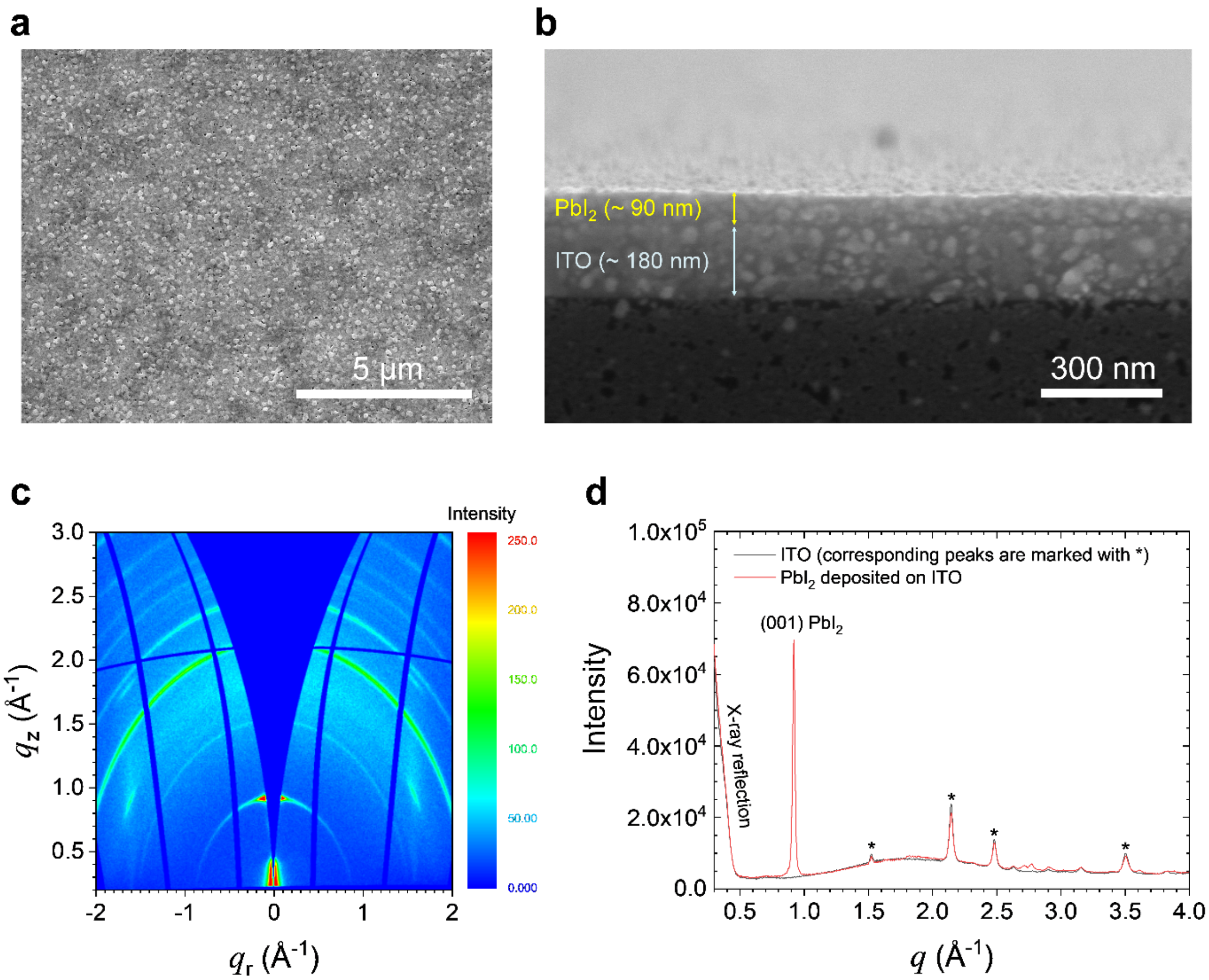

Supplementary Fig. 1 a-b, Top-view and cross-sectional SEM images for a PbI_2 film deposited on the ITO substrate. The PbI_2 film was prepared using 0.5 M coating solution. **c,** 2D GIWAXS frame for the PbI_2 film. **d,** Diffraction patterns obtained from the GIWAXS image illustrated in this Figure, panel **c**.

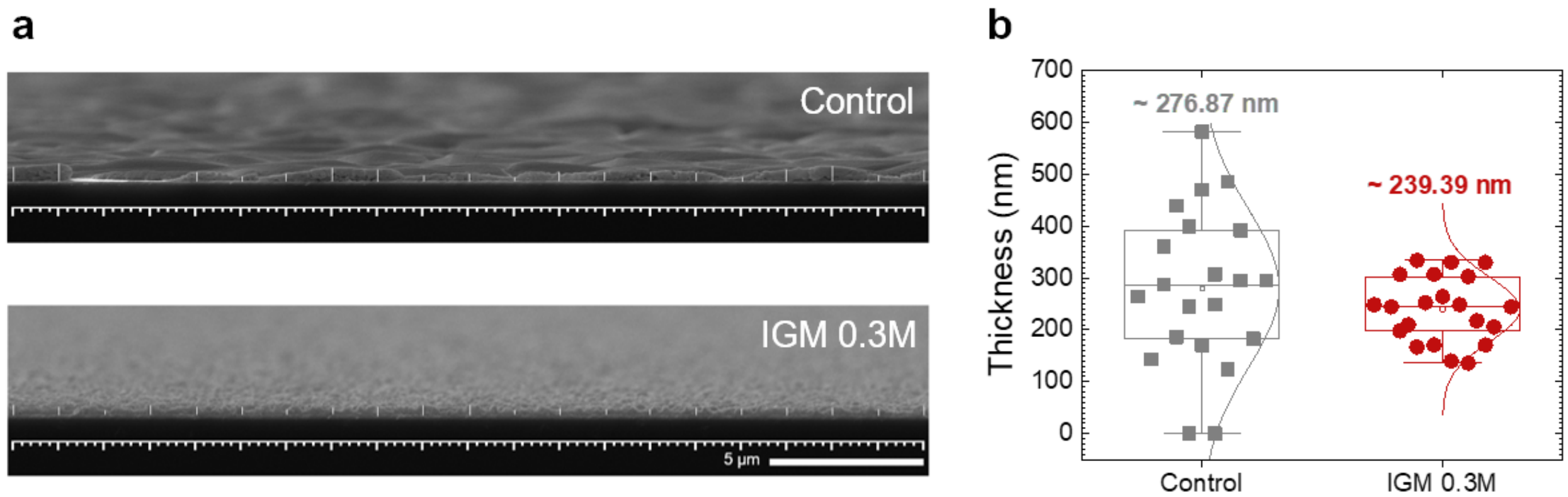

Supplementary Fig. 2 a, Cross-sectional SEM images for control and 0.3M IGM films. **b,** Statistics of the distribution of film thicknesses from cross-sectional SEM images by estimating 21 spots.

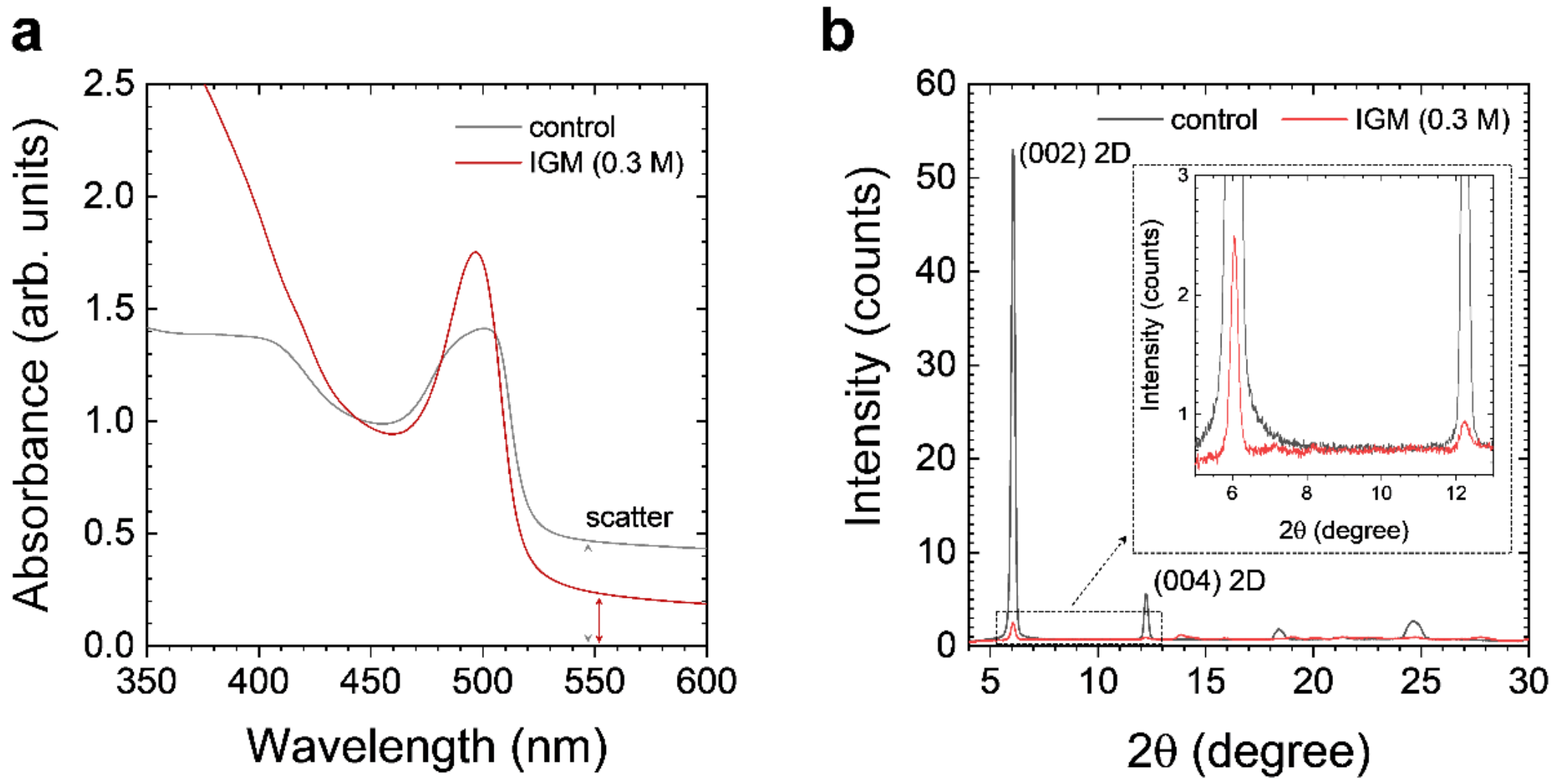

Supplementary Fig. 3 a, absorbance spectra and **b,** X-ray diffraction (XRD) patterns for control and IGM *S*-MBA_2PbI_4 films. The inset zooms in on the (002) and (004) peak region.

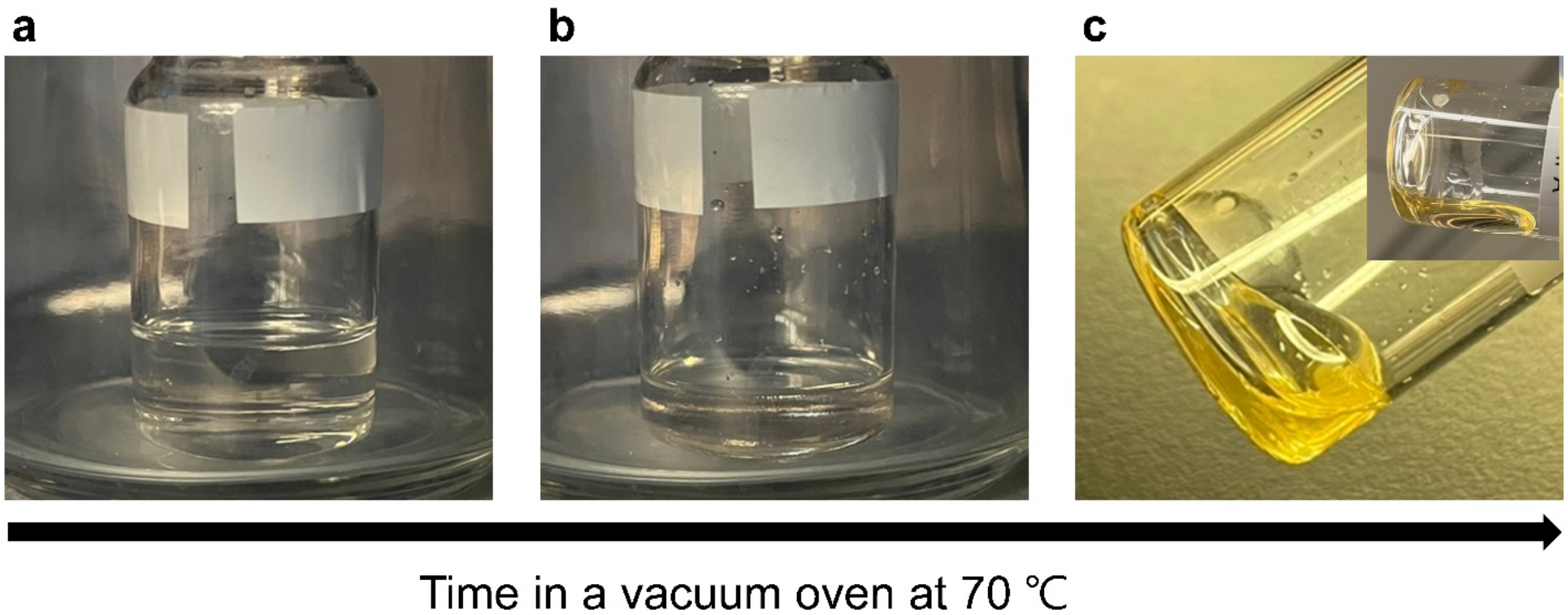

Supplementary Fig. 4 a-c, Photographs of 1.0 M *S*-MBAI solution in IPA over time upon drying in a vacuum oven at 70 °C: fresh, after 1 h, and after 24 h. After 24 h a yellowish gel-like liquid without any precipitates is observed.

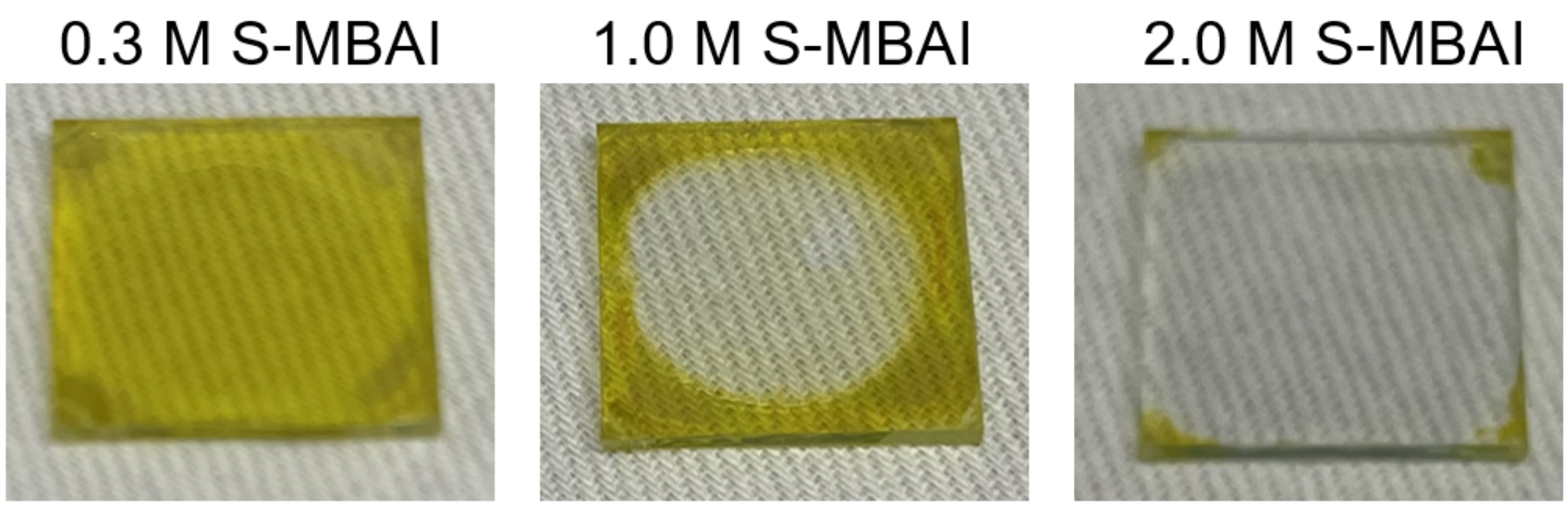

Supplementary Fig. 5 Photographs of PbI_2 films in which different concentrations (0.3 M, 1.0 M, and 2.0 M) of *S*-MBAI solutions were dropped on the PbI_2 film surface.

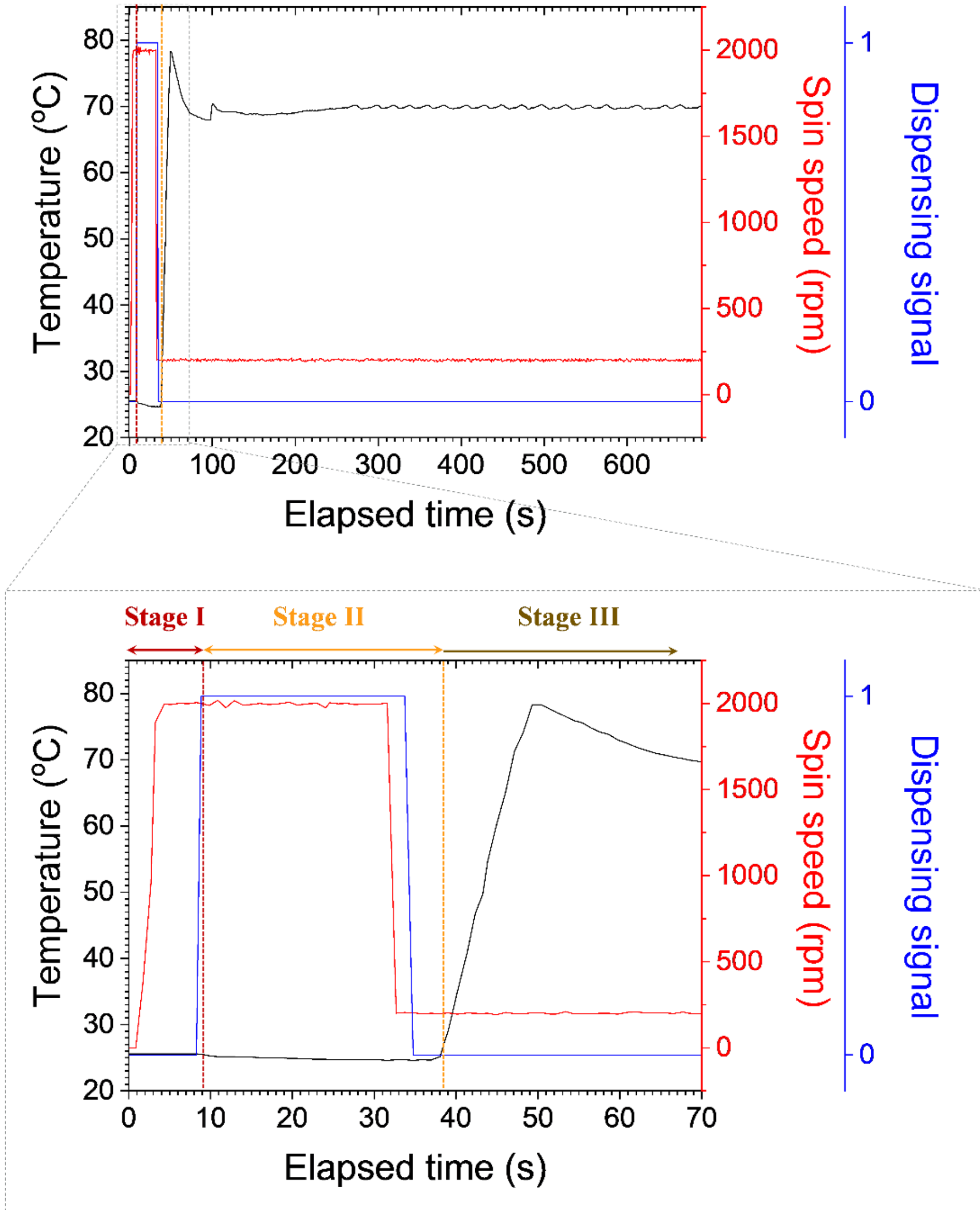

Supplementary Fig. 6 Details of *in-situ* GIWAXS measurement. Time evolution of temperature (black), spin speed (red), and dispensing signal (blue). For the latter, "1" indicates that the plunger of the micropipette was pressed down to dispense the *S*-MBAI in IPA solution onto the substrate, and "0" indicates that the plunger was released back to the initial position. The overall process is divided into three stages, as shown in the expanded view: stage I involves solution dispensing and spin acceleration at room temperature (~25 ℃); stage II corresponds to film formation at room temperature and constant spin speed at 2000 rpm; stage III represents the post-spin annealing step, where the temperature is increased to 70 ℃ after the spin speed is reduced up to 200 rpm.

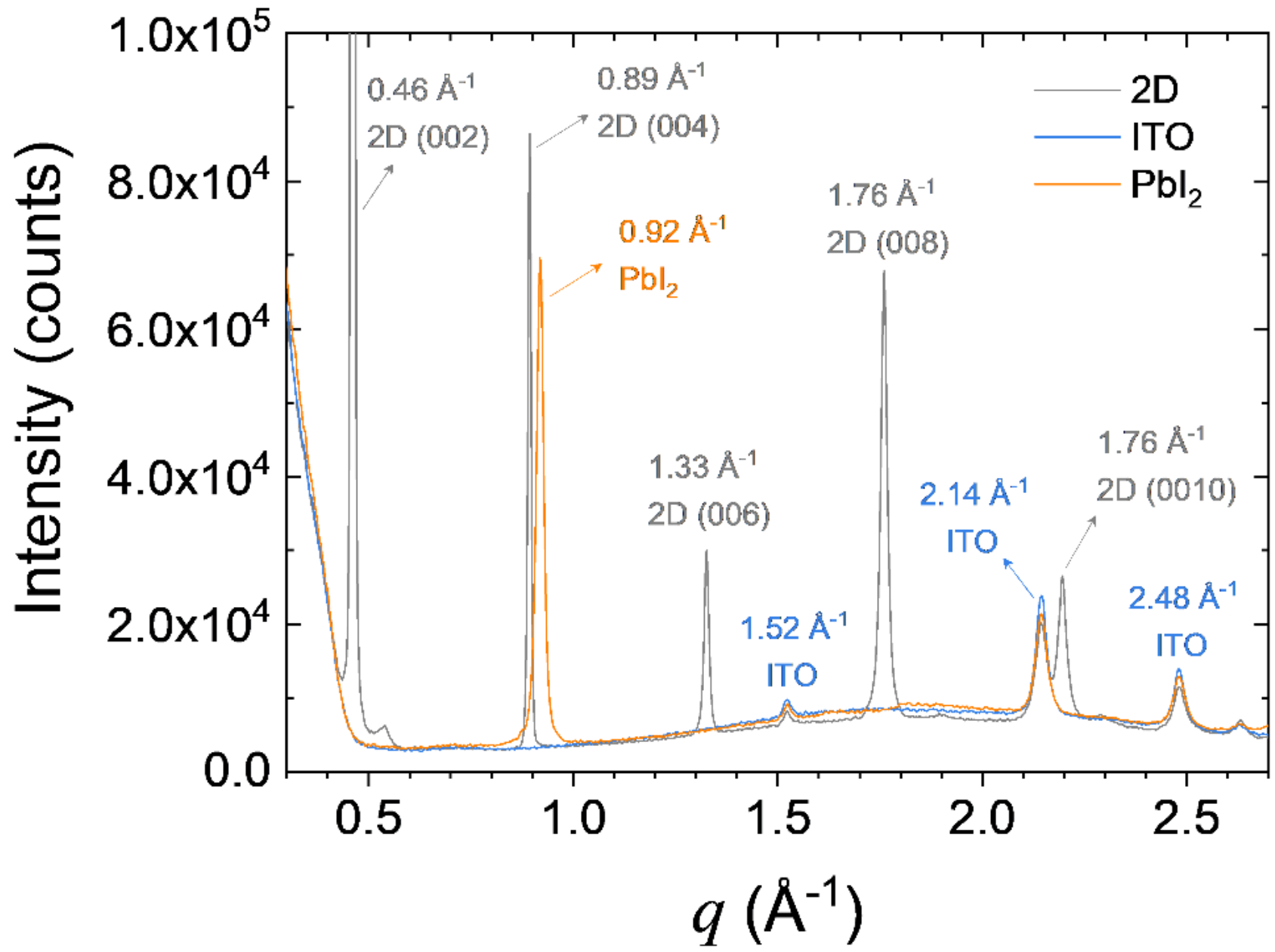

Supplementary Fig. 7 Diffraction patterns for 2D *S*-MBA_2PbI_4, bare ITO substrate, and PbI_2 films. These diffraction patterns were obtained from synchrotron-based GIWAXS measurements.

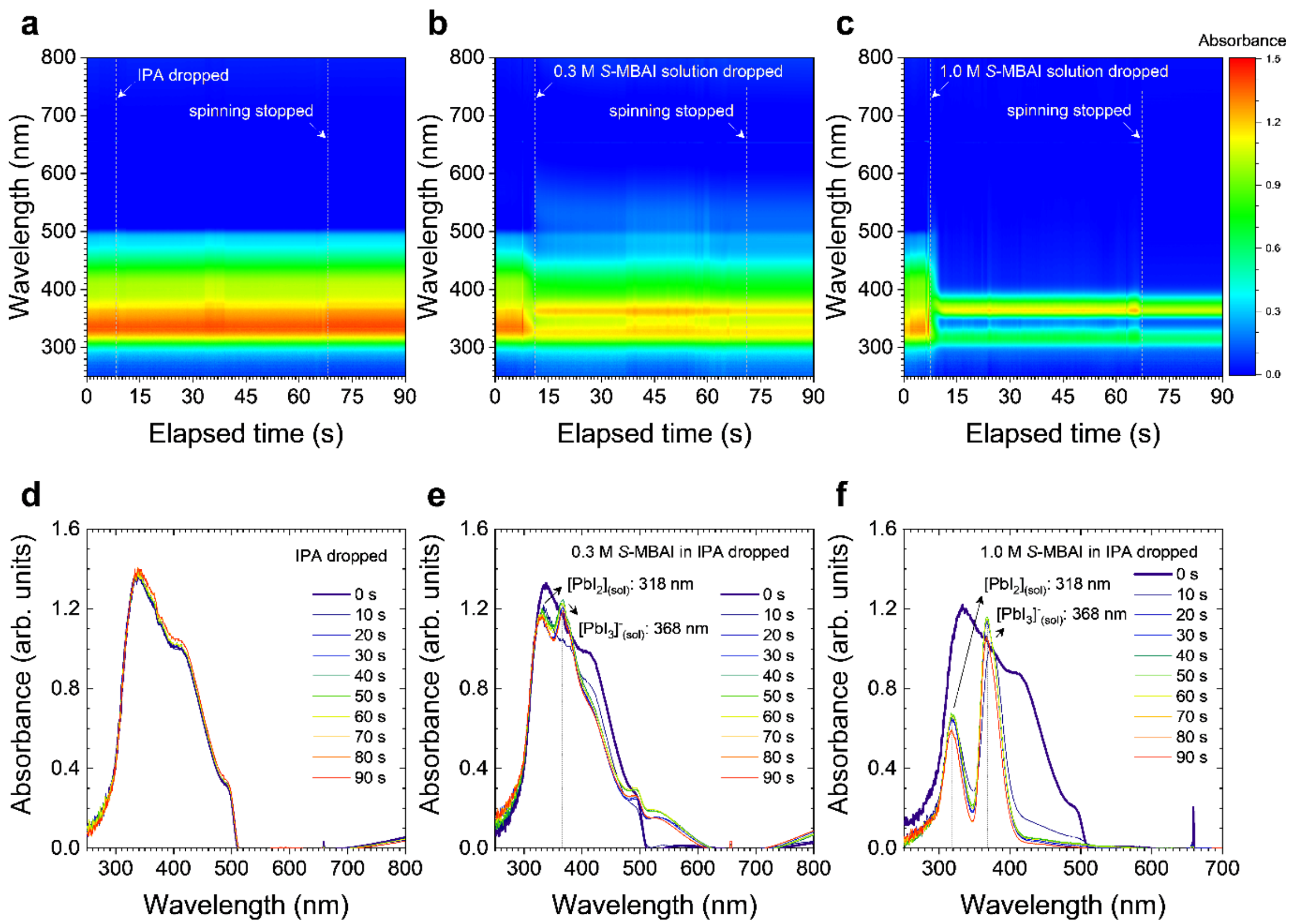

Supplementary Fig. 8 a-c, Contour plots and **d-f,** individual spectra of *in-situ* absorbance data collected during the spin coating process for IGM samples. The PbI_2 films were surface-treated by using pure IPA (**a,d**), 0.3 M (**b,e**), and 1.0 M *S*-MBAI solutions (**c,f**). The solutions were dynamically dropped on the PbI_2 film at 5 s after the beginning of the spinning process.

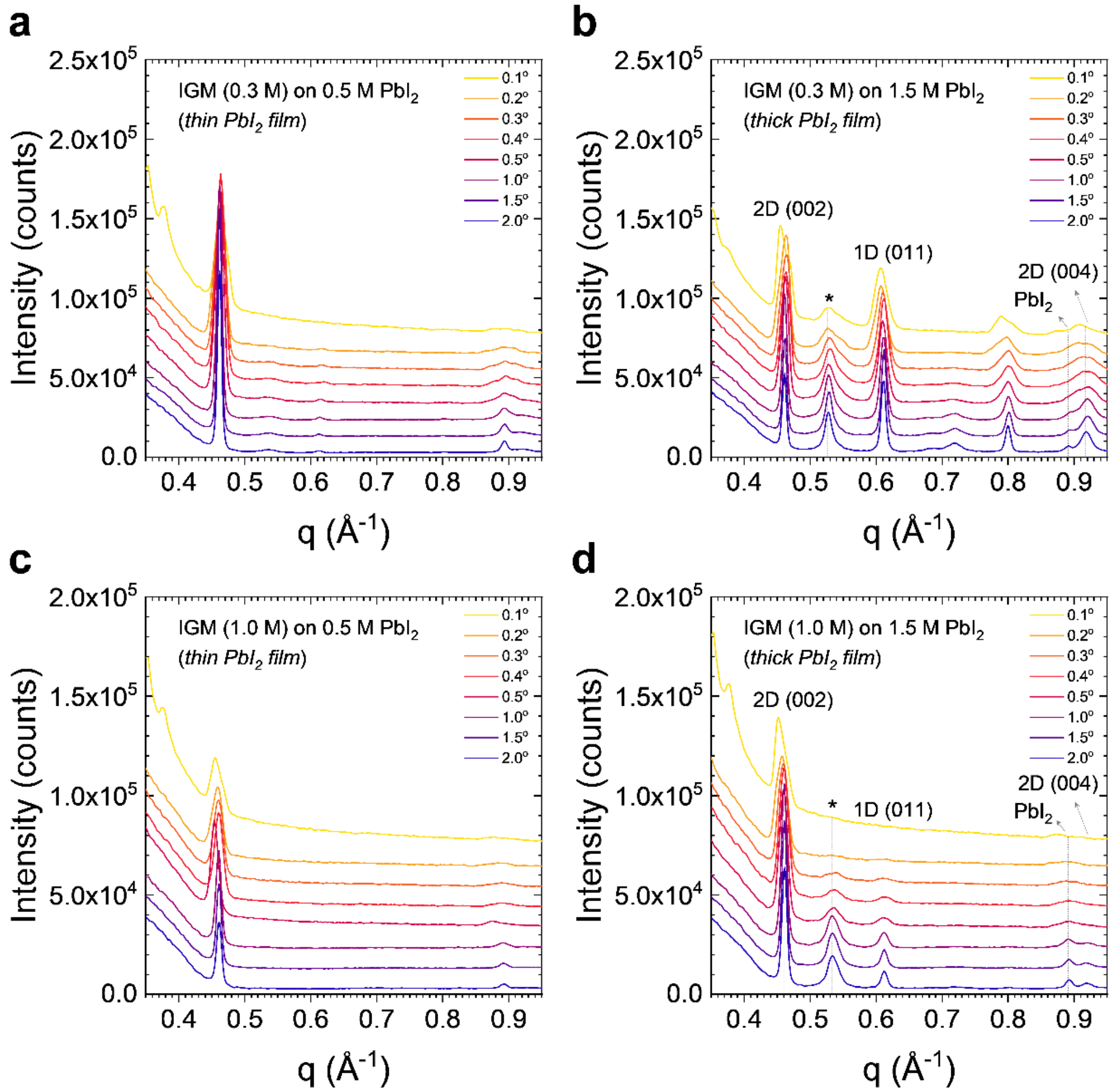

Supplementary Fig. 9 Depth-dependent GIWAXS at X-ray grazing incidence angles from 0.1° (surface) to 2.0° (bulk) for IGM films, where thin (**a,c**) *i.e.* 0.5 M and thick (**b,d**) *i.e.* 1.5 M PbI_2 films have been treated with (**a,b**) 0.3 M and (**c,d**) 1.0 M *S*-MBAI solutions. The PbI_2 thickness is approximately 90 nm for the 0.5 M PbI_2 solution and 550 nm for the 1.5 M PbI_2 solution. The origin of the crystalline phase with diffraction peak at 0.53 Å^{-1} (marked as *) remains unclear, but is likely attributable to the formation of an MBA-intercalated phase induced by the MBAI deficiency.

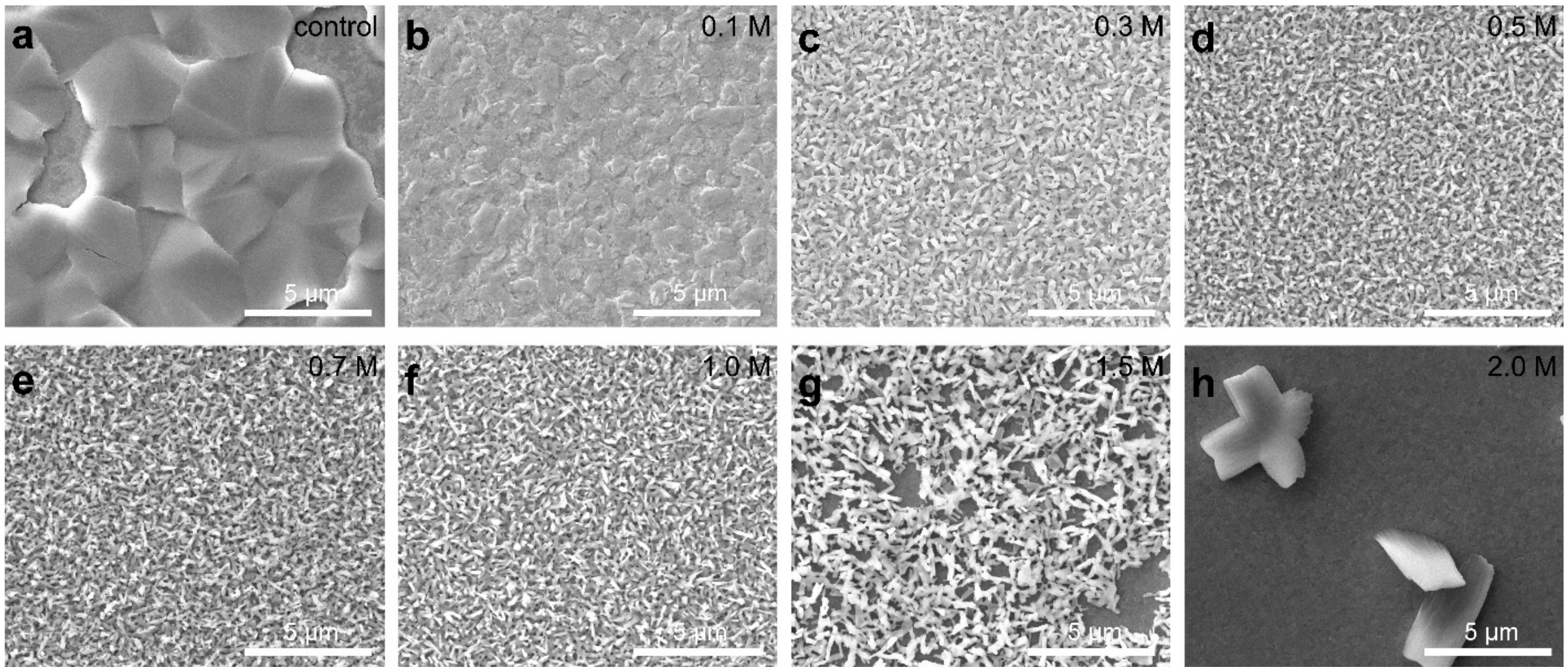

Supplementary Fig. 10 a-h, top-view SEM images for (**a**) control and (**b-h**) IGM films with various concentrations of *S*-MBAI solutions. The scale bar represents 5 μm.

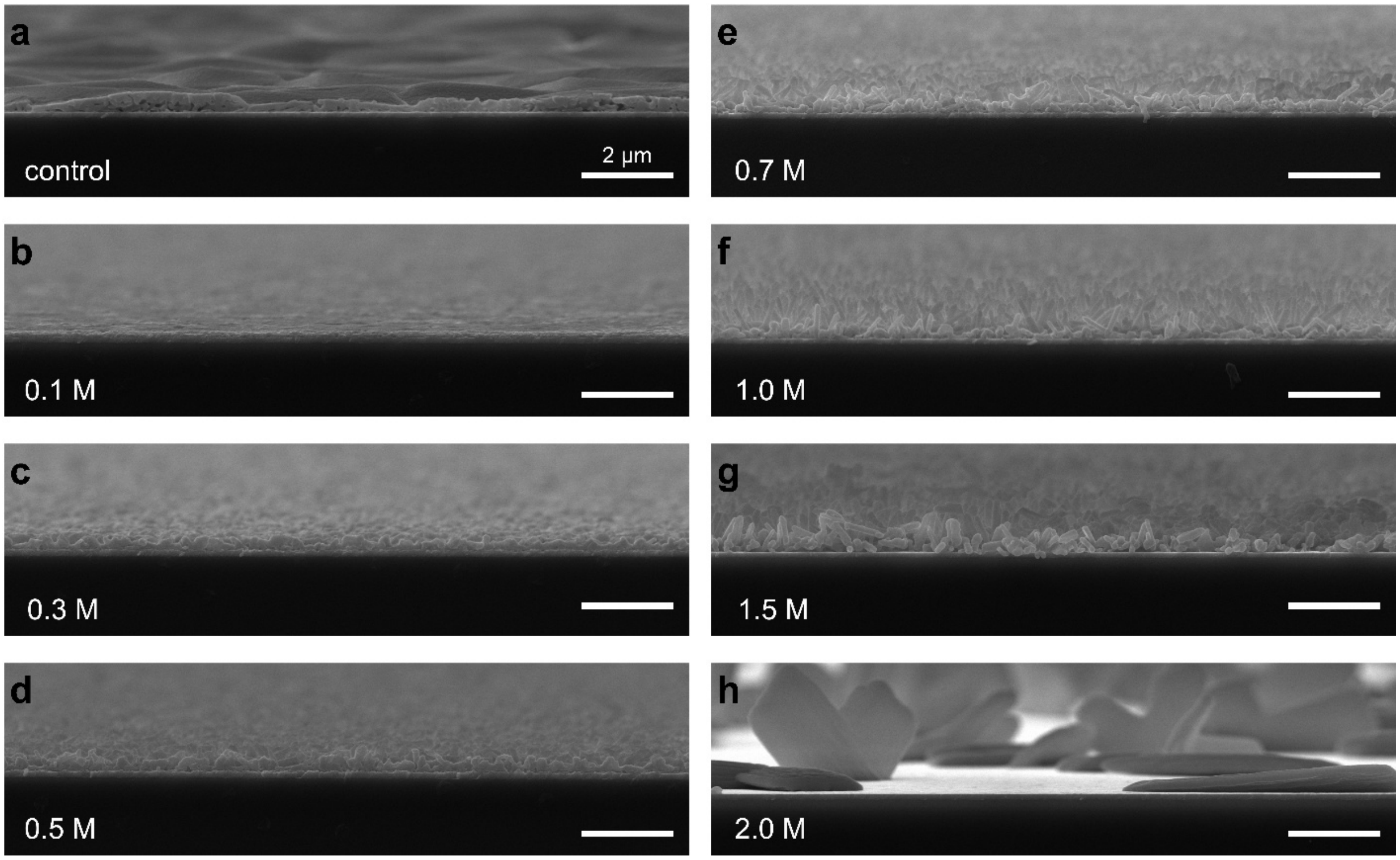

Supplementary Fig. 11 a-h, cross-sectional SEM images for (**a**) control and (**b-h**) IGM films with various concentrations of *S*-MBAI solutions. The scale bar represents 2 μm.

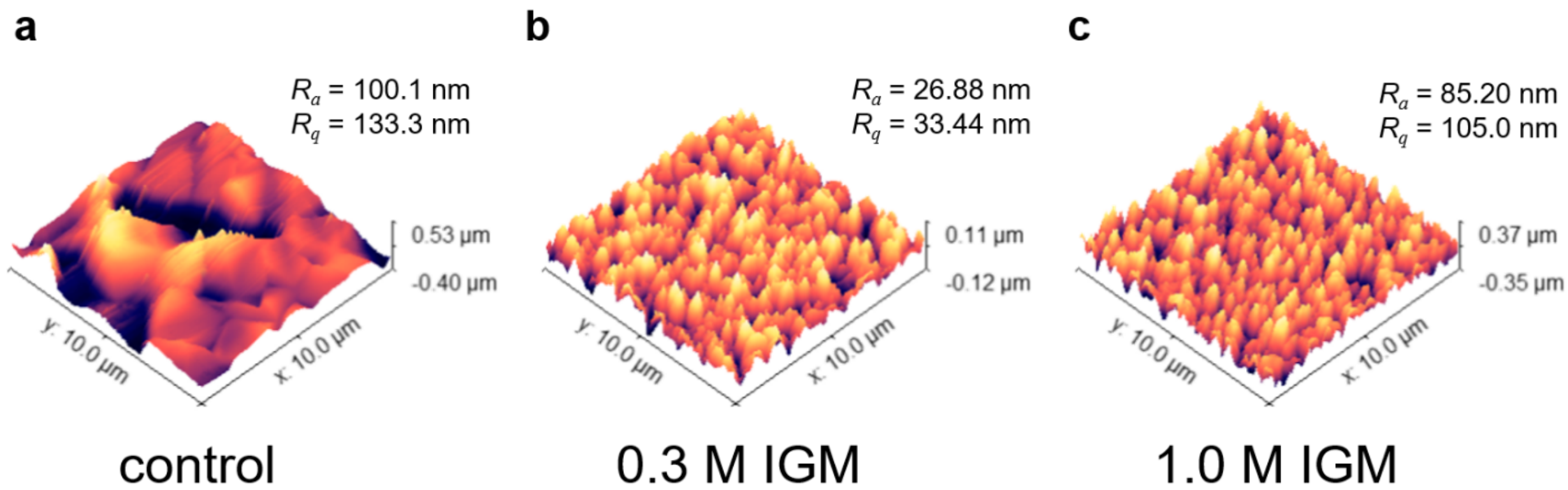

Supplementary Fig. 12 a-c, 3D view of AFM images for (**a**) control and IGM films ((**b**) 0.3 M and (**c**) 1.0 M), where R_a is average surface roughness and R_q is Root-Mean-Square surface roughness.

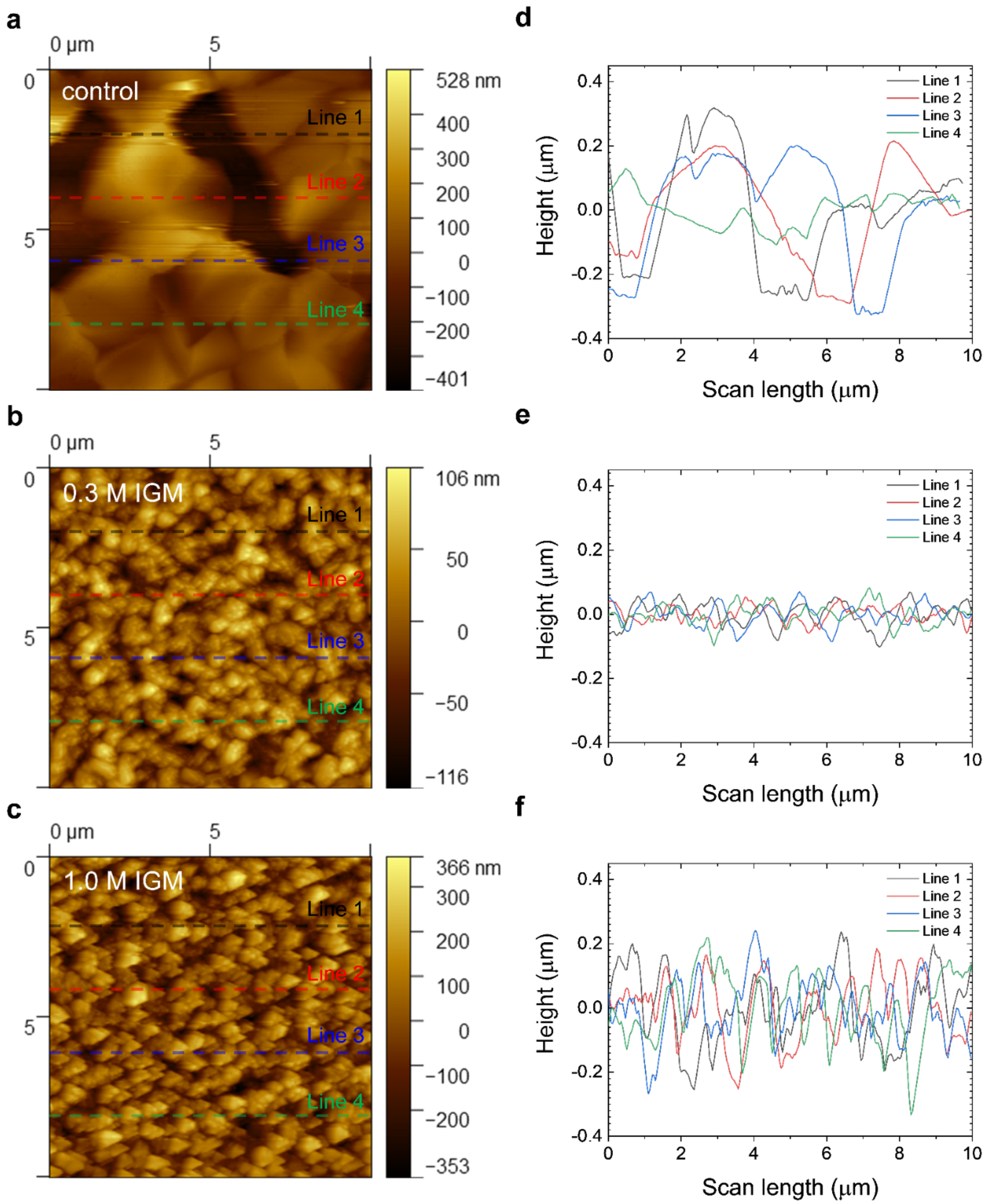

Supplementary Fig. 13 (**a-c**) 2D AFM images and (**d-f**) line profiles with 4 lines.

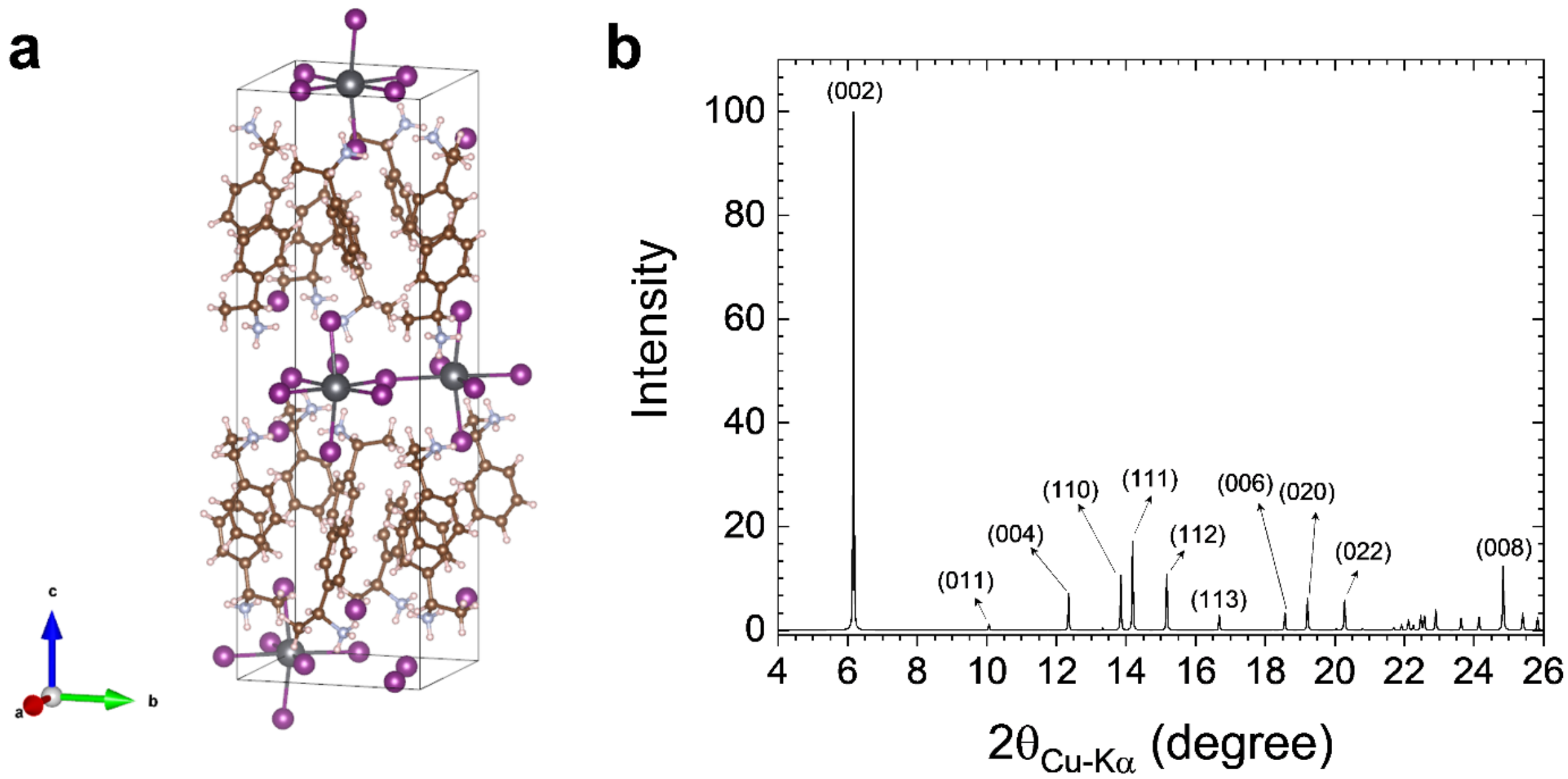

Supplementary Fig. 14 a, Crystal structure of *S*-MBA_2PbI_4 chiral halide perovskite Ruddlesden-Popper (RP) phase. **b**, Calculated diffraction pattern corresponding to the *S*-MBA_2PbI_4 chiral halide perovskite phase.

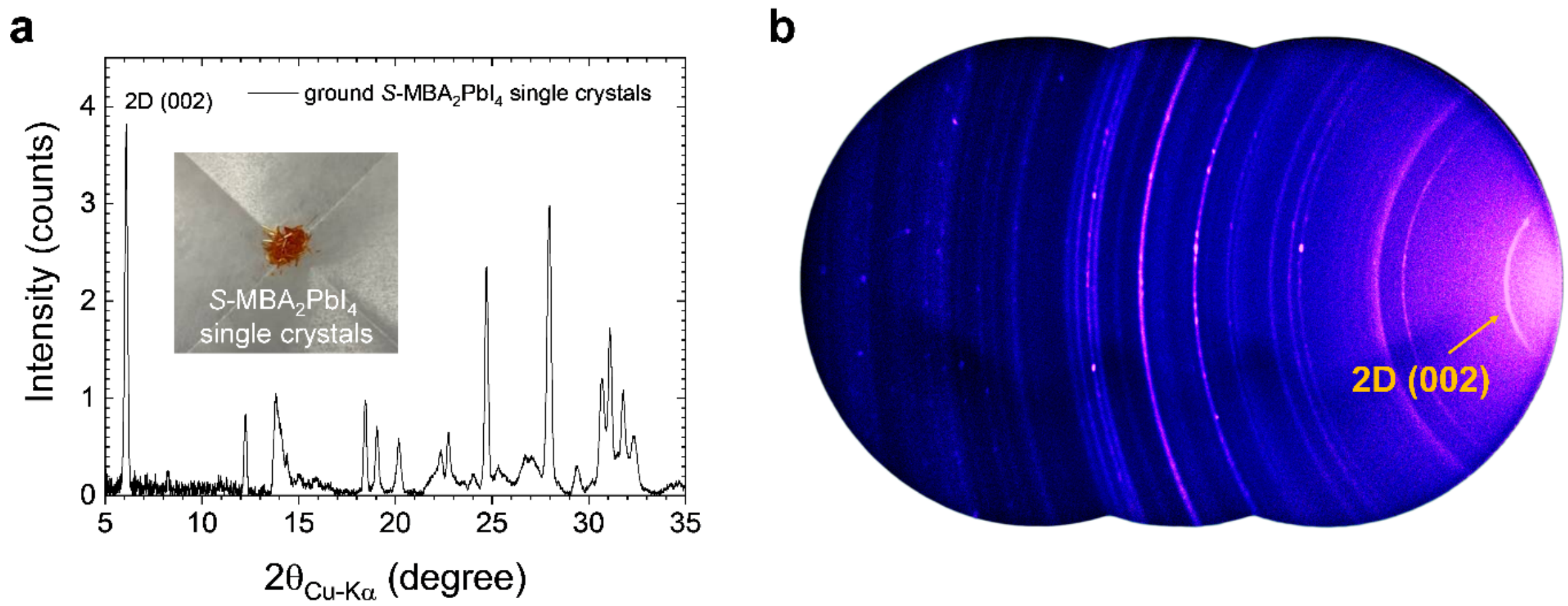

Supplementary Fig. 15 a,b, Background subtracted powder diffraction pattern (**a**) and 2D detector frames (**b**) of ground *S*-MBA_2PbI_4 single crystals.

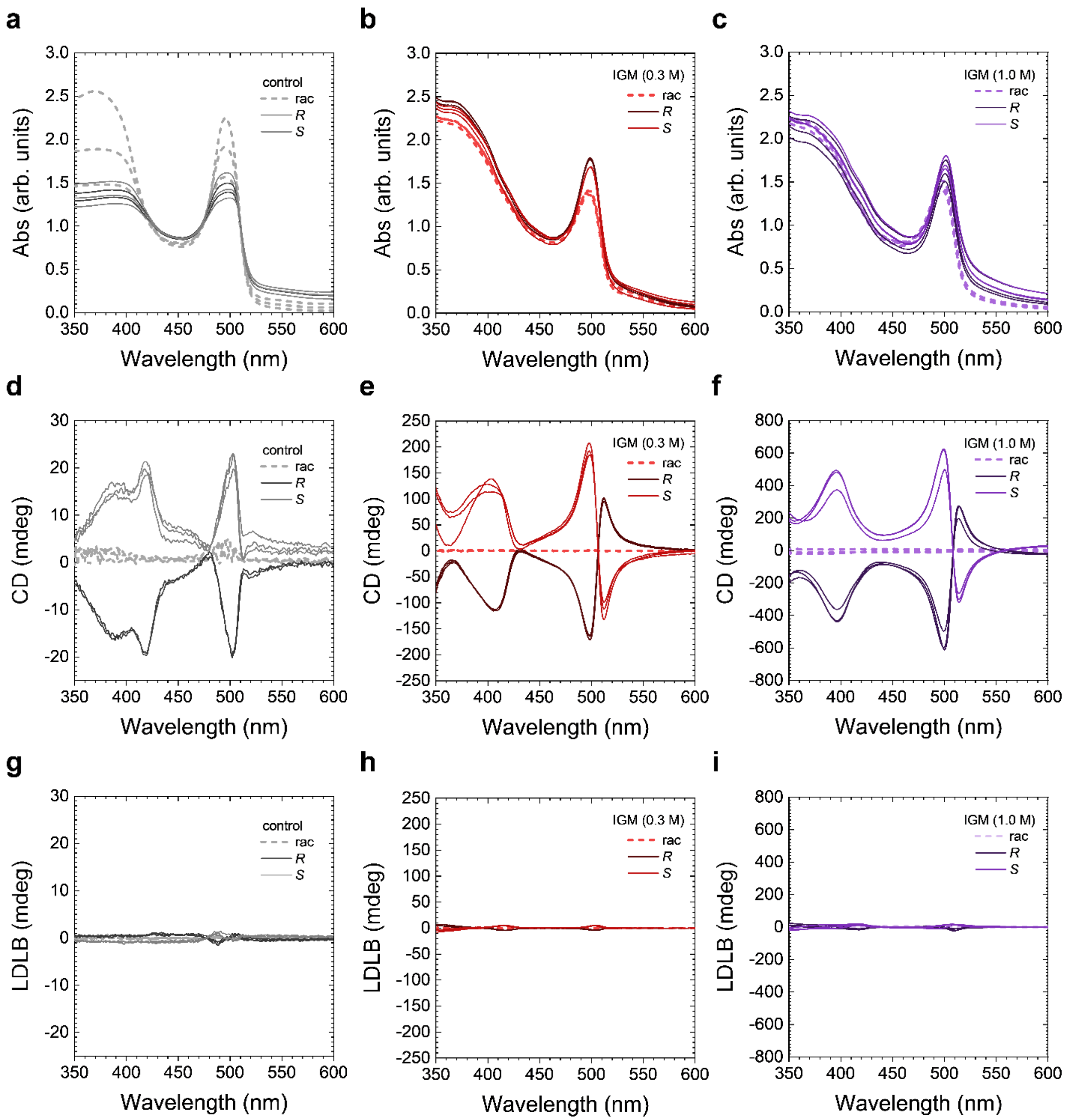

Supplementary Fig. 16 a-c, Absorbance spectra. For the continuum region (<500 nm), the absorbance is gradually enhanced from 0.1 M to 0.5 M, likely due to increasing substrate coverage, while the control film shows comparatively low absorbance due to incomplete substrate coverage. With concentrations > 0.5 M, absorbance drops due to nanorod formation until micrometer-sized crystals form at concentrations > 1.5 M, also leading to poor substrate coverage, thus decreasing absorbance. **e-f,** CD spectra, and **g-i,** LDLB spectra for control (**a,d,g**) and IGM films (0.3 M: **b,e,h** & 1.0 M: **c,f,i**). CD signals were measured from the front- and back-side of the films to calculate the LDLB signals: $(CD_{front} - CD_{back})/2$.

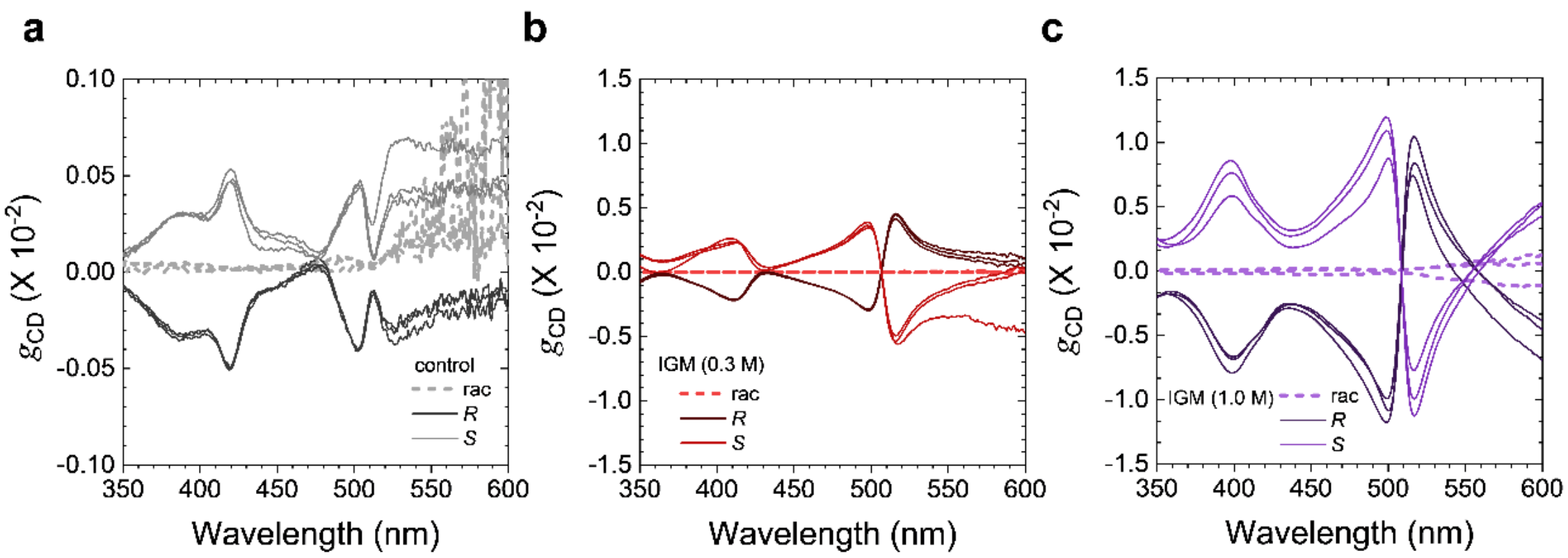

Supplementary Fig. 17 a-c, Circular dichroism dissymmetry factor (g_{CD}) spectra for control and IGM (0.3 M & 1.0 M) films.

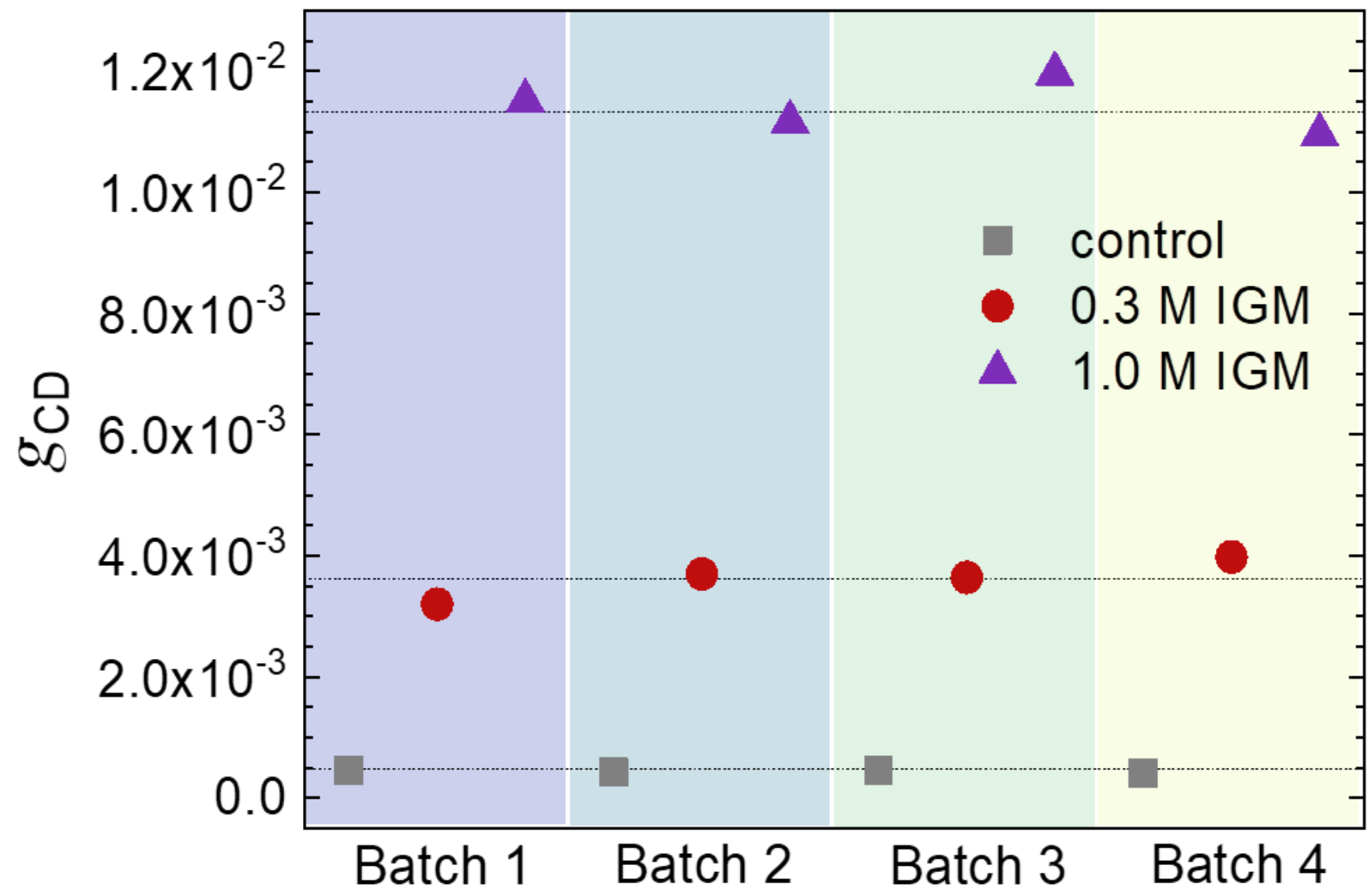

Supplementary Fig. 18 g_{CD} to demonstrate reproducibility in independent batches for control and IGM (0.3 M and 1.0 M) conditions. g_{CD} was obtained from CD spectra at ~ 500 nm corresponding to the excitonic absorption of *S*-MBA_2PbI_4.

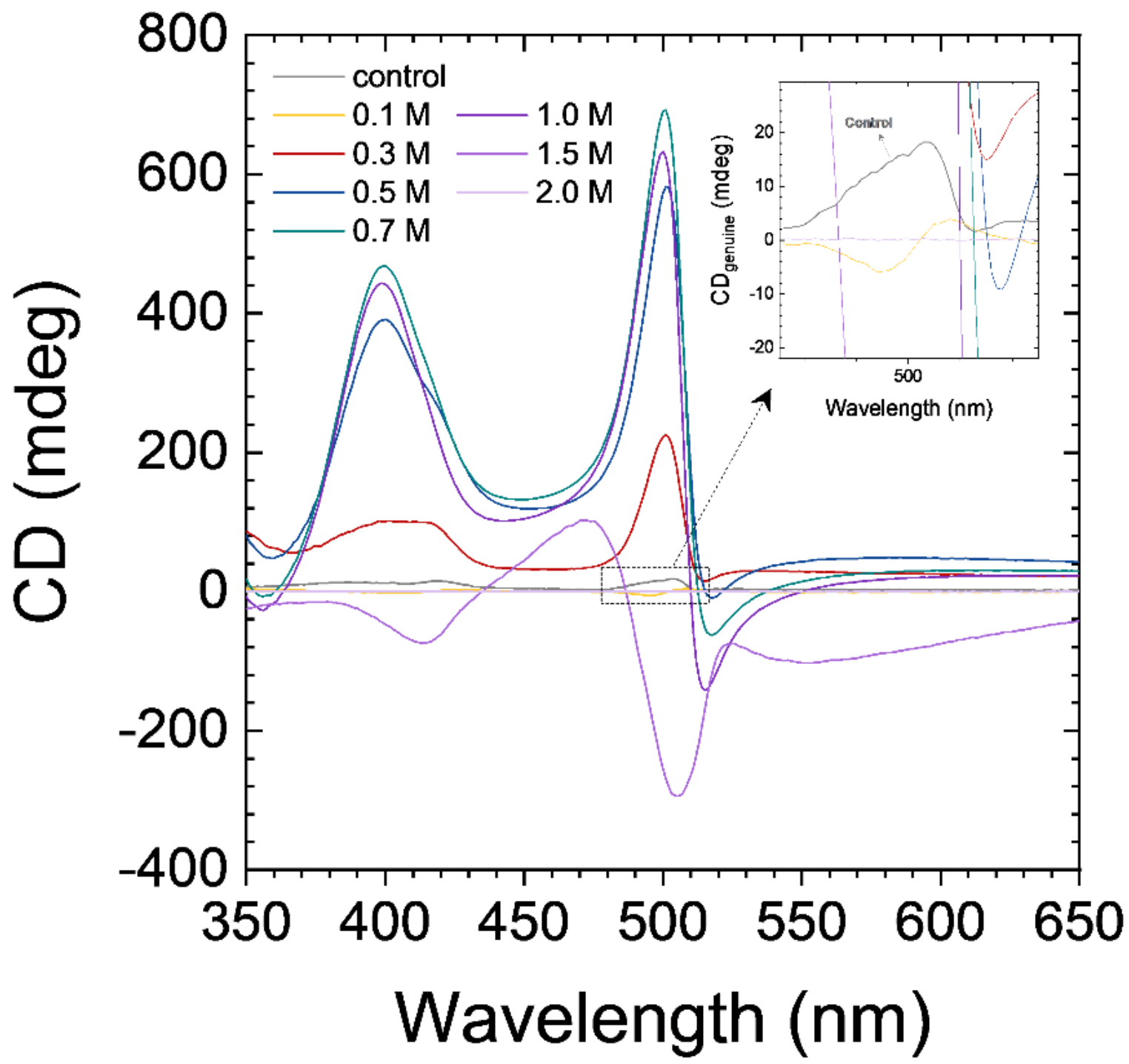

Supplementary Fig. 19 CD spectra for control and IGM (0.1 M ~ 2.0 M). The films measured both sides (back-side and front-side) to obtain these genuine CD responses.

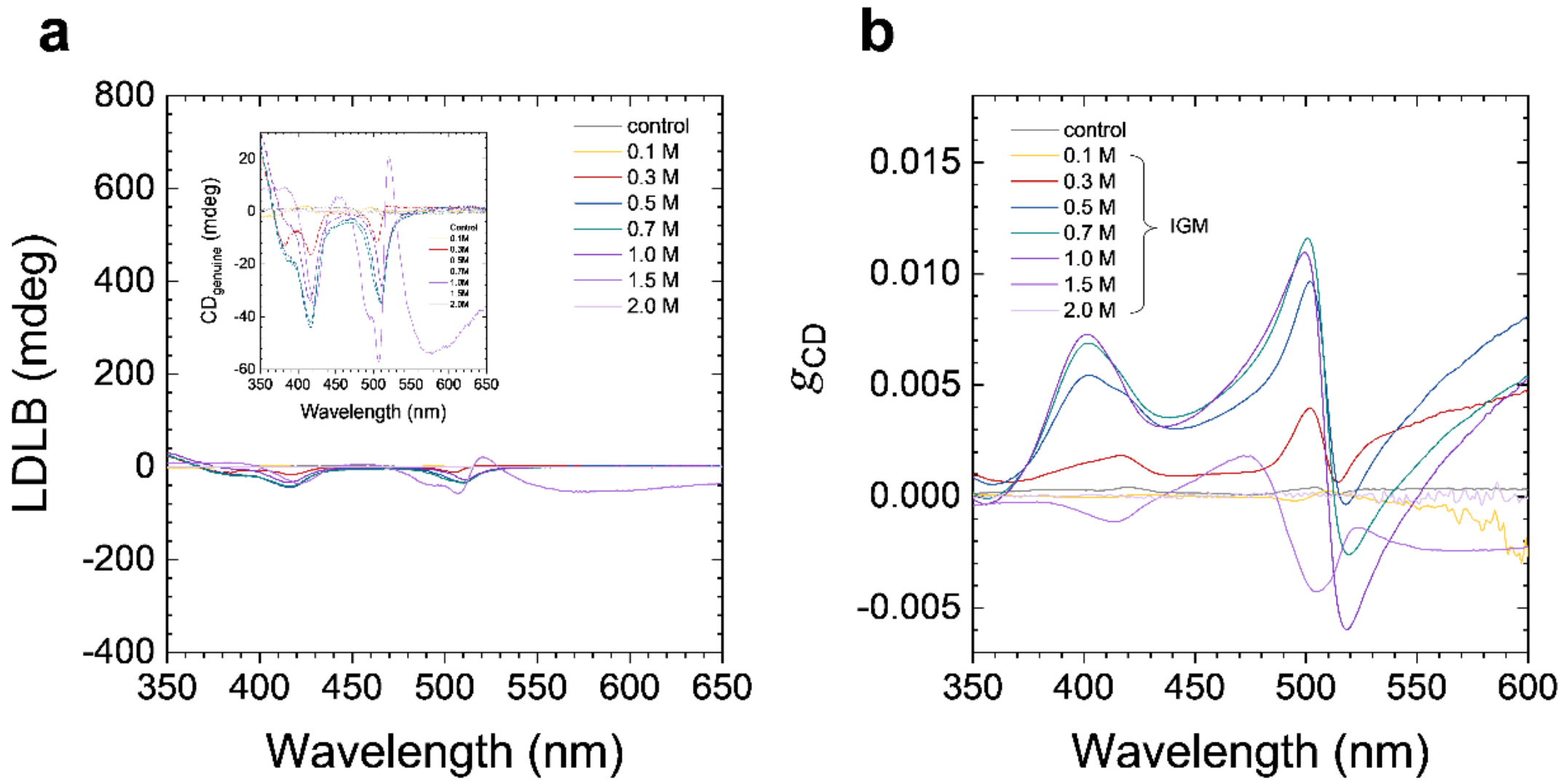

Supplementary Fig. 20 LDLB (**a**) and g_{CD} spectra (**b**) for control and IGM (0.1 M ~ 2.0 M) films.

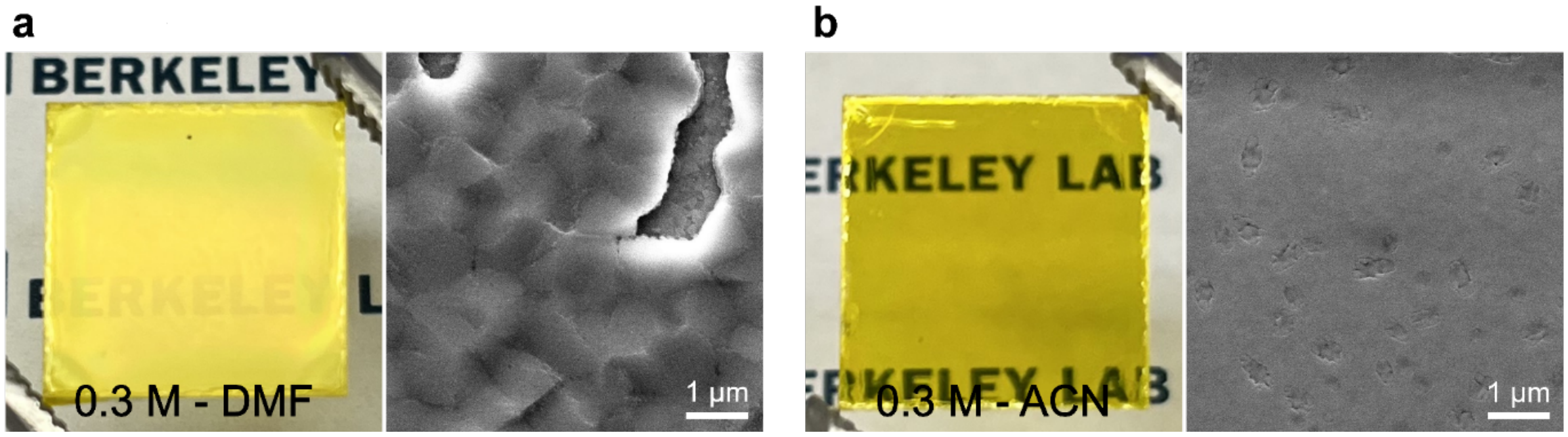

Supplementary Fig. 21 a,b, (left) photographs and top-view SEM images for *S*-MBA_2PbI_4 perovskite films using (**a**) DMF and (**b**) ACN solvents via a one-step deposition method. The transparency indicates the film coverage and surface roughness.

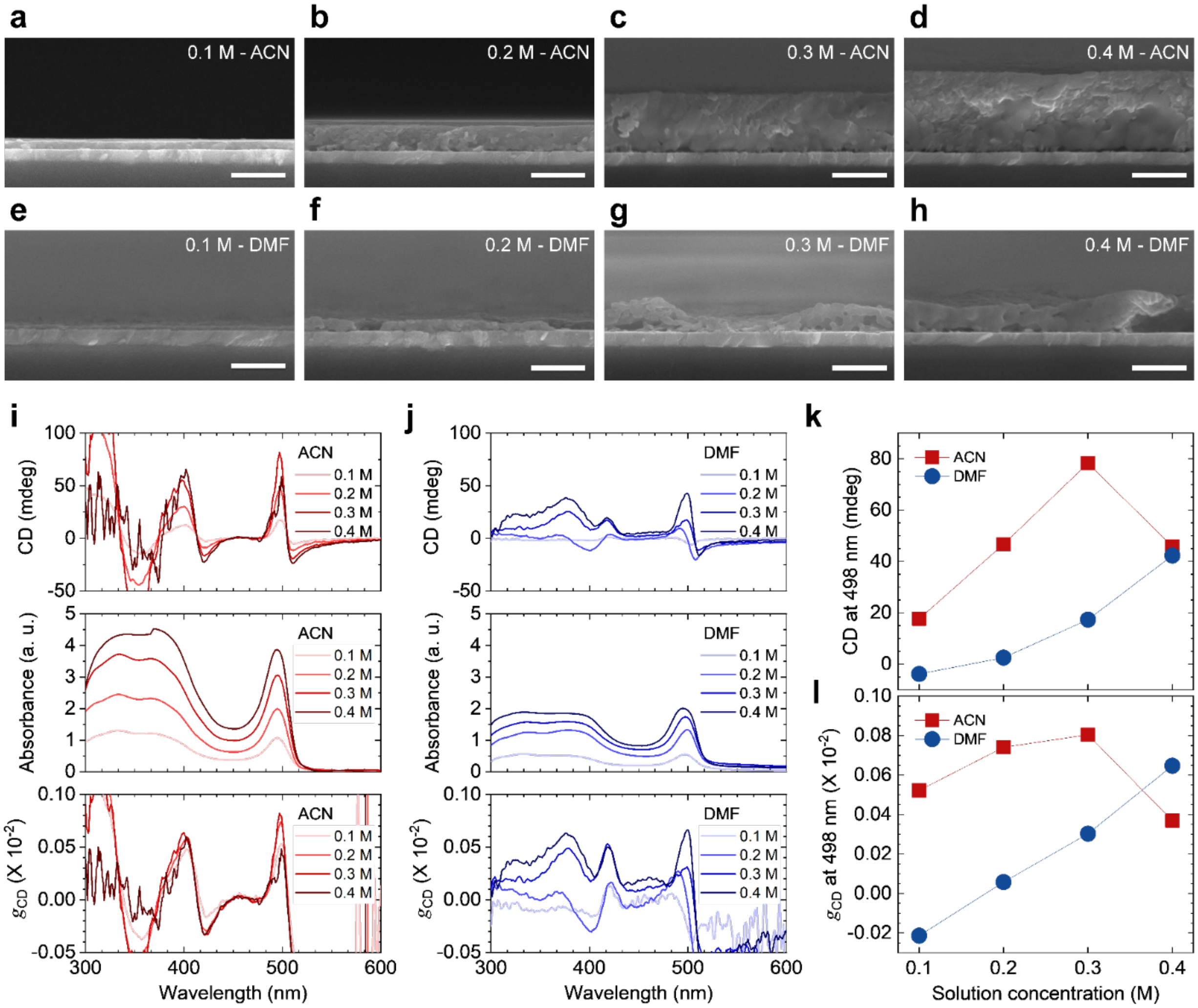

Supplementary Fig. 22 Morphological and chiroptical characteristics of *S*-MBA_2PbI_4 halide perovskite films with different concentrations (0.1 ~ 0.4 M) and solvent system (ACN, DMF) of precursor coating solution. **a-h**, Cross-sectional SEM images for (**a-d**) ACN-based and (**e-h**) DMF-based films. The scale bar is 500 nm. **i-j**, CD, absorbance, and g_{CD} spectra for the corresponding films. **k,l**, CD and g_{CD} values at 498 nm as a function of the precursor solution concentration.

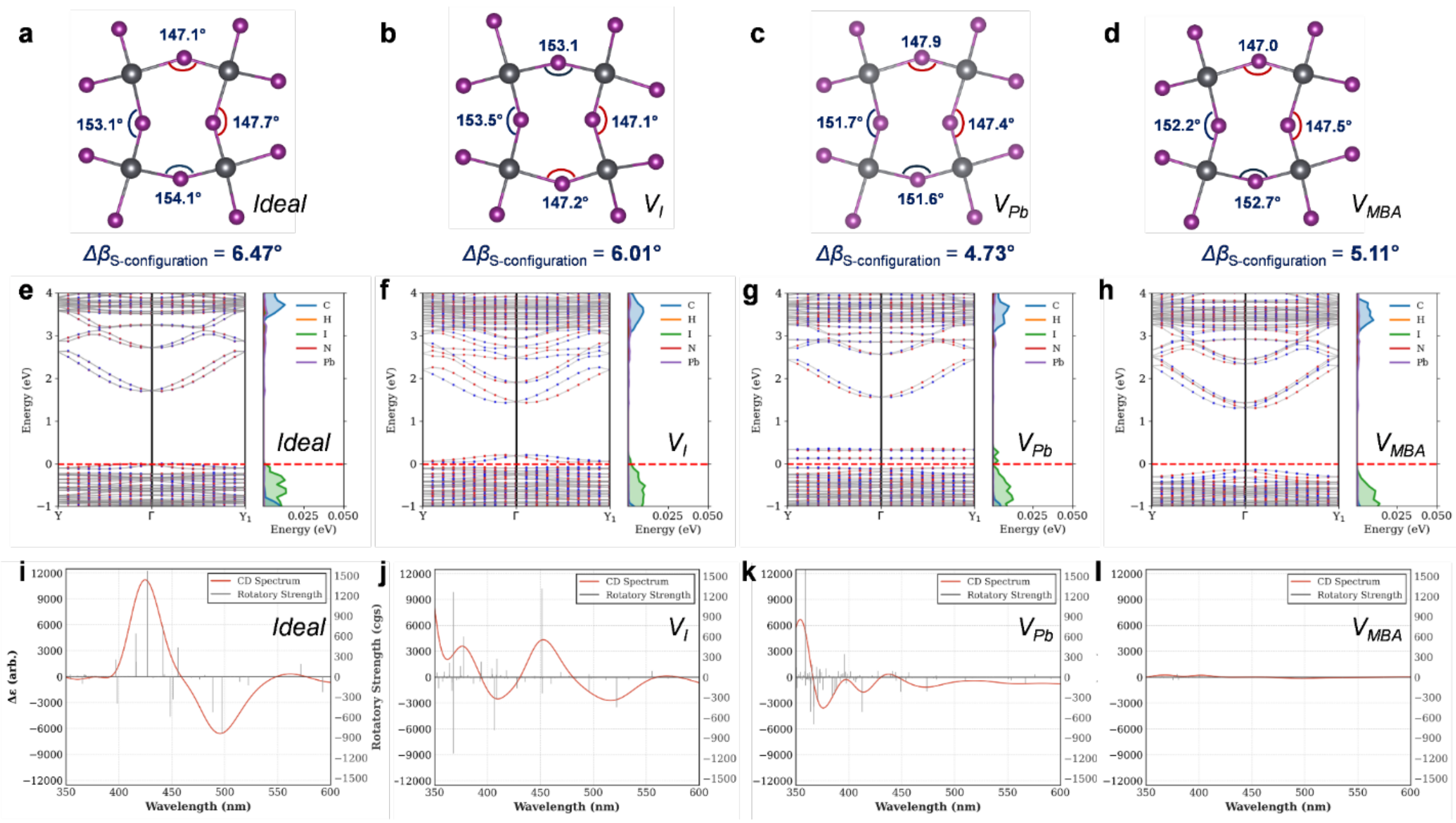

Supplementary Fig. 23 Calculated band structures (top row, a–d), Scheme of tilted octahedral frameworks (middle row, e–h), and CD spectra (bottom row, i–l) for ideal structure and structures with defects: V_I (iodine vacancy), V_{Pb} (lead vacancy), and V_{MBA} (*S*-MBA vacancy). $\Delta\boldsymbol{\beta}$ represents the tilt angle discrepancy between an inequivalent pair of neighboring octahedra.

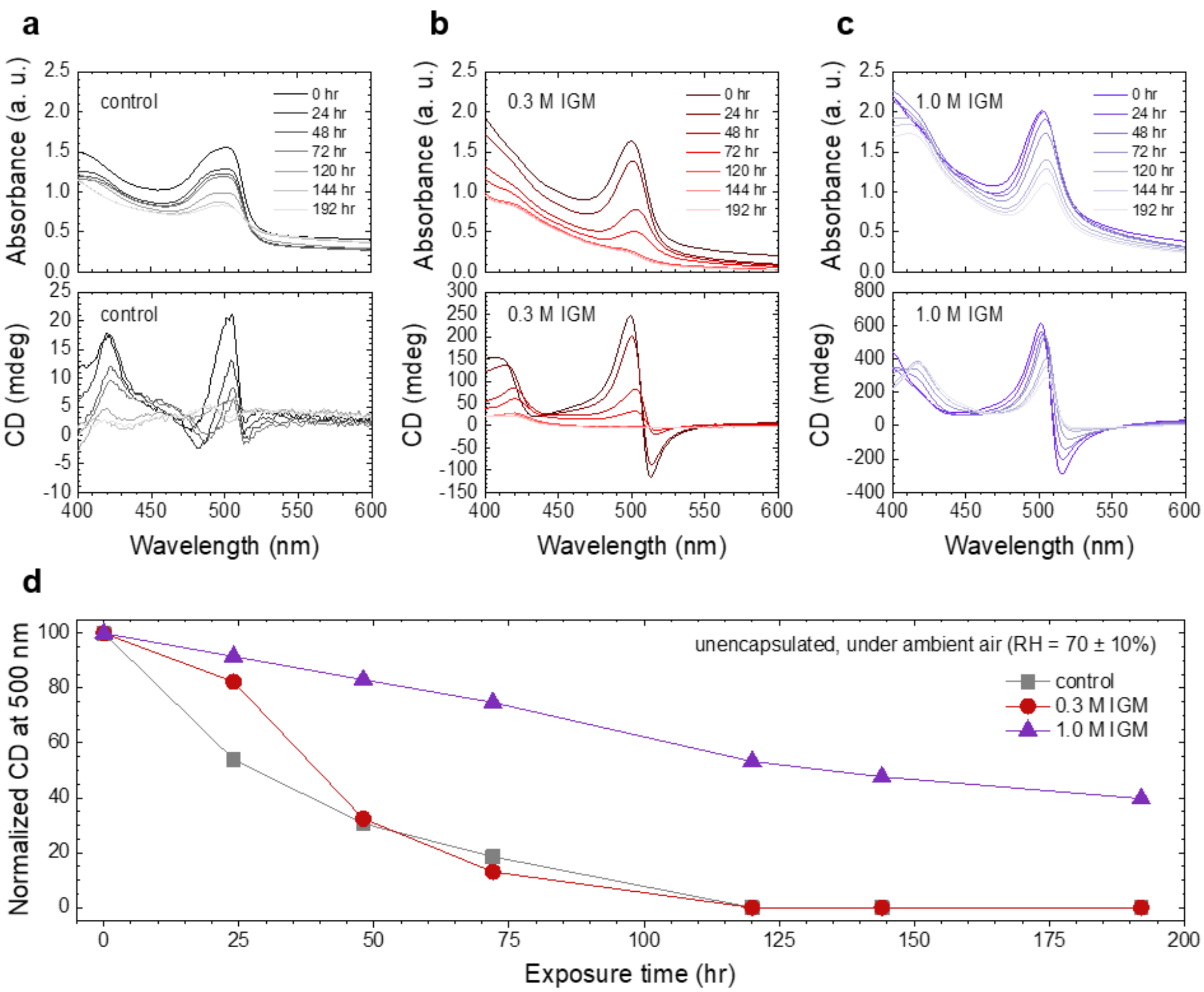

Supplementary Fig. 24 Moisture stability of chiral halide perovskite films for control and IGM (0.3 M and 1.0 M) with (**a-c**) absorption (top), CD (bottom), and (**d**) normalized CD at 500 nm over exposure time.

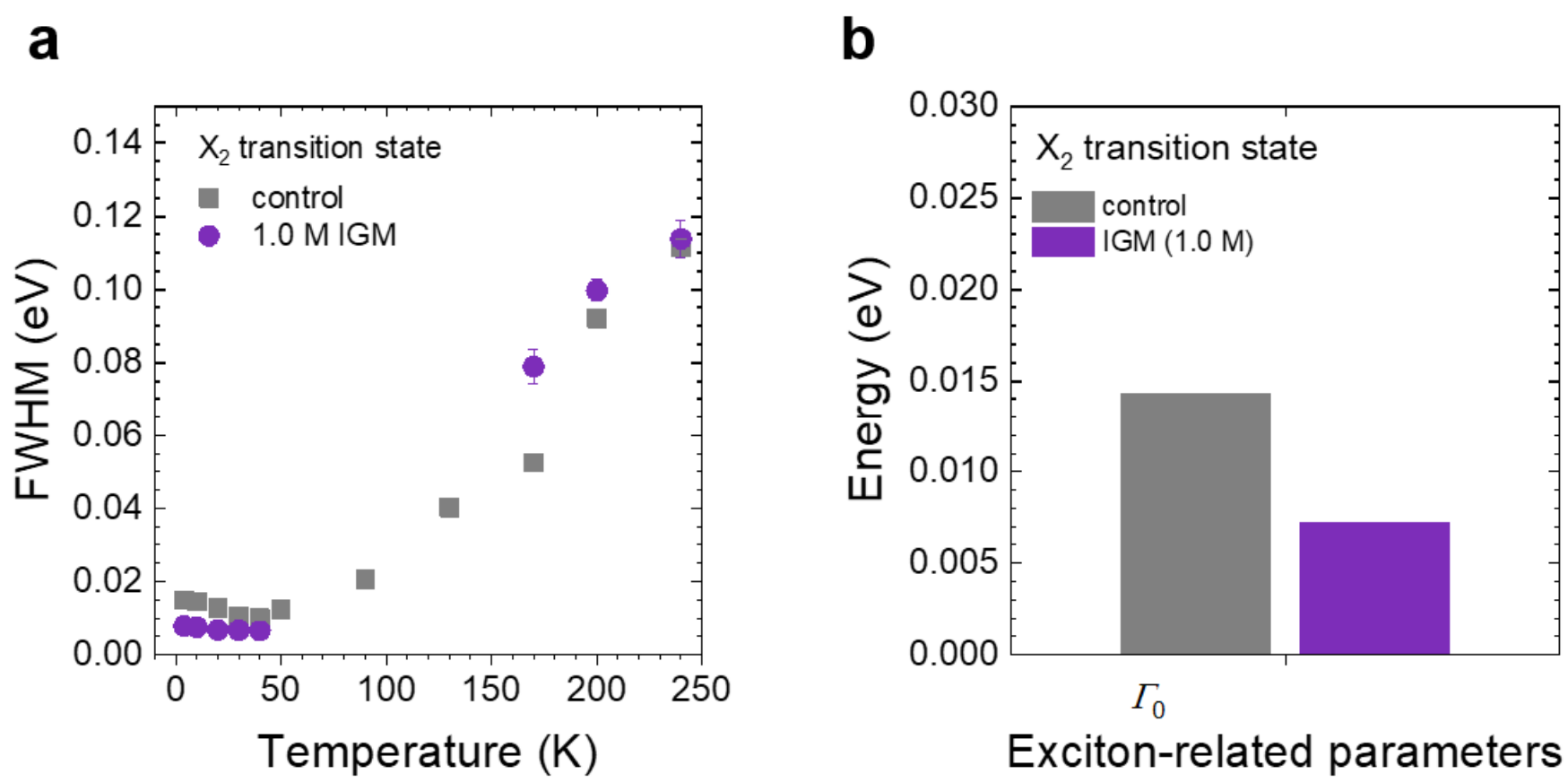

Supplementary Fig. 25 a, Temperature-dependent FWHM of steady-state PL peaks associated with X_2 transition state. **b,** The corresponding exciton-related parameters of Γ_0 and Γ_{LO} obtained from temperature-dependent FWHM analysis.

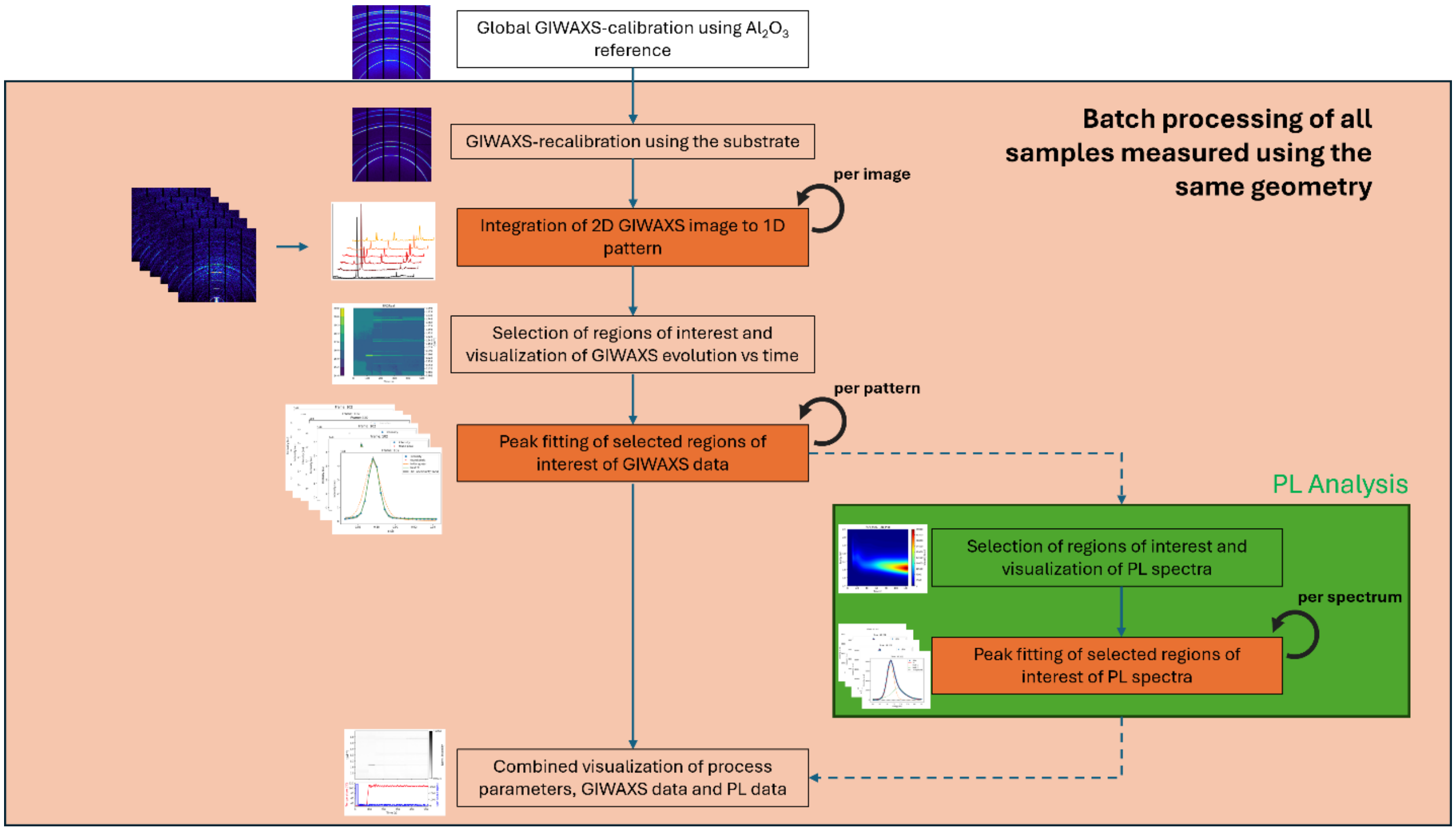

Supplementary Fig. 26 Workflow of *in-situ* data analysis using GIWAXS analysis coding package (MultiModalAnalysis). The integration of the PL data analysis into the workflow is optional and was not used for this work.

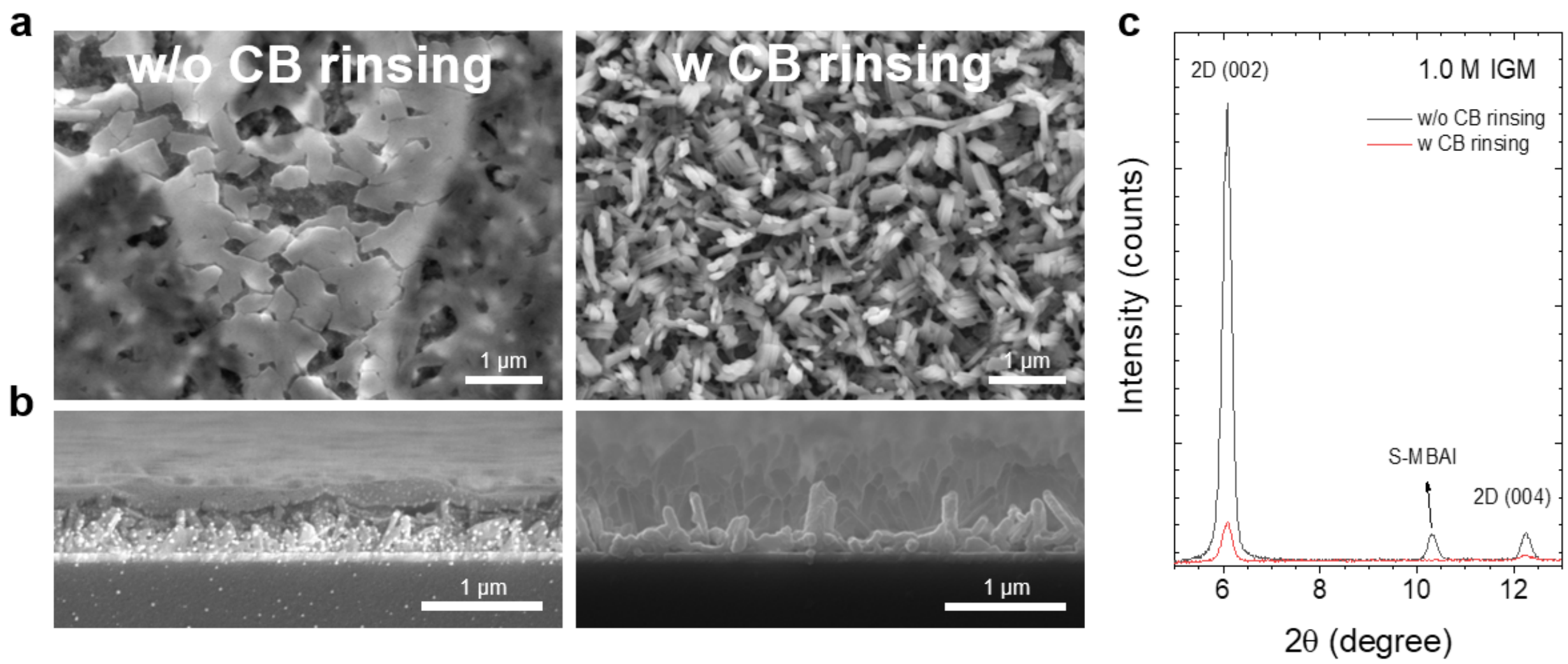

Supplementary Fig. 27 a-c, (**a**) Top-view and (**b**) cross-sectional SEM images and (**c**) XRD patterns for 1.0 M IGM films without and with CB rinsing step.

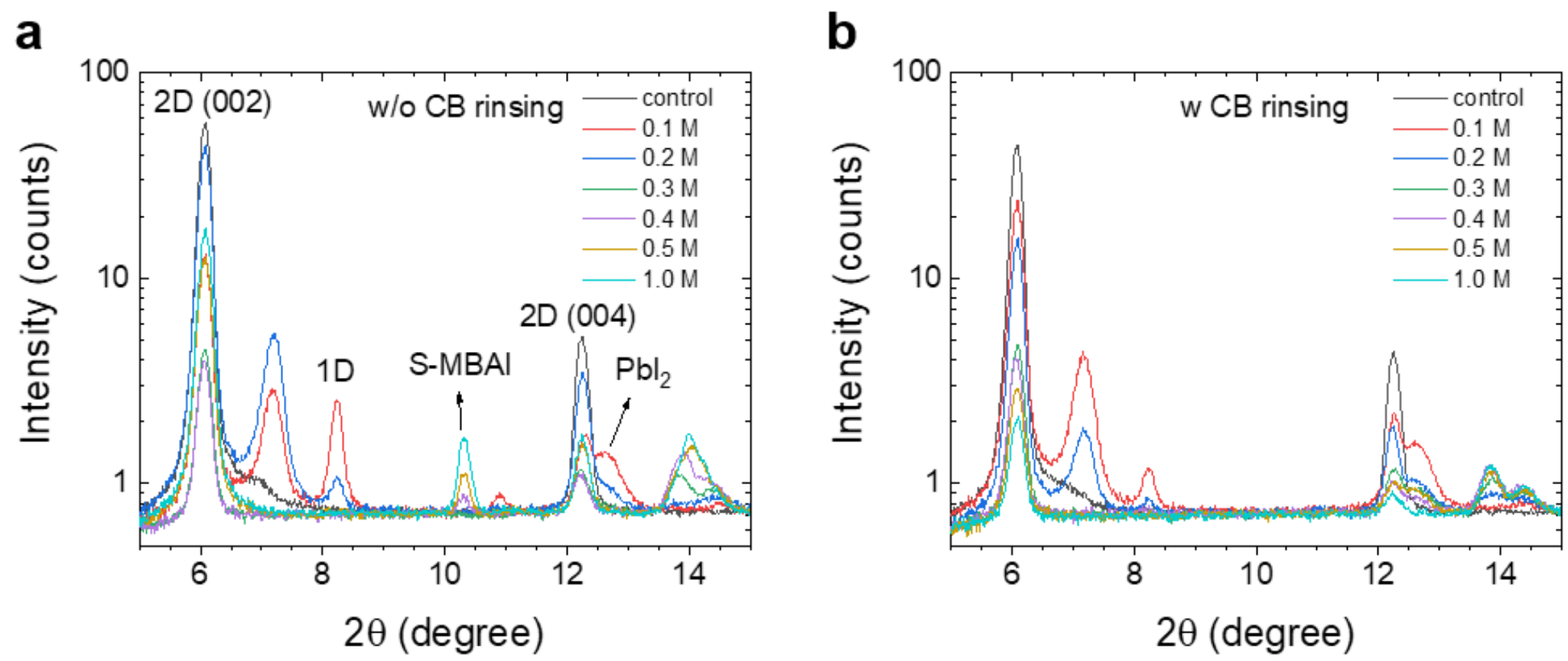

Supplementary Fig. 28 a,b, XRD patterns for control and IGM (0.1 ~ 1.0 M) films (**a**) without and (**b**) with CB rinsing step.

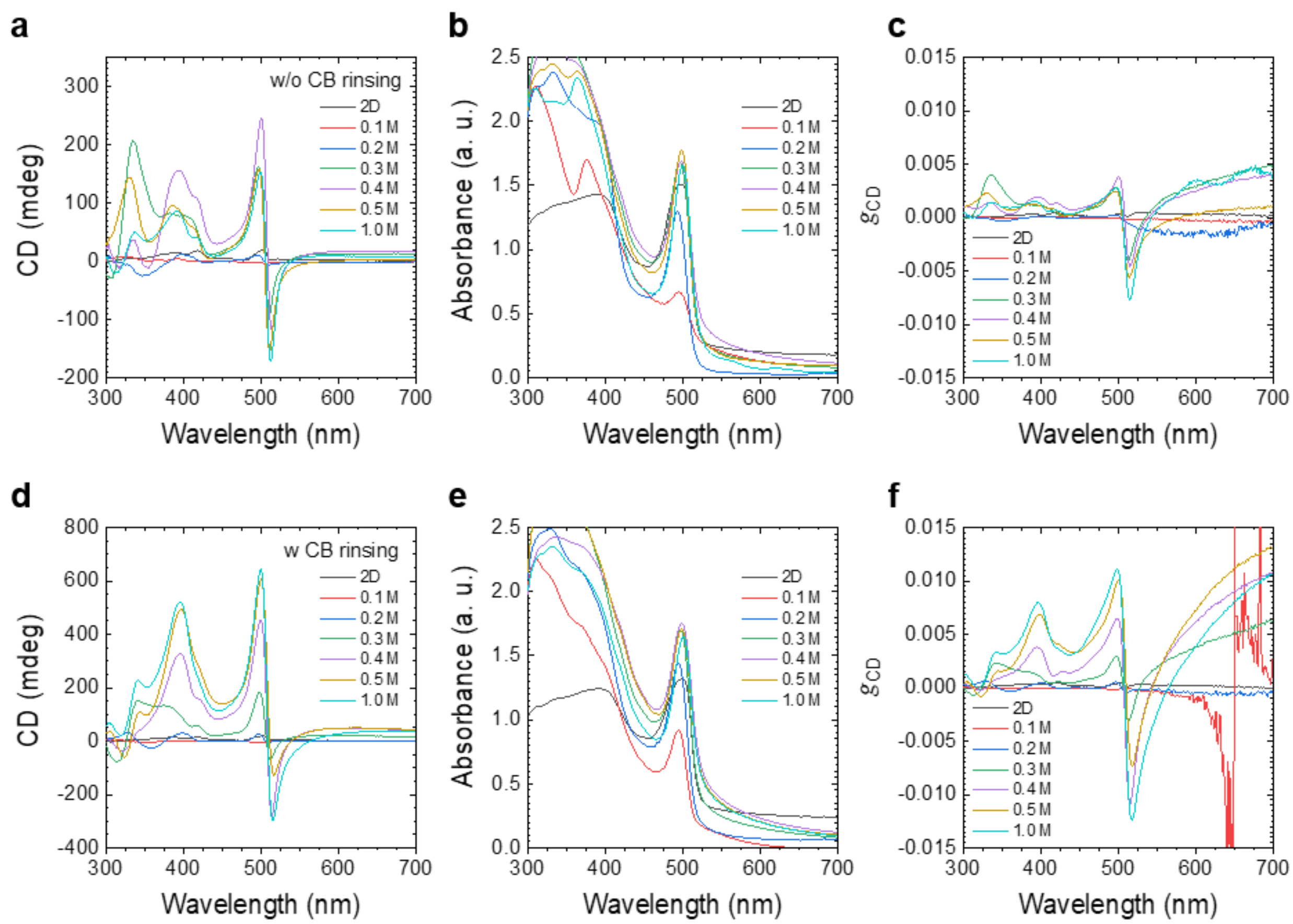

Supplementary Fig. 29 a-f, (**a,d**) CD, (**b,e**) absorbance, and (**c,f**) g_{CD} spectra for control and IGM (0.1 ~ 1.0 M) films (**a-c**) without and (**d-f**) with CB rinsing step.

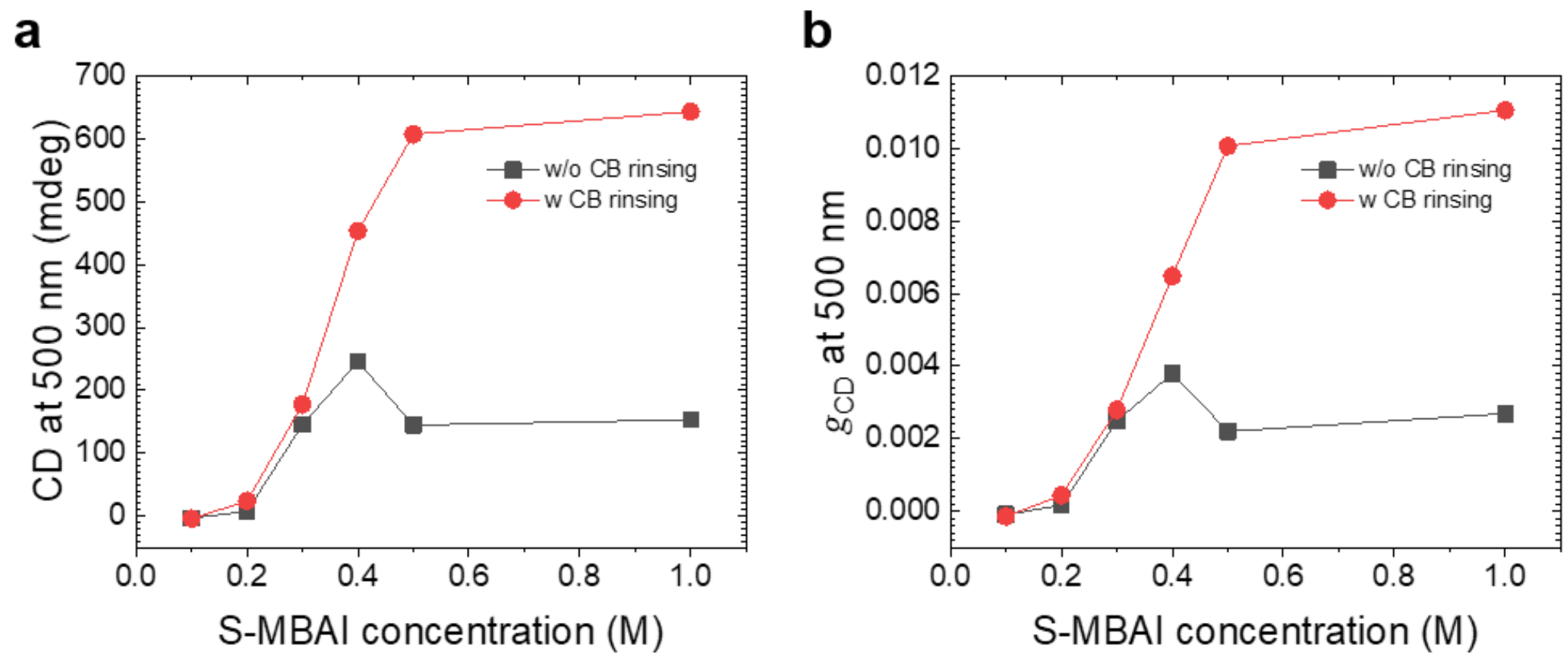

Supplementary Fig. 30 (**a**) CD intensity and (**b**) g_{CD} at 500 nm and for IGM (0.1 ~ 1.0 M) films without and with CB rinsing step.

Supplementary Table 1 Quantitative comparison of calculated defect-induced octahedral distortion in *S*-MBA_2PbI_4 crystal structure with 2 × 2 × 1 supercell. $\Delta\boldsymbol{\beta}$ represents the angular difference in Pb-I-Pb tilt angle between an inequivalent pair of neighboring octahedra. Ideal refers to a defect-free crystal structure.

Type of defects	$\Delta\boldsymbol{\beta}$ (°)
Ideal	6.47
V_I	6.01
V_{Pb}	4.73
V_{MBA}	5.11
I_i	4.84
Pb_i	4.38

Supplementary Table 2 Fitted parameters extracted from TRPL spectra. A bi-exponential decay function has been used ($y = y_0 + A_1*\exp(-(x-x_0)/t_1) + A_2*\exp(-(x-x_0)/t_2)$). The average lifetime was calculated using $(A_1t_1^2 + A_2t_2^2)/(A_1t_1 + A_2t_2)$ equation.

	control	0.3 M IGM	1.0 M IGM
A_1	0.7424 (92.90%)	0.6477 (90.19%)	0.53446 (90.56%)
t_1 (ns)	0.2492	0.2681	0.3193
A_2	0.05671 (7.10%)	0.0704 (9.81%)	0.05569 (9.44%)
t_2 (ns)	1.9423	1.9555	2.9142
$t_{average}$ (ns)	0.8810	1.0146	1.5841

Supplementary Table 3 Fitting parameters obtained from temperature-dependent PL linewidth analysis for X_1 transition state.

	control	1.0 M
Γ_0 [eV]	1.071×10^{-2}	0.670×10^{-2}
γ_{ac} [eV K^{-1}]	1.150×10^{-4}	1.011×10^{-4}
γ_{LO} [eV]	26.998	0.033
E_{LO} [eV]	0.141	0.018

Supplementary Table 4 Comparison of reported g_{CD} values for various materials.

Type	Target material	$\|g_{CD}\|$	Ref.
Plasmonic	Au nanoparticles	0.2 at 622 nm	Nature, 2018, 556, 360-365.
	Pt on chiral BINOL-CTAC-covered helical-micelle Au nanorods	0.1 ~ 0.2 at 600 ~ 1200 nm	Science, 2020, 368(6498), 1472-1477.
	Au nanoparticles	0.4 at ~ 600 nm	Nature, 2022, 601, 366-373.
Semiconductor	Assembled CdS helical clusters	0.025 ~ 0.05 at 300 ~ 350 nm	Science, 2025, 387, eado7201.
	Cd28S17 cluster	0.00016 at 304 nm	J. Am. Chem. Soc., 2025, 147(21), 17890-17901.
	D-/L-Cys Co_3O_4 nanoparticles	0.01 ~ 0.02 at 550 nm	Science, 2018, 259(6373), 309-314.
Organic	Synthesized helical organic fluorenones	0.014 at ~ 350 nm	Nature Communications, 2025, 16, 9083.
Chiral Perovskites	(*S*-MBA)$_4$Bi$_2$I$_5$Br$_5$ single crystal	0.025 at ~ 400 nm	Sci. Adv., 2025, 11, eads6123.
	(*R*-/*S*-BrBA)$_2$PbBr$_4$ single crystal	0.14 at 405 nm	ACS Appl. Mater. Interfaces, 2025, 17, 17127-17134.
	(*R*-/*S*-3APr)PbI$_4$ single crystal	0.00117 at 495 nm	Adv. Mater., 2025, 37(10), 2414199.
	(*R*-/*S*-BrsBA)$_2$PbBr$_4$ single crystal	0.023 at 354 nm	Adv. Funct. Mater., 2026, 36(12), e08904.
	(*R*-/*S*-NEA)PbI$_3$ film	0.04 at ~ 400 nm	Sci. Adv., 2020, 6(46), adb3274.
	(*R*-/*S*-MBA)$_4$Bi$_2$I$_{10}$ film	0.001 at ~ 530 nm	Nano Lett., 2024, 24(3), 1001-1008.
	($R_{0.4}S_{0.6}$/$R_{0.6}S_{0.4}$-MBA)BiI$_4$ film	~ 0.03 at 550 nm	J. Am. Chem. Soc., 2025, 147(40), 36420-36427.
	(*R*-/*S*-MBA)$_2$PbI$_4$ film with TADDOLs additive	~ 0.006 at ~ 500 nm	Sci. Adv. 2024, 10, eado5942.
	(*R*-MBA)$_2$PbI$_{2.7}$Br$_{1.3}$ crystals grown on AAO template substrate	0.002 at ~ 490 nm	Nature Communications, 2022, 13, 3259.
	(*R*-/*S*-PEA)PbI$_3$ film	~ 0.02 at 400 nm	Nature Communications, 2019, 10, 1927.
	S-MBA$_2$PbI$_4$ film	0.00054	Adv. Funct. Mater., 2025, 35(2), 2413041.
	S-2F-MBA$_2$PbI$_4$ film	0.00168 at ~ 500 nm	
	S-4F-MBA$_2$PbI$_4$ film	0.00031	
	(*R*-/*S*-MBA)$_2$PbI$_4$ film	< 0.001	Angew. Chem. Int. Ed., 2021, 60, 21434-21440.
	(*R*-/*S*-F-MBA)$_2$PbI$_4$ film	0.0004	
	(*R*-/*S*-Cl-MBA)$_2$PbI$_4$ film	0.0031 at ~ 470 nm	
	(*R*-/*S*-Br-MBA)$_2$PbI$_4$ film	0.0022 at ~ 470 nm	
	**(*R*-/*S*-MBA)$_2$PbI$_4$ film**	**0.012 at 500 nm**	**This work**

[**Supplementary Note 1**]

We conducted atomic force microscopy (AFM) measurements (**Supplementary Fig. 12**). The average surface roughness (R_a) and root mean square surface roughness (R_q) are quantitatively estimated on a 10 × 10 μm^2 scan area. The control film exhibits rough and poor coverage with R_a of 100.1 nm and R_q of 133.3 nm. In contrast, a lower surface roughness is observed for IGM samples (R_a of 26.88 nm and R_q of 33.44 nm for 0.3 M IGM, and R_a of 85.20 nm and R_q of 105.0 nm for 1.0 M IGM). Comparing different IGM concentrations, it was observed that the film roughness increases as the concentration of the *S*-MBAI solution increases, a trend consistent with the cross-sectional SEM results (**Supplementary Fig. 11**). This increased roughness can be attributed to the enhanced vertical growth of nanorod-like crystals.

To further characterize the uniformity of those films, we analyzed AFM line profiles with 4 lines per image (**Supplementary Fig. 13**). The control sample shows about one order of magnitude higher height fluctuations compared to the 0.3 M sample and slightly higher fluctuations compared to the 1.0 M sample. The line scans of the control sample seem to be dominated by large crystals with poor coverage while the IGM films show a more homogeneous distribution with smaller crystals.

[**Supplementary Note 2**]

In this section, we present the rationale for ruling out exciton coupling as the origin of the bisignate CD response:

i) <u>Misaligned zero-crossing in bisignate CD spectra with excitonic absorption maximum in absorbance</u>: Through temperature-dependent PL linewidth analysis (**Fig. 5c-f**) we estimate the electron-phonon coupling. It is well-known that a high phonon population is resulting in significant exciton scattering, leading to lower exciton coupling[1,2]. The E_{LO} obtained from PL linewidth fitting represents an energy required to create an LO phonon. Higher E_{LO} means low LO phonon population, ideally leading to strong exciton coupling[3]. If the appearance of the bisignate CD feature is dominated by exciton coupling, it should appear more clearly in the control film ($E_{LO,control}$ = 141 meV) than in the 1.0 M IGM film ($E_{LO,1.0\ M\ IGM}$ = 18 meV). This was not the case. In addition, the spin splitting energy extracted from low-temperature PL spectra at 4 K is the same, indicating that the exciton coupling strengths via electron-hole exchange interaction is identical. Therefore, the origin of the bisignate CD feature is predominantly affected by spin splitting via strong electrodynamic dipole coupling.

ii) <u>Dimensions of nanorods in IGM films</u>: In previous reports, chiral MBA_2PbI_4 single crystals typically form rod or wire shapes, with (110) or (111) planes oriented along the long axis[4,5]. This means that the (00*l*) facet is face-up onto the flat surface. In comparison, our nanorod-shaped crystals in the IGM film might have a similar orientation as chiral MBA_2PbI_4 single crystals with (110) or (111) planes oriented along a long axis. Despite the presence of single-crystal-like domains within the IGM film, the GIWAXS results exhibit semi-ring shapes of the scattering pattern along (002) plane owing to randomly distributed crystals. We approximate the crystal size associated with the (002) facet in the IGM film, to be around 300 ~ 500 nm along the long axis and 70 ~ 120 nm along the short axis. These dimensions are not comparable to the Bohr radius (< 20 nm, typically for quantum dots). Furthermore, excitons are delocalized in in-plane of 2D layered crystal structures and possibly transferred between their inorganic interlayers with Therefore, there is likely no significant exciton coupling in our system, and we assume that the contribution of the exciton coupling to the CD response is minor.

[**Supplementary Note 3**]

The dimensionless oscillator strength (f) is an indicator to quantitatively estimate the probability of the electronic transition, which in turn determines the total absorption or emission strength. This f-value is determined by the transition electric dipole moment (μ), which can be expressed by the following equation for a single unit cell[6],

$$f_{12} = \frac{2m_e}{3h^2}(E_2 - E_1)\sum_{\alpha = x,y,z}|\langle 1m_1|r|2m_2\rangle|^2 = \frac{2m_e}{3h^2e^2}\Delta E_{12}|\vec{\boldsymbol{\mu}}_{12}|^2 \quad (1)$$

where, h is the reduced Planck constant, m_e is the mass of an electron. The energy and sublevel of transition states from the initial state 1 to the final state 2 are denoted as E_x and m_x, respectively. In the case of scaling up a single unit cell to multiple N unit cells (N: the number of unit cells), the individual transition electric dipole moments interfere constructively when these N unit cells are close enough to oscillate in-phase. The oscillator strength equation for the N unit cells case is given below[7-9]. This indicates that the oscillator strength dramatically increases if there are strong constructive interferences owing to the electrodynamic coupling of the transition dipoles.

$$f_{total} = \frac{2m_e}{3h^2e^2}\Delta E_{12}\left|\sum_{i=1}^{N}\vec{\boldsymbol{\mu}}_{12}\right|^2 \propto \left(N \times \vec{\boldsymbol{\mu}}_{single}\right)^2 \quad (2)$$

With similar concepts based on the quantum-mechanical theory, the rotational strength (R) can be expressed by the following equation below. However, unlike the oscillator strength, the transition electric dipole moment (μ) and magnetic dipole moment (m) are involved in the expression of R[10-13].

$$R = Im\{\vec{\boldsymbol{\mu}} \cdot \vec{\boldsymbol{m}}\} \quad (3)$$

According to these theoretical models for multiple unit cells, the strong long-range electrodynamic coupling of dipoles induced by the constructive interference magnifies rotational strength, leading to the amplification of the CD response. To induce the electrodynamic coupling of dipoles, coherence of crystallite domains is critical[14,15]. The coherent crystallite domains, with uniform orientation and few crystallographic defects, provide a structurally in-phase environment of dipoles with their neighbors, indicating that this structural perfection allows the excitonic transition dipoles to couple coherently. Furthermore, high crystallinity and less defects facilitate the prolonged carrier/exciton diffusion lengths, resulting in the enhancement of the electronic coupling[16,17].

[**Supplementary Note 4**]

The rinsing step ensures the removal of residual gel phase or MBAI and is referred to as CB rinsing step. **Supplementary Fig. 27** shows IGM films without and with CB rinsing. Before rinsing some covering layer is observed which disappears after rinsing. XRD confirms that after the CB rinsing step residual *S*-MBAI disappears. As seen in the XRD patterns (**Supplementary Fig. 28**) for films w/o CB rinsing step, the diffraction peak at 10.3° corresponding to *S*-MBAI crystalline phase gradually appears as the *S*-MBAI concentration increases above 0.4 M. There is no *S*-MBAI associated peak in films with CB rinsing step. Chiroptical properties corresponding to these films are illustrated in **Supplementary Fig. 29**. CD intensities and g_{CD} at 500 nm are shown in **Supplementary Fig. 30**.

In the IGM synthesis route, the dissolution-recrystallization mechanism is the primary way in which the in-situ formed ionic gel phase on the film surface is involved during the spin-coating and annealing processes. This gel phase does not solidify when cooled down to room temperature. We found that the crystallization of this gel phase is triggered by exposure to the ambient air. An oily surface was observed for as-annealed films under the inert N_2 condition, indicating that the ionic gel phase still exists at the film surface without crystallizing. This indicates that the dissolution-recrystallization pathway might still be proceeding at the film surface. Therefore, these findings suggest that the presence of the residual ionic gel phase at the film surface is linked to the deformation of isotropic nanostructured crystals leading to lower CD response (**Supplementary Fig. 30**).

Supplementary References

1. Alvertis, A. M., Haber, J. B. & Neaton, J. B. Phonon screening and dissociation of excitons at finite temperature from first principles. *PNAS* **121**(30), e2403434121 (2024).

2. Sarkar, S., Kamath, N. S., Gayen, K. & Pal, S. K. Exciton-phonon coupling in quasi-two-dimensional Ruddlesden-Popper perovskites: impact of a mixed-phase structure. *Nanoscale* **17**, 10771-10783 (2025).

3. Gramlich M., Lampe, C., Drewniok J. & Urban, A. S. How exciton-phonon coupling impacts photoluminescence in halide perovskite nanoplatelets. *J. Phys. Chem. Lett.* **12**(46), 11371-11377 (2021).

4. Liu, Z. *et al.* Chiral hybrid perovskite single-crystal nanowire arrays for high-performance circularly polarized light detection. *Adv. Sci.* **8**(21), 2102065 (2021).

5. Zhao, Y. *et al.* Chiral 2D/quasi-2D perovskite heterojunction nanowire arrays for high-performance full-stokes polarization detection. *Adv. Optical Mater.* **11**(19), 2301239 (2023).

6. Hilbom, R. C. Einstein coefficients, cross sections *f* values, dipole moments, and all that. *Am. J. Phys.* **50**, 982-986 (1982).

7. Franta, D., Nečas, D. & Zajíčková, L. Application of Thomas-Reiche-Kuhn sum rule to construction of advanced dispersion models. *Thin Solid Films* **534**, 432-441 (2013).

8. Lestrange, P. J., Egidi F. & Li, X. The consequences of improperly describing oscillator strengths beyond the electric dipole approximation. *J. Chem. Phys.* **143**, 234103 (2015).

9. Zheng, L., Polizzi, N. F., Dave, A. R., Migliore, A. & Beratan, D. N. Where is the electronic oscillator strength? Mapping oscillator strength across molecular absorption spectra. *J. Phys. Chem. A.* **120**(11), 1933-1943 (2016).

10. Tinoco, I. & Woody, R. W. Optical rotation of oriented helices. IV. A free electron on a helix. *J. Chem. Phys.* **40**, 160-165 (1964).

11. Caricato, M. Orbital analysis of molecular optical activity based on configuration rotatory strength. *J. Chem. Theory Comput.* **11**(4), 1349-1353.

12. Berova, N., Bari, L. D. & Pescitelli, G. Application of electronic circular dichroism in configurational and conformational analysis of organic compounds. *Chem. Soc. Rev.* **36**, 914-931 (2007).

13. Albano, G., Pescitelli, G. & Bari, L. D. Chiroptical properties in thin films of π-conjugated systems. *Chem. Rev.* **120**(18), 10145-10243 (2020).

14. Zhang Y. *et al.* Visualizing coherent intermolecular dipole-dipole coupling in real space. *Nature* **531**, 623-627 (2016).

15. Muller, E. A., Gray, T. P., Zhou, Z. & Raschke, M. B. Vibrational exciton nanoimaging of phases and domains in porphyrin nanocrystals. *PNAS* **117**(13), 7030-7037 (2020).

16. Pandya, R. et al. Femtosecond transient absorption microscopy of singlet exciton motion in side-chain engineered perylene-diimide thin films. J. Phys. Chem. A. 124(13), 2721-2730 (2020).

17. Kranz, J. J. & Elstner, M. Simulation of singlet exciton diffusion in bulk organic materials. J. Chem. Theory Comput. 12(9), 4209-4221 (2016).
