# Supplementary material for: Ionic gel-mediated synthesis of nanostructured chiral 2D halide perovskites with amplified chiroptical response
Source: Nat Commun. 2026 Aug 29;17:10316. doi: 10.1038/s41467-026-77274-w (PMC13620143; doi:10.1038/s41467-026-77274-w)
Supplement: Supplementary file 2 [file 41467_2026_77274_MOESM2_ESM.pdf]

Peer Review File

Ionic gel-mediated synthesis of nanostructured chiral 2D halide perovskites with amplified chiroptical response

Corresponding Author: Dr Carolin Sutter-Fella

This file contains all reviewer reports in order by version, followed by all author rebuttals in order by version.

Version 0:

Reviewer comments:

Reviewer #1

(Remarks to the Author)
Lee and colleagues demonstrated an ionic gel-mediated (IGM) nucleation and crystallization strategy of chiral 2D perovskites. This two-step spin-coating method yields a uniform rod-like film morphology, thereby enhancing the electron-hole exchange interaction and leading to a significant amplification of the circular dichroism (CD) response. Although the reported chiroptical property of IGM-chiral perovskites is promising, several issues still require clarification, including random crystal orientation, circularly polarized emission, and potential practical applications etc. The detailed comments are provided below:

1. Authors showed optical photographs of films in Figure 1b to compare their optical transparency as evidence of homogenous film morphology. However, optical transparency is strongly dependent on film thickness. It is therefore recommended to show the thickness of control films to rule out the possibility that their poor transparency arises from greater film thickness.

2. GIWAXS results indicate that IGM films exhibit random and isotropic crystal orientation. Their contribution to the observed anisotropic CD response is unclear. How such isotropic crystal orientation interacts or competes with their anisotropic film morphology in determining the chiral optical properties should be discussed and investigated in depth.

3. The authors discussed the possible LDLB effect of the CD response in chiral perovskites. However, previous relevant studies were not cited properly, e.g. JACS 2022 (Lu); Chem. Rev. 2020 (Di Bari). Please discuss more on the fundamental origin of the CD amplification. Was it due to the anisotropic structure, enhanced light-matter interaction, or there are fundamental differences in their electronic structures, such as an increased spin-splitting? That is, is it because of a morphological effect or electronic effect?

4. Authors had investigated the effect of ionic gel concentration on the crystallization behavior and chiroptical property of chiral 2D perovskites. However, several important factors that can also significantly influence crystallization were not included in the study. For example, does the gel droplet volume at a fixed concentration, or the thickness of PbI_2 layer still affect perovskite growth and the resulting CD response? In addition, how do the results differ between directly dropping an MBA-IPA solution and dropping a pre-formed gel obtained by evaporating IPA? More detailed and systematic experiments should be designed and conducted to fully elucidate the crystallization mechanism and its relationship to chiroptical response.

5. IGM chiral 2D perovskites showed the large gcd values, which form the basis for circularly polarized emission. Authors are therefore strongly encouraged to include circularly polarized luminescence measurements in the manuscript to further highlight the potential of IGM approach.

6. In Supplementary Fig. 3c, does the gel formed after the IPA evaporation solidify during the cooling process or under the ice condition? If solidification occurs, XRD patterns of these solids could further help identify the proposed 1D intermediates.

7. Chiral 2D perovskites are promising semiconductors for electronic applications. However, the IGM films appear to contain numerous pinholes. AFM measurements should be performed to further evaluate the surface roughness of films. Also, study of spin-dependent charge transports would be necessary to substantiate their potential for practical spintronic applications.

8. Please clarify if there are any unreacted PbI_2 or MBAI left on the film and they the residues play any roles in their chiroptical and optoelectronic properties. Please discuss the broad applicability of this IGM method, in particular, from the perspective of phase purity.

9. As Nature Communications is a multidisciplinary journal, it would be beneficial for the authors to include demonstration of potential applications, such as circularly polarized photodetectors in the manuscript to broaden its appeal to a wider readership.

10. Authors should carefully check their manuscript for formatting issues. For example, there are redundant punctuation marks in line 37, incorrect characters in line 198, and inconsistent formatting in line 230.

Reviewer #2

(Remarks to the Author)
This manuscript presents a study on the ionic gel-mediated (IGM) synthesis of nanostructured chiral 2D halide perovskites, reporting an enhanced chiroptical response with a dissymmetry factor (gCD) up to ～10^{-2}—higher than many values previously reported for similar systems. The two-step IGM approach appears to offer improved control over film morphology compared to conventional one-step methods, and the experimental data are generally well presented. Overall, the work is scientifically sound, clearly written, and makes a valuable contribution to the field of chiral optoelectronic materials. I would like to recommend the publication of this manuscript in Nature Communications after the following revisions:
1. The manuscript mentions experiments with racemic MBAI but doesn't show CD data for it. It would strengthen the argument if the authors explicitly confirm that racemic films show no CD signal—this helps rule out artifacts.
2. The authors reasonably dismiss exciton coupling and suggest Rashba-type spin splitting as the origin of the bisignate CD lineshape. However, this remains somewhat speculative. Could they briefly discuss whether magnetic circular dichroism (MCD) or magneto-optical measurements could further test this hypothesis?
3. Figure 4e shows gCD distribution, which is good. But how many independent film batches were used to generate this statistic?
4. The manuscript mentions using a "custom-made ionic gel," but doesn't specify its exact chemical components or preparation method. Since the IGM process is central to this work, readers will need enough detail to reproduce it.
5. Chiral perovskites often suffer from environmental instability. Did the authors test the CD signal or PL intensity of their films after exposure to air?
6. The gCD value of ～10^{-2} is impressive, but it would help readers gauge its significance if the authors briefly compare it to other state-of-the-art chiral semiconductors (e.g., chiral quantum dots, organic-inorganic hybrids, or chiral MOFs).

Reviewer #3

(Remarks to the Author)
In this manuscript, the authors demonstrated an ionic gel-mediated two-step spin coating method for nanostructured chiral MBA_2PbI_4 thin films. Based on in-situ GIWAXS patterns and absorption spectra, the authors unveiled that the MBAI ionic gel facilitates the formation of 1D [PbI_3^--MBA^+] gel intermediate in stage II and thus the rod-like growth in the subsequent annealing process. Due to the high crystallinity and low defect density, the as-grown nanorod film shows the highest dissymmetry factor gCD of 1.2 × 10-2 among pristine chiral 2D perovskites, two orders of magnitude higher than that of the conventional one-step spin-coated film. These results would promote the design and fabrication of chiral 2D perovskite films towards spin-resolved optoelectronic applications. Therefore, to be accepted for publication in Nature Communications, the manuscript should address the questions listed below first:

1. The preparation of the MBAI ionic gel solution is pretty easy, simply dissolving the MBAI powder into the IPA. Is this a universal method? The authors should show more examples of this ionic gel-mediated method.
2. The authors assigned the enhanced chirality in IGM films partially to lower defect density, which is supported by the narrower PL linewidth and longer PL lifetime. 1) According to Figure 5e, the FWHMs of IGM films are actually larger than those of control films above ~90K. I assume this is a fitting issue. The authors need to double-check it. 2) According to Figure 5b, there are two PL decay channels, in which the fast radiative recombination takes the dominant role. Therefore, the average lifetime is more related to the radiative recombination and is not a good indicator for defect-related non-radiative recombination. It is suggested that the authors show the fitting results for TRPL and compare the non-radiative lifetime.
3. Following the previous question, another possible reason for enhanced chirality is the improved crystallographic orientation. The reported dissymmetry factor gCD value is even comparable to that of the chiral 2D perovskite single crystal (ACS Appl. Mater. Interfaces 2025, 17, 17127−17134). It is highly suggested that the authors provide a table to summarize the chiroptical metrics for 2D perovskites.
4. What are the fitted ELO energies in Figure 5e? Do they correspond to different Raman modes for control and IGM films, respectively?

Version 1:

Reviewer comments:

Reviewer #1

(Remarks to the Author)
The authors have addressed most of my previous comments. However, there are still three issues of this manuscript.
1. It seems that this method is not universal, and only applies to MBA2PbI4 system, which limits its applicability.
2. It seems that the chiral MBA2PbI4 films, although displaying large gCD, show no CPL or spin-dependent charge transport, which limits their practical applications.
3. The authors stated that the surface roughness was improved compared to the control sample. However, how was the control film fabricated? Literature reports have shown a smooth surface of chiral MBA2PbI4 film prepared by the traditional one-step method.
Can the authors further address or discuss these points?

(Remarks on code availability)

Reviewer #2

(Remarks to the Author)
The authors have responded thoroughly and convincingly to most of the reviewers' comments, and the manuscript has been significantly improved through the necessary revisions and clarifications. Only minor editorial adjustments—such as copyediting for language style and a final verification of figure captions—may be needed at the proof stage. Nevertheless, I would like to request that the following two points be addressed before the paper is considered for publication:
1. The link between enhanced crystallinity/morphology and amplified Rashba splitting is plausible but indirect. Can the authors provide even qualitative theoretical support (e.g., a simple model or calculation) to strengthen this key claim?
2. While reproducibility across batches is shown, how many spots per film were measured for gCD? Please confirm the signal uniformity across the nanostructured film to rule out local artifacts.

(Remarks on code availability)
While I did not personally run the code, the provided documentation—especially the step-by-step setup guide and usage description—suggests that a user could reasonably install and execute it. Thus, the results of the associated paper are likely reproducible with this resource.

Reviewer #3

(Remarks to the Author)
The authors addressed all the comments. Now the paper is ready for publication.

(Remarks on code availability)

Version 2:

Reviewer comments:

Reviewer #1

(Remarks to the Author)
The authors have addressed my comments. Although the strategy is limited to a specific composition and the resulting chiral 2D perovskite displays no CPL, the enhancement of CD is quite interesting.

(Remarks on code availability)

Reviewer #2

(Remarks to the Author)
The authors have carefully addressed all comments raised in a convincing manner. Therefore, I would like to recommend publication of this paper.

(Remarks on code availability)

Open Access This Peer Review File is licensed under a Creative Commons Attribution 4.0 International License, which permits use, sharing, adaptation, distribution and reproduction in any medium or format, as long as you give appropriate credit to the original author(s) and the source, provide a link to the Creative Commons license, and indicate if changes were made.
In cases where reviewers are anonymous, credit should be given to 'Anonymous Referee' and the source.
The images or other third party material in this Peer Review File are included in the article's Creative Commons license, unless indicated otherwise in a credit line to the material. If material is not included in the article's Creative Commons license and your intended use is not permitted by statutory regulation or exceeds the permitted use, you will need to obtain permission directly from the copyright holder.
To view a copy of this license, visit https://creativecommons.org/licenses/by/4.0/

A point-by-point response

Journal: Nature Communications
Manuscript ID: NCOMMS-25-86761-T
Title: Ionic gel-mediated synthesis of nanostructured chiral 2D halide perovskites with amplified chiroptical response
Author(s): Do-Kyoung Lee, Raphael F. Moral, Finn Babbe, Tim Kodalle, Fabrice Roncoroni, Harishankar Jayakumar, Yen Jea Lee, Brooks A. Abel, Craig P. Schwartz, Carolin M. Sutter-Fella*

First of all, we thank reviewers and editors for their valuable comments on our manuscript entitled "Ionic gel-mediated synthesis of nanostructured chiral 2D halide perovskites with amplified chiroptical response" (corresponding author: Carolin M. Sutter-Fella). Thank you for giving us the opportunity to address the points raised. We strive to improve the manuscript based on the feedback received. Here we have addressed the queries from the reviewers and revised the manuscript according to the reviewer's comments. The revised parts are highlighted in yellow in the revised manuscript.

Reviewer #1 (Remarks to the Author):

Comments:
Lee and colleagues demonstrated an ionic gel-mediated (IGM) nucleation and crystallization strategy of chiral 2D perovskites. This two-step spin-coating method yields a uniform rod-like film morphology, thereby enhancing the electron-hole exchange interaction and leading to a significant amplification of the circular dichroism (CD) response. Although the reported chiroptical property of IGM-chiral perovskites is promising, several issues still require clarification, including random crystal orientation, circularly polarized emission, and potential practical applications etc. The detailed comments are provided below:

We highly appreciate the reviewers' constructive feedback and thank them for taking their time to assess our manuscript. The detailed comments were all addressed below and new measurement data was included to back up our discussions. Please find a point-by-point response below.

1. Authors showed optical photographs of films in Figure 1b to compare their optical transparency as evidence of homogenous film morphology. However, optical transparency is strongly dependent

on film thickness. It is therefore recommended to show the thickness of control films to rule out the possibility that their poor transparency arises from greater film thickness.

Ans) We thank the reviewer for raising this point. We agree that thickness can partially influence optical transparency. The thickness of the control as well as 0.3 M IGM films was estimated from cross-sectional SEM images, as can be seen in **Fig. R1**. (please see **Supplementary Fig. 11** in the revised Supplementary Information as well)

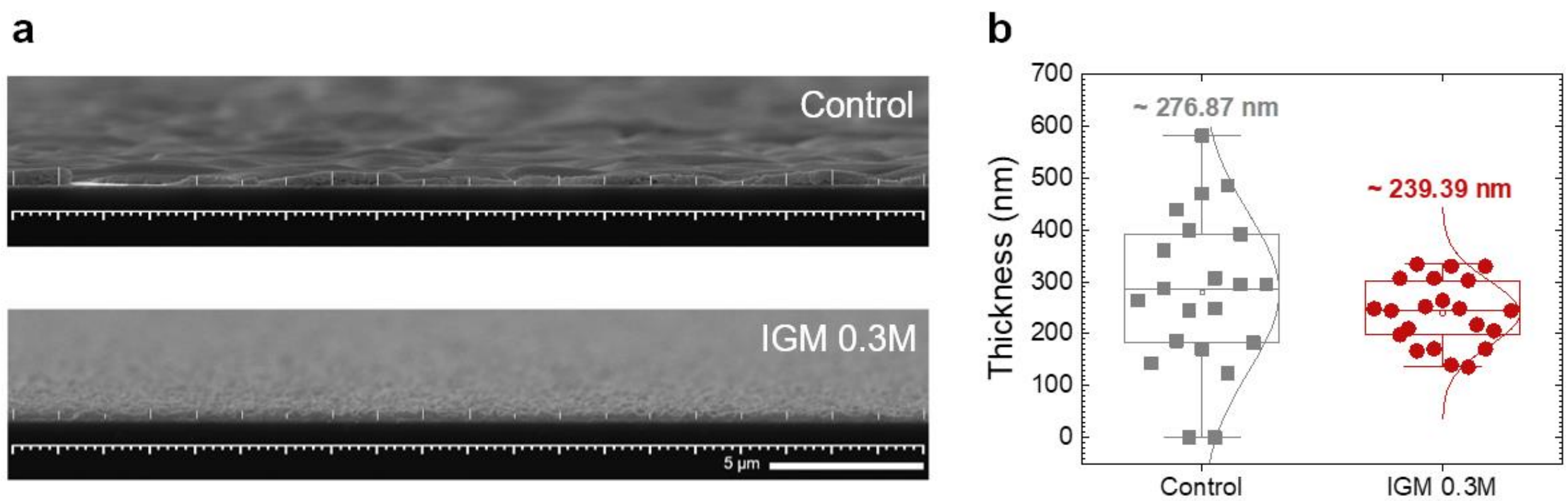

Fig. R1. a, Cross-sectional SEM images for control and 0.3M IGM films. **b**, Statistics of the distribution of film thicknesses from cross-sectional SEM images by estimating 21 spots.

We would like to note that precisely estimating the thickness for the control film is challenging because of its inhomogeneous crystal size and poor coverage, resulting in a broad distribution shown in **Fig. R1b**. The average thickness is 276.87 nm for control and 239.39 nm for 0.3 M IGM, respectively, which is not a significant difference. Please also see the newly added comprehensive AFM data in **Supplementary Fig. 12-13** (answer for Q7).

To rule out thickness as the cause of optical transparency, we combined both data from cross-sectional SEM and absorbance measurements for concentration-dependent *S*-MBA_2PbI_4 films (0.05 M ~ 0.40 M). We prepared coating solutions for a one-step deposition method, where *S*-MBAI and PbI_2 precursors at a ratio of 2:1 were dissolved in acetonitrile with different concentrations. The precursor coating solution was loaded on the substrate, and then spin-coated at 2000 rpm for 30 s. The as-coated film was immediately transferred to the hot plate, and then annealed at 70 °C for 10 min. The use of acetonitrile as the solvent, instead of the typical polar aprotic solvents such as DMF, DMSO, and their mixture, leads to a smooth and highly uniform morphology of chiral MBA_2PbI_4 perovskite film[R1]. In this case, the influence of surface roughness was excluded, which is the primary rationale for our use of acetonitrile here. **Fig. R2** shows a variation in film thickness and the resulting absorbance spectra as a function of precursor coating solution concentration. Since the optical bandgap of chiral MBA_2PbI_4 perovskite is around 2.2 to 2.4 eV[R2], this material exhibits no absorption at wavelengths below 550 nm. Even with thicker films, the scatter intensity above 550 nm wavelength range does not increase. It indicates that film

thickness is not a governing parameter on scattering. Therefore, we conclude that scattering in the absorbance spectra is predominantly determined by film morphology traits such as roughness, uniformity, and coverage rather than film thickness.

References

[R1] Scalon, L. *et al*. Tuning phase purity in chiral 2D perovskites, *Adv. Optical Mater.* **12**(2), 2300776 (2024).

[R2] Zhang, H. *et al*. Self-powered circularly polarized photodetectors based on chiral (R/S-MBA)$_2$PbI$_4$ perovskite, *Adv. Optical Mater.* **13**, e01133 (2025).

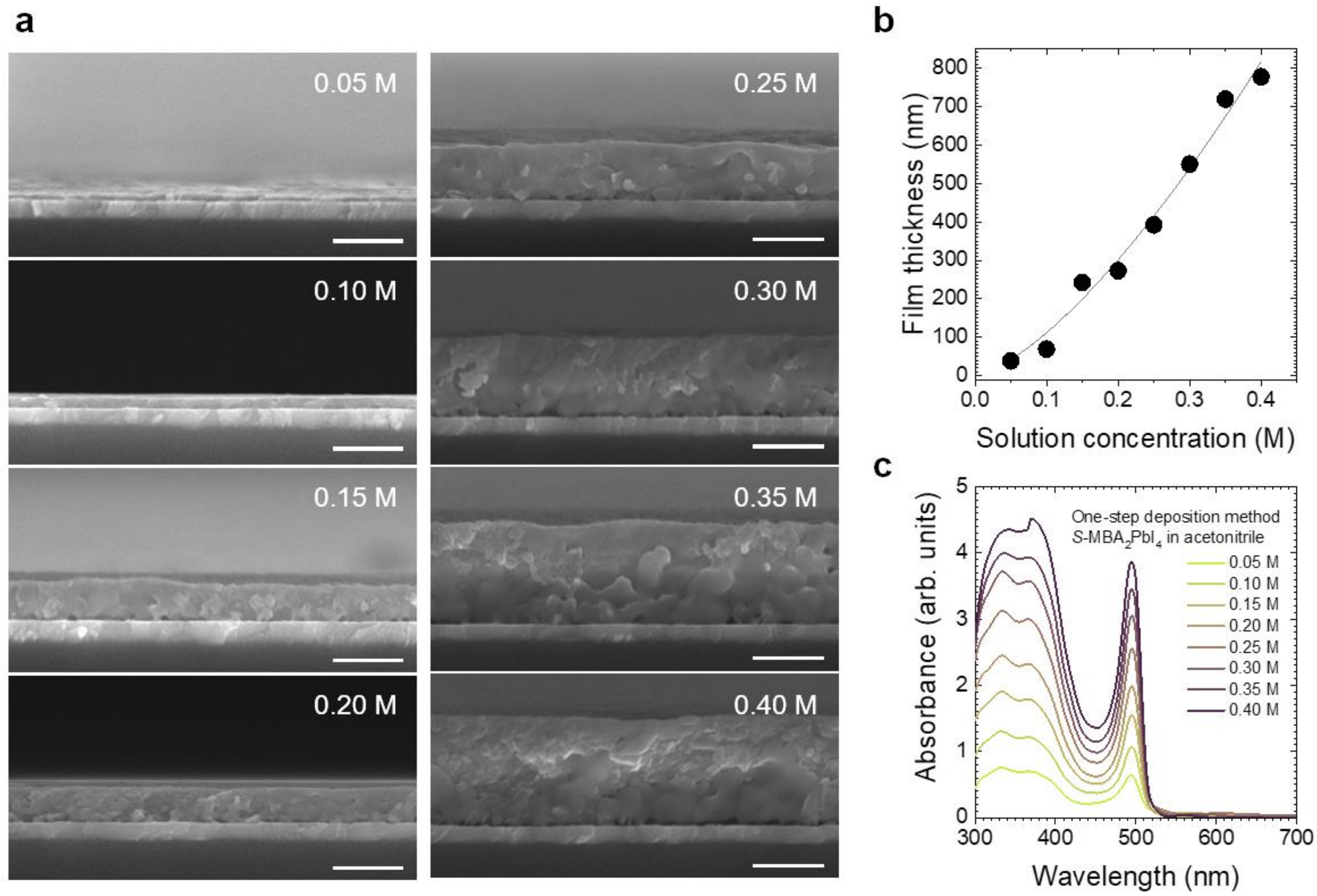

Fig. R2. a-c, (**a**) Cross-sectional SEM images, (**b**) film thickness profile, and (**c**) absorbance spectra for S-MBA$_2$PbI$_4$ perovskite films depending on the concentration of precursor coating solutions. The scale bar in cross-sectional SEM images is 500 nm.

We added the following to the revised manuscript and supplementary information.

On page 4-5 in the revised manuscript,

"To synthesize the chiral 2D MBA$_2$PbI$_4$ thin films, we utilized one-step and two-step deposition techniques, schematically illustrated in **Fig. 1a**. For the one-step method, the coating solution containing both chiral methylbenzylammonium iodide (MBAI) and PbI$_2$ precursors, with a 2:1

molar ratio dissolved in a mixture of dimethylformamide (DMF) and dimethyl sulfoxide (DMSO) was spin-coated onto an indium-doped tin oxide (ITO) substrate, and then annealed at 70 ℃ for 10 min. For the two-step method, a 0.5 M PbI_2 solution was first spin-coated onto the bare ITO substrate, followed by thermal annealing at 70 ℃ for 1 min, forming an approximately 90 nm thick PbI_2 film (**Supplementary Fig. 1**). Second, an MBAI solution (MBAI salts dissolved in isopropyl alcohol (IPA)) was dropped onto the spinning PbI_2 substrate and subsequently annealed at 70 ℃ for 10 min. The films will be referred to as 'control' and 'ionic gel-mediated (IGM)' for those using the one-step and two-step deposition methods, respectively. The control shows an opaque appearance with inhomogeneous, large macroscopic crystals, indicating poor coverage and rough film morphology (**Fig. 1b**). In sharp contrast, the IGM film exhibits a semitransparent and reflective film appearance, indicating a homogeneous and smooth film surface, with uniform rod-like nanoscale crystallites. The average thickness is 276.87 nm for control and 239.39 nm for 0.3 M IGM, which is not a significant difference (**Supplementary Fig. 2**). It indicates that the optical transparency of the films are related to their coverage. It is in good agreement with the enhanced optical scattering absorption at above 550 nm in absorbance spectra (**Supplementary Fig. 3a)**."

In the revised Supplementary Information,

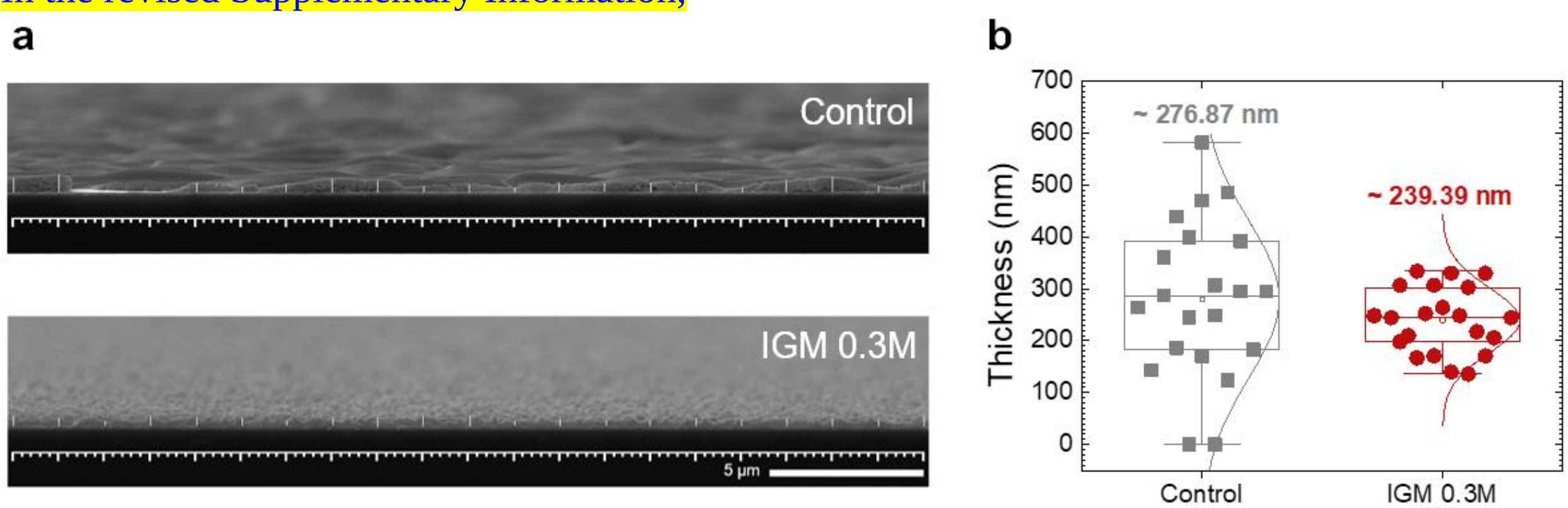

Supplementary Fig. 2. a, Cross-sectional SEM images for control and 0.3M IGM films. **b,** Statistics of the distribution of film thicknesses from cross-sectional SEM images by estimating 21 spots.

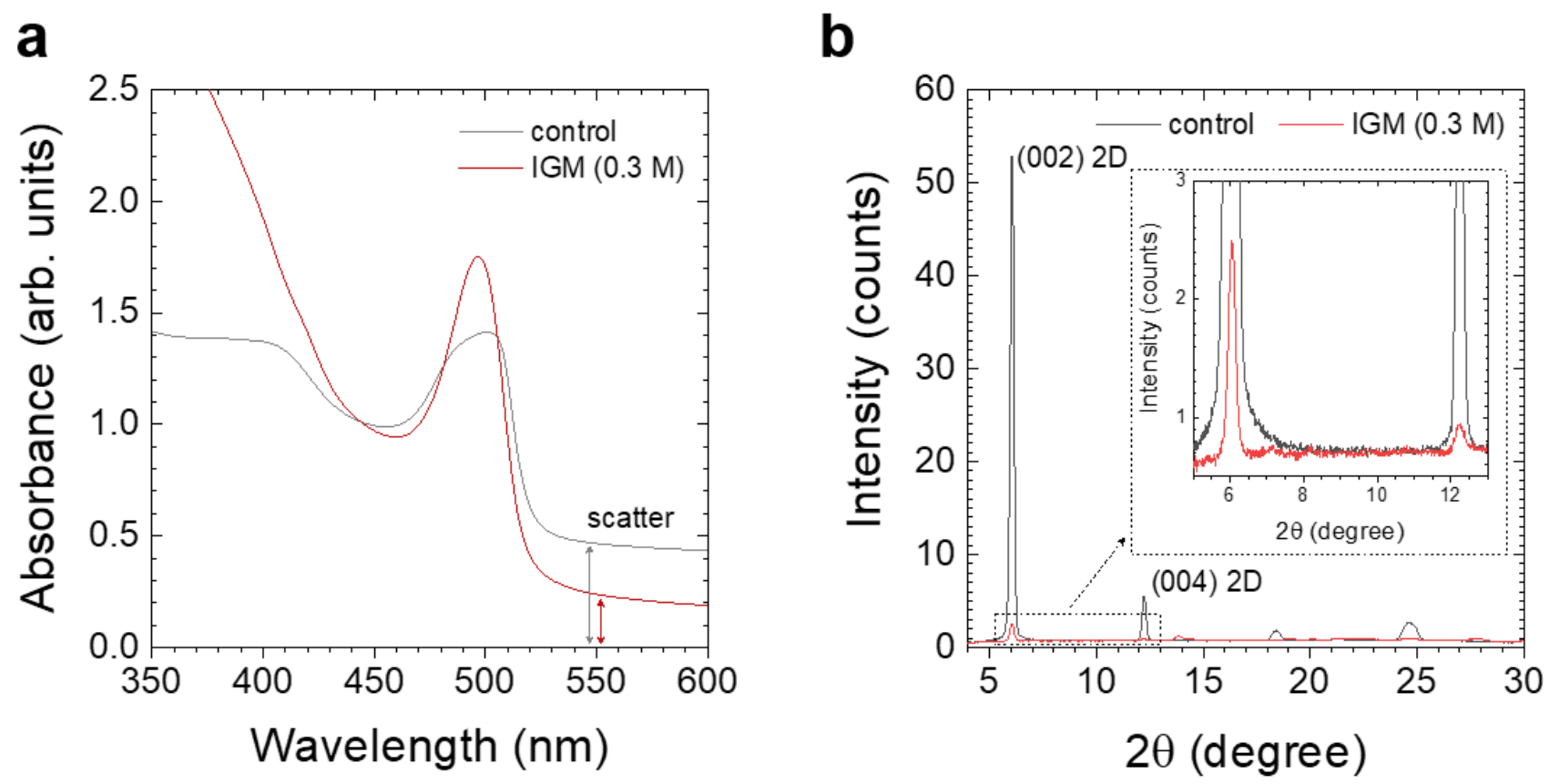

Supplementary Fig. ~~2~~ 3. a, ~~X-ray diffraction (XRD) patterns~~ absorbance spectra and **b,** ~~absorbance spectra~~ X-ray diffraction (XRD) patterns for control and IGM *S*-MBA$_2$PbI$_4$ films. The inset zooms in on the (002) and (004) peak region.

2. GIWAXS results indicate that IGM films exhibit random and isotropic crystal orientation. Their contribution to the observed anisotropic CD response is unclear. How such isotropic crystal orientation interacts or competes with their anisotropic film morphology in determining the chiral optical properties should be discussed and investigated in depth.

Ans) The reviewer raises an important point. The interaction of isotropic crystal orientation with anisotropic film morphology has been broadly investigated in other materials including chiral organic molecules, membrane proteins, and chiral semiconducting polymers[R3-6]. However, this interaction has not been fully established for chiral halide perovskites and their chiroptical properties.

E.g. Bürck et al. reported that the membrane alignment of an alpha-helical peptide can reflect different CD intensity and features. It was indicated that the material's orientation can interact differently with circularly polarized light[R4]. Wang et al. investigated circular dichroism spectra for a different chiral poly(3-(S)-2-methylbutylthiophene orientation on the film. They explained that differently-oriented polymers in the film could contribute to a dynamic change in the excitonic interaction, leading to different CD appearance with respect to the intensity and shape[R5]. Wade et al. found that the orientation of 2,2'-dicyano[6]helicene (CN6H) is dependent on the substrate type, where the chiroptical response is governed by the orientation of the CN6H molecules[R6]. According to 2D GIWAXS results, it confirmed that the CN6H molecules have a) anisotropic, b) face-on, and c) edge-on orientation on quartz, organic (3,4,9,10-perylenetetracarboxylic dianhydride (PTCDA), and inorganic copper iodide (CuI) templating substrates, respectively. These various configurations of CN6H molecules on the substrates induced different CD shapes and intensities. It indicates that the chiroptical response can be modified by controlling the orientation of target materials. Based on this literature assessment, it is likely that the generated excitons or

chromophores in the materials can lead to different interactions with circularly polarized lights depending on orientation of their crystal structures or molecules, leading to different chiroptical responses.

In our work, we expect that there are two major factors explaining the origin of the amplified CD response in IGM films: the enhanced chiral light-matter interaction via textured morphology with isotropically distributed crystals as well as electrodynamic coupling of exciton dipoles. First, these isotropic distributions of crystalline domains can substantially contribute to the enhanced CD response[R7-8]. When circularly polarized light passes the textured or patterned film, the incident light can be refracted and redirected depending on the crystal's geometry[R8]. The CD intensity is a function of the strength of the chiral light-matter interaction, and can be influenced by effective electronic dipole moments in nanocrystals[R9-10]. The transition dipoles in isotropically structured crystals can interact more efficiently with the electric dipole transition vectors of circularly polarized light owing to these refraction phenomena modeled by Hautzinger et al.[R8], leading to more intense CD response. In addition, the isotropic orientation of nanorod-like crystals also critically contributes to the enhanced light-matter interaction.

In our work, CD response for 1.0 M IGM film is dramatically amplified more than that for 0.3 M IGM film. 0.3 M IGM film consists of nanorod-like crystals growing with a predominantly parallel direction relative to the substrate, while the crystals on 1.0 M IGM film have a more isotropic structure with both perpendicular and parallel direction growth (in **Fig. 3b-c** and **e-f**). It is hypothesized that when the circularly polarized light passes through randomly oriented crystals, strong coupling between the electric field vector of the light with randomly oriented crystal domains can occur.

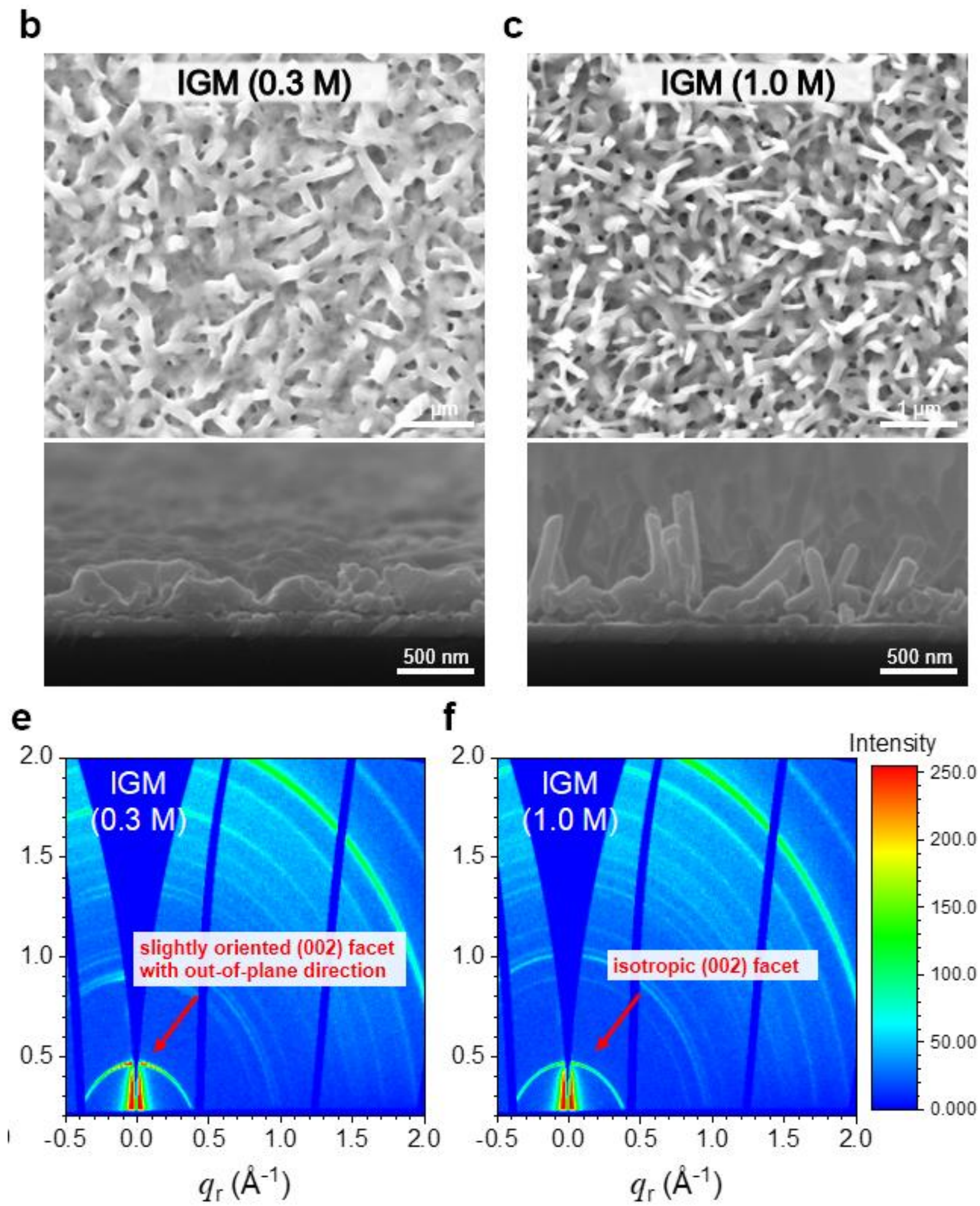

Fig 3b-c and **3e-f** in the main text

Second, rotational strength of excitons, which is proportional to the CD response[R11-12], depends on the homogeneity of crystalline domain size and crystallographic defects in the crystals. Low temperature PL results showed similar exciton fine-structure splitting energy for control and 1.0 M IGM films, indicating that the degree of electron-hole exchange interactions is identical or similar. However, the PL linewidth for the control is remarkably broader resulting in high □$_0$. This indicates that high crystalline imperfection and inhomogeneity are possibly linked to crystallographic defects. These factors can diminish electrodynamic coupling of exciton dipoles induced by the reduced constructive interference of dipoles, leading to lower rotational strength. Therefore, lower defects and crystallite domain homogeneity in IGM films can induce the enhancement of the oscillator strength, resulting in the amplified CD response.

References

[R4] Miles, A. J. & Wallace, B. A. Circular dichroism spectroscopy of membrane proteins. *Chem. Soc. Rev.* **45**, 4859-4872 (2016).

[R4] Bürck, J. *et al*. Oriented circular dichroism: a method to characterize membrane-active peptides in oriented lipid bilayers. *Acc. Chem. Res.* **49**(2), 184-192 (2016).

[R5] Wang, F. *et al*. Inversion of circular dichroism signals in chiral polythiophene films induced by end-on-oriented surface-segregated monolayers. *ACS Appl. Mater. Interfaces* **13**, 7510-7516 (2021).

[R6] Wade, J. *et al*. Controlling anisotropic properties by manipulating the orientation of chiral small molecules. *Nat. Chem.* **14**, 1373-1389 (2022).

[R7] Makhija, U. *et al*. Effect of film morphology on circular dichroism of low dimensional chiral hybrid perovskites. *J. Chem. Phys.* **160**, 021102 (2024).

[R8] Hautzinger, M. P. *et al*. Control over banded morphologies and circular dichroism in chiral halide perovskites, *ACS Nano* **19**(20), 19141-19148 (2025).

[R9] Goerlitzer, E. S. A. *et al*. The beginner's guide to chiral plasmonics: mostly harmless theory and the design of large-area substrates. *Adv. Opt. Mater.* **9**(16), 2100378 (2021).

[R10] Govorov, A. O. *et al*. Theory of circular dichroism of nanomaterials comprising chiral molecules and nanocrystals: plasmon enhancement, dipole interactions, and dielectric effects. *Nano Lett.* **10**(4), 1374-1382 (2010).

[R11] Albano, G., Pescitelli, G. & Bari, L. D. Chiroptical properties in thin films of π-conjugated systems. *Chem. Rev.* **120**(18), 10145-10243 (2020).

[R12] Ugras, T. J. *et al*. Transforming achiral semiconductors into chiral domains with exceptional circular dichroism. *Science* **387**(6733), eado7201 (2025).

To explain the possible origin of the amplified CD response, we added the following paragraphs in the revised Supplementary Information as **Supplementary Note 3**, and revised the discussion in the manuscript as follows:

[**Supplementary Note 3**]

The dimensionless oscillator strength (*f*) is an indicator to quantitatively estimate the probability of the electronic transition, which in turn determines the total absorption or emission strength. This *f*-value is determined by the transition electric dipole moment (μ), which can be expressed by the following equation for a single unit cell[6],

$$f_{12} = \frac{2m_e}{3h^2}(E_2 - E_1)\sum_{\alpha = x,y,z}|\langle 1m_1|r|2m_2\rangle|^2 = \frac{2m_e}{3h^2e^2}\Delta E_{12}|\vec{\mu}_{12}|^2$$

where, h is the reduced Planck constant, m_e is the mass of an electron. The energy and sublevel of transition states from the initial state 1 to the final state 2 are denoted as E_x and m_x, respectively. In the case of scaling up a single unit cell to multiple N unit cells (N: the number of unit cells), the individual transition electric dipole moments interfere constructively when these N unit cells are close enough to oscillate in-phase. The oscillator strength equation for the N unit cells case is given below[7-9]. This indicates that the oscillator strength dramatically increases if there are strong constructive interferences owing to the electrodynamic coupling of the transition dipoles.

$$f_{total} = \frac{2m_e}{3h^2e^2}\Delta E_{12}\left|\sum_{i=1}^{N}\vec{\mu}_{12}\right|^2 \propto \left(N \times \vec{\mu}_{single}\right)^2$$

With similar concepts based on the quantum-mechanical theory, the rotational strength (R) can be expressed by the following equation below. However, unlike the oscillator strength, the transition electric dipole moment (μ) and magnetic dipole moment (m) are involved in the expression of R[10-13].

$$R = Im\{\vec{\mu} \cdot \vec{m}\}$$

According to these theoretical models for multiple unit cells, the strong long-range electrodynamic coupling of dipoles induced by the constructive interference magnifies rotational strength, leading to the amplification of the CD response. To induce the electrodynamic coupling of dipoles, coherence of crystallite domains is critical[14,15]. The coherent crystallite domains, with uniform orientation and few crystallographic defects, provide a structurally in-phase environment of dipoles with their neighbors, indicating that this structural perfection allows the excitonic transition dipoles to couple coherently. Furthermore, high crystallinity and less defects facilitate the prolonged carrier/exciton diffusion lengths, resulting in the enhancement of the electronic coupling[16,17].

References for the revised Supplementary Information

6. Hilbom, R. C. Einstein coefficients, cross sections *f* values, dipole moments, and all that. *Am. J. Phys.* **50**, 982-986 (1982).

7. Franta, D., Nečas, D. & Zajíčková, L. Application of Thomas-Reiche-Kuhn sum rule to construction of advanced dispersion models. *Thin Solid Films* **534**, 432-441 (2013).

8. Lestrange, P. J., Egidi F. & Li, X. The consequences of improperly describing oscillator strengths beyond the electric dipole approximation. *J. Chem. Phys.* **143**, 234103 (2015).

9. Zheng, L., Polizzi, N. F., Dave, A. R., Migliore, A. & Beratan, D. N. Where is the electronic oscillator strength? Mapping oscillator strength across molecular absorption spectra. *J. Phys. Chem. A.* **120**(11), 1933-1943 (2016).

10. Tinoco, I. & Woody, R. W. Optical rotation of oriented helices. IV. A free electron on a helix. *J. Chem. Phys.* **40**, 160-165 (1964).

11. Caricato, M. Orbital analysis of molecular optical activity based on configuration rotatory strength. *J. Chem. Theory Comput.* **11**(4), 1349-1353.

12. Berova, N., Bari, L. D. & Pescitelli, G. Application of electronic circular dichroism in configurational and conformational analysis of organic compounds. *Chem. Soc. Rev.* **36**, 914-931 (2007).

13. Albano, G., Pescitelli, G. & Bari, L. D. Chiroptical properties in thin films of π-conjugated systems. *Chem. Rev.* **120**(18), 10145-10243 (2020).

14. Zhang Y. *et al.* Visualizing coherent intermolecular dipole-dipole coupling in real space. *Nature* **531**, 623-627 (2016).

15. Muller, E. A., Gray, T. P., Zhou, Z. & Raschke, M. B. Vibrational exciton nanoimaging of phases and domains in porphyrin nanocrystals. *PNAS* **117**(13), 7030-7037 (2020).

16. Pandya, R. et al. Femtosecond transient absorption microscopy of singlet exciton motion in side-chain engineered perylene-diimide thin films. J. Phys. Chem. A. 124(13), 2721-2730 (2020).

17. Kranz, J. J. & Elstner, M. Simulation of singlet exciton diffusion in bulk organic materials. J. Chem. Theory Comput. 12(9), 4209-4221 (2016).

On page 10 in the revised manuscript,

"Next, we take a closer look at the structural features of the chiral 2D perovskite films for both control and IGM conditions. At an *S*-MBAI concentration between 0.5 - 1.0 M, anisotropic growth was found, resulting in rod-like nanostructures with a width of tens of nm to 100 nm (**Fig. 3a-c** and **Supplementary Fig. ~~8-9~~-10-11**). If the concentration is too low, a typical polycrystalline structure is observed with submicrometer grain size; if the concentration is too high (exceeding 1.5 M), the rod-like nanostructures continue growing laterally into micrometer-sized platelets with incomplete substrate coverage. The nanorod morphology, which is similar shape as its single crystals[32-34], suggests a higher structural order and better crystallinity. In contrast, the control film exhibits crystals with rounded edges and agglomerated particles, as seen in the cross-sectional SEM image (**Fig. 3a**), indicating there might be substantial crystallographic defects and inhomogeneity. To further evaluate the surface topography of control and IGM (0.3 M and 1.0 M)

films, we performed atomic force microscopy (AFM) measurements (see **Supplementary Fig. 12-13**, and **Supplementary Note 1**). These AFM results indicate poor coverage of control film, which is in line with the morphological results (**Fig. 1b** and **Supplementary Fig. 3a**).
The 2D GIWAXS patterns of the control and IGM samples reveal distinct diffraction intensity distributions over the azimuthal angle (□) of the chiral 2D perovskite phase (**Fig. 3d-f**)."

On page 14 in the revised manuscript,

"~~The strength of rotatory transitions and exciton formation[34] can be enhanced by the spatial proximity of crystallite domains, as observed in the IGM films. Smaller crystallites tend to preserve a single-domain structure, as opposed to large crystallites with multiple domains with different lattice orientations, higher defect density, and local distortions. Moreover, the smaller crystallites allows for more effective overlap of excitonic wavefunctions, which results in higher oscillator strength. Chiroptical transitions can benefit from the same effects and also from higher phase coherence between electric and magnetic transition dipole moments,~~ We hypothesize that there are two major factors contributing to the amplified CD response in IGM films: the enhanced light-matter interaction via textured morphology with randomly oriented crystals as well as enhanced electrodynamic coupling of transition dipoles. First, these isotropic distributions of crystalline domains can substantially contribute to the enhanced CD response. When circularly polarized light passes the textured or patterned film, the incident light can be refracted depending on the crystal's geometry[22]. When the circularly polarized light passes through randomly oriented crystals, strong and highly efficient light-matter interactions occur by coupling the electric field vector of the light with a more diverse population of crystalline domains considering all possible interaction geometries[22,42]. Therefore isotropic orientation of nanorods leads to more intense CD response. Second, the interaction strength of electrodynamic coupling of transition dipoles (excitons) can be enhanced by higher structural order and homogeneity of crystallite domains, as observed in the IGM films. Lower defects and homogeneous crystallite domains with smaller crystal sizes in IGM films can induce the enhancement of the rotational strength, resulting in an improved dissymmetry factor g_{CD} (**Supplementary Note 3**). The correlation between the crystallite homogeneity and the amplified CD response is further discussed in the next section based on temperature-dependent PL linewidth analysis.

"

References for the revised manuscript

32. Liu, Z. *et al.* Chiral Hybrid Perovskite Single-Crystal Nanowire Arrays for High-Performance Circularly Polarized Light Detection. *Adv. Sci.* **8**(21), 2102062 (2021).

33. Pan, R. *et al.* Tunable and Large Magneto-Photoluminescence for Single-Crystalline Chiral Perovskites. *Adv. Optical Mater.* **10**(15), 2200064 (2022).

34. Wang, Z. *et al.* Chiroptical Response Inversion and Enhancement of Room-Temperature Exciton-Polaritons Using 2D Chirality in Perovskites. *Adv. Mater.* **35**(42), 2303203 (2023).

~~34.~~42. ~~David, A. & Miller, B. Optical physics of quantum wells. in *Quantum Dynamics of Simple Systems* (CRC Press, 1997).~~ Pols, M., Brocks, G., Calero, S. & Tao, S. Temperature-dependent chirality in halide perovskites. *J. Phys. Chem. Lett.* **15**(31), 8057-8064 (2024).

3. The authors discussed the possible LDLB effect of the CD response in chiral perovskites. However, previous relevant studies were not cited properly, e.g. JACS 2022 (Lu); Chem. Rev. 2020 (Di Bari). Please discuss more on the fundamental origin of the CD amplification. Was it due to the anisotropic structure, enhanced light-matter interaction, or there are fundamental differences in their electronic structures, such as an increased spin-splitting? That is, is it because of a morphological effect or electronic effect?

Ans) We agree with the reviewer, LDLB is important to consider. We revised the paragraph regarding LDLB effects in the revised manuscript. Please see below. We also cited the reference pointed out by the reviewer.

The second part about the discussion on the origin of the CD amplification in our work is similar to Q2. Please see the answer of Q2.

On page 13-14 in the revised manuscript,

"To quantitatively assess the influence of crystallization on chiroptical activity, we calculate the dissymmetry factor from CD spectra using g_{CD} = CD/(32980 × A) equation, where A is the absorbance corresponding to CD spectra~~[33]~~. ~~We~~ CD spectra were measured ~~the thin film samples~~ from both the front and back sides to account for interference of macroscopic anisotropies; *i.e.*, linear dichroism ~~(LD)~~ and linear birefringence (LDLB) effects[39-41]. The LDLB contributions are ~~small~~ negligible (**Fig. 4d**) and g_{CD} is corrected for any LDLB contributions ~~(also see **Supplementary Fig. 12-13 15-16**~~)."

References for the revised manuscript

39. Albano, G., Pescitelli, G. & Di Bari, L. Chiroptical Properties in Thin Films of π-Conjugated Systems. *Chem. Rev.* **120**, 10145–10243 (2020).

40. Han, X.-B., Wang, W., Jin, M.-L., Jing, C.-Q., Liang, B.-D., Chai, C.-Y., Xiong, R.-G., and Zhang W., Unveiling Chiral Perovskite CD Signal Scaling: Discerning Authentic and Counterfeit Signals through Sample-State Analysis. *Anal. Chem.* **95**(44), 16201-16209 (2023).

41. Zhang, Z., Wang, Z., Sung, H. H.-Y., Williams, I. D., Yu, Z.-G., and Lu, H., Revealing the intrinsic chiroptical activity in chiral metal-halide semiconductors, J. Am. Chem. Soc. 144, 22242-22250 (2022).

4. Authors had investigated the effect of ionic gel concentration on the crystallization behavior and chiroptical property of chiral 2D perovskites. However, several important factors that can also significantly influence crystallization were not included in the study. For example, does the gel droplet volume at a fixed concentration, or the thickness of PbI2 layer still affect perovskite growth and the resulting CD response? In addition, how do the results differ between directly dropping an MBA-IPA solution and dropping a pre-formed gel obtained by evaporating IPA? More detailed and systematic experiments should be designed and conducted to fully elucidate the crystallization mechanism and its relationship to chiroptical response.

Ans) We appreciate the valuable comments and questions raised by the reviewer. We agree with the reviewer that there are several synthesis parameters such as droplet volume, solution concentration, and thickness of PbI_2 layer, which might influence the crystallization of chiral halide perovskite films as well as their chiroptical properties.

Effect of <u>volume of MBAI-IPA precursor solution</u>: In this study, we demonstrated that the concentration effect of the *S*-MBAI solution dropped on the PbI_2 layer and PbI_2 layer thickness effect. We think that its effect regarding the droplet volume of *S*-MBAI solution on the underlying PbI_2 layer is mechanistically analogous to the concentration effect of *S*-MBAI solution discussed in our manuscript. The most important factor governing the crystallization of overall halide perovskite films via two-step deposition methods is the amount of the treated organic or inorganic salts (*S*-MBAI) in the dropped solution available to diffuse into and react with the underlying PbI_2 layer. So, we expected that increasing the droplet volume of the *S*-MBAI solution would increase the supply of *S*-MBAI salts into the PbI_2 layer, probably leading to similar results observed when increasing the concentration of the *S*-MBAI solution (please see **Supplementary Fig. 19-20** in the revised Supplementary Information, corresponding to the resulting CD response for *S*-MBA_2PbI_4 films formed by using varying concentrations of *S*-MBAI solutions). In the case of insufficient supply of *S*-MBAI (0.1 M IGM), we will discuss it in detail later at Q8 for reviewer 1.

Effect of <u>PbI_2 layer thickness</u>: To further discuss the effect of PbI_2 layer thickness on *S*-MBA_2PbI_4 film formation and their chiroptical responses, we modulated the PbI_2 film thickness by varying

the concentration of the PbI_2 coating solution (see **Fig. R3**). The corresponding diffraction patterns of thickness-dependent PbI_2 films are demonstrated in **Fig. R4a-b**. In addition, we prepared IGM samples using 0.3 M and 1.0 M *S*-MBAI solutions treated on varying-thickness PbI_2 films, and then measured synchrotron-based GIWAXS by varying the X-ray incidence angles from 0.1° to 2.0° and CD to investigate PbI_2 layer thickness effect on the film formation and their chiroptical properties (**Fig. R4c-h** and **R5**). The representative diffraction peaks corresponding to 2D *S*-MBA_2PbI_4, 1D *S*-$MBAPbI_3$, and PbI_2 phases are observed at q = 0.46, 0.61, and 0.92 $Å^{-1}$, respectively. The absence of residual 1D and PbI_2 phases in films for 0.3 M and 1.0 M IGM conditions with 0.5 M PbI_2 layers is indicative of the sufficient supply of *S*-MBAI salts into the PbI_2 layer. However, as increasing the PbI_2 film thickness (from 0.5 M to 1.5 M PbI_2) for both 0.3 M and 1.0 M IGM conditions, the unreacted 1D and PbI_2 phases become more pronounced gradually from the surface to the bottom perovskite/substrate interfaces. We already exhibited these results in **Fig. 2d-e** and **Supplementary Fig. 9**. According to this basis of our finding, the crystallization mechanism via IGM methods can be expected as following several steps during film formation; i)When treating *S*-MBAI solution (*S*-MBAI salts dissolved in isopropanol) onto the PbI2 layer, the treated *S*-MBAI solution partially dissolves the underlying PbI_2 layer, starting from its surface. The extent of this solubility depends on the concentration of *S*-MBAI solution; a higher concentration leads to higher solubility of PbI_2 (**Supplementary Fig. 5**). ii) During the spin-coating process, the IPA solvent is partially evaporated, promoting the formation of both gel-like amorphous intermediate (consisting of a complex of PbI_2, *S*-MBAI, and residual IPA) and ionic gel (consisting of a complex of *S*-MBAI and residual IPA) phases on the surface. For instance, in case of the 0.3 M IGM condition, we observed that the PbI_2 layer exists undissolved upon the initial treatment of the *S*-MBAI solution, although there are no unreacted PbI_2 and 1D phases at the final film after annealing. In the case of a low *S*-MBAI solution concentration, ionic gel phase consisting of unreacted *S*-MBAI and residual IPA predominantly remains on the as-coated film surface. On the other hand, for 1.0 M IGM condition, the treated *S*-MBAI solution can mostly dissolve the underlying PbI_2 layer, resulting in predominantly forming the intermediate phase. iii) during the annealing process, there are two crystallization processes. The first is that the intermediate phase is directly converted to 2D phase. The second is that *S*-MBAI ion species in ionic gel phase gradually diffuse into the underlying PbI_2 layer, where the elevated temperature triggers a phase transformation into the 1D (if *S*-MBAI species is insufficient) and/or 2D (if *S*-MBAI species is sufficient) phase based on a diffusion-driven phase conversion mechanism, as can be seen in **Fig. 2e**.

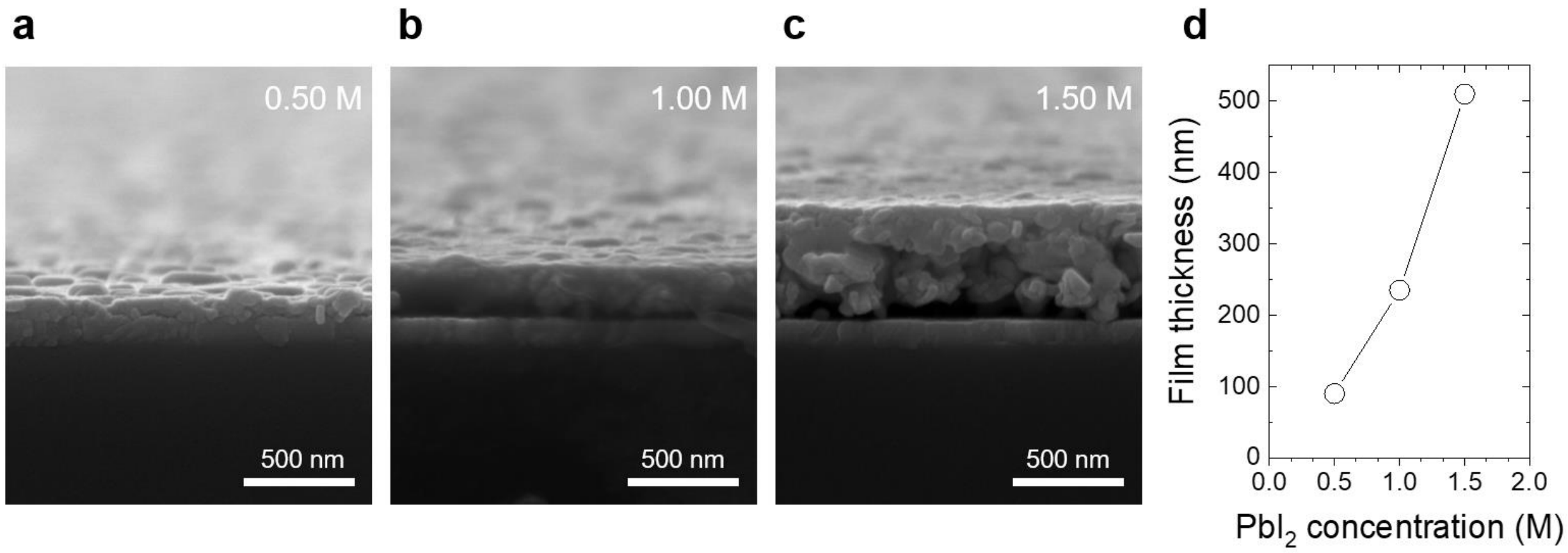

Fig. R3. a-c, Cross-sectional SEM images of PbI_2 films. PbI_2 thickness are controlled by adjusting the precursor solution concentration with (a) 0.5 M, (b) 1.0 M, and (c) 1.5 M. **d,** PbI_2 film thickness profile as a function of PbI_2 solution concentration.

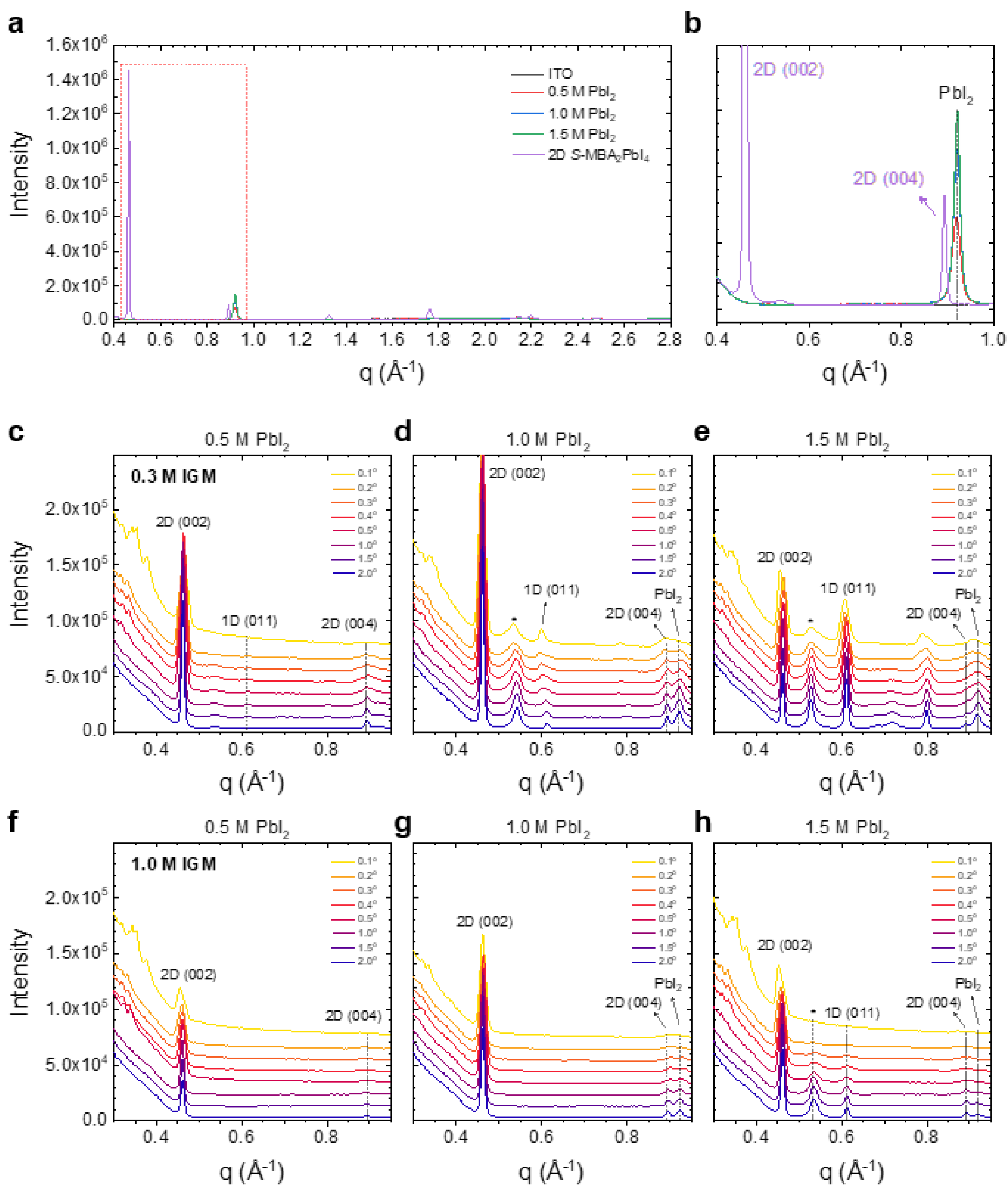

Fig. R4. **a-b**, Diffraction patterns for ITO (bare substrate), PbI_2 films with varying thickness conditions, and 2D *S*-MBA2PbI4 film. The diffraction patterns were obtained from synchrotron-based GIWAXS frames. **c-h**, Depth-dependent diffraction patterns obtained from GIWAXS at X-ray grazing incidence angles from 0.1° to 2.0° for (**c-e**) 0.3 M and (**f-h**) 1.0 M IGM films as different PbI2 film thickness.

Fig. R5 shows the resulting chiroptical responses for 0.3 M and 1.0 M IGM samples with varying PbI_2 film thickness. Considering CD response and corresponding g_{CD} for 0.3 M IGM film at around 500 nm wavelength region (excitonic absorption), no significant difference depending on PbI_2 layer thickness is observed. However, for 1.0 M IGM film, CD intensity and g_{CD} at ~500 nm decrease significantly with increasing PbI_2 film thickness.

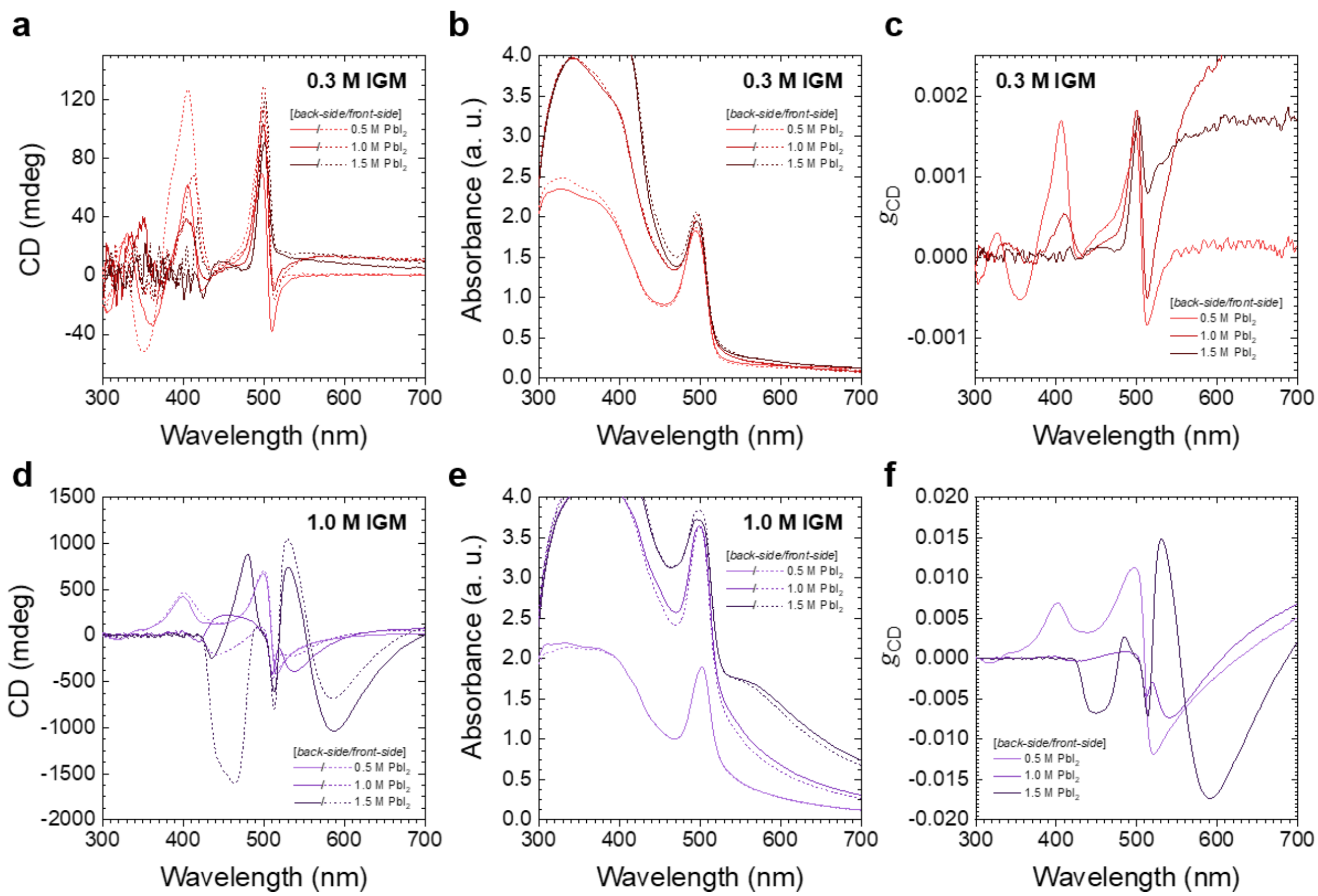

Fig. R5. (**a,d**) CD, (**b,e**) absorbance, and (**c,f**) g_{CD} spectra for (**a-c**) 0.3 M and (**d-f**) 1.0 M IGM films as different PbI_2 film thickness.

Film formation from <u>pre-formed gel</u>: We also prepared IGM films using pre-formed gel, shown in **Fig R6**. To make a pre-formed gel, 1.0 M *S*-MBAI (dissolved in IPA) solution was stored in a vacuum oven at 70 °C for 12 hours to evaporate the IPA solvent. Because of high viscosity of the pre-formed gel, it was not spread out evenly into the PbI_2 film during dynamic spin-coating, resulting in inhomogeneous film formation. In XRD patterns, unreacted *S*-MBAI and PbI_2 phases are observed. As a result, the film exhibits abnormal CD features, high LDLB effect, and relatively low g_{CD}. Obviously, their reproducibility will be low.

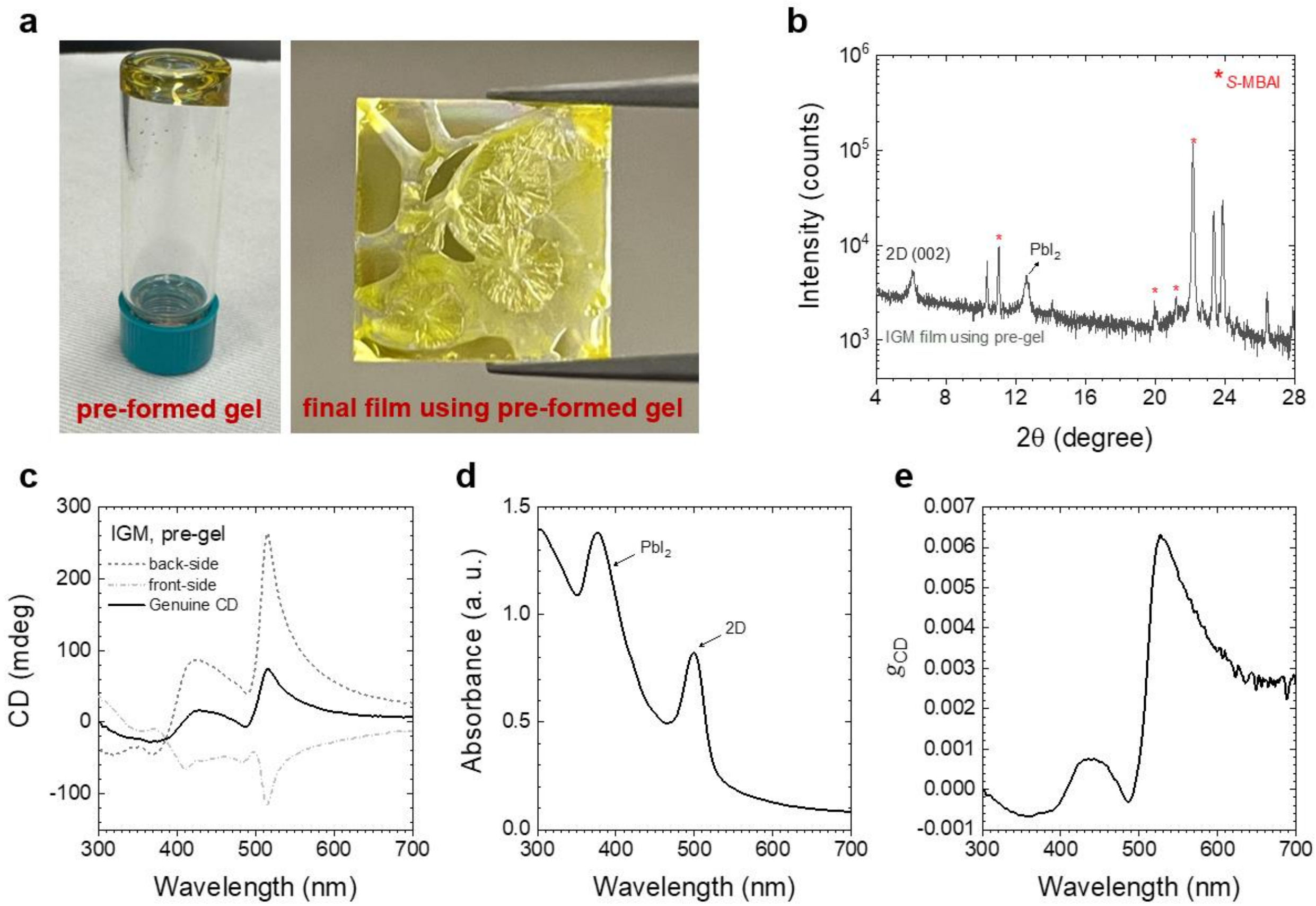

Fig. R6. **a**, Photographs of pre-formed gel (left) and annealed film using pre-formed gel (right). **b-e**, (b) XRD pattern, (c) CD, (d) absorbance, and (e) g_{CD} spectra for the film using pre-formed gel.

5. IGM chiral 2D perovskites showed the large gcd values, which form the basis for circularly polarized emission. Authors are therefore strongly encouraged to include circularly polarized luminescence measurements in the manuscript to further highlight the potential of IGM approach.

Ans) Following the reviewer's comment, we carried out circularly polarized photoluminescence (CPPL) at room temperature for control and IGM (0.3 M and 1.0 M) films, as can be seen in **Fig R7** below. For all conditions, no differences in intensity between the left- (σ-) and right-circularly polarized (σ+) photoluminescence were observed.

The origin of negligible CPPL response measured at room temperature for chiral halide perovskite materials is probably related to the spin relaxation of charge carriers at the excited states. The emission of CPPL is related to the spin alignment of electrons and holes when they are excited from the ground to excited state. Chiral halide perovskites, as part of the broader class of hybrid metal halide perovskites, are physically soft materials leading to considerable thermally activated motion. It is well known that there is internal motion in these materials incl. molecular rotation-vibration in the cuboctahedral cavity; (i) drift and diffusion of large polarons; (ii) transport of

charged ionic defects[R13]. These motions can lead to spin relaxation processes in the excited state[R13-14], leading to a negligible CPPL response at room temperature.

While CD measurements can reveal the inherent chirality of the material's structure in the ground state (ground state geometry), CPPL measures the differential emission of left- and right-circularly polarized photons from the excited state (excited state geometry). The interplay between CD and CPPL depends on its structural rigidity upon photoexcitation. If the material's ground and excited state geometries are similar, the CD and CPPL responses would be comparable. In contrast, if its structures or charge carriers in the excited state have enough time to undergo relaxation, the resulting CPPL appearance can differ from the ground state CD in sign and intensity[R15]. Therefore, even though there are correlations between CD and CPPL, the strong CD responses do not always guarantee CPPL signal[R16].

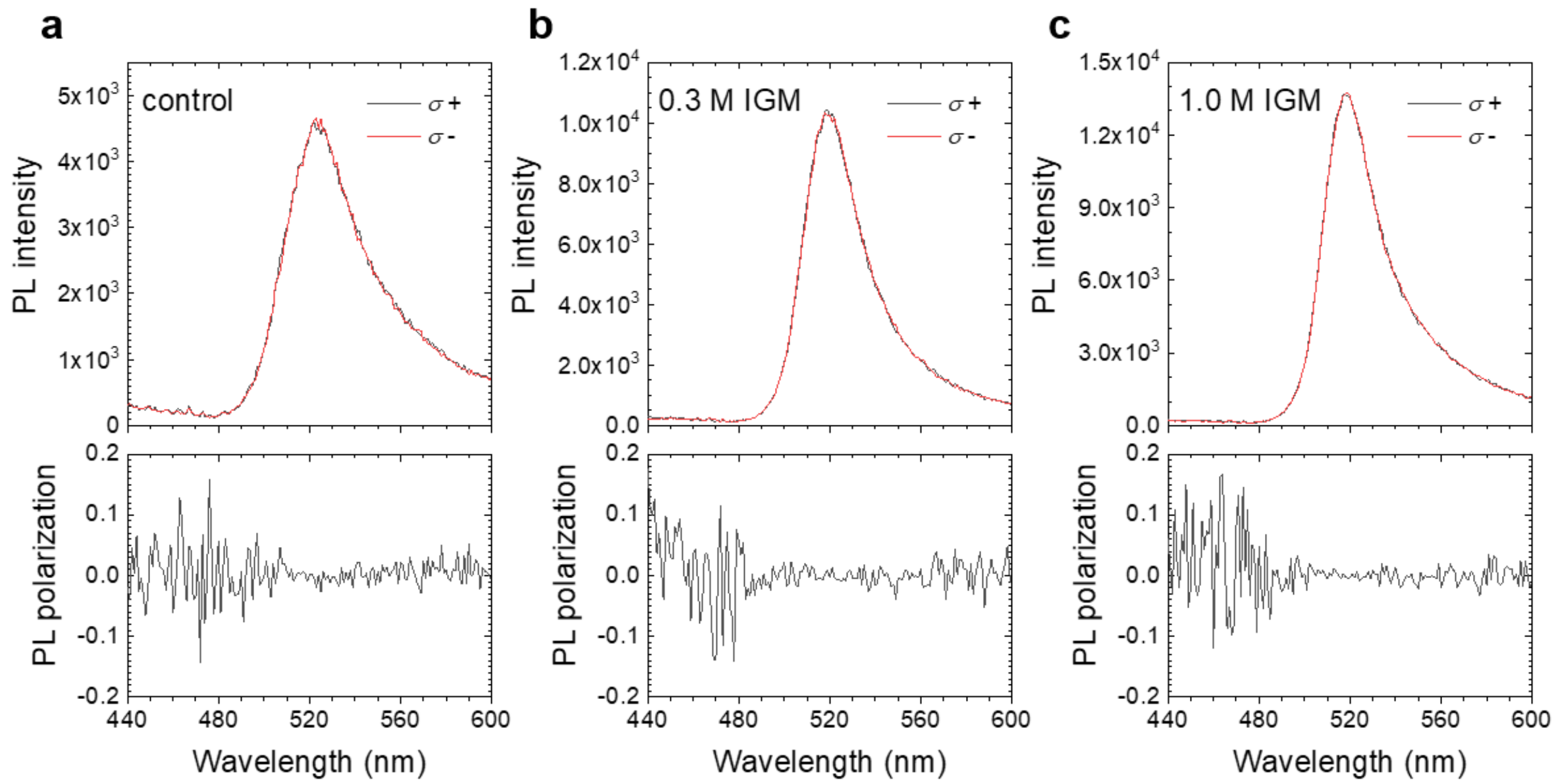

Fig. R7. **a-c**, Circularly polarized PL (top) and PL polarization (bottom) spectra for control and IGM (0.3 M and 1.0 M) films at room temperature. PL polarization was quantified by the difference between left- and right-circularly polarized PL intensities.

References

[R13] Frost, J. M. & Walsh, A. What is moving in hybrid halide perovskite solar cells? *Acc. Chem. Res.* **49**(3), 528-535 (2016).

[R14] Ma, J. *et al.* Chiral 2D perovskites with a high degree of circularly polarized photoluminescence. *ACS Nano* **13**(3), 3659-3665 (2019).

[R15] Pecul, M. & Ruud, K. The optical activity of β,γ-enones in ground and excited states using circular dichroism and circularly polarized luminescence. *Phys. Chem. Chem. Phys.* **13**, 643-650 (2011).

[R16] Liu, S. *et al.* Bright circularly polarized photoluminescence in chiral layered hybrid lead-halide perovskites. *Sci. Adv.* **9**, eadh5083 (2023).

6. In Supplementary Fig. 3c, does the gel formed after the IPA evaporation solidify during the cooling process or under the ice condition? If solidification occurs, XRD patterns of these solids could further help identify the proposed 1D intermediates.

Ans) We thank the reviewer for the careful reading and thoughtful questions. We designed several experiments to tackle the reviewers' point.

The pre-formed gel, shown in **Supplementary Fig. 4c** does not solidify during cooling. However, we found that the crystallization of this gel phase is triggered by exposure to the ambient air. An oily surface was observed for as-annealed films under the inert N_2 condition, indicating that the ionic gel phase still exists at the film surface without crystallizing. To further clarify the crystallization of the ionic gel phase, we prepared 2.0 M *S*-MBAI solution, and then spin-coated it on the ITO substrate under N_2 condition, shown in **Fig. R8**. Afterward, the as-coated film was annealed at 70 °C for 10 min under the inert condition, leading to the formation of an amorphous ionic gel phase (**Fig. R8a**). When this ionic gel film is exposed to the ambient air, the crystallization of excess S-MBAI occurs on the film surface within a few minutes (**Fig. R8b**). As can be seen in **Fig R8c**, there are two distinguishable phases: amorphous and crystalline. XRD patterns (**Fig. R8d**) show these films (fresh and aged films for 2.0 M IGM condition, S-MBAI film), where the peaks at 11.0° and 22.1° are indicative of S-MBAI crystalline phase. When the film is exposed to the ambient air (RH ~ 60%) for 30 min, the crystalline phase of S-MBAI on the surface is observed (photographs in **Fig. R8a-b**).

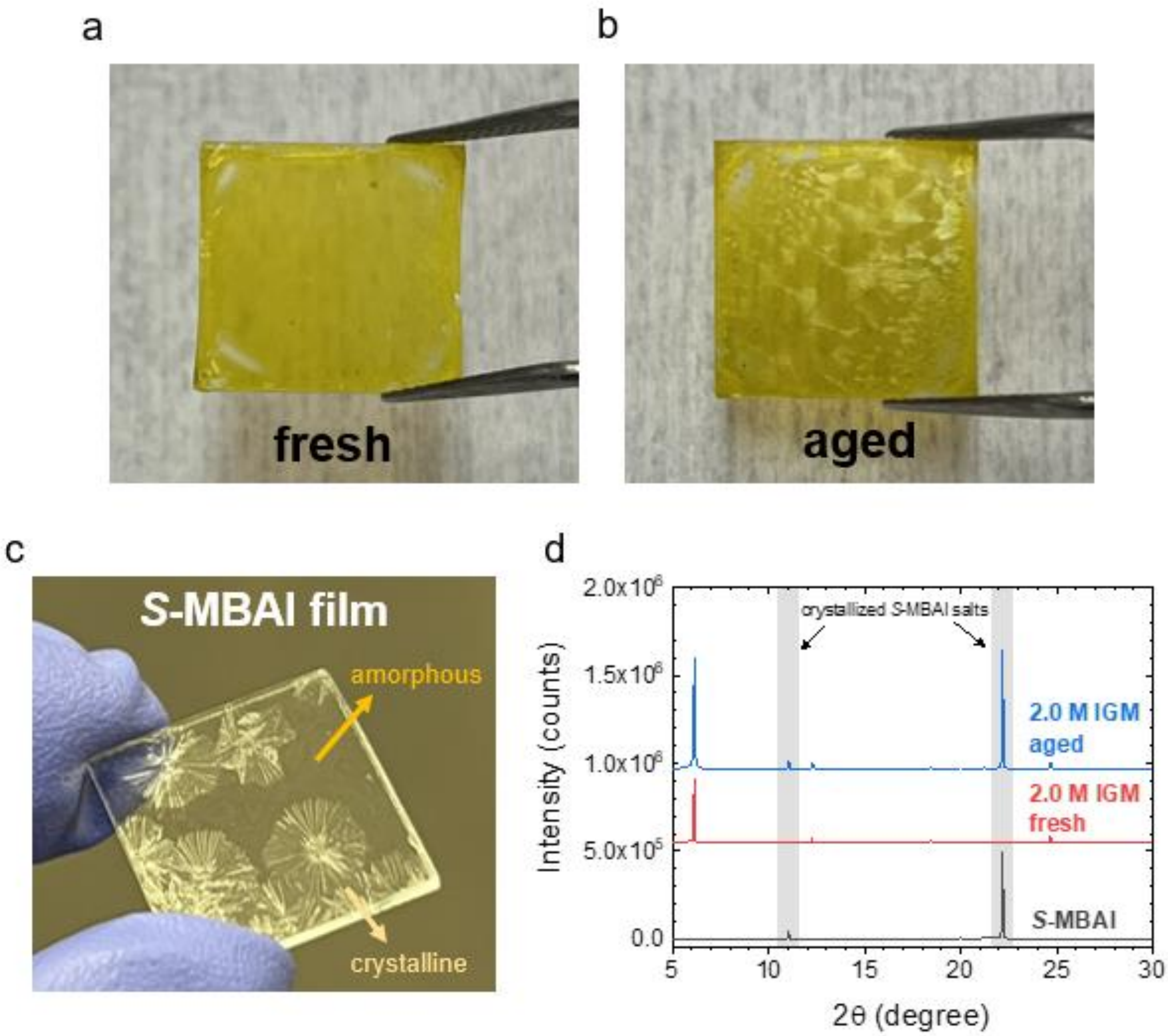

Fig. R8. **a,b**, Photographs of (**a**) fresh and (**b**) aged 2.0 M IGM film. The film was aged for 30 min in the ambient air. **c**, Photograph of S-MBAI film. d, XRD patterns for S-MBAI, fresh and aged 2.0 M IGM films. The black pattern corresponds to diffraction peaks for S-MBAI crystalline phase.

To identify a gel intermediate of oligomeric $[PbI_3]^-$ plumbate ion species, we performed in-situ UV-Vis measurements (shown in **Fig. 2c-d** and **Supplementary Fig. S8**) and compared SEM images (**Supplementary Fig. S10-11**), since these 1D face-sharing oligomers promote anisotropic rod-like crystal growth in IGM films. However, such analyses of colloidal chemistry and morphology are typically reserved for hybrid perovskite synthesized from mixed organic and inorganic precursors, not for systems derived from a single precursor. As suggested by the reviewer, we added more details and references in the main text to clarify the formation of the proposed 1D oligomeric species and its influence on the crystal formation. Please see below.

On page 7-8 in the revised manuscript,

"*In-situ* UV-Vis transmission measurements during spin coating (**Fig. 2c** and **Supplementary Fig.** ~~**6**~~ **8**) show that the original PbI_2-related absorption features diminish and a new absorption peak

around 368 nm appears a few seconds after the MBAI is deposited. It is well-known that PbI_2 undergoes complexation in the presence of excess iodide ions to form higher-coordinated plumbate species[26]. Consequently, during IGM crystallization, PbI_2 undergoes complexation to form soluble PbI_3^- with an absorption feature at 368 nm[27]. The PbI_3^- likely forms a gel intermediate of oligomeric [PbI_3^--MBA^+] species through coulombic and hydrogen bonding interactions: $[PbI_3]_n^-{}_{(sol)} + n\ MBA^+{}_{(sol)}$. During the subsequent spin coating process, IPA solvent evaporates. We hypothesize that templating of the chiral 2D material is initiated from a 1D gel framework of $[PbI_3^- -MBA^+]_n$ species, promoting anisotropic rod-like growth. The crystal growth is governed by the geometry of colloidal species and crystal structures[23,28]. The $[PbI_3]^-$ species have an one-dimensional polymeric face-sharing octahedral formed by interacting organic cations with their (001) edge[23,29,30]. It is inferred that these structures of oligomeric [PbI_3^- -MBA^+] species can trigger 1D-structured crystal growth. ~~$[PbI_3]^-$ species are prone to forming 1D, face-sharing oligomers[23].~~ Furthermore, a strong intermolecular interaction of organic components can modulate the directional crystal growth kinetics differently along longer and shorter axes, promoting needle-like shape formation[28]. Turning to the experimental results, ~~A~~at low *S*-MBAI concentrations, the conversion of PbI_2 is limited to the surface and proceeds *via* the gel intermediate and MBAI intercalation. ~~This results in horizontally aligned rods templated by the underlying oriented PbI_2.~~ In contrast, ~~A~~at higher *S*-MBAI concentrations (*e.g.*, 1.0 M), the ~~ionic gel~~ IG phase can fully dissolve the underlying PbI_2. This is followed by the fast growth of randomly oriented rods, similar to single crystal growth in solutions. By controlling the *S*-MBAI concentration, one can modulate the IGM growth and sample morphology. The formation kinetics are significantly slower and more gradual in the control compared to the IGM samples, as extracted from the slopes of the 2D peak intensities in the bottom panel of **Fig. 2b**. The rapid crystallization in the IGM process is indicative of a fast nucleation process with many nucleation sites, while in the control sample longer growth time likely leads to Ostwald ripening and consequently to the large-grained films discussed in **Fig. 1b**."

References for the revised manuscript

28. Zhang, M., Jin, L., Zhang, T., Jiang, X., Li, M., Guan, Y. & Fu, Y. Two-dimensional organic-inorganic hybrid perovskite quantum-well nanowires enabled by directional noncovalent intermolecular interactions. *Nat. Commun.* **16**, 2997 (2025).

29. Zhang, L., Zheng, W., Li, S., Han, J., Jiang, W. & Jia D. Iodoplumbates from 1D chain to 2D layer: Syntheses, crystal structures, and photocatalytic properties of organic hybrid lead iodides with diammonium structural templating. *Inorg. Chim. Acta.* **484**, 104-110 (2019).

30. Tremblay, M.-H. *et al.* Hybrid organic lead iodides: Role of organic cation structure in obtaining 1D chains of face-sharing octahedra vs 2D perovskites. *Chem. Mater.* **34**(3), 935-946 (2022).

7. Chiral 2D perovskites are promising semiconductors for electronic applications. However, the IGM films appear to contain numerous pinholes. AFM measurements should be performed to further evaluate the surface roughness of films. Also, study of spin-dependent charge transports would be necessary to substantiate their potential for practical spintronic applications.

Ans) We thank the reviewer for the constructive feedback. To evaluate the surface topography of control and IGM (0.3 M and 1.0 M) films, we performed atomic force microscopy (AFM) measurements (see **Fig. R9**). The average surface roughness (R_a) and root mean square surface roughness (R_q) are quantitatively estimated on a 10 × 10 □m² scan area. The control film exhibits rough and poor coverage with R_a of 100.1 nm and R_q of 133.3 nm. In contrast, a lower surface roughness is observed for IGM samples (R_a of 26.88 nm and R_q of 33.44 nm for 0.3 M IGM, and R_a of 85.20 nm and R_q of 105.0 nm for 1.0 M IGM). Comparing different IGM concentrations, it was observed that the film roughness increases as the concentration of the *S*-MBAI solution increases, a trend consistent with the cross-sectional SEM results (**Supplementary Fig. 11** in the revised Supplementary Information). This increased roughness for IGM films made with higher concentration of *S*-MBAI solution can be attributed to the enhanced vertical growth of nanorod-like crystals.

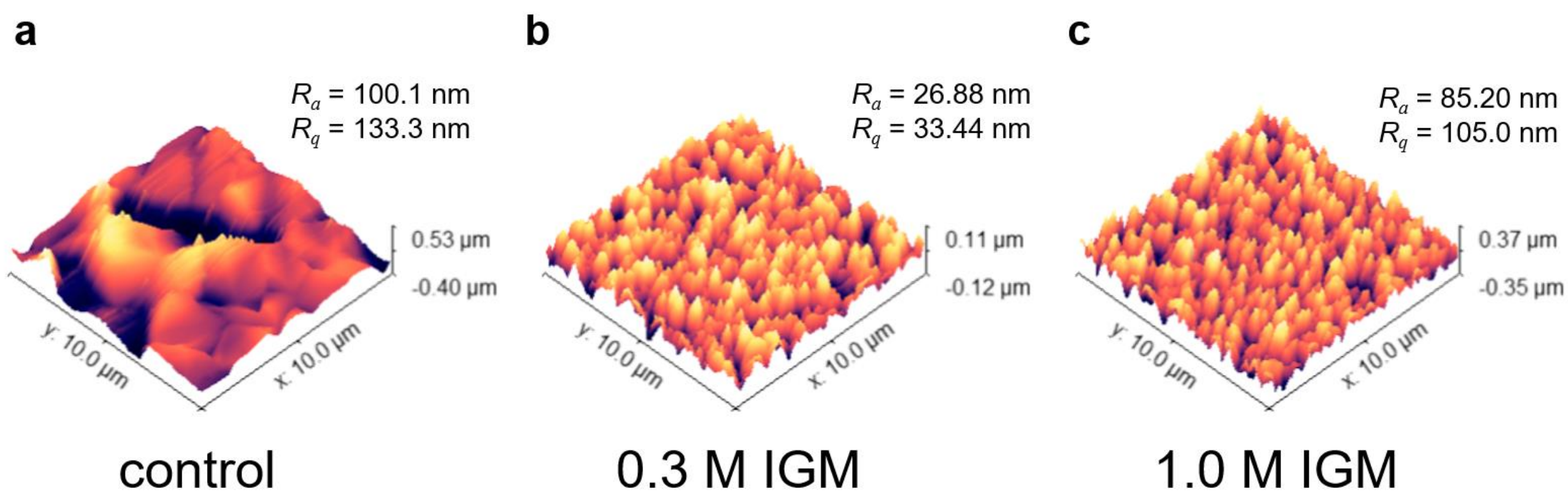

Fig. R9. **a-c**, 3D view of AFM images for (**a**) control and IGM films ((**b**) 0.3 M and (**c**) 1.0 M), where R_a is average surface roughness and R_q is Root-Mean-Square surface roughness.

To further characterize the uniformity of those films, we analyzed AFM line profiles with 4 lines on the 2D AFM images (**Fig. R10**). The control sample shows about one order of magnitude higher height fluctuations compared to the 0.3 M sample and slightly higher fluctuations compared to the 1.0 M sample. The line scans of the control sample seem to be dominated by large crystals with poor coverage while the IGM films show a more homogeneous distribution with smaller crystals.

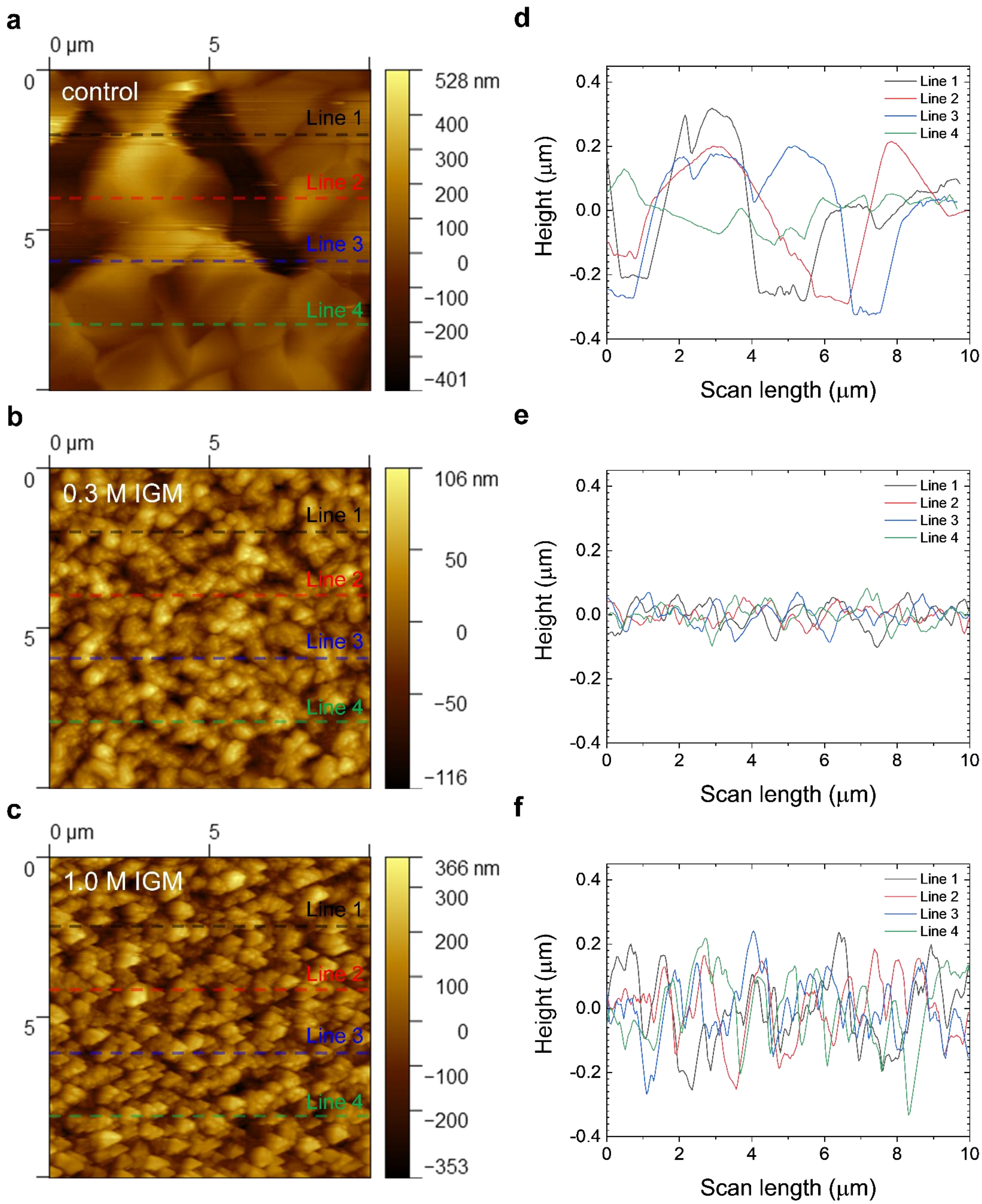

Fig. R10. (**a-c**) 2D AFM images and (**d-f**) line profiles with 4 lines.

As the reviewer mentioned, producing uniform and pinhole-free films is crucial for future semiconductor-based device applications, particularly diode junctions. Although IGM films are

rough, there are no obvious microscopically visible pinholes in the SEM top view images shown in **Supplementary Fig. 10a-f.**

We agree with the reviewer that a study of spin-dependent charge transport would provide compelling evidence for spintronic applications. This is beyond the expertise of our group, and we will seek collaboration to test our IGM films in devices.

Regarding roughness analysis using AFM results, we added the sentence in the revised main text and **Supplementary Note 2** in the revised Supplementary Information. Also, **Fig. R9** and **R10** are added into the revised SI as **Supplementary Fig. 12** and **13**, respectively. Please see below.

On page 10,

"Next, we take a closer look at the structural features of the chiral 2D perovskite films for both control and IGM conditions. At an *S*-MBAI concentration between 0.5 - 1.0 M, anisotropic growth was found, resulting in rod-like nanostructures with a width of tens of nm to 100 nm (**Fig. 3a-c** and **Supplementary Fig.** ~~**8-9**~~ **10-11**). If the concentration is too low, a typical polycrystalline structure is observed with submicrometer grain size; if the concentration is too high (exceeding 1.5 M), the rod-like nanostructures continue growing laterally into micrometer-sized platelets with incomplete substrate coverage. The nanorod morphology, which exhibits similar to its single crystals[32-34], suggests a higher structural order and better crystallinity. In contrast, the control film exhibits the crystals with rounded edges and agglomerated particles, as seen in the cross-sectional SEM image (**Fig. 3a**), indicating there might be substantial crystallographic defects and inhomogeneity. To further evaluate the surface topography of control and IGM (0.3 M and 1.0 M) films, we performed atomic force microscopy (AFM) measurements (see **Supplementary Fig. 12-13**, and **Supplementary Note 1**). These AFM results indicate poor coverage of control film, which is in line with the morphological results (**Fig. 1b** and **Supplementary Fig. 3a**)."

In the revised Supplementary information,

[**Supplementary Note 1**]

We conducted atomic force microscopy (AFM) measurements (**Supplementary Fig. 12**). The average surface roughness (R_a) and root mean square surface roughness (R_q) are quantitatively estimated on a 10 × 10 μm^2 scan area. The control film exhibits rough and poor coverage with R_a of 100.1 nm and R_q of 133.3 nm. In contrast, a lower surface roughness is observed for IGM samples (R_a of 26.88 nm and R_q of 33.44 nm for 0.3 M IGM, and R_a of 85.20 nm and R_q of 105.0 nm for 1.0 M IGM). Comparing different IGM concentrations, it was observed that the film roughness increases as the concentration of the *S*-MBAI solution increases, a trend consistent with the cross-sectional SEM results (**Supplementary Fig. 11**). This increased roughness can be attributed to the enhanced vertical growth of nanorod-like crystals.

To further characterize the uniformity of those films, we analyzed AFM line profiles with 4 lines per image (**Supplementary Fig. 13**). The control sample shows about one order of magnitude higher height fluctuations compared to the 0.3 M sample and slightly higher fluctuations compared to the 1.0 M sample. The line scans of the control sample seem to be dominated by large crystals with poor coverage while the IGM films show a more homogeneous distribution with smaller crystals.

8. Please clarify if there are any unreacted PbI2 or MBAI left on the film and they the residues play any roles in their chiroptical and optoelectronic properties. Please discuss the broad applicability of this IGM method, in particular, from the perspective of phase purity.

Ans) The reviewer raises important points. As described in the Methods section of the manuscript, the annealed films are immediately transferred to the spin coater for a washing step, where 200 µL of CB is slowly dispensed onto the spinning film. This rinsing step ensures the removal of residual gel phase or MBAI and is referred to as CB rinsing step. **Fig. R11** shows IGM films without and with CB rinsing. Before rinsing some covering layer is observed which disappears after rinsing. XRD confirms that after the CB rinsing step residual *S*-MBAI disappears.

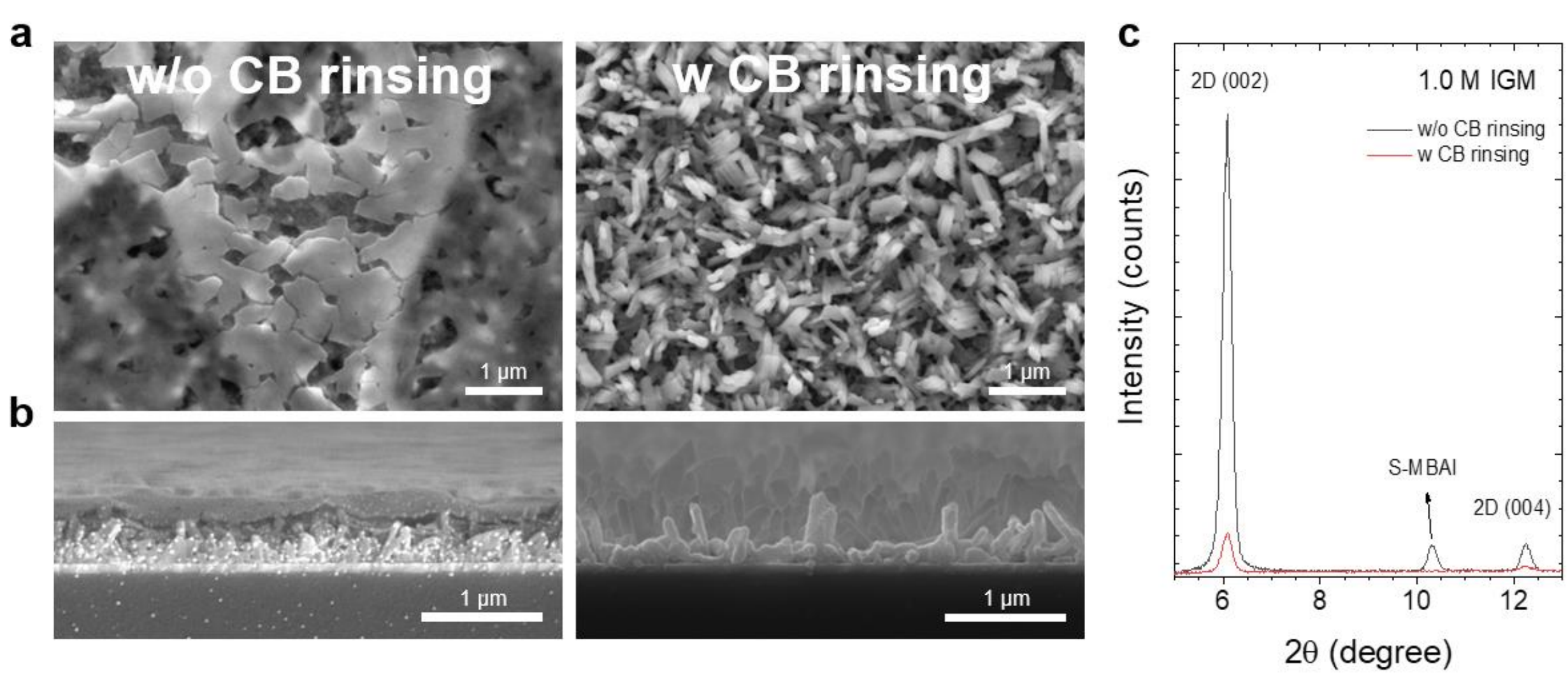

Fig. R11 a-c, (**a**) Top-view and (**b**) cross-sectional SEM images and (**c**) XRD patterns for 1.0 M IGM films without and with CB rinsing step.

We investigated the influence of unreacted PbI_2. XRD results (**Fig. R12**) indicate the co-presence of phase impurities with unreacted PbI2 and 1D phases for *S*-MBAI concentrations between 0.1 ~ 0.2 M. This is likely due to an insufficient supply of *S*-MBAI on the PbI_2 layer for phase conversion to 2D phase. The diffraction peak at 7.19° has not been identified, but it is expected to stem from

this S-MBAI deficiency. At *S*-MBAI concentrations > 0.3 M, fully converted 2D phase is observed. In **Fig. R12b**, a tiny amount of PbI_2 is induced due to CB rinsing. **Fig. R13** shows corresponding CD, absorbance, and g_{CD} data. Very low CD is observed for films grown with low *S*-MBAI concentration.

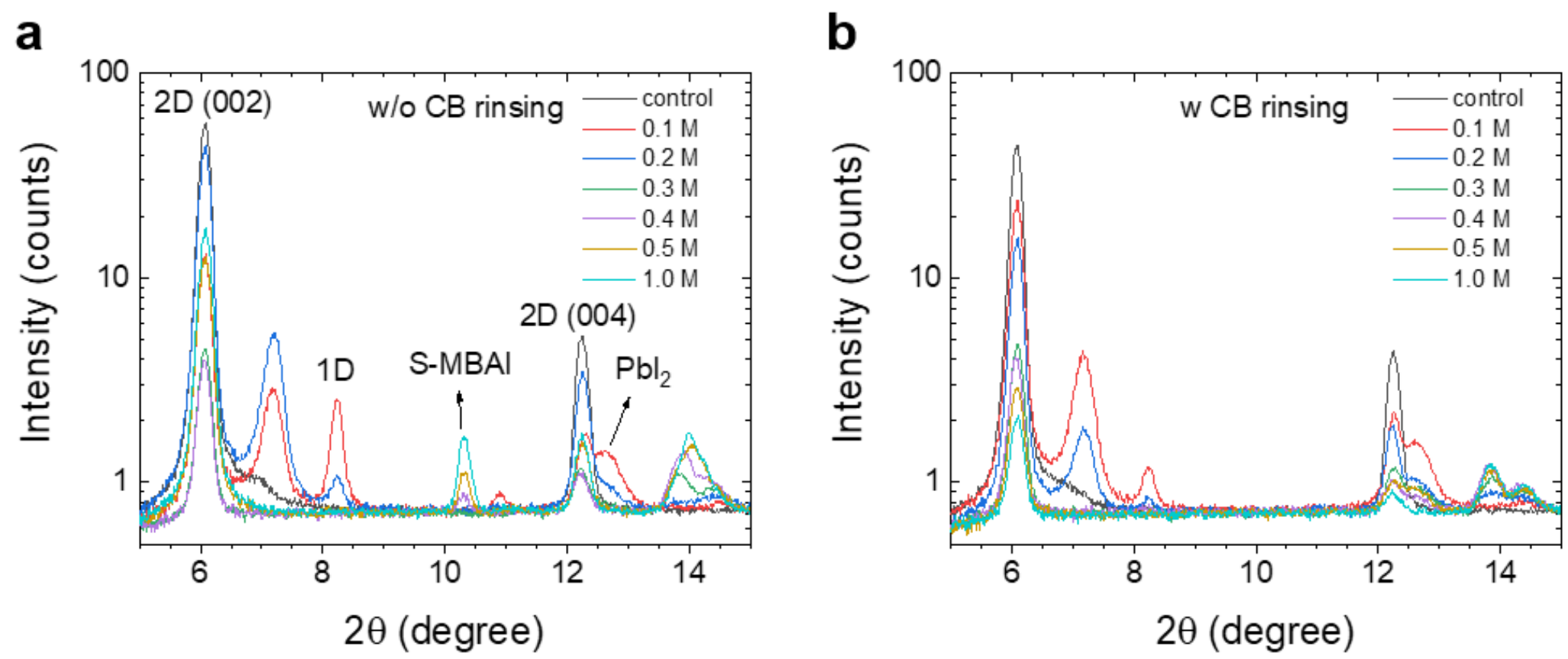

Fig. R12 a,b, XRD patterns for control and IGM (0.1 ~ 1.0 M) films (**a**) without and (**b**) with CB rinsing step.

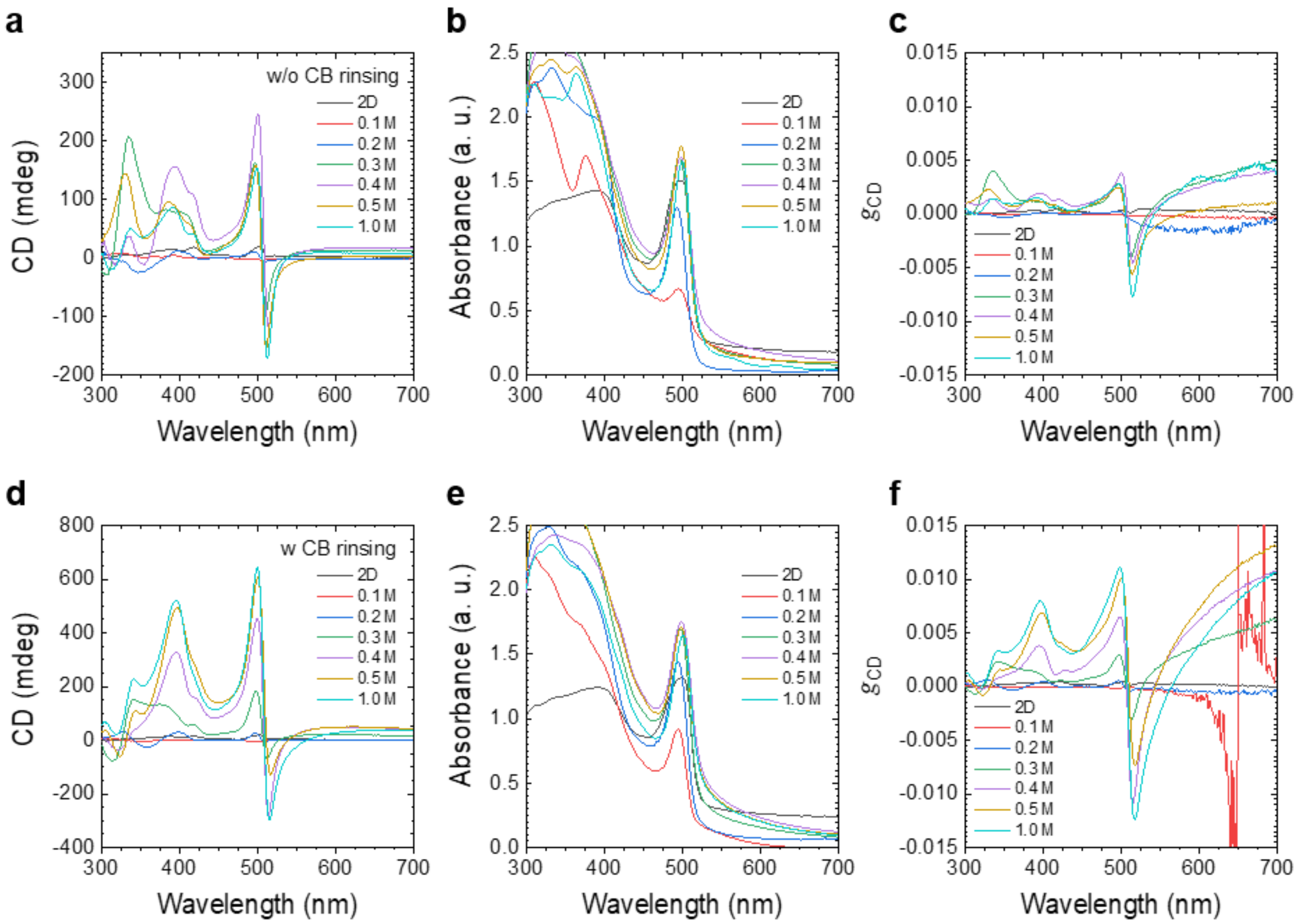

Fig. R13 a-f, (**a,d**) CD, (**b,e**) absorbance, and (**c,f**) g_{CD} spectra for control and IGM (0.1 ~ 1.0 M) films (**a-c**) without and (**d-f**) with CB rinsing step.

Now we turn our intention to the case where unreacted *S*-MBAI is observed. As seen in the XRD patterns (**Fig. R12**) for films w/o CB rinsing step, the diffraction peak at 10.3° corresponding to *S*-MBAI crystalline phase gradually appears as the S-MBAI concentration increases above 0.4 M. There is no *S*-MBAI associated peak in films with CB rinsing step. Chiroptical properties corresponding to these films are illustrated in **Fig R13**. CD intensities and g_{CD} at 500 nm are shown in **Fig. R14**. In the low S-MBAI concentration range of 0.1 ~ 0.3 M, CD intensities and g_{CD} values are comparable for both conditions, i.e. without and with CB rinsing step. However, above 0.4 M concentration, their chiroptical responses diverge dramatically. These observed differences might originate from the fact that the soluble ionic gel phase can hinder the formation of isotropic nanostructured crystals. To verify this hypothesis, we investigated the influence of the annealing time at 70 °C on the 1.0 M IGM film formation and their chiroptical response (**Fig. R15** and **R16**). As the annealing time was prolonged beyond 15 minutes, the nanostructured crystals grew in size and exhibited enhanced aggregation, resulting in the reduction of their isotropic feature as well as film coverage. These findings suggest that the remaining ionic gel phase on the film surface during annealing process tends to continuously deform the isotropic nanostructured crystals through a dissolution-recrystallization mechanism. It is potentially linked to the reduced CD response of IGM films.

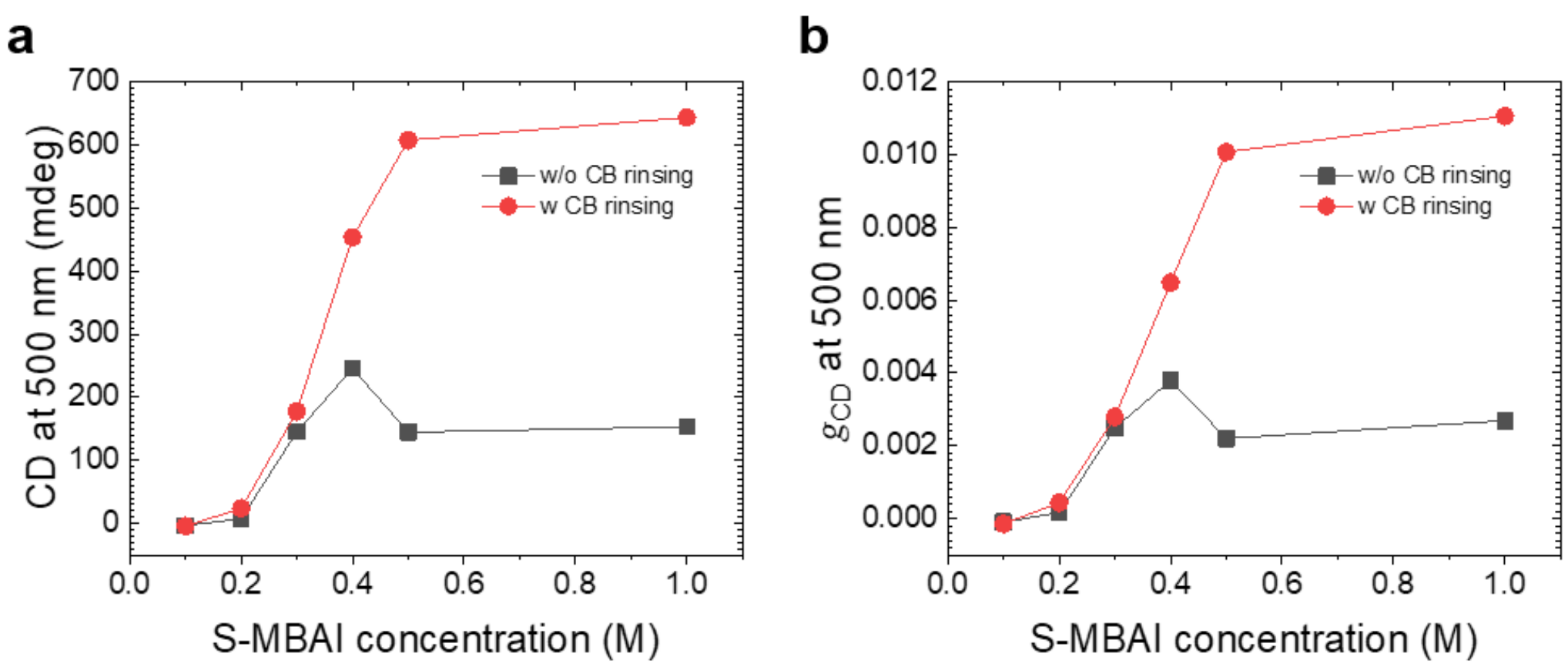

Fig. R14 (**a**) CD intensity and (**b**) g_{CD} at 500 nm and for IGM (0.1 ~ 1.0 M) films without and with CB rinsing step.

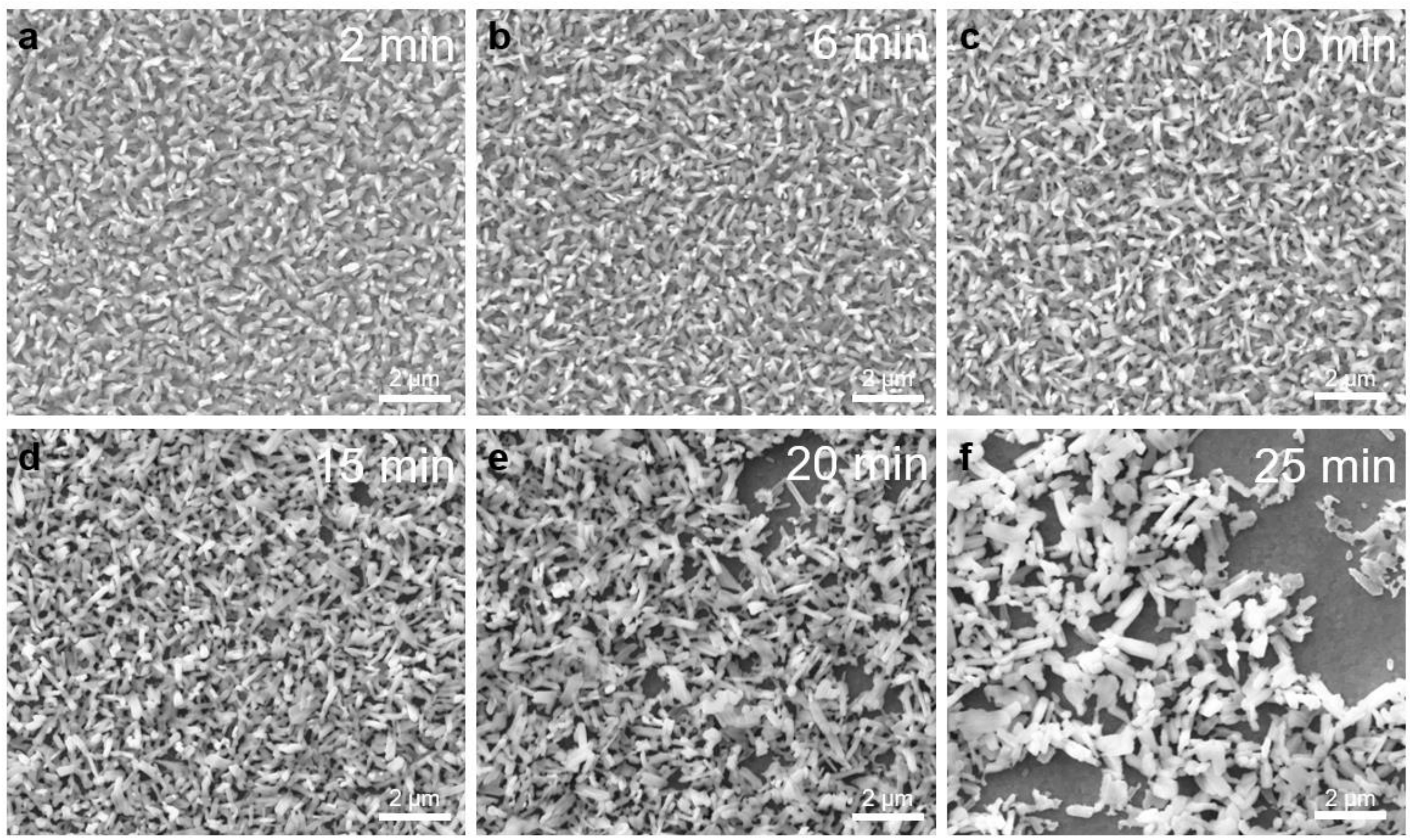

Fig. R15 Top-view SEM images of 1.0 M IGM films as different annealing times for (**a**) 2 min, (**b**) 6 min, (**c**) 10 min, (**d**) 15 min, (**e**) 20 min, and (**f**) 25 min. The films were annealed at 70 ℃ with CB rinsing step.

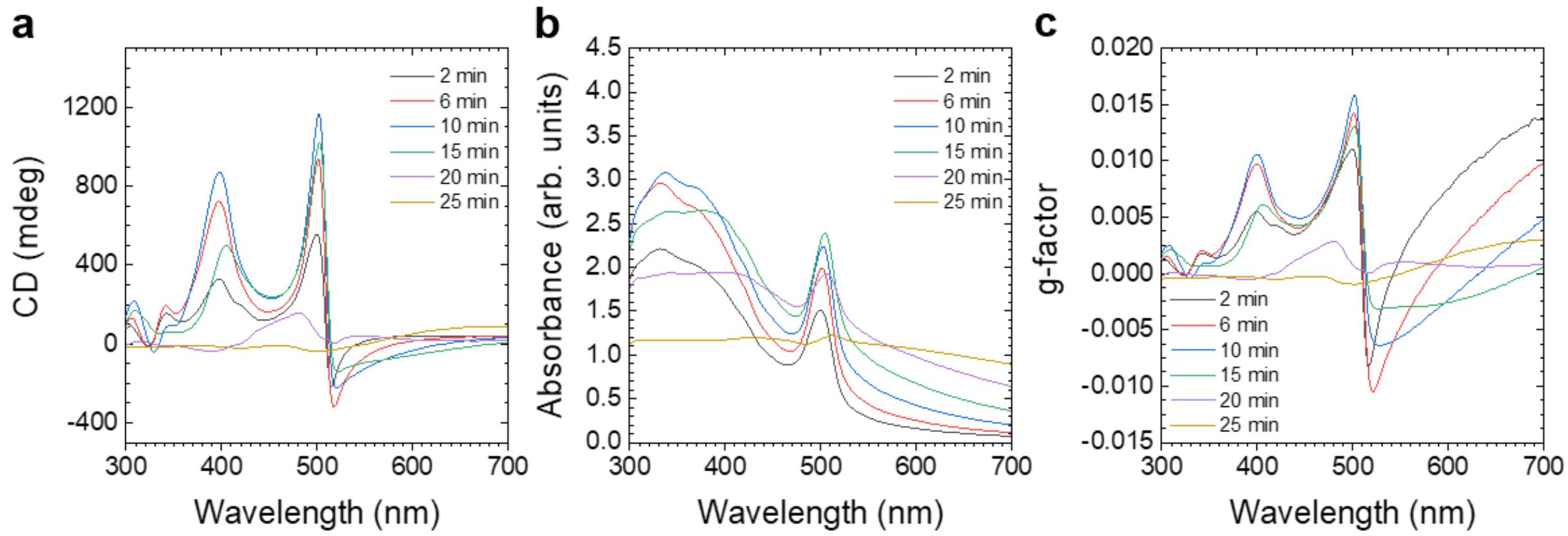

Fig. R16 (**a**) CD, (**b**) absorbance, and (**c**) g_{CD} spectra for 1.0 M IGM films as different annealing times for (**a**) 2 min, (**b**) 6 min, (**c**) 10 min, (**d**) 15 min, (**e**) 20 min, and (**f**) 25 min. The films were annealed at 70 °C with CB rinsing step.

Details about the residual S-MBAI ionic gel phase on the film surface were added as **Supplementary Note 3**. Please see below. Also, **Fig. R11~R14** are added into the revised Supplementary information as **Supplementary Fig. 23~26**, respectively.

We added to the Methods section in the main text (on page 20):
"To ensure the removal of residual gel phase or MBAI on the resulting IGM films, the annealed films on the hot plate were immediately transferred to the spin coater and subsequently washed with 200 μL of chlorobenzene by slowly dispensing it on the spinning substrates (see **Supplementary Note 4** for more details regarding the influence of CB rinsing step on IGM films)."

In the revised Supplementary information,

[**Supplementary Note 4**]

The rinsing step ensures the removal of residual gel phase or MBAI and is referred to as CB rinsing step. **Supplementary Fig. 24** shows IGM films without and with CB rinsing. Before rinsing some covering layer is observed which disappears after rinsing. XRD confirms that after the CB rinsing step residual S-MBAI disappears. As seen in the XRD patterns (**Supplementary Fig. 25**) for films w/o CB rinsing step, the diffraction peak at 10.3° corresponding to S-MBAI crystalline phase gradually appears as the *S*-MBAI concentration increases above 0.4 M. There is no *S*-MBAI associated peak in films with CB rinsing step. Chiroptical properties corresponding to these films are illustrated in **Supplementary Fig. 26**. CD intensities and g_{CD} at 500 nm are shown in **Supplementary Fig. 27**.

In the IGM synthesis route, the dissolution-recrystallization mechanism is the primary way in which the in-situ formed ionic gel phase on the film surface is involved during the spin-coating and annealing processes. This gel phase does not solidify when cooled down to room temperature. We found that the crystallization of this gel phase is triggered by exposure to the ambient air. An oily surface was observed for as-annealed films under the inert N_2 condition, indicating that the ionic gel phase still exists at the film surface without crystallizing. This indicates that the dissolution-recrystallization pathway might still be proceeding at the film surface. Therefore, these findings suggest that the presence of the residual ionic gel phase at the film surface is linked to the deformation of isotropic nanostructured crystals leading to lower CD response (**Supplementary Fig. 27**).

9. As Nature Communications is a multidisciplinary journal, it would be beneficial for the authors to include demonstration of potential applications, such as circularly polarized photodetectors in the manuscript to broaden its appeal to a wider readership.

Ans) We completely agree with the reviewer that the development of applications, such as circularly polarized photodetectors, are the ultimate goal for new functional materials. Our contribution has the primary objective and core scientific direction of introducing a novel method of synthesizing nanostructured thin films and investigating the origin of their amplified chiroptical response. We believe that this fundamental CD enhancement is a significant scientific advance on its own. It solves a key materials science problem and provides high-quality films that are a necessary prerequisite for the fabrication of high-performance devices. As mentioned above, a next step will be the collaboration with a device group to demonstrate working devices integrating our IGM films. Based on the reviewer's comment, we now state how the strong chiroptical response demonstrated here will directly benefit chiroptical device technologies. We hope this revision effectively bridges our fundamental study with the exciting applications it enables.

10. Authors should carefully check their manuscript for formatting issues. For example, there are redundant punctuation marks in line 37, incorrect characters in line 198, and inconsistent formatting in line 230.

Ans) We thank the reviewer for pointing this out. We carefully went over the manuscript to resolve any formatting issues.

Reviewer #2 (Remarks to the Author):

Comments:
This manuscript presents a study on the ionic gel-mediated (IGM) synthesis of nanostructured chiral 2D halide perovskites, reporting an enhanced chiroptical response with a dissymmetry factor (gCD) up to ~ 10^{-2}—higher than many values previously reported for similar systems. The two-step IGM approach appears to offer improved control over film morphology compared to conventional one-step methods, and the experimental data are generally well presented. Overall, the work is scientifically sound, clearly written, and makes a valuable contribution to the field of chiral optoelectronic materials. I would like to recommend the publication of this manuscript in Nature Communications after the following revisions:

We thank the reviewer for taking their time to provide positive feedback and encouraging publication in Nature Communications. We strive to improve the manuscript based on the constructive feedback provided. Please find our responses and revisions below.

1. The manuscript mentions experiments with racemic MBAI but doesn't show CD data for it. It would strengthen the argument if the authors explicitly confirm that racemic films show no CD signal—this helps rule out artifacts.

Ans) The reviewer raises an excellent point and we apologize for the sub-optimal graphical illustration. The CD data for the racemic conditions was already included in the manuscript and supporting information, but we acknowledge that it was not sufficiently visible in our previous **Figure 4a-c**. We changed the line width and color shades to make it more visible in the revised manuscript and supplementary information (**Fig. 4** and **Supplementary Fig. 16-17**).

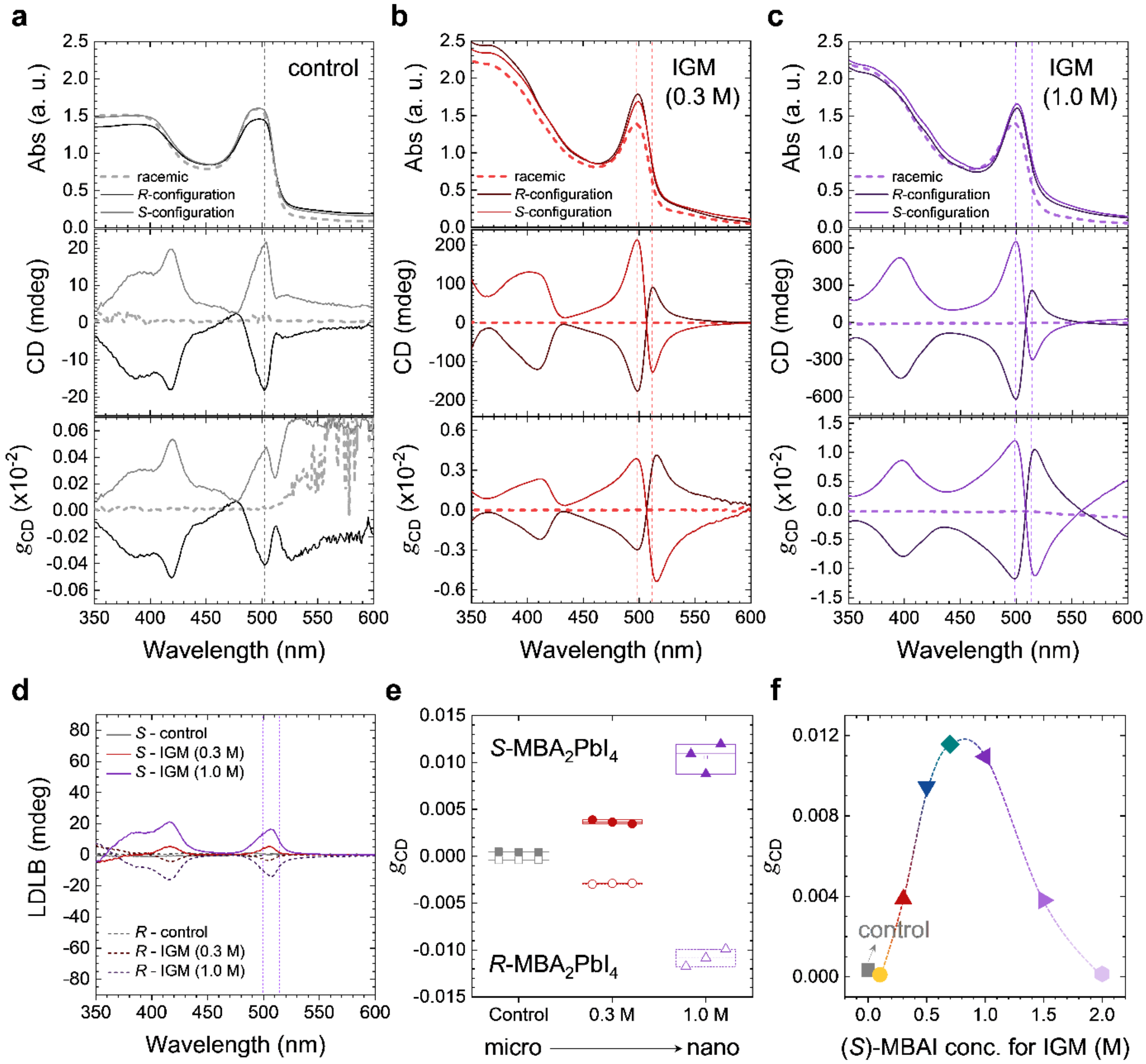

Updated **Fig. 4**. in the revised manuscript.

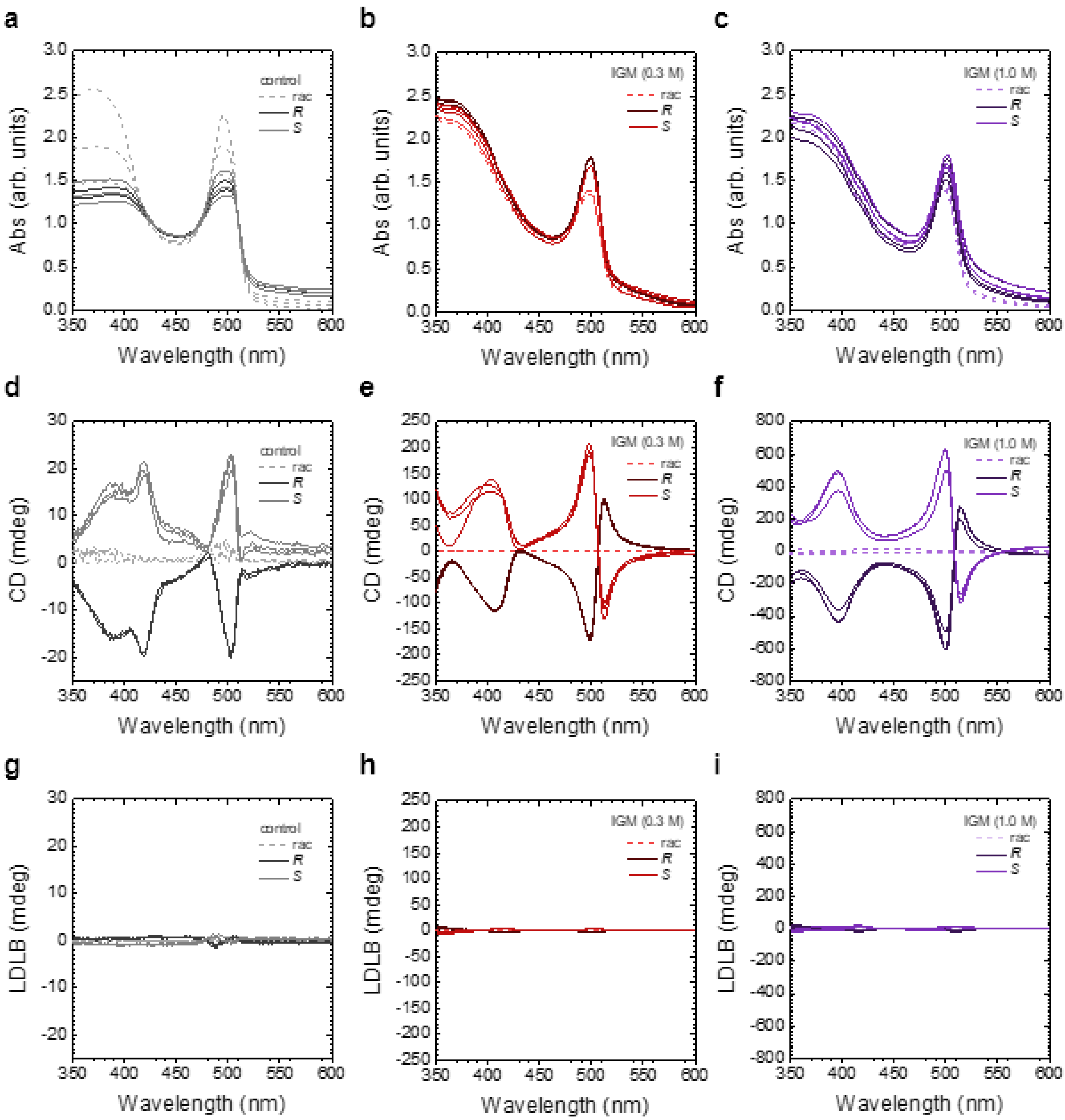

Updated **Supplementary Fig. 16**. in the revised Supplementary Information.

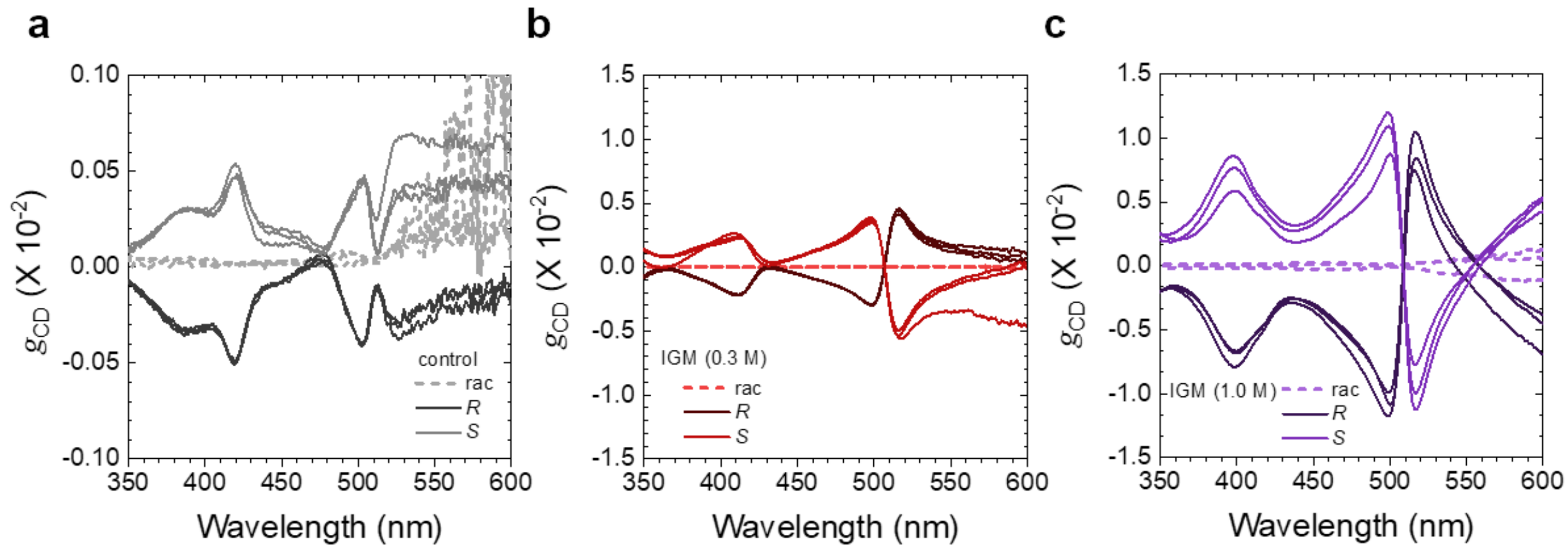

Updated **Supplementary Fig. 17**. in the revised Supplementary Information.

2. The authors reasonably dismiss exciton coupling and suggest Rashba-type spin splitting as the origin of the bisignate CD lineshape. However, this remains somewhat speculative. Could they briefly discuss whether magnetic circular dichroism (MCD) or magneto-optical measurements could further test this hypothesis?

Ans) We thank the reviewer for this important question and pointing out different mechanisms. In our work, we explained that the bisignate CD feature might rather originate from Rashba-type spin splitting of electronic transition states than from exciton coupling because the zero-crossing point in the bisignate CD couplets is not aligned with the excitonic absorption maximum. To provide further evidence to support our speculation, we analyzed temperature-dependent PL linewidth data (**Fig. 5d-f**). This PL linewidth analysis is able to estimate the electron-phonon coupling. It is well-known that a high phonon population is resulting in significant exciton scattering, leading to lower exciton coupling[R17,18]. The E_{LO} obtained from PL linewidth fitting represents an energy required to create an LO phonon. Higher E_{LO} means low LO phonon population, ideally leading to strong exciton coupling[R19]. If the appearance of the bisignate CD feature is dominated by exciton coupling, it should appear more clearly in the control film ($E_{LO,control}$ = 141 meV) than in the 1.0 M IGM film ($E_{LO,1.0\ M\ IGM}$ = 18 meV). This was not the case. Therefore, the origin of the bisignate CD feature is predominantly affected by spin splitting via strong electrodynamic couplings of dipoles.

On the other hand, we observed the crystal size is significantly reduced from the microscale (~ 5 μm for control film) to the nanoscale (100 ~ 500 nm for IGM films). However, the feature size of the nanostructured crystals in IGM films is insufficient to induce quantum confinement effect, thereby precluding the possibility of exciton coupling mediated by this effect.

In more detail, we estimated the nanostructured crystal size in the 1.0 M IGM film. In previous reports, chiral MBA_2PbI_4 single crystals typically form with rod or wire shape, growing (110) or (111) planes along the long axis[R20,21]. This means that the (00*l*) facet is face-up onto the flat surface. Compared to this, these nanorod-shaped crystals on the IGM film might have a similar orientation with chiral MBA_2PbI_4 single crystals growing (110) or (111) planes along a long axial. Despite

the presence of single-crystal-like crystalline domains within the IGM film, the GIWAXS results exhibit semi-ring shapes of the scattering pattern along (002) plane owing to isotropically distributed crystals. By approximately estimating the crystal size associated with the (002) facet in the IGM film, their dimensions are around 300 ~ 500 nm along the long axis and 70 ~ 120 nm along the short axis. These dimensions are not comparable to the Bohr radius (< 20 nm, typically for quantum dots), and thus, quantum confinement is excluded. Consequently there might not be significant exciton coupling in our system, so we assume that the contribution of the exciton coupling to the CD response is minor.

Lastly, thanks for the comment recommending the magnetic circular dichroism (MCD) measurement. Unfortunately we do not have access to MCD and magneto-optical instruments. We would like to use this study to follow up and collaborate with groups having such experimental capabilities.

Reference

[R17] Alvertis, A. M., Haber, J. B. & Neaton, J. B. Phonon screening and dissociation of excitons at finite temperature from first principles. *PNAS* **121**(30), e2403434121 (2024).

[R18] Sarkar, S., Kamath, N. S., Gayen, K. & Pal, S. K. Exciton-phonon coupling in quasi-two-dimensional Ruddlesden-Popper perovskites: impact of a mixed-phase structure. *Nanoscale* **17**, 10771-10783 (2025).

[R19] Gramlich M., Lampe, C., Drewniok J. & Urban, A. S. How exciton-phonon coupling impacts photoluminescence in halide perovskite nanoplatelets. *J. Phys. Chem. Lett.* **12**(46), 11371-11377 (2021).

[R20] Liu, Z. *et al.* Chiral hybrid perovskite single-crystal nanowire arrays for high-performance circularly polarized light detection. *Adv. Sci.* **8**(21), 2102065 (2021).

[R21] Zhao, Y. *et al.* Chiral 2D/quasi-2D perovskite heterojunction nanowire arrays for high-performance full-stokes polarization detection. *Adv. Optical Mater.* **11**(19), 2301239 (2023).

We added **Supplementary Note 2** in the revised Supplementary Information and revised the sentences in the revised manuscript. Please see below.

On page 16 in the revised manuscript,

"These measurements reveal a weak PL splitting of the excitonic emissions for the control, but a clear splitting for the IGM films, which originates from a more well-defined exciton fine structure, potentially stemming from the stronger ~~electron-hole exchange interactions~~ crystal domain homogeneity and minimized structural disorder in the material[~~35~~43].

The consistent PL peak splitting energy for both control and IGM films (12.8 meV) suggests that the spin-split states are preserved with identical electron-hole exchange interaction strength~~,~~. This indicates ~~indicating~~ that the fundamental mechanism responsible for this spin splitting induced by SOC is not significantly altered by the size and shape perturbations of chiral halide perovskite crystals. Even so, the imperfection induced by defects and lattice disorder can influence the

differential absorption of circularly polarized light and exciton formation associated with different spin states, ultimately impacting the rotatory strength of CD transitions."

On page 13 in the revised manuscript,

"The emergence of the bisignate curves is followed by an increase in the amplitude of both negative and positive bands. This is often an indication of exciton coupling, which occurs when two electric transition dipole moments are spatially close enough to couple, resulting in excited state splitting into two nondegenerate levels and bisignate CD response[~~29,30~~35,36]. In such cases, the sum of the integrated intensity of each couplet should be zero. However, in this work, the CD spectra for IGM films (**Fig. 4b-c**) exhibit excitonic peak positions aligned with the excitonic peak in the absorption spectrum (positive couplet for *S*-MBA_2PbI_4 or negative couplet for *R*-MBA_2PbI_4 in the bisignate CD spectrum). In addition, given that the observed nanorod-shaped crystals in the IGM film are orders of magnitude larger than the typical Bohr radius, exciton coupling is excluded. For ~~this~~ these reasons (further details are provided in **Supplementary Note 2**), we rule out the hypothesis of exciton coupling and speculate that the bisignate CD response might be more attributed to Rashba-type spin splitting of electronic transition states due to spin-orbit coupling (SOC)[~~31,32~~37,38]."

In the revised Supplementary Information,

[**Supplementary Note 2**]

In this section, we present the rationale for ruling out exciton coupling as the origin of the bisignate CD response:

i) Misaligned zero-crossing in bisignate CD spectra with excitonic absorption maximum in absorbance: Through temperature-dependent PL linewidth analysis (**Fig. 5c-f**) we estimate the electron-phonon coupling. It is well-known that a high phonon population is resulting in significant exciton scattering, leading to lower exciton coupling[1,2]. The E_{LO} obtained from PL linewidth fitting represents an energy required to create an LO phonon. Higher E_{LO} means low LO phonon population, ideally leading to strong exciton coupling[3]. If the appearance of the bisignate CD feature is dominated by exciton coupling, it should appear more clearly in the control film ($E_{LO,control}$ = 141 meV) than in the 1.0 M IGM film ($E_{LO,1.0\ M\ IGM}$ = 18 meV). This was not the case.

In addition, the spin splitting energy extracted from low-temperature PL spectra at 4 K is the same, indicating that the exciton coupling strengths via electron-hole exchange interaction is identical. Therefore, the origin of the bisignate CD feature is predominantly affected by spin splitting via strong electrodynamic dipole coupling.

ii) Dimensions of nanorods in IGM films: In previous reports, chiral MBA_2PbI_4 single crystals typically form rod or wire shapes, with (110) or (111) planes oriented along the long axis[4,5]. This means that the (00*l*) facet is face-up onto the flat surface. In comparison, our nanorod-shaped crystals in the IGM film might have a similar orientation as chiral MBA_2PbI_4 single crystals with (110) or (111) planes oriented along a long axis. Despite the presence of single-crystal-like domains within the IGM film, the GIWAXS results exhibit semi-ring shapes of the scattering pattern along (002) plane owing to randomly distributed crystals. We approximate the crystal size associated with the (002) facet in the IGM film, to be around 300 ~ 500 nm along the long axis and 70 ~ 120 nm along the short axis. These dimensions are not comparable to the Bohr radius (< 20 nm, typically for quantum dots). Furthermore, excitons are delocalized in in-plane of 2D layered crystal structures and possibly transferred between their inorganic interlayers with Therefore, there is likely no significant exciton coupling in our system, and we assume that the contribution of the exciton coupling to the CD response is minor.

References for the revised Supplementary Information,

1. Alvertis, A. M., Haber, J. B. & Neaton, J. B. Phonon screening and dissociation of excitons at finite temperature from first principles. *PNAS* **121**(30), e2403434121 (2024).
2. Sarkar, S., Kamath, N. S., Gayen, K. & Pal, S. K. Exciton-phonon coupling in quasi-two-dimensional Ruddlesden-Popper perovskites: impact of a mixed-phase structure. *Nanoscale* **17**, 10771-10783 (2025).
3. Gramlich M., Lampe, C., Drewniok J. & Urban, A. S. How exciton-phonon coupling impacts photoluminescence in halide perovskite nanoplatelets. *J. Phys. Chem. Lett.* **12**(46), 11371-11377 (2021).
4. Liu, Z. *et al.* Chiral hybrid perovskite single-crystal nanowire arrays for high-performance circularly polarized light detection. *Adv. Sci.* **8**(21), 2102065 (2021).

5. Zhao, Y. *et al.* Chiral 2D/quasi-2D perovskite heterojunction nanowire arrays for high-performance full-stokes polarization detection. *Adv. Optical Mater.* **11**(19), 2301239 (2023).

3. Figure 4e shows gCD distribution, which is good. But how many independent film batches were used to generate this statistic?

Ans) We thank the reviewer for appreciating the statistics in the data (**Fig. 4e**). To clarify, we prepared four independent batches on different days and compared their g_{CD}. as can be seen in **Fig. R17**. To clearly illustrate independent batch reproducibility, we added this figure to the revised Supplementary information as **Supplementary Fig. 18**.

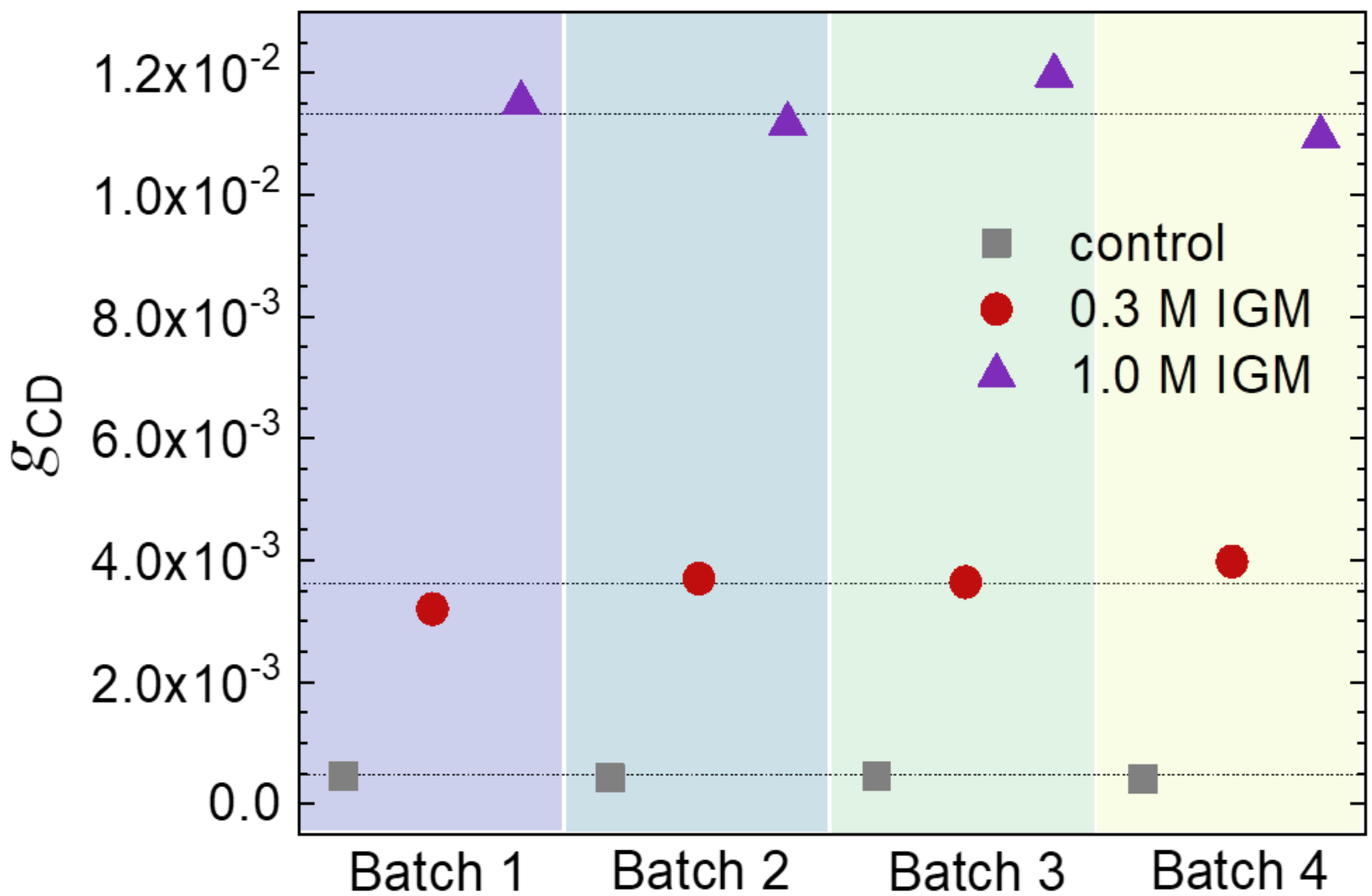

Fig. R17 g_{CD} measured in independent batches to illustrate reproducibility for control and IGM (0.3 M and 1.0 M) conditions. g_{CD} was obtained from CD spectra at ~ 500 nm corresponding to the excitonic absorption of *S*-MBA_2PbI_4.

On page 13 in the revised manuscript,

"Both control and IGM syntheses are very reproducible, as illustrated by the g_{CD} distribution shown in **Fig. 4e** and **Supplementary Fig. 16-17** (**Supplementary Fig. 18** for independent batches)."

4. The manuscript mentions using a "custom-made ionic gel," but doesn't specify its exact chemical components or preparation method. Since the IGM process is central to this work, readers will need enough detail to reproduce it.

Ans) Thank you for pointing this out and apologize for the confusion. We do not use a gel precursor to prepare the chiral perovskites on PbI_2 substrates because its viscosity hinders the formation of a uniform film (please see Q4 for reviewer 1). Instead, we use a *S*-MBAI solution in IPA to prepare the films, and the recipe for these solutions is described in the methods section. The ionic gel phase forms *upon* coating the PbI_2 substrates with the *S*-MBAI precursor solution due to the spinning of the film and subsequently during annealing. To support this hypothesis of gel formation on the surface of the film, we prepared a *S*-MBAI solution (S-MBAI salt dissolved in IPA) and tested its ability to gellify by storing the solution at 70 °C under the vacuum (**Supplementary Fig. 4**). Indeed, the ionic gel phase can be formed by evaporating residual IPA solvent. We anticipated that residual IPA molecules still exist due to the interaction between IPA and *S*-MBAI because the ionic gel phase remains stable even at the elevated temperature and vacuum conditions for a while.

Therefore, during the film formation in both spin-coating and annealing processes, the *S*-MBAI solution treated on the PbI_2 layer undergoes in-situ gelation by partially evaporating IPA solvent. Here, we use two terminologies: "gel-like intermediate phase" and "ionic gel phase". *S*-MBAI solution can dissolve the PbI_2 layer, with dependence on the *S*-MBAI concentration. To verify this, we dropped *S*-MBAI solutions with varying concentrations (0.3 M, 1.0 M, and 2.0 M) on the PbI_2 film, as can be seen in **Fig. R18** below. In-situ UV-Vis results (**Fig. 2c** and **Supplementary Fig. 8**) further corroborate this finding. During the spin-coating process, the gel phase consisting of S-MBAI and PbI_2 species are formed by evaporating IPA solvent, where we denote this phase as "gel-like intermediate phase". And then, during the annealing process, 2D chiral perovskite crystals form. The residual gel phase including excess *S*-MBAI left after film formation is denoted as "ionic gel phase". This ionic gel phase is responsible for the nanostructured crystal growth during the annealing process, and that is why we name it "ionic gel-mediated synthesis".

For example, for the 0.3 M IGM condition, the solubility of 0.3 M *S*-MBAI solution is insufficient to dissolve the underlying PbI_2 layer, so both intermediate phase (consisting of PbI_2 and *S*-MBAI species with residual IPA molecules) and ionic gel phase (consisting of *S*-MBAI with residual IPA molecules) exist on the film surface. In contrast, for the 1.0 M IGM condition, the treated 1.0 M *S*-MBAI solution can fully dissolve the PbI_2 layer, where the gel-like intermediate phase mostly remains on the as-coated film. During the crystallization process at 70 °C annealing temperature, the intermediate phase is converted to 2D chiral perovskites, and the ionic gel phase exists on the film surface. We confirmed this observation by comparing fresh and aged 2.0 M IGM films, please see the answer of Q6 for reviewer 1.

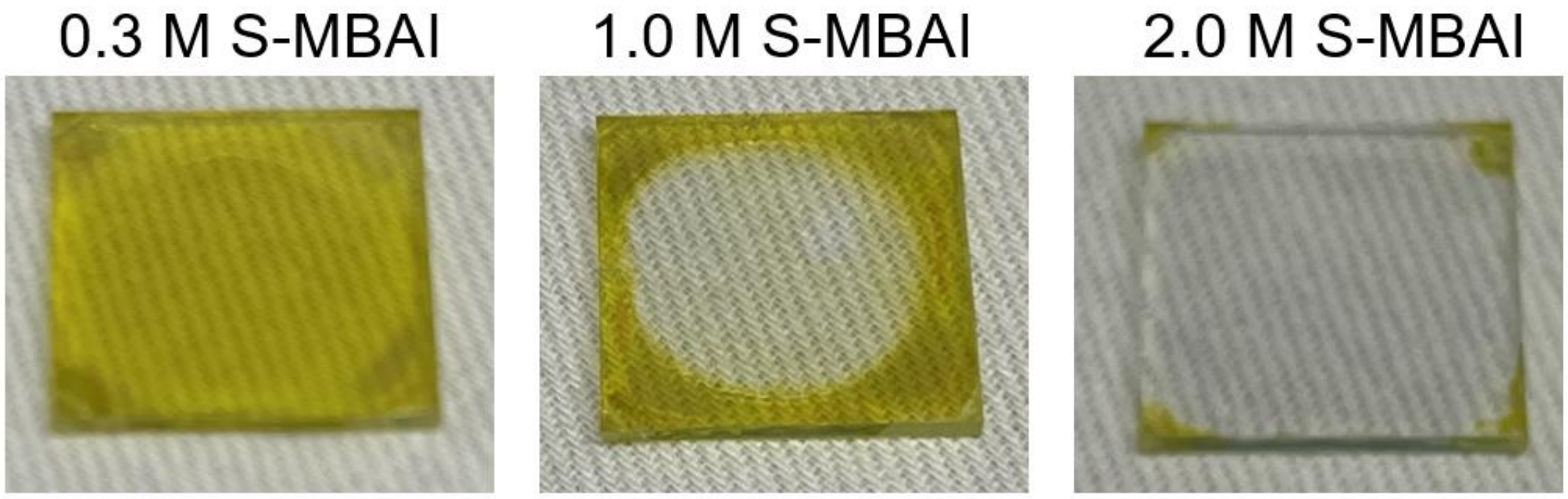

Fig. R18 Photographs of PbI_2 films in which different concentrations (0.3 M, 1.0 M, and 2.0 M) of *S*-MBAI solutions were dropped.

We added **Fig. R18** in the revised Supplementary Information as **Supplementary Fig. 5**. Also, to further elucidate the crystallization pathway of IGM synthesis, we added the sentence in the revised manuscript. Please see below.

On page 5 in the revised manuscript,

" We hypothesize that the IGM crystallization ~~features affect chiroptical response and propose that the IGM crystallization~~ plays a key role in the enhancement of the CD response~~, in particular in the second step where the MBAI solution is deposited on the PbI_2 film~~. In **Fig. 1d**, we propose a crystallization pathway of 2D chiral halide perovskite films via IGM synthesis. ~~In a test experiment Indeed,~~ ~~i~~It was found that an amorphous ionic gel (IG) phase is formed upon evaporation of the IPA solvent during spin coating and subsequent ~~heating~~ annealing (~~**Fig. 1d**, left side;~~ **Supplementary Fig. ~~3~~4**). According to this basis of our finding, the crystallization mechanism via IGM methods can be expected as following several steps during film formation; i) When treating *S*-MBAI solution onto the PbI_2 layer, the treated *S*-MBAI solution partially dissolves the underlying PbI_2 layer, starting from its surface. The extent of this solubility depends on the concentration of *S*-MBAI solution (**Supplementary Fig. 5**). For example, in the case of a low *S*-MBAI solution concentration, ionic gel phase consisting of unreacted *S*-MBAI and residual IPA predominantly remains on the as-coated film surface. On the other hand, for 1.0 M IGM condition, the treated *S*-MBAI solution can mostly dissolve the underlying PbI_2 layer, resulting in predominantly forming the intermediate phase. ii) During the spin-coating process, the IPA solvent is partially evaporated, promoting the *in-situ* formation of both gel-like amorphous intermediate (consisting of a complex of PbI_2, *S*-MBAI, and residual IPA) and ionic gel (consisting of a complex

of *S*-MBAI and residual IPA) phases on the surface. iii) During the annealing process, there are two crystallization processes. The first is that the intermediate phase is directly converted to 2D phase. The second is that *S*-MBAI ion species in ionic gel phase gradually diffuse into the underlying PbI_2 layer, where the elevated temperature triggers a phase transformation into the 1D (if *S*-MBAI species is insufficient) and/or 2D (if *S*-MBAI species is sufficient) phase based on a diffusion-driven phase conversion mechanism."

Also, to avoid confusion, we removed photographs on the left side of **Fig. 1d** and updated it in the revised manuscript.

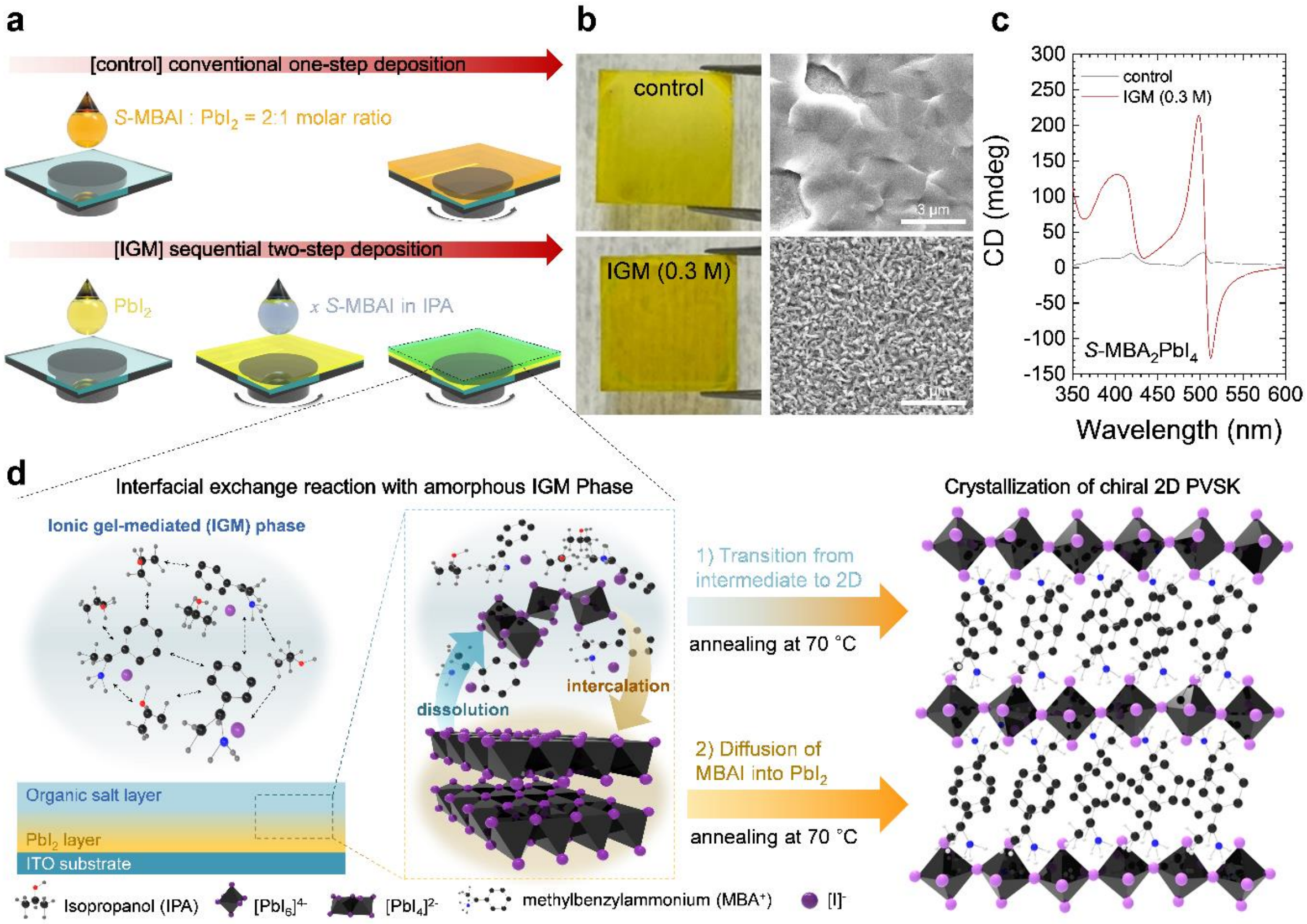

Updated **Fig. 1**. in the revised manuscript.

5. Chiral perovskites often suffer from environmental instability. Did the authors test the CD signal or PL intensity of their films after exposure to air?

Ans) That is a good point. Chiral perovskite materials are absolutely sensitive to environmental conditions, especially under a humidity condition, because of the hygroscopic ionic nature of their

crystal structure. To address the reviewers point, we tested environmental stability as shown in **Fig. R19** where the chiral perovskite films for control and IGM (0.3 M and 1.0 M) were exposed to the ambient air with ~ 70% relative humidity for up to 192 hours. At different points in time, absorbance and CD were measured. 1.0 M IGM films show the improved moisture stability compared to control and 0.3 M IGM films. Under humid conditions, we found that the 2D MBA_2PbI_4 phase converts to 1D $MBAPbI_3$ and hydrated phases, resulting in diminished CD intensity. Please also see our previous paper supported by first principle simulations[R22]. Based on our experimental findings we explain the enhanced moisture stability of the IGM samples by having less defects compared to the control.

References

[R22] Nurdillayeva, R. N. et al. Humidity disrupts structural and chiroptical properties of chiral 2D perovskites. *ACS Nano* **19**(11), 11348-11357 (2025).

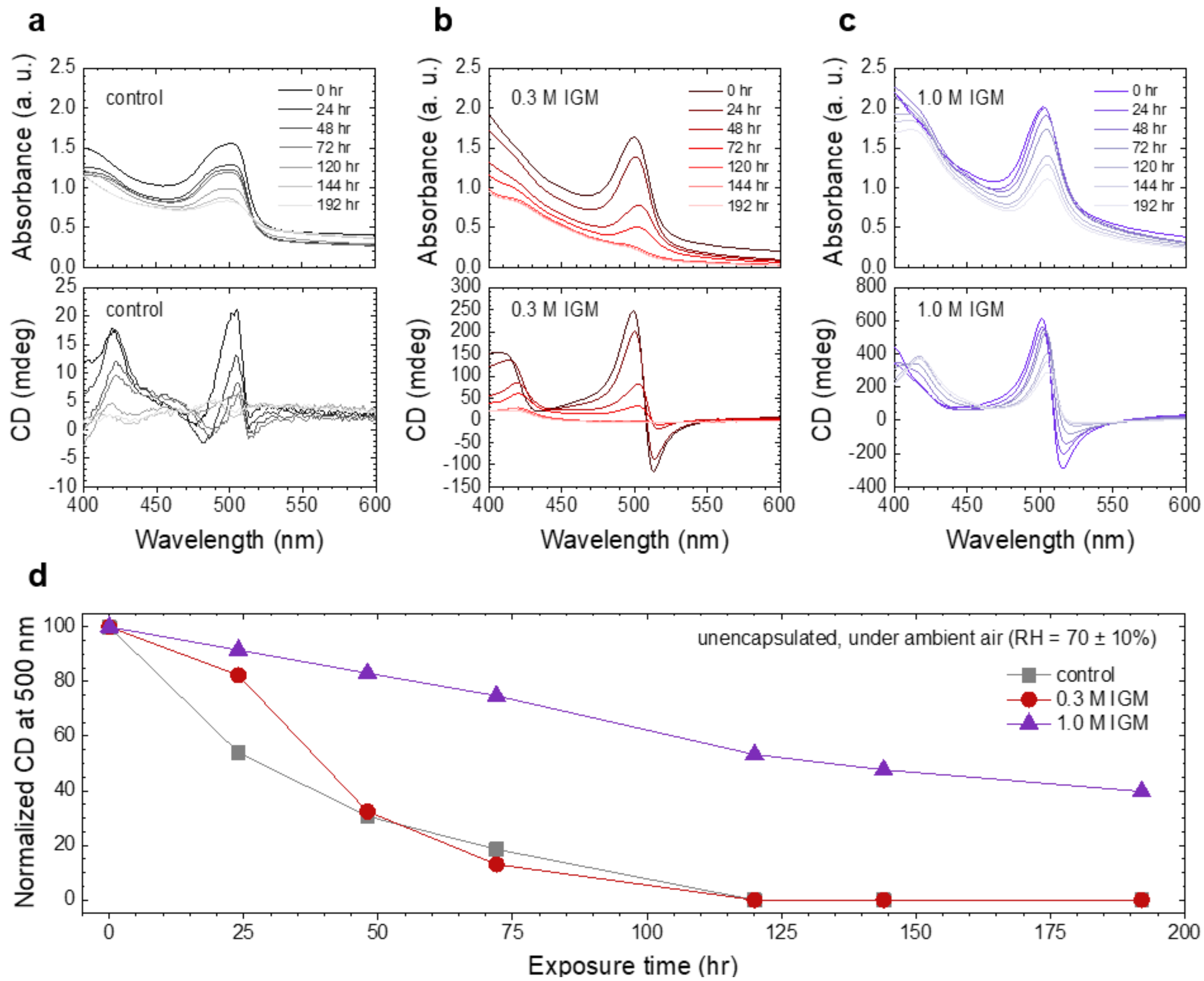

Fig R19 Moisture stability of chiral halide perovskite films for control and IGM (0.3 M and 1.0 M) with (**a-c**) absorption (top), CD (bottom), and (**d**) normalized CD at 500 nm over exposure time.

We added the following sentence in the revised manuscript, and **Fig. R19** in the revised Supplementary Information as **Supplementary Fig. 21**.

On page 15-16 in the revised manuscript:

"To further evaluate the beneficial effect of the IGM nanocrystalline structure on the excitonic transitions and oscillator strength, we performed temperature-dependent and time-resolved PL (TRPL) measurements. Room temperature PL measurements display monotonously increasing PL intensity from control to 0.3 and 1.0 M IGM samples (**Fig. 5a**). The higher PL intensity indicates increased contribution of radiative recombination, typical of less defective sites. TRPL confirms an increase in charge carrier lifetime by 80%, from 0.88 ns for the control to 1.58 ns for the 1.0 M

IGM film (**Fig. 5b**). The parameters obtained from fitted TRPL spectra are summarized in **Supplementary Table 1**, indicating that the number of defects is reduced in nanostructured IGM films. On the basis of our finding in PL results, the defects are reduced in high-quality nanostructured IGM films compared to microcrystals in control film, resulting in suppressing trap-mediated non-radiative recombination. We also tested environmental stability as shown in **Supplementary Fig. 21** where the chiral perovskite films were exposed to the ambient air with ~ 70% relative humidity for up to 192 hours. It suggests the enhanced moisture stability of the IGM films can be attributed to less defects and improved crystal quality. To further gain insights, we measured low temperature PL at 4 K Next, low temperature PL was measured at 4 K (**Fig. 5c**). These measurements reveal a weak PL splitting of the excitonic emissions for the control, but a clear splitting for the IGM films, which originates from a more well-defined exciton fine structure, potentially stemming from the stronger electron-hole exchange interactions crystal domain homogeneity and minimized structural disorder in the material[3543]."

6. The gCD value of ˜ 10^{-2} is impressive, but it would help readers gauge its significance if the authors briefly compare it to other state-of-the-art chiral semiconductors (e.g., chiral quantum dots, organic-inorganic hybrids, or chiral MOFs).

Ans) We thank the reviewer for appreciating our comparatively high g_{CD} value and agree that it's worthwhile to compare this to other materials. We added the following table in the revised supplementary information. Our g_{CD} value (~ 1.2 X 10^{-2}) exceeds most reported chiral perovskite thin films and is comparable to their single crystal counterparts as well as inorganic semiconductor clusters. Our comparatively high g_{CD} highlights the potential of synthesis engineering to boost chiroptical performance of hybrid metal halide perovskite thin films.

We added the following sentence in the revised manuscript, and **Supplementary Table 3** in the revised Supplementary Information.

On page 17 in the revised manuscript:

"This study presents an approach to amplify chiroptical response in chiral 2D MBA_2PbI_4 via IGM crystallization. Specifically, a dissymmetry factor g_{CD} of 1.2 × 10^{-2} with negligible linear dichroism and linear birefringence (LDLB) contribution was measured for the best IGM sample. This is among the highest intrinsic g_{CD} reported, *i.e.*, without any additional chiral additives, which is comparable to their single crystal counterparts as well as inorganic semiconductor clusters (**Supplementary Table 3**). "

In the revised Supplementary Information:

Supplementary Table 3. Comparison of reported g_{CD} values for various materials.

Type	Target material	$\|g_{CD}\|$	Ref.
Plasmonics	Au nanoparticles	0.2 at 622 nm	Nature, 2018, 556, 360-365.
	Pt on chiral BINOL-CTAC-covered helical-micelle Au nanorods	0.1 ~ 0.2 at 600 ~ 1200 nm	Science, 2020, 368(6498), 1472-1477.
	Au nanoparticles	0.4 at ~ 600 nm	Nature, 2022, 601, 366-373.
Semiconductor	Assembled CdS helical clusters	0.025 ~ 0.05 at 300 ~ 350 nm	Science, 2025, 387, eado7201.
	Cd28S17 cluster	0.00016 at 304 nm	J. Am. Chem. Soc., 2025, 147(21), 17890-17901.
	D-/L-Cys Co_3O_4 nanoparticles	0.01 ~ 0.02 at 550 nm	Science, 2018, 259(6373), 309-314.
Organic	Synthesized helical organic fluorenones	0.014 at ~ 350 nm	Nature Communications, 2025, 16, 9083.
Chiral Perovskites	(*S*-MBA)$_4Bi_2I_5Br_5$ single crystal	0.025 at ~ 400 nm	Sci. Adv., 2025, 11, eads6123.
	(*R*-/*S*-BrBA)$_2PbBr_4$ single crystal	0.14 at 405 nm	ACS Appl. Mater. Interfaces, 2025, 17, 17127-17134.
	(*R*-/*S*-3APr)PbI_4 single crystal	0.00117 at 495 nm	Adv. Mater., 2025, 37(10), 2414199.
	(*R*-/*S*-BrsBA)$_2PbBr_4$ single crystal	0.023 at 354 nm	Adv. Funct. Mater., 2026, 36(12), e08904.
	(*R*-/*S*-NEA)PbI_3 film	0.04 at ~ 400 nm	Sci. Adv., 2020, 6(46), adb3274.
	(*R*-/*S*-MBA)$_4Bi_2I_{10}$ film	0.001 at ~ 530 nm	Nano Lett., 2024, 24(3), 1001-1008.
	($R_{0.4}S_{0.6}$/$R_{0.6}S_{0.4}$-MBA)BiI_4 film	~ 0.03 at 550 nm	J. Am. Chem. Soc., 2025, 147(40), 36420-36427.
	(*R*-/*S*-MBA)$_2PbI_4$ film with TADDOLs additive	~ 0.006 at ~ 500 nm	Sci. Adv. 2024, 10, eado5942.
	(*R*-MBA)$_2PbI_{2.7}Br_{1.3}$ crystals grown on AAO template substrate	0.002 at ~ 490 nm	Nature Communications, 2022, 13, 3259.
	(*R*-/*S*-PEA)PbI_3 film	~ 0.02 at 400 nm	Nature Communications, 2019, 10, 1927.
	S-MBA_2PbI_4 film	0.00054	Adv. Funct. Mater., 2025, 35(2), 2413041.
	S-2F-MBA_2PbI_4 film	0.00168 at ~ 500 nm	
	S-4F-MBA_2PbI_4 film	0.00031	
	(*R*-/*S*-MBA)$_2PbI_4$ film	< 0.001	Angew. Chem. Int. Ed., 2021, 60, 21434-21440.

(R-/S-F-MBA)$_2$PbI$_4$ film	0.0004	
(R-/S-Cl-MBA)$_2$PbI$_4$ film	0.0031 at ~ 470 nm	
(R-/S-Br-MBA)$_2$PbI$_4$ film	0.0022 at ~ 470 nm	
**(R-/S-MBA)$_2$PbI$_4$ film**	**0.012 at 500 nm**	**This work**

Reviewer #3 (Remarks to the Author):

Comments:

In this manuscript, the authors demonstrated an ionic gel-mediated two-step spin coating method for nanostructured chiral MBA2PbI4 thin films. Based on in-situ GIWAXS patterns and absorption spectra, the authors unveiled that the MBAI ionic gel facilitates the formation of 1D [PbI3--MBA+] gel intermediate in stage II and thus the rod-like growth in the subsequent annealing process. Due to the high crystallinity and low defect density, the as-grown nanorod film shows the highest dissymmetry factor gCD of 1.2 × 10-2 among pristine chiral 2D perovskites, two orders of magnitude higher than that of the conventional one-step spin-coated film. These results would promote the design and fabrication of chiral 2D perovskite films towards spin-resolved optoelectronic applications. Therefore, to be accepted for publication in Nature Communications, the manuscript should address the questions listed below first:

We thank the reviewer for taking their time to carefully read our manuscript. We also appreciate that the reviewer points out the high g_{CD} and how this moves the field further towards spintronic applications. We address each point raised by the reviewer below.

1. The preparation of the MBAI ionic gel solution is pretty easy, simply dissolving the MBAI powder into the IPA. Is this a universal method? The authors should show more examples of this ionic gel-mediated method.

Ans) Although the two-step approach is simple, the gel formation significantly depends on the chemistry of the chiral molecule. To illustrate this we synthesized three different homo-chiral organic salts (S-MBAI: (S)-(-)-alpha-Methylbenzylammonium iodide, S-MeOMBAI: (S)-(-)-4-Methoxy-alpha-Methylbenzylammonium iodide, S-ClMBAI: (S)-(-)-1-(4-Chlorophenyl)ethylammonium iodide), dissolved them in isopropanol solvent, and evaporated the residual solvent using a rotary evaporator at 60 °C under the vacuum, as can be seen in **Fig R20**. Only the S-MBAI organic salt remains in gel form while the others transform into a powder. Possibly, other organic solvents need to be tested to transform the other organic salts tested here into a gel.

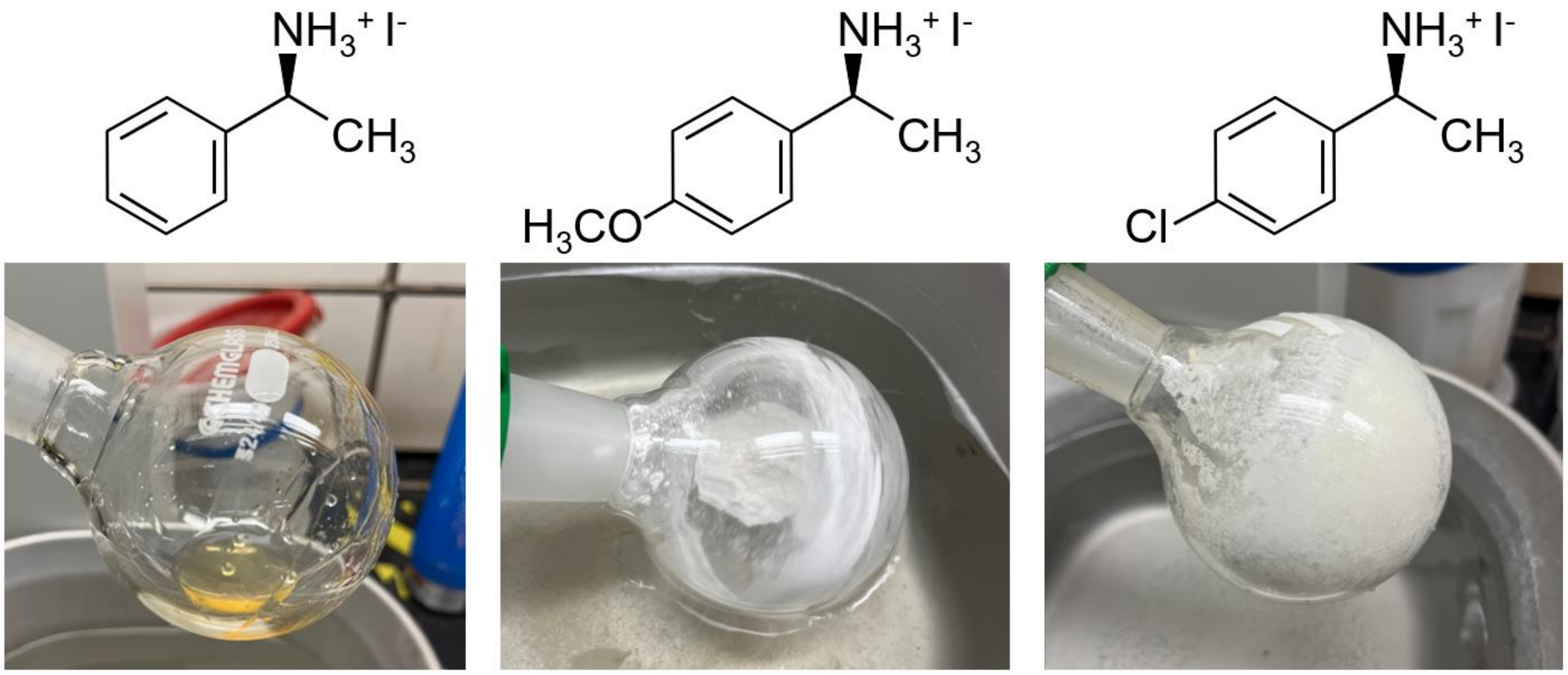

Fig. R20 Chemical structures (top) and photographs of the resulting products after evaporating residual IPA solvent using a rotary evaporator at 60 °C under the vacuum. From left to right, (*S*)-MBAI, (*S*)-MeOMBAI, and (*S*)-ClMBAI.

2. The authors assigned the enhanced chirality in IGM films partially to lower defect density, which is supported by the narrower PL linewidth and longer PL lifetime. 1) According to Figure 5e, the FWHMs of IGM films are actually larger than those of control films above ~90K. I assume this is a fitting issue. The authors need to double-check it. 2) According to Figure 5b, there are two PL decay channels, in which the fast radiative recombination takes the dominant role. Therefore, the average lifetime is more related to the radiative recombination and is not a good indicator for defect-related non-radiative recombination. It is suggested that the authors show the fitting results for TRPL and compare the non-radiative lifetime.

Ans) We thank the reviewer for the constructive comments. Accordingly, we double-checked the fitting results of the temperature-dependent PL spectra (**Fig. R21**) and we revised the fitting results for the X_1 excitation state in **Fig 5e** in the revised manuscript. We point out that larger FWHM values for the 1.0 M IGM sample above ~90 K are because this sample does not show a peak splitting for the datapoints within the 50 to 130 K window. Therefore, any FWHM value within this window for the IGM sample will be larger compared to decoupled FWHM values (X_1 and X_2) of the control samples. That is why we focus our discussion on the low temperature region (< 90 K) that shows clearly narrower FWHM values for the IGM sample. In addition, the FWHM of the IGM sample at temperatures above 130 K is also narrower as can be verified from the overlap of the normalized emission spectra at 240 K. Therefore, this apparent larger FWHM for the IGM sample is indeed a limitation of the fitting.

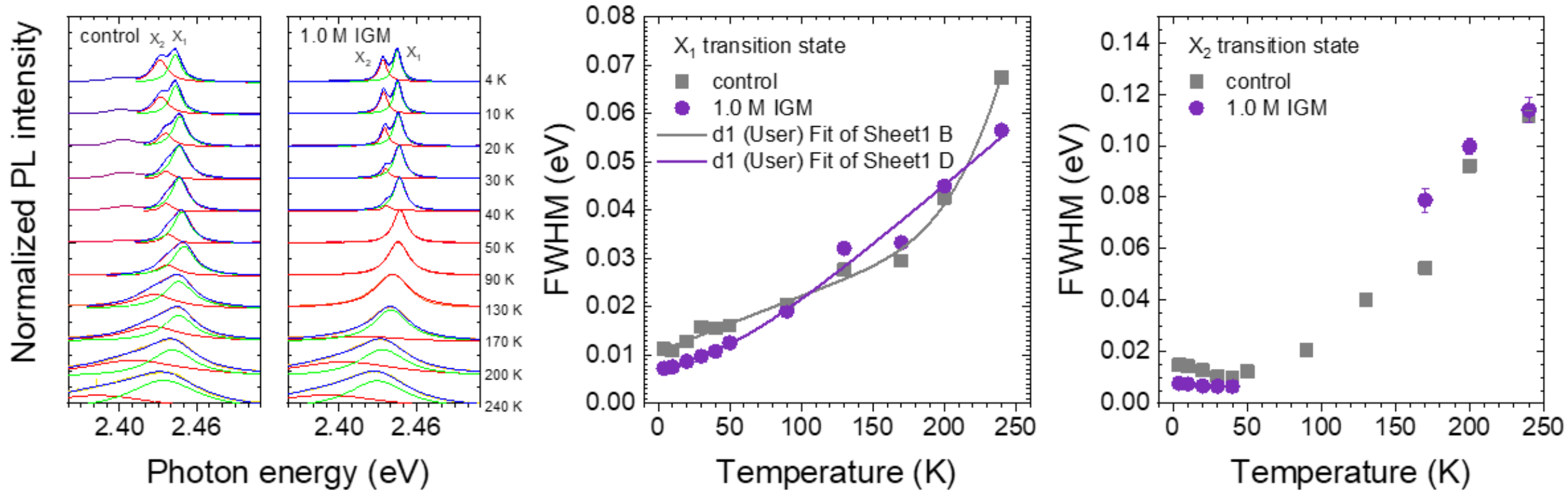

Fig. R21 a, Fitting results of temperature-dependent PL spectra using multi-peak Lorentz function. **b-c**, Analysis of temperature-dependent PL linewidth broadening associated with (b) X_1 and (c) X_2 transition states.

In order to evaluate the average charge carrier lifetime contribution in detail, we compare the fitted parameters obtained from room temperature time-resolved photoluminescence spectra, in Table R1. There are fast (A1 and t1) and slow (A2 and t2) components, where A_x indicates the proportionally weighted value and t_x is a recombination lifetime of excited charge carriers. In general, fast components are associated with trap-mediated non-radiative recombination, while slow components are corresponding to radiative recombination. Average charge carrier lifetimes were calculated using $(A_1t_1^2 + A_2t_2^2)/(A_1t_1 + A_2t_2)$ equation, which comprehensively includes *overall recombination pathways* (see details below) on the sample. Considering the fast components (A_1 and t_1) trap-mediated non-radiative recombination rate is obviously decreased, with lifetime values of from 0.2492 ns for control, to 0.2681 ns for 0.3 M IGM, and 0.3193 ns for 1.0 M IGM, with weighted fractions decreased from 92.90% for control to 90.19% for 0.3 M IGM and 90.56% for 1.0 IGM. On the other hand, for the slow components (A_2 and t_2), although control and 0.3 M IGM are similar, the average lifetime is remarkably increased for 0.3 M and 1.0 M IGM samples. The results of these fast components associated with non-radiative recombination pathways are in line with $\square_0$ term results at low temperatures (4 ~ 40 K) extracted from temperature-dependent PL linewidth analyses. As a result, we expect that the nanostructured crystals from the ionic gel-mediated synthesis method exhibit high crystal quality with less defects compared to the control. The improved environmental stability for IGM samples discussed in answer of Q5 for reviewer 2 is another indication of that.

We would like to emphasize that the radiative recombination is always coupled to the non-radiative recombination, even though we can only detect the radiative channels. To illustrate this, let us consider the depletion of an excitated charge carriers' population in a chiral perovskite (E*) *via* three recombination channels: one non-radiative (X_{nr}) and two radiative ($A_{1(r)}$ and $A_{2(r)}$). Hence:

$$\square * \rightarrow \square_{\square\square} + \square_{1(\square)} + \square_{2(\square)},$$

each channel with a rate 1/□$_X$, 1/□$_{A1}$, and 1/□$_{A2}$, respectively. The overall recombination rate (1/□$_E$) will be a function of all three rates from each individual channel, X_n, $A_{1(r)}$, and $A_{2(r)}$, such that:

$$\square_{\square} = \frac{1}{\square_{\square}} = \frac{1}{\square_{\square}} + \frac{1}{\square_{\square 1}} + \frac{1}{\square_{\square 2}}$$

and the PL decay is given by

$$\square_{\square\square} = \square_{0}.\,\square^{-\square_{\square}\square}$$

Therefore, even probing only the radiative channels (photon counting), the non-radiative channel is intrinsically accounted for.
Now, considering that the photoluminescence quantum-yield (PLQY) of $(PEA)_2PbI_4$ 2D perovskite (a benchmark composition) is approximately 0.3%[R23], the PLQY (□$_f$) of (R-/S-MBA)$_2$PbI$_4$ must be similar. Hence:

$$\Phi_{\square} = \frac{\square_{\square}}{\square_{\square}+\square_{\square\square}} = \frac{(1/\square_{\square})}{(1/\square_{\square})+(1/\square_{\square})} \approx 0.003,$$

where 1/□$_A$ is the total radiative recombination rate considering channels A_1 and A_2. With this analysis, it is clear that the non-radiative recombination channels are *dominant* in the recombination dynamics of (*R*-/*S*-MBA)$_2$PbI$_4$ chiral 2D perovskites. Thus, our average lifetime is much more related to the non-radiative channels than to the radiative ones, irrespective of the weighted contribution of the individual radiative channels. With that said, it is safe to conclude that the substantial average lifetime improvement in the IGM samples is indeed related to a decrease in non-radiative recombination due to a lower density of defects and better crystallinity of the nanostructured films.
As mentioned before, fast components are associated with trap-mediated non-radiative recombination. The fact that the weighted contributions of the fast component decreases from control to IGM samples (A_1 in Table R1), clearly shows that our IGM synthesis results in nanostructured materials with a lower density of defects.

References
[R23] Ma, P., Zhuang, Y., Lin, H., You, G., Xu, G. & Cai, B. Quantum yield and stability improvement of two-dimensional perovskite via a second fluorinated insulator layer. *Adv. Mater. Interfaces* **10**(9), 2202003 (2023).

Table R1 Fitted parameters extracted from TRPL spectra. A bi-exponential decay function has been used ($y = y_0 + A_1*exp(-(x-x_0)/t_1) + A_2*exp(-(x-x_0)/t_2)$). The average lifetime was calculated using $(A_1t_1^2 + A_2t_2^2)/(A_1t_1 + A_2t_2)$ equation.

	control	0.3 M	1.0 M
A_1	0.7424 (92.90%)	0.6477 (90.19%)	0.53446 (90.56%)
t_1 (ns)	0.2492	0.2681	0.3193
A_2	0.05671 (7.10%)	0.0704 (9.81%)	0.05569 (9.44%)
t_2 (ns)	1.9423	1.9555	2.9142
$t_{average}$ (ns)	0.8810	1.0146	1.5841

We added the following sentence in the revised manuscript, and **Table R1** in the revised Supplementary Information as **Supplementary Table 1**.

"To further evaluate the beneficial effect of the IGM nanocrystalline structure on the excitonic transitions and oscillator strength, we performed temperature-dependent and time-resolved PL (TRPL) measurements. Room temperature PL measurements display monotonously increasing PL intensity from control to 0.3 and 1.0 M IGM samples (**Fig. 5a**). The higher PL intensity indicates increased contribution of radiative recombination, typical of less defective sites. TRPL confirms an increase in charge carrier lifetime by 80%, from 0.88 ns for the control to 1.58 ns for the 1.0 M IGM film (**Fig. 5b**). The parameters obtained from fitted TRPL spectra are demonstrated in **Supplementary Table 1**."

3. Following the previous question, another possible reason for enhanced chirality is the improved crystallographic orientation. The reported dissymmetry factor gCD value is even comparable to that of the chiral 2D perovskite single crystal (ACS Appl. Mater. Interfaces 2025, 17, 17127−17134). It is highly suggested that the authors provide a table to summarize the chiroptical metrics for 2D perovskites.

Ans) It's great to see the reviewer's excitement. As the reviewer suggested, we added **Supplementary Table 3** in the revised Supplementary Information. We compared the circular dichroism dissymmetric factor (g_{CD}) of chiral metal halide perovskite single crystals and polycrystalline films as well as various other semiconductor and plasmonic materials. Please see Q6 by reviewer 2.

4. What are the fitted ELO energies in Figure 5e? Do they correspond to different Raman modes for control and IGM films, respectively?

Ans) Each fitting parameter obtained from temperature-dependent PL linewidth analysis demonstrated in **Table R2** below. To obtain these parameters, we fitted FWHM of PL linewidth at different temperatures based on the following equation,

$$\Gamma(T) = \Gamma_0 + \Gamma_{ac} + \Gamma_{LO} + \Gamma_{imp}$$

$$= \Gamma_0 + \gamma_{ac}T + \gamma_{LO}\left(e^{(E_{LO}/k_B T)} - 1\right)^{-1} + \gamma_{imp}\, e^{-(E_b/k_B T)}$$

where, γ_{ac} and γ_{LO} indicate the charge-carrier (or exciton)-phonon coupling strengths with acoustic and longitudinal optical phonons, respectively. E_{LO} represents an energy of LO phonon involved in the phonon scattering. As we mentioned in the manuscript, the contribution of ionized impurities can be omitted based on the profile of the curve given by FWHM as a function of temperature[R23]. Furthermore, γ_{ac} values for both control (1.150×10^{-4}) and 1.0 M IGM (1.011×10^{-4}) are similar, and much lower than γ_{LO} values. This means that the contribution of the acoustic phonons to the PL linewidth broadening induced by exciton scattering is negligible. **According to these results, we expect that the LO phonon mode is dominant for both control and IGM films.** By comparing the E_{LO} values for each condition (141 meV of control and 18 meV of 1.0 M IGM), we can evaluate the energy of the dominant phonon mode responsible for exciton scattering. For the 1.0 M IGM condition, the E_{LO} value is much lower than the thermal energy at room temperature (25.85 meV at 300 K), indicating that the LO phonon, even at low temperature, is able to scatter excitons.

We do not assign these LO phonon energies to any specific vibrational mode in the material because it would require detailed Raman/FTIR correlation. In addition, because of the limitation in the fitting of our low-temperature PL data (discussed above in Q2), these phonon energies are approximate values and may not match the energy of actual vibrational modes in the material. This detailed assessment, however, falls out of the scope of this manuscript.

It is known that the soft crystal structure of organic/inorganic layered metal halide perovskites is prone to intense exciton-phonon coupling[R24]. In addition, in 2D perovskites, these phonons can originate from high-energy vibrations (*e.g.*, 141 meV) from the organic spacers[R25]. We hypothesize that these different phonon energies (18 and 141 meV) reflect the difference in crystallinity between the control and IGM samples. The LO energy extracted from the control sample (141 meV) can be an indication of exciton-phonon coupling with vibrational modes from the methylbenzyllamonium cations. Local disorder and residual strain in less crystalline control samples can induce more intense exciton-phonon scattering[R26], consistent with a higher γLO value (~27 meV, **Table R2**).

References
[R23] Wright, A. D. et al. Electron-phonon coupling in hybrid lead halide perovskites. *Nat. Commun.* **7**, 11755 (2016).
[R24] Spirito, D., Asensio, Y., Hueso, L. E. and Martín-García, B. Raman spectroscopy in layered hybrid organic-inorganic metal halide perovskites. *J. Phys. Mater.* **5**, 034004 (2022).
[R25] Moral, R. F. et al. Influence of the vibrational modes form the organic moieties in 2D lead halides on excitonic recombination and phase transition. *Adv. Optical Mater.* **8**(24), 2001431 (2020).
[R26] Du, Q. et al. Stacking effects on electron-phonon coupling in layered hybrid perovskites via microstrain manipulation. *ACS Nano* **14**(5), 5806-5817 (2020).

Table R2 Fitting parameters obtained from temperature-dependent PL linewidth analysis for X_1 transition state.

	control	1.0 M
$\Gamma 0$ [eV]	1.071×10^{-2}	0.670×10^{-2}
γac [eV/K]	1.150×10^{-4}	1.011×10^{-4}
γ_{LO} [eV]	26.998	0.033
E_{LO} [eV]	0.141	0.018

We added **Table R2** in the Supplementary Information as **Supplementary Table 2**, and revised the paragraphs regarding the temperature-dependent PL linewidth analysis as follows:

On page 16-17 in the revised manuscript,

"Lastly, ~~the narrower linewidth and higher intensity of low temperature PL signals for IGM films also indicate less structural disorder and imperfections[36,37]. It was reported that phonon scattering governs emission linewidth broadening in halide perovskites and limits charge-carrier mobilities[3644]. T~~ to get a deeper understanding of exciton-phonon interactions, we analyze the different contributions to linewidth broadening Γ~~(T)~~(T) by extracting the temperature-dependent FWHM of the PL spectra for the control and 1.0 M IGM samples (**Fig. 5e**). We apply a modified PL linewidth model to the data to differentiate scattering mechanisms caused by optical phonons and crystal imperfections (**Equation (1)**),

$$\Gamma(T) = \Gamma_0 + \Gamma_{ac} + \Gamma_{LO} = \Gamma_0 + \gamma_{ac}T + \gamma_{LO}(e^{E_{LO}/k_BT} - 1)^{-1} \quad (1)$$

where $\Gamma_{(T)}$ is the linewidth at temperature T, Γ_0 is the temperature-independent linewidth due to inhomogeneities such as disorder and crystalline imperfections, γ_{ac} and ~~Γ~~ γ_{LO} is the analog broadening term for ~~the~~ acoustic and longitudinal optical (LO) phonon ~~(LO)~~, respectively, E_{LO} is the energy of the LO phonon, and k_B is the Boltzmann constant. Given that the contributions of acoustic phonons and ionized impurities can be negligible based on the characteristics of the temperature-dependent FWHM plot[~~36~~44], the related terms in ~~full~~ the PL linewidth equation were omitted. Γ_0 (X_1) is ~~11~~ 10.71 meV and ~~7~~ 6.70 meV for the control and 1.0 M IGM, respectively, confirming higher inhomogeneity in the control sample (**Fig. 5e-f**). The same is observed in the X_2 excitonic transition, with Γ_0 of 14.34 meV and 7.22 meV for control and 1.0 M IGM samples, respectively (**Supplementary Fig. ~~16~~22**). The narrower linewidth and higher intensity of the PL signals at 4 ~ 40 K for IGM films indicate less structural disorder and imperfections[44-45]. Additionally, higher crystalline imperfections and lattice distortions result in increased scattering by optical phonons, contributing to the PL linewidth[~~38~~46]. This is reflected by the significantly larger ~~Γ~~ γ_{LO} terms associated with X_1 transition state ~~for both X_1 and X_2 transition states~~, with values of ~~91~~ 26.998 meV for control ~~and 20 meV, respectively~~; in contrast, the 1.0 M IGM sample showed considerably lower values of ~~24~~ 0.033 meV for X_1 transition state ~~and 6 meV for X_2 transition state~~. γ_{ac} values for both control (1.150×10^{-4} eV K^{-1}) and 1.0 M IGM (1.011×10^{-4} eV K^{-1}) are similar, and much lower than γ_{LO} values. This means that the contribution of the acoustic phonon to the PL linewidth broadening induced by exciton scattering is negligible, and the LO phonon in the control film is more dominantly influenced by exciton scattering than in IGM films."

A point-by-point response

Journal: Nature Communications
Manuscript ID: NCOMMS-25-86761-T
Title: Ionic gel-mediated synthesis of nanostructured chiral 2D halide perovskites with amplified chiroptical response

Author(s): Do-Kyoung Lee[1,2], Raphael F. Moral[1,2,3], Finn Babbe[4], Tim Kodalle[2,5], Fabrice Roncoroni[2], Harishankar Jayakumar[2], Kun Zhou[6], Yuanyuan Zhou[6,7,8], Yen Jea Lee[~~6~~ 9], Brooks A. Abel[~~6,7~~ 9,10], Craig P. Schwartz[1,~~8~~ 11], Carolin M. Sutter-Fella[2]*

[1]Department of Physics & Astronomy, University of Nevada, Las Vegas, Las Vegas, Nevada 89154, USA

[2]Molecular Foundry Division, Lawrence Berkeley National Laboratory, 1 Cyclotron Road, Berkeley, California 94720, USA

[3]Brazilian Synchrotron Light Laboratory (LNLS), Brazilian Center for Research in Energy and Materials (CNPEM), Campinas, Sao Paulo 13083-970, Brazil.

[4]Chemical Sciences Division, Lawrence Berkeley National Laboratory, 1 Cyclotron Road, Berkeley, California 94720, USA

[5]Advanced Light Source, Lawrence Berkeley National Laboratory, 1 Cyclotron Road, Berkeley, California 94720, USA

[6]Department of Chemical and Biological Engineering, The Hong Kong University of Science and Technology, Kowloon, Hong Kong SAR, China

[7]Energy Institute, The Hong Kong University of Science and Technology, Hong Kong, China

[8]Otto Poon Centre for Climate Resilience and Sustainability, The Hong Kong University of Science and Technology, Hong Kong, China

[~~6~~ 9]Materials Sciences Division, Lawrence Berkeley National Laboratory, 1 Cyclotron Road, Berkeley, California 94720, USA

[~~7~~ 10]Department of Chemistry, University of California, Berkeley, Berkeley, California 94720, USA

[~~8~~ 11]Advanced Photon Source, Argonne National Laboratory, Lemont, Illinois 60439, USA.

*e-mail: csutterfella@lbl.gov

Here we have addressed the queries from the reviewers and revised the manuscript according to the reviewer's comments. The revised parts are highlighted in yellow in the revised manuscript.

REVIEWER COMMENTS

Reviewer #1 (Remarks to the Author):

The authors have addressed most of my previous comments. However, there are still three issues of this manuscript.

1. It seems that this method is not universal, and only applies to MBA2PbI4 system, which limits its applicability.

Ans) We thank the reviewer for the critical evaluation regarding the applicability of our approach. In this work, we focus on the MBA_2PbI_4 perovskite system because it has been widely used in the research field[1,2] as a representative composition for chiral halide perovskite materials[3,4]. The primary takeaway of our study is that the formation of nanostructured thin films enables the amplified circular dichroism dissymmetry factor. In fact, a study by Hautzinger et al. also shows the importance of exploiting structural features for light polarization control[5].

Although the ionic gel-mediated synthesis route might not be applicable to many chiral spacers e.g. because of solubility constraints, the approach of the nanostructured film formation is transferable across other chiral 2D compositions. Because chemical properties vary, different chiral organic cations and material systems will require different synthesis methods to successfully achieve these nanostructured thin films.

References

[R1] Liu, Y., Shrestha, R., Denisov, K., Ayala, D., Schilfgaarde, M., Nie, W. & Žutić, I . Unconventional spintronics from chiral perovskites. *Adv. Funct. Mater.* **35**(52), e09127 (2025).

[R2] Kwon, B., Park, J., Choi, W., Song, H. & Oh, J. H. Ligand-driven chirality in perovskites for advanced optoelectronics. *ACS Appl. Mater. Interfaces* **17**, 54356-54379 (2025).

[R3] Dilling, D. G. & Lemmerer, A. Synthesis and crystal structures of inorganic-organic hybrids incorporating an aromatic amine with a chiral functional group. *CrystEngComm* **8**, 686-695 (2006).

[R4] Lu, H., Vardeny, Z. V. & Beard, M. C. Control of light, spin and charge with chiral metal halide semiconductors. *Nat. Rev. Chem.* **6**, 470-485 (2022).

[R5] Hautzinger, M. P. *et al.* Control over Banded Morphologies and Circular Dichroism in Chiral Halide Perovskites. *ACS Nano* **19**, 19141–19148 (2025).

We revised the paragraphs in the revised manuscript to reflect the reviewers point. Please see below.

In the Abstract section,

"Chiroptical properties in metal halide perovskites typically arise from asymmetric crystal structures induced by chiral organic molecules during synthesis or chiral additives. However, the synthesis of chiral 2D halide perovskites with sufficiently high dissymmetry factors for practical applications remains challenging. Herein, we present a two-step ionic gel-mediated (IGM) nucleation and recrystallization reaction, enabling the synthesis of nanostructured chiral 2D halide perovskites exhibiting large intrinsic circular dichroism dissymmetry factors (g_{CD}) up to 10^{-2}. The ionic gel mediates the reaction between the PbI_2 and MBAI (MBA = methylbenzylamine) salt, to facilitate the formation of higher-order iodoplumbate PbI_3^- and oligomeric Pb-I-MBA species, which recrystallize during heat treatment as anisotropic nanostructures without preferential

orientation. IGM films exhibit a very pronounced peak splitting originating from a well-defined exciton fine structure and narrow photoluminescence linewidth. This is indicative of ~~stronger~~ the presence of electron-hole exchange interactions and minimized structural disorder in the material leading to significantly amplified chiroptical response. This work demonstrates a synthesis route for nanostructured chiral MBA_2PbI_4 films and how its unique structural features with less crystalline imperfection enable amplified CD response and g_{CD}."

In Conclusion section,

"This study presents an approach to amplify chiroptical response in chiral 2D MBA_2PbI_4 via IGM crystallization. Specifically, a dissymmetry factor g_{CD} of 1.2×10^{-2} with negligible linear dichroism and linear birefringence (LDLB) contribution was measured for the best IGM sample. This is among the highest intrinsic g_{CD} reported, *i.e.*, without any additional chiral additives, which is comparable to their single crystal counterparts as well as inorganic semiconductor clusters (**Supplementary Table 3**). This study leverages *in-situ* GIWAXS and UV-Vis transmission to investigate the IGM crystallization, leading to anisotropic crystal orientation. By mediating the reaction between the PbI_2 and MBAI salt, nanostructured chiral 2D MBA_2PbI_4 with enhanced CD response was observed. CD analysis together with PL at 4 K revealed the origin of amplified CD response and spectral bisignate appearance. A more pronounced exciton fine structure feature for IGM samples is a direct consequence of superior crystallinity and crystallite domain homogeneity. This leads to enhanced rotational transitions owing to strong electrodynamic coupling of transition dipoles. In conclusion, we introduce a strategy to amplify chiroptical properties of chiral 2D MBA_2PbI_4 halide perovskites by tailoring a synthesis route towards nanostructured crystals. Although the ionic gel-mediated synthesis route might not be applicable to many organic spacers due to solubility constraints, the approach of the nanostructured film formation and manipulation of morphological features is transferable across other chiral 2D materials to boost chiroptical properties. Therefore, we believe that our study demonstrates versatility and applicability in its proof-of-concept, establishing a pathway to amplify chiroptical properties through controlled nanostructure formation."

2. It seems that the chiral MBA2PbI4 films, although displaying large gCD, show no CPL or spin-dependent charge transport, which limits their practical applications.

Ans) We would like to start by clarifying that CPL was measured at room temperature in our study. CPL is important for light-emitting applications. The absence of room temperature CPL is, however, not a disqualifying criterion for CISS or other applications. As the reviewer knows, the CPL response is governed by the radiative recombination dynamics of spin-polarized charge carriers. For CPL responses to be observed, the spin lifetime should be longer than the radiative recombination lifetime[5]. In chiral MBA_2PbI_4, spin lifetimes have been reported to be on the order

of a few picoseconds[6,7]. Since spin relaxation occurs much faster than radiative recombination[7], the carriers lose their spin polarization before emission step, resulting in no CPL response. Moreover, because of their soft lattice nature and polycrystalline features, spin relaxation can be significantly attributed to a high frequency of scattering events such as thermal scattering induced by lattice vibration, phonon vibration, and strong electron-phonon coupling, resulting in the loss of spin-dependent properties[8-12]. Therefore, CPL response at room temperature in chiral halide perovskites is noticeably challenging, which is a widespread issue in chiral halide perovskite materials[13,14], not isolated to this work. Just to give an example, Ma et al. investigated temperature dependent CPL in $(R\text{-MBA})_2PbI_4$ and $(S\text{-MBA})_2PbI_4$ microplates and found a monotonous decrease with increasing temperature stating that it “nearly vanishes at room temperature.”[13]. Thus, low or no CPL at room temperature is frequently observed in chiral 2D perovskite films[13,14]. This, however, does not prevent these materials from demonstrating the CISS effect or chiral LEDs[15].

In chiral halide perovskite materials, chirality is a key property because it is directly linked to their coherent spin-dependent characteristics and chiroptical properties based on non-centrosymmetric crystal structures. These chirality-induced properties in the materials have addressed a strong potential for applications such as circularly polarized light photodetector, bio-imaging sensor, 3D displays, memory devices, spintronics, and quantum computing[16-19]. Among characterization tools, circular dichroism (CD) is a common, but highly sensitive characterization technique to measure their chirality and structural properties. The studies have been conducted in varying organic or inorganic semiconductor materials demonstrating chiral-dependent properties by using CD measurements to exhibit the potential for next-generation optoelectronic and spintronic applications, owing to their possibility of having chirality-induced spin selectivity (CISS) effect[20,21]. Although chiroptical activity measurements such as CD and CPL may not grant the presence of spin-dependent properties in the material, the reported calculation and empirical results have reported the significant correlation between CD response and spin splitting[22]. Furthermore, a bisignate CD appearance without magnetic fields results from strong electromagnetic interaction, chiral symmetry breaking, and Cotton effect, which is also related to Rashba-type spin splitting and spin-textures as well as intense chiroptical properties[22-24]. Consequently, CD measurements offer a robust approach to evaluating the material chirality, facilitating a comprehensive and reliable analysis of the associated chirality-induced spin polarization phenomena. As the reviewer mentioned, the emissive optoelectronic device applications such as spin light-emitting iodides (spin-LEDs) may limit to be facilitated in case of no CPL response. However, by amplifying the chirality of intrinsic chiral MBA_2PbI_4 perovskites to achieve an exceptionally strong CD response, this work can demonstrate the strong potential to high-performance of advanced device applications that leverage chirality-induced properties. Also, the improved film coverage and uniformity in IGM samples are a fundamental prerequisite for device applications.

References

[R5] Lu, H., Vardeny, Z. V. & Beard, M. C. Control of light, spin and charge with chiral metal halide semiconductors. *Nat. Rev. Chem.* **6**, 470-485 (2022).

[R6] Dong, Y., Hautzinger, M. P., Haque, M. A. & Beard, M. C. Chirality-induced spin selectivity in hybrid organic-inorganic perovskite semiconductors. *Annu. Rev. Phys. Chem.* **76**, 519-537 (2025).
[R7] Pan, R., Wang, K. & Yu, Z.-G. Magnetic-field manipulation of circularly polarized photoluminescence in chiral perovskites. *Mater. Horiz.* **9**, 740-747 (2022).
[R8] Xu, J., Li, K., Huynh, U. N., Fadel, M., Huang, J., Sundararaman, R., Vardeny, V. & Ping Y. How spin relaxes and dephases in bulk halide perovskites. *Nat. Commun.* **15**, 188 (2024).
[R9] Qin, Y. *et al.* Enhancing room-temperature spin lifetimes in molecular semiconductors by designing intramolecular dipole orientations. *Adv. Mater.* **37**(28), 2500521 (2025).
[R10] Quite, C. & Venkataraman, V. Temperature dependence of spin lifetime of conduction electrons in bulk germanium. *Appl. Phys. Lett.* **101**, 252404 (2012).
[R11] Kiss, A., Szolnoki, L. & Simon, F. The Elliott-Yafet theory of spin relaxation generalized for large spin-orbit coupling. *Sci. Rep.* **6**, 22706 (2016).
[R12] Tao, W., Zhou, Q. & Zhu, H. Dynamic polaronic screening for anomalous exciton spin relaxation in two-dimensional lead halide perovskites. *Sci. Adv.* **6**, eabb7132 (2020).
[R13] Ma, J., Fang, C., Chen, C., Jin, L., Wang, J., Tang, J. & Li D. Chiral 2D perovskites with a high degree of circularly polarized photoluminescence. ACS Nano, 13(3), 3659-3665 (2019).
[R14] Liu, S. *et al*. Bright circularly polarized photoluminescence in chirallayered hybrid lead-halide perovskites. *Sci. Adv.* **9**, eadh5083 (2023).
[R15] Kim, Y.-H. et al. Chiral-induced spin selectivity enables a room-temperature spin light-emitting diode. *Science* **371**(6534), 1129-1133 (2021).
[R16] Long, G., Sabatini, R., Saidaminov, M., Lakhwani, G., Rasmita, A., Liu, X., Sargent, E. H. & Gao, W. Chiral-perovskite optoelectronics. *Nat. Rev. Mater.* **5**, 423 (2020).
[R17] Dang, Y., Liu, X., Cao, B. & Tao, X. Chiral halide perovskite crystals for optoelectronic applications. *Matter* **4**, 794-820 (2021).
[R18] Liu, Y., Shrestha, R., Denisov, K., Ayala, D., Schilfgaarde M., Nie, W. & Žutić, I. Unconventional spintronics from chiral perovskites. *Adv. Funct. Mater.* **35**(52), e09127 (2025).
[R19] Ding, Z., Chen, Q., Jiang, Y. & Yuan, M. Structural-guided approaches for enhanced spin-splitting in chiral perovskite. *J. Am. Chem. Soc. Au.* **4**, 1263-1277 (2024).
[R20] Ugras, T. J. et al. Transforming achiral semiconductors into chiral domains with exceptional circular dichroism. *Science* **387**, 490 (2025).
[R21] Wade, J. et al. Controlling anisotropic properties by manipulating the orientation of chiral small molecules. *Nat. Chem.* **14**, 1383-1389 (2022).
[R22] Sercel, P. C., Hautzinger, M. P., Song, R., Blum, V. & Beard, M. C. Optical activity of chiral excitons. *Adv. Mater.* **37**, 2415901 (2025).
[R23] Ma, S. et al. Elucidating the origin of chiroptical activity in chiral 2D perovskites through nano-confined growth. *Nat. Commun.* **13**, 3259 (2022).
[R24] Kim, H. et al. Giant chiral amplification of chiral 2D perovskites via dynamic crystal reconstruction. *Sci. Adv.* **10**, eado5942 (2024).

3. The authors stated that the surface roughness was improved compared to the control sample. However, how was the control film fabricated? Literature reports have shown a smooth surface of chiral MBA2PbI4 film prepared by the traditional one-step method.
Can the authors further address or discuss these points?

Ans) We thank the reviewer for raising this point. We would like to begin the response with a clarification: the 'traditional one-step method' for 2D chiral perovskites is oftentimes based on dissolving single crystals to fabricate thin films. In our study, we use commercial lead iodide and synthesized MBAI precursors and refer to this as a one-step method. To thoroughly address the reviewer's point, we conducted additional experiments and formed *S*-MBA_2PbI_4 perovskite films via the one-step deposition method. The precursor coating solutions were prepared using acetonitrile (ACN) and *N*,*N*-dimethylformamide (DMF) as solvents, separately. Using volatile solvents such as ACN facilitates highly uniform and smooth halide perovskite films to promote rapid crystallization[25-28]. As can be seen in **Fig. R1**, we observed that ACN-based film shows smoother and fully-covered morphology compared to DMF-based films. To rule out thickness-dependent artifacts we formed chiral halide perovskite films across varying coating solution concentrations. Thickness-dependent morphological observations and chiroptical properties are illustrated in **Fig. R2**. Cross-sectional SEM images (**Fig. R2a-h**) exhibit smooth and uniform surface morphology for ACN-based films. In contrast, DMF-based films show poor surface morphology compared to ACN-based films, which further exacerbated as the solution concentration increased. The corresponding chiroptical characteristics are shown in **Fig R2i-j**. Overall CD response and g_{CD} at 498 nm (**Fig. R2k,l**) for ACN-based films are higher than those of DMF-based films. This indicates that the improved uniformity and smoothness of chiral halide perovskite films can lead to higher CD response and g_{CD}.

As the reviewer mentioned, using traditional one-step deposition method with precursor solution engineering enables the formation of highly uniform and smooth chiral halide perovskite films, driving improved chiroptical performance[27,28]. However, despite high quality thin films, chiroptical response for ACN-based films is still low (CD < 100 mdeg, $g_{CD} < 10^{-3}$) compared to IGM films (CD > 600 mdeg, $g_{CD} > 10^{-2}$). This indicates that merely reducing film roughness is not sufficient to significantly improve the chiroptical response.

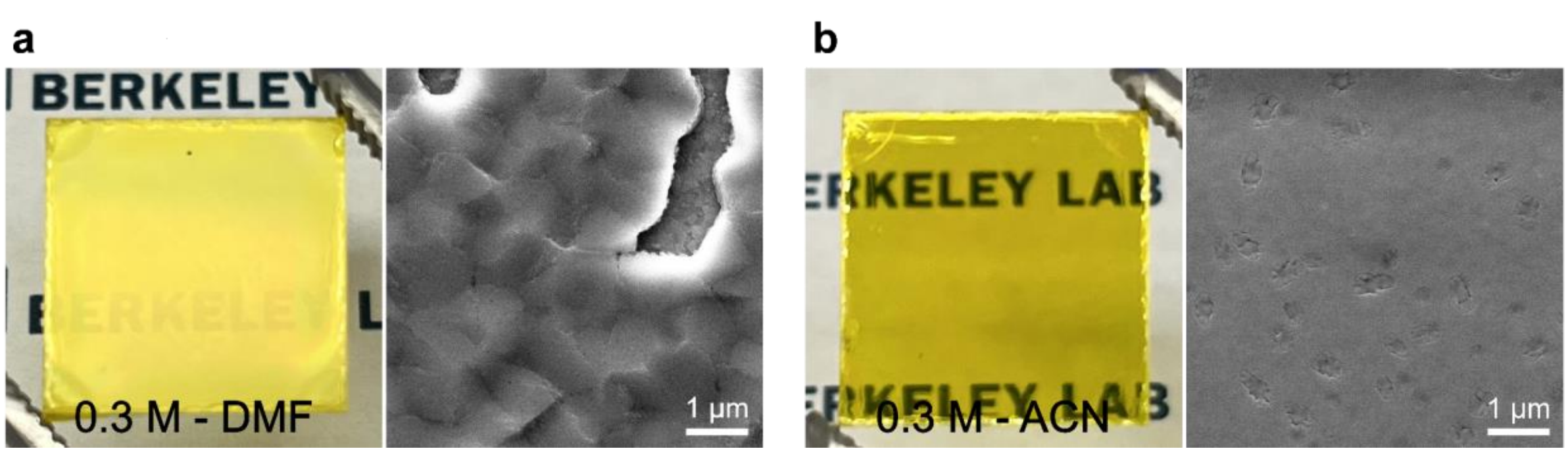

Fig. R1. **a,b**, (left) photographs and top-view SEM images for *S*-MBA_2PbI_4 perovskite films using (**a**) DMF and (**b**) ACN solvents via a one-step deposition method. The transparency indicates the film coverage and surface roughness.

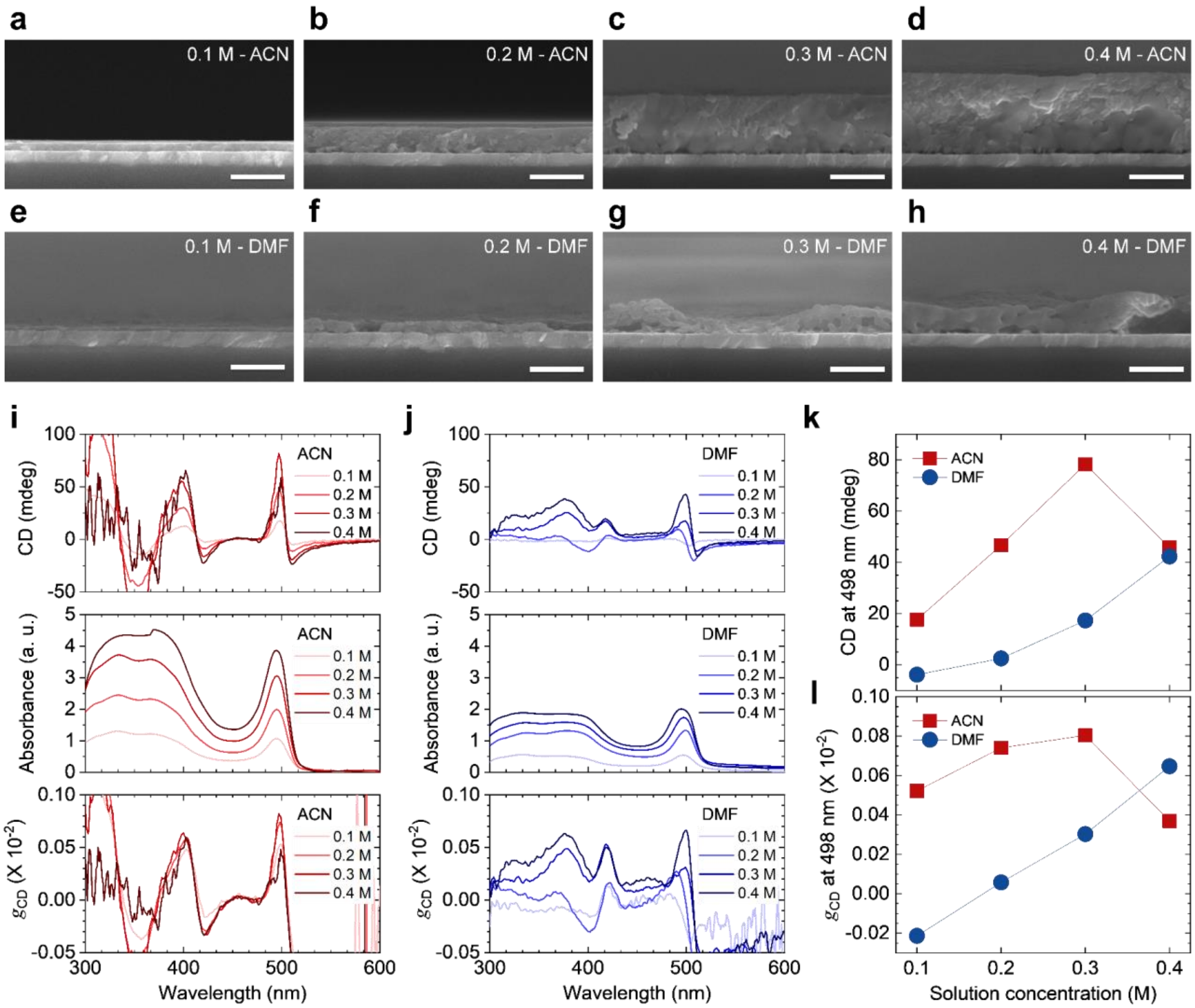

Fig. R2. Morphological and chiroptical characteristics of *S*-MBA_2PbI_4 halide perovskite films with different concentrations (0.1 ~ 0.4 M) and solvent system (ACN, DMF) of precursor coating solution. **a-h**, Cross-sectional SEM images for (**a-d**) ACN-based and (**e-h**) DMF-based films. The scale bar is 500 nm. **i-j**, CD, absorbance, and g_{CD} spectra for the corresponding films. **k,l**, CD and g_{CD} values at 498 nm as a function of the precursor solution concentration.

We made several additions to the revised manuscript. Also, **Fig. R1** and **R2** are added into the revised SI as **Supplementary Fig. 21** and **22**.

"We hypothesize that there are two major factors contributing to the amplified CD response in IGM films: the enhanced light-matter interaction via textured morphology with randomly oriented

crystals as well as enhanced electrodynamic coupling of transition dipoles. First, these isotropic distributions of crystalline domains can substantially contribute to the enhanced CD response. When circularly polarized light passes the textured or patterned film, the incident light can be refracted depending on the crystal's geometry[22]. When the circularly polarized light passes through randomly oriented crystals, strong and highly efficient light-matter interactions occur by coupling the electric field vector of the light with a more diverse population of crystalline domains considering all possible interaction geometries[22,42]. Therefore isotropic orientation of nanorods leads to more intense CD response. To further investigate the influence of the structural features of nanostructured films on CD response, we prepared the films via one-step deposition method using acetonitrile (ACN)- and N,N-dimethylformamide (DMF)-based S-MBA_2PbI_4 precursor solutions. While poor coverage and rough surface are observed in the DMF film, the ACN film shows highly semi-transparent, uniform, and fully-covered morphology (**Supplementary Fig. 21**). The corresponding CD response is shown in **Supplementary Fig. 22**. Although the CD response intensity and g_{CD} for ACN films are enhanced owing to better coverage and surface morphology, those values are still low (CD < 100 mdeg, $g_{CD} < 10^{-3}$) compared to IGM films (CD > 600 mdeg, $g_{CD} > 10^{-2}$). This indicates that reducing film roughness and improving coverage are not sufficient to significantly improve the chiroptical response."

References

[R25] Noel, N. K., Habisreutinger S. N., Wenger B., Klug, M. T., Hörantner, M. T., Johnston, M. B., Nicholas, R. J., Moore, D. T. & Snaith, H. J. A low viscosity, low boiling point, clean solvent system for the rapid crystallization of highly specular perovskite films. *Energy Environ. Sci.* **10**, 145-152 (2017).

[R26] Deng, Y., Van Brackle, C. H., Dai, X., Chen, B. & Huang J. Tailoring solvent coordination for high-speed, room-temperature blading of perovskite photovoltaic films. *Sci. Adv.* **5**(12), aax7537 (2019).

[R27] Scalon, L. *et al*. Tuning phase purity in chiral 2D perovskites. *Adv. Optical Mater.* **12**(2), 2300776 (2024).

[R28] Moral, R. F. *et al*. Absorption dissymmetry factor enhancement: A data-driven approach to unravel the synthesis knobs of chiral 2D perovskites. *Matter* **9**(5), 102676 (2026).

Reviewer #2 (Remarks to the Author):

The authors have responded thoroughly and convincingly to most of the reviewers' comments, and the manuscript has been significantly improved through the necessary revisions and clarifications. Only minor editorial adjustments—such as copyediting for language style and a final verification of figure captions—may be needed at the proof stage. Nevertheless, I would like to request that the following two points be addressed before the paper is considered for publication:

1. The link between enhanced crystallinity/morphology and amplified Rashba splitting is plausible but indirect. Can the authors provide even qualitative theoretical support (e.g., a simple model or calculation) to strengthen this key claim?

Ans) We thank the reviewer for the constructive comments. To further solidify our discussion regarding the link between crystalline imperfections and CD response in *S*-MBA_2PbI_4 films, we calculated defect-induced octahedral distortions in our material system and its electronic band structure based on the PBE-SOC model[29].

To quantify the octahedral distortion, the angular difference in the calculated tilt angles between an inequivalent pair of neighboring octahedra is estimated, denoted as $\Delta\boldsymbol{\beta}$. This parameter indicates structural chirality induced by spatial inversion symmetry-breaking of the octahedral framework owing to the chiral organic ligands, which is linked to their chirality-induced electronic and optical properties[29-32]. For example, **Fig. R3** illustrates the computational results with the comparison of $\Delta\boldsymbol{\beta}$ between racemic- and *S*-MBA_2PbI_4 chiral perovskites. It reveals a larger angular difference in Pb-I-Pb tilt angles for *S*-MBA_2PbI_4 (6.47°) than racemic (1.60°), indicating the pronounced octahedral distortion and loss of centrosymmetry in *S*-MBA_2PbI_4. This structural symmetry breaking directly governs its CD response. Furthermore, higher bonding angle tilting enables significant Rashba spin splitting manifestation in the calculated band structure, implying high chirality-induced spin properties.

Regarding the influence of defect-induced structural features on chiroptical properties, CD spectra and electrical band structures depending on defect conditions are calculated (**Fig. R4**), where vacancies (V_I, V_{Pb}, and V_{MBA}) are considered as representative defects. **Table R1** shows the calculated $\Delta\boldsymbol{\beta}$ values for various defect types. Given the decreased $\Delta\boldsymbol{\beta}$ values for defect-induced conditions (6.01° for V_I, 4.73° for V_{Pb}, 5.11° for V_{MBA}, 4.84° for I_i, and 4.38° for Pb_i), the presence of defects can attenuate the local geometric asymmetry, leading to loss of its chirality. The calculation results of density of states with varying defect conditions are shown in **Fig. R4e-h**. Since Rashba spin splitting relies on inversion symmetry breaking of crystal structures, defect-induced structural asymmetry relaxation corresponds directly to a reduction in energy level splitting, as observed in **Fig. R4e-h**. In addition, these findings in the calculated density of states provide an explanation for a strong bisignate CD appearance in IGM films, indicating that the CD features result from less defective materials in this case. Furthermore, CD spectrum simulations corroborate that the chiroptical response is attenuated in the presence of defects due to the reduced structural asymmetry, which is good agreement with the calculated $\Delta\boldsymbol{\beta}$ results.

References

[R29] Masud, M. M., Viera, J., Ben-Akacha, A., Ma, B. & Strubbe, D. A. Computational and experimental investigation of chiral and achiral two-dimensional organic lead bromide perovskites: octahedral distortions and electronic and optical properties. *ChemPhysChem* **26**, e202500423 (2025).

[R30] Son, J. et al. Unraveling chirality transfer mechanism by structural isomer-derived hydrogen bonding interaction in 2D chiral perovskite. *Nat. Commun.* **14**, 3124 (2023).
[R31] Kim, Y.-H. et al. The structural origin of chiroptical properties in perovskite nanocrystals with chiral organic ligands. *Adv. Funct. Mater.* **32**, 2200454 (2022).
[R32] Liu, Q., Ren, H., Wei, Q. & Li, M. Multifunctional chiral halide perovskites: Advancing chiro-optics, chiro-optoelectronics, and spintronics. *Adv. Sci.* **12**, e09155 (2025).

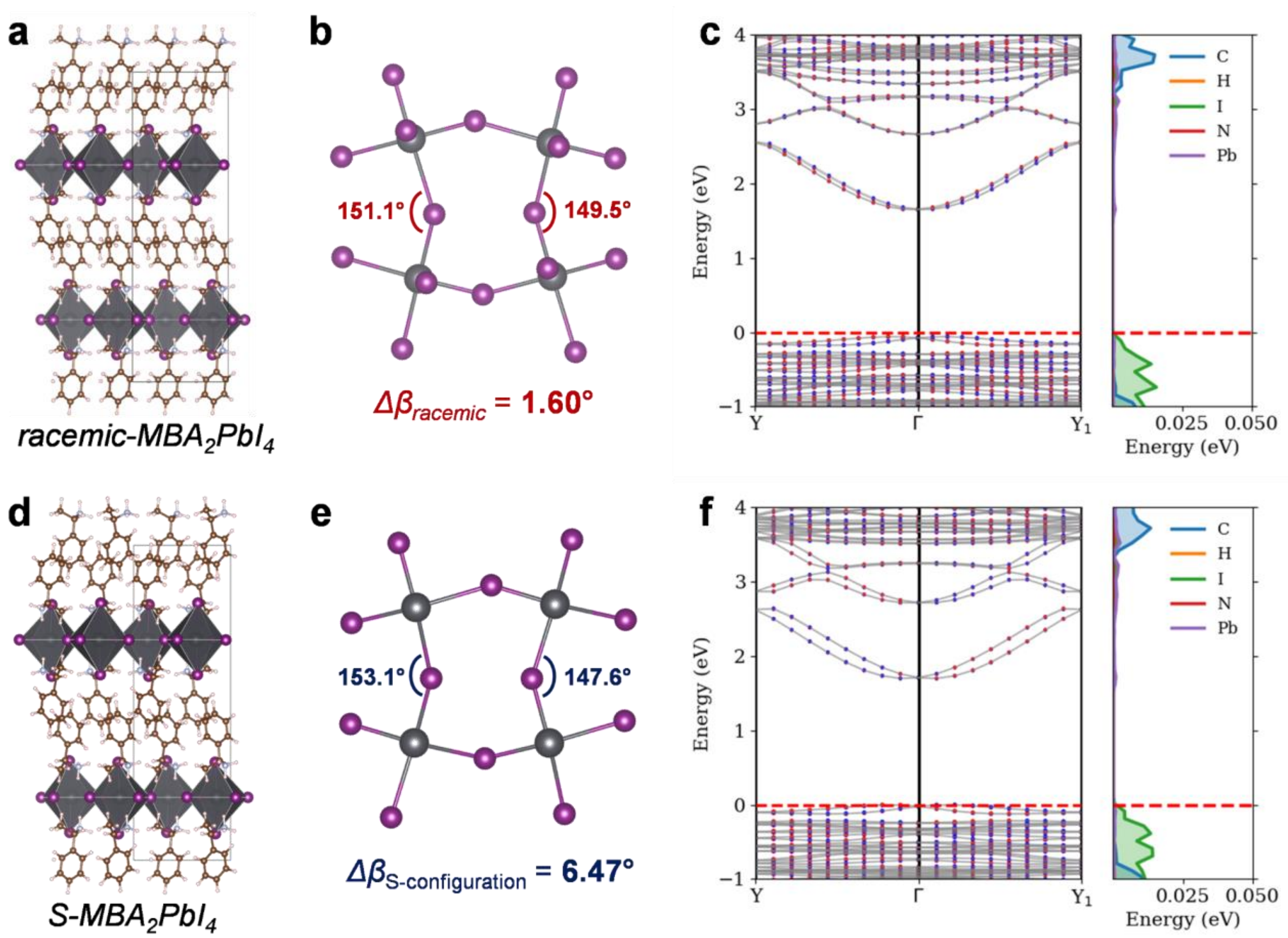

Fig. R3. Crystal structure, scheme of tilted octahedral frameworks caused by chiral organic species, and calculated band structures for racemic-MBA_2PbI_4 (top, a–d) and S-MBA_2PbI_4 (bottom, e–h). $\boldsymbol{\Delta\beta}$ represents the tilt angle discrepancy between an inequivalent pair of neighboring octahedra.

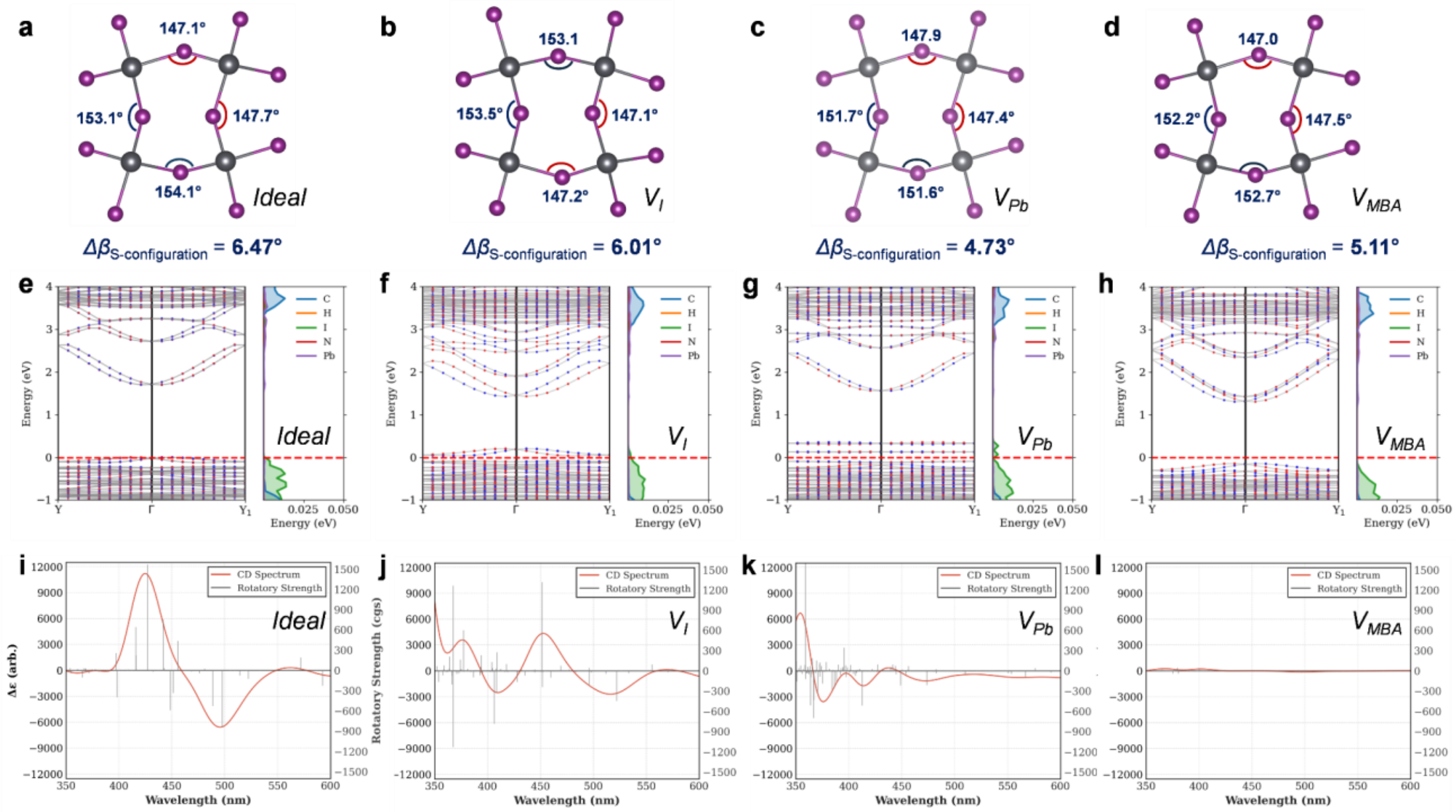

Fig. R4. Calculated band structures (top row, a–d), Scheme of tilted octahedral frameworks (middle row, e–h), and CD spectra (bottom row, i–l) for ideal structure and structures with defects: V_I (iodine vacancy), V_{Pb} (lead vacancy), and V_{MBA} (*S*-MBA vacancy). ***Δβ*** represents the tilt angle discrepancy between an inequivalent pair of neighboring octahedra.

Regarding DFT calculations, we added the following sections in the revised manuscript as well as details in the Methods. Also, we added **Fig. R4** as **Supplementary Fig. 23** in the revised SI. Please see below.

In the revised manuscript,

"To support this correlation, density functional theory (DFT) calculations were carried out, to quantify octahedral distortion, where the angular difference of Pb-I-Pb bonding between an inequivalent pair of neighboring octahedra was denoted as ***Δβ*** (see **Supplementary Fig. 23)**. The calculated ***Δβ*** values for various defect types are summarized in **Supplementary Table 1**. Given the decreased ***Δβ*** values for defect-induced conditions (6.01° for V_I, 4.73° for V_{Pb}, 5.11° for V_{MBA}, 4.84° for I_i, and 4.38° for Pb_i), the presence of defects can attenuate the local geometric asymmetry, leading to loss of its chirality. The density of states calculation results with varying defect conditions are shown in **Supplementary Fig. 23e-h**. Since Rashba spin splitting relies on inversion symmetry breaking of crystal structures, defect-induced structural asymmetry relaxation corresponds directly to a reduction in energy level splitting, as observed in **Supplementary Fig. 23e-h**. In addition, these findings in the calculated density of states provide an explanation for a strong bisignate CD appearance in IGM films, indicating that the CD features result from less

defects. Furthermore, CD spectra simulations (**Supplementary Fig. 23i-l**) corroborate that the chiroptical response is attenuated in the presence of defects due to the reduced structural asymmetry, which is good agreement with the calculated ***Δβ*** results. The correlation between the crystallite homogeneity and the amplified CD response is further discussed in the next section based on temperature-dependent PL linewidth analysis."

In the Methods section,

DFT calculation

First-principles calculations were performed within the framework of density functional theory (DFT) using the plane-wave pseudopotential method as implemented in the Vienna Ab initio Simulation Package (VASP)[55]. The generalized gradient approximation (GGA) formulated by Perdew, Burke, and Ernzerhof (PBE) was employed as the exchange–correlation functional[56]. Electron–ion interactions were described by the projector augmented wave (PAW) method[57]. A plane-wave cutoff energy of 520 eV was used to expand the Kohn–Sham orbitals. A Gamma-centered k-point mesh of 2 × 2 × 1 was adopted for electronic Brillouin-zone integration. Equilibrium structural parameters (including lattice parameters and internal coordinates) for each involved bulk material were obtained by total energy minimization using the conjugate-gradient algorithm, with force and energy convergence thresholds of 0.05 eV $Å^{-1}$ and 1×10^{-5} eV, respectively. To properly account for the non-negligible long-range van der Waals interactions in hybrid perovskites containing organic molecules, the DFT-D3 van der Waals correction was included. Band structure calculations incorporating spin-orbit coupling were performed to evaluate the Rashba splitting strength. The electronic circular dichroism (ECD) calculations were performed using the GPAW package with the LCAO mode[58-59]. The PBE functional within the GGA framework was used for the exchange-correlation functional, and the PAW method was used for core-valence interactions. A real-space grid spacing of 0.2 Å was employed. DZP basis sets were used for all atoms (C, H, N, Pb, I)[60]. The SCF density convergence criterion was set to eV. The number of empty bands was automatically determined based on the number of occupied orbitals, with an additional 12 bands to ensure convergence. The LCAO-based RT-TDDFT implementation in GPAW was used for the time propagation[61]. Electron dynamics were initiated by an electric kick perturbation of a.u. under the velocity gauge, using the adiabatic PBE kernel, a time step of 10 as, and a total propagation time of 20 fs.

References

55. Kresse, G. & Furthmu¨ller, J. Efficient iterative schemes for ab initio total-energy calculations using a plane-wave basis set. *Phys. Rev. B* 54(16), 11169 (1996).

56. Perdew, J. P., Burke, K. & Ernzerhof, M. Generalized Gradient Approximation Made Simple. Phys. Rev. Lett. 77(18), 3865 (1996).

57. Blöchl, P. E. Projector augmented-wave method. *Phys. Rev. B* 50, 17953 (1994).

58. Mortensen, J. J. et al. GPAW: An open Python package of electronic structure calculations. *J. Chem. Phys.* 160, 092503 (2024).

59. Larsen A. H., Vanin, M., Mortensen, J. J., Thygesen, K. S. & Jacobsen K. W. Localized atomic basis set in the projector augmented wave method. *Phys. Rev. B* 80, 195112 (2009).

60. Rossi, T. P., Lehtola, S., Sakko, A., Puska, M. J. & Nieminen, R. M. Nanoplasmonics simulations at the basis set limit through completeness-optimized, local numerical basis sets. *J. Chem. Phys.* 142(9), 094114 (2015).

61. Rossi, T. P., Kuisma, M., Puska, M. J., Nieminen, R. M. & Erhart, P. Kohn-Sham Decomposition in Real-Time Time-Dependent Density-Functional Theory: An Efficient Tool for Analyzing Plasmonic Excitations. *J. Chem. Theory Comput.* 13(10), 4779-4790 (2017).

In the revised SI,

Supplementary Table 1 Quantitative comparison of calculated defect-induced octahedral distortion in *S*-MBA_2PbI_4 crystal structure with 2 × 2 × 1 supercell. $\Delta\boldsymbol{\beta}$ represents the angular difference in Pb-I-Pb tilt angle between an inequivalent pair of neighboring octahedra. Ideal refers to a defect-free crystal structure.

Type of defects	$\Delta\boldsymbol{\beta}$ (°)
Ideal	6.47
V_I	6.01

V_{Pb}	4.73
V_{MBA}	5.11
I_i	4.84
Pb_i	4.38

2. While reproducibility across batches is shown, how many spots per film were measured for gCD? Please confirm the signal uniformity across the nanostructured film to rule out local artifacts.

Ans) That's a good catch regarding signal uniformity. We confirmed that the incident beam spot size on the film samples for CD measurements is around 0.6 × 0.7 cm^2. Given the total dimensions of our sample area is 1.5 × 1.5 cm^2, the beam covers a substantial macroscopic area of the nanostructured film during CD measurement. Since the beam illuminates a large portion of the film samples, we believe that the CD measurements inherently averages out microscopic variations and reasonably rules out the possibility that the observed CD signals originate from isolated local artifacts.

We added information regarding the incident beam spot size for CD measurements in the Characterization section in the revised manuscript. Please see below.

"Circular dichroism (CD) and absorbance spectra were measured using a J-815 Jasco spectropolarimeter. The incident beam spot size on the film samples for CD measurements is around 0.6 × 0.7 cm^2. In this work, all CD, absorbance, and g_{CD} spectra are demonstrated with genuine values."

<u>Reviewer #2 (Remarks on code availability)</u>:

While I did not personally run the code, the provided documentation—especially the step-by-step setup guide and usage description—suggests that a user could reasonably install and execute it. Thus, the results of the associated paper are likely reproducible with this resource.

Ans) We sincerely thank the reviewer for the positive feedback and were very glad to hear that you liked the step-by-step setup guidance in the documentation.

Reviewer #3 (Remarks to the Author):

The authors addressed all the comments. Now the paper is ready for publication.

Ans) We sincerely thank the reviewer for the positive recommendation to publish the work as is.

A point-by-point response

Journal: Nature Communications
Manuscript ID: NCOMMS-25-86761B
Title: Ionic gel-mediated synthesis of nanostructured chiral 2D halide perovskites with amplified chiroptical response
Author(s): Do-Kyoung Lee[1,2], Raphael F. Moral[1,2,3], Finn Babbe[4], Tim Kodalle[2,5], Fabrice Roncoroni[2], Harishankar Jayakumar[2], Kun Zhou[6], Yuanyuan Zhou[6,7,8], Yen Jea Lee[9], Brooks A. Abel[9,10], Craig P. Schwartz[1,11], Carolin M. Sutter-Fella[2]*

[1]Department of Physics & Astronomy, University of Nevada, Las Vegas, Las Vegas, Nevada 89154, USA

[2]Molecular Foundry Division, Lawrence Berkeley National Laboratory, 1 Cyclotron Road, Berkeley, California 94720, USA

[3]Brazilian Synchrotron Light Laboratory (LNLS), Brazilian Center for Research in Energy and Materials (CNPEM), Campinas, Sao Paulo 13083-970, Brazil.

[4]Chemical Sciences Division, Lawrence Berkeley National Laboratory, 1 Cyclotron Road, Berkeley, California 94720, USA

[5]Advanced Light Source, Lawrence Berkeley National Laboratory, 1 Cyclotron Road, Berkeley, California 94720, USA

[6]Department of Chemical and Biological Engineering, The Hong Kong University of Science and Technology, Kowloon, Hong Kong SAR, China

[7]Energy Institute, The Hong Kong University of Science and Technology, Hong Kong, China

[8]Otto Poon Centre for Climate Resilience and Sustainability, The Hong Kong University of Science and Technology, Hong Kong, China

[9]Materials Sciences Division, Lawrence Berkeley National Laboratory, 1 Cyclotron Road, Berkeley, California 94720, USA

[10]Department of Chemistry, University of California, Berkeley, Berkeley, California 94720, USA

[11]Advanced Photon Source, Argonne National Laboratory, Lemont, Illinois 60439, USA.

*e-mail: csutterfella@lbl.gov

REVIEWER COMMENTS

Reviewer #1 (Remarks to the Author):

The authors have addressed my comments. Although the strategy is limited to a specific composition and the resulting chiral 2D perovskite displays no CPL, the enhancement of CD is quite interesting.

Ans) We thank the reviewer for constructive feedback throughout the revision steps and for recognizing the value of our work. Although we agree with these specific limitations regarding the

compositional generalization with IGM synthesis technique and the absence of CPL, we believe the significant enhancement of the circular dichroism (CD) response could be an interesting and valuable contribution to the research field.

Reviewer #2 (Remarks to the Author):
The authors have carefully addressed all comments raised in a convincing manner. Therefore, I would like to recommend publication of this paper.

Ans) We sincerely thank the reviewer for their thorough evaluation of our revisions and for the positive recommendation to publish this work. We deeply appreciate the time and effort you have dedicated to reviewing our manuscript, and your constructive comments (especially in terms of DFT calculation) have been invaluable in improving the quality of our paper.
